# Progression of Type 1 Diabetes: Circulating MicroRNA Expression Profiles Changes from Preclinical to Overt Disease

**DOI:** 10.1155/2022/2734490

**Published:** 2022-07-19

**Authors:** Aritania Sousa Santos, Ludmila Rodrigues Pinto Ferreira, Amanda Cabral da Silva, Laís Isidoro Alves, Jullian Gabriel Damasceno, Leslie Kulikowski, Edecio Cunha-Neto, Maria Elizabeth Rossi da Silva

**Affiliations:** ^1^Laboratorio de Carboidratos e Radioimunoensaios (LIM 18) Faculdade de Medicina FMUSP, Universidade de Sao Paulo, Sao Paulo, SP, Brazil; ^2^RNA Systems Biology Laboratory (RSBL), RNA Systems Biology Laboratory, Department of Morphology, Institute of Biological Sciences, Federal University of Minas Gerais (UFMG), Belo Horizonte, MG, Brazil; ^3^Division of Clinical Immunology and Allergy, School of Medicine, University of Sao Paulo, Sao Paulo, Brazil; ^4^Laboratory of Cytogenomics and Molecular Pathology-Department of Pathology, Faculty of Medicine of the University of Sao Paulo, Brazil; ^5^Laboratory of Immunology, Heart Institute, School of Medicine, University of São Paulo, São Paulo, Brazil Institute for Investigation in Immunology (iii) INCT, São Paulo, Brazil; ^6^Hospital das Clinicas HCFMUSP, Faculdade de Medicina, Universidade de Sao Paulo, Sao Paulo, SP, Brazil

## Abstract

**Objectives:**

To evaluate the potential biological involvement of miRNA expression in the immune response and beta cell function in T1D.

**Methods:**

We screened 377 serum miRNAs of 110 subjects divided into four groups: healthy individuals (control group) and patients at different stages of T1D progression, from the initial immunological manifestation presenting islet autoantibodies (AbP group) until partial and strong beta cell damage in the recent (recent T1D group) and long-term T1D, with 2 to 5 years of disease (T1D 2-5y group).

**Results:**

The results revealed 69 differentially expressed miRNAs (DEMs) in relation to controls. Several miRNAs were correlated with islet autoantibodies (IA2A, GADA, and Znt8A), age, and C-peptide levels, mainly from AbP, and recent T1D groups pointing these miRNAs as relevant to T1D pathogenesis and progression. Several miRNAs were related to metabolic derangements, inflammatory pathways, and several other autoimmune diseases. Pathway analysis of putative DEM targets revealed an enrichment in pathways related to metabolic syndrome, inflammatory response, apoptosis and insulin signaling pathways, metabolic derangements, and decreased immunomodulation. One of the miRNAs' gene targets was DYRK2 (dual-specificity tyrosine-phosphorylation-regulated kinase 2), which is an autoantigen targeted by an antibody in T1D. ROC curve analysis showed hsa-miR-16 and hsa-miR-200a-3p with AUCs greater than for glucose levels, with discriminating power for T1D prediction greater than glucose levels. *Conclusions/Interpretation.* Our data suggests a potential influence of DEMs on disease progression from the initial autoimmune lesion up to severe beta cell dysfunction and the role of miRNAs hsa-miR-16 and hsa-miR-200a-3p as biomarkers of T1D progression.

## 1. Introduction

miRNAs are small noncoding RNAs functioning as posttranscriptional regulators of gene expression, affecting cell proliferation, differentiation, apoptosis, metabolism, and immunity. They can be released actively by cells or during tissue damage and have been used as biomarkers of destruction or regeneration of beta cells and of altered immunological activity, revealing mechanisms underlying the pathophysiology of type 1 diabetes [1–3]. A previous work from our group revealed the potential role of circulating miR-101 in its pathogenesis [4]. However, there are still contradictory data comparing miRNA profiles from individuals at different stages of diabetes, from preclinical to recent and long-duration T1D. Furthermore, the same miRNAs were shown to be up- or downregulated in the same phase of the disease and to be protective or at risk for diabetes. There is no clear definition about the effects of age, diabetes duration, and glucose levels, considering that metabolic derangements caused by glucolipotoxicity and inflammatory cytokines can change the miRNA milieu and interfere with the results [5–7]. Here we investigated whether there is a differential profile of serum miRNAs at different stages of T1D, which could suggest their participation in its pathogenesis considering these variables. We covered the phases of the highly active autoimmune process and those subject to glucolipotoxicity effects, e.g., from the first autoimmune manifestations (islet autoantibodies) without diabetes to recent and long-duration T1D. Additional information can come from the admixed populations of Brazil, expressing different frequencies of genetic markers of T1D [8].

## 2. Methods

The study was approved by the Ethical Committee of Hospital das Clinicas, Faculdade de Medicina, Universidade de São Paulo (Cappesq 11601), and followed the guidelines of the Declaration of Helsinki. Informed consent was obtained from all patients. Collected serum samples were stored at −80°C until use. Hemolyzed samples were excluded.

### 2.1. Experimental Design

We screened 377 serum miRNAs of 110 subjects divided into four groups according to ADA criteria [9]: individuals with islet autoantibodies without diabetes (AbP group; *n* = 25), newly diagnosed patients with T1D with duration ≤6 months (recent T1D group; *n* = 30), patients with T1D with 2 to 5 years of duration (T1D 2-5y group; *n* = 26), and islet autoantibody negative healthy individuals (control group; *n* = 29). Exclusion criteria comprised other types of diabetes, use of medications except insulin, a febrile state within 10 days prior to blood collection, and individuals with liver, kidney, thyroid, and inflammatory/autoimmune diseases.

Demographic characteristics, such as age, self-reported skin color, and sex were similar between groups (Table 1). Patients with T1D (both recent and lasting 2-5 years) were similar to each other and differed from the control group by higher values of glucose, HbA1c, islet autoantibody, and lower C-peptide levels (*p* < 0.05). AbP group presented intermediate characteristics (lower HbA1c and IA2A and higher C-peptide levels than both groups with T1D) and differed from the controls by higher IAA and GADA levels. HLA high-risk alleles for diabetes (DR3/DR4; DQ2/DQ8) were less frequent in the control group (*p* < 0.05).

### 2.2. RNA Analysis

RNA/miRNAs were isolated from 200 *μ*L serum samples using the miRNeasy Serum/Plasma kit (Qiagen, Hilden, Germany), and the reverse transcription reaction was performed using Megaplex™ RT Human Pool A (Thermo Fisher, USA), TaqMan® MicroRNA Reverse Transcription Kit (Applied Biosystems, Foster City, California, USA), according to manufacturer's instructions. The RT products were preamplified according to the manufacturer's protocol—Megaplex™ pool for microRNA Expression Analysis (Thermo Fisher, USA). Real-Time RT-PCR was performed using the TLDA TaqMan® Low Density Array-Card A v2.0 for humans (384 microRNAs), according to manufacturer's instructions, on the QuantStudio12K Flex (Applied Biosystems, Foster City, California, USA).

### 2.3. MiRNAs Target Prediction and Pathway Analyses

The potential targets of the differentially expressed miRNAs (DEMs) were predicted using miRWalk v2.0, 2018 (http://zmf.umm.uni-heidelberg.de/mirwalk2), TargetScan Human v7.2, 2018 (https://www.targetscan.org/vert_72/), and IPA software (Ingenuity® Pathways Analysis-5.0; Ingenuity Systems, Qiagen, USA). Pathway enrichment analysis was performed using IPA and KEGG database (https://www.genome.jp/kegg/tool/map_pathway1.html), considering only experimentally validated DEM targets and prioritized targets related to beta cell function and autoimmune manifestations.

### 2.4. Glucose, HbA1c, C-Peptide, and Autoantibody Levels

Fasting glucose levels were determined by enzymatic colorimetric assay (LABTEST GOD-ANA, SP, Brazil), HbA1c by HPLC, and C-peptide levels by radioimmunoassay (HCP20K, Millipore Corporation, Billerica, MA, USA; normal values>0.5 ng/mL; intra- and interassay CVs: 4.5% and 9.3%, respectively). IAA, GADA, and IA2A levels were determined by radioimmunoassay (RSR limited, High Bentham, Lancaster, UK; CV<7%). The normal values for 700 healthy controls (3 SD) were <100nU/mL, <25 IU/mL, and <125 IU/mL, respectively. ZnT8A levels were measured by ELISA (KR770-96; Kronus, Boise, Idaho, USA; CV<7%). The normal value in 321 healthy controls was ≤16 IU/mL (3 SD).

### 2.5. Statistical Analysis

The analysis of miRNAs expression used the Cloud program (Thermo Fisher Scientific, Waltham, MA, USA), an online data analysis software of the C*τ* comparative method [10]. The threshold was manually aligned. The chosen criteria for the validation of qRT-PCR reactions were exponential and plateau amplification curves. miRNAs with Ct up to 35 were selected. The relative expression of miRNAs was obtained by the comparative method of Ct (2^-*ΔΔ*Ct^), using global normalization and Benjamini and Hochberg's false discovery rate method, and represented as fold change (FC) in relation to controls. Values >1 were considered increased and < 1 decreased. Variable distributions were verified by the Shapiro–Wilk normality test. Numerical variables with parametric and nonparametric distributions were analyzed by ANOVA and Kruskal–Wallis with Tukey's or Dunn's multiple comparisons posttest, respectively. Correlations were performed using the Spearman correlation test. Qualitative variables were compared using chi-square test or Fisher's exact test (statistical package GraphPad Prism, La Jolla, CA, USA). Data were considered significant at *p* < 0.05. Fisher exact test and the Benjamini and Hochberg's false discovery method were applied to obtain the target pathways in IPA analysis.

### 2.6. Results of miRNA Profiling

**Figure 1:**
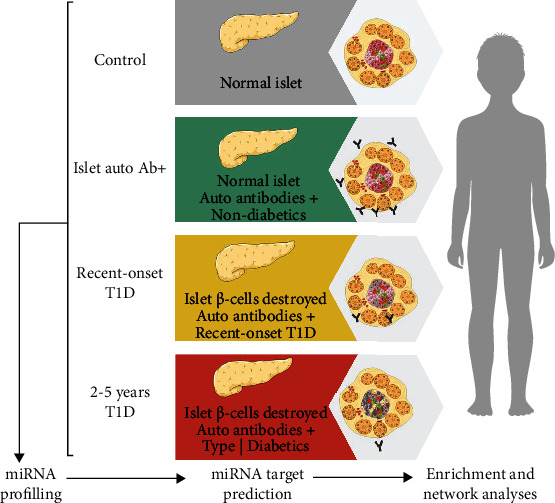
Progression of type 1 diabetes and experimental design.

**Figure 2:**
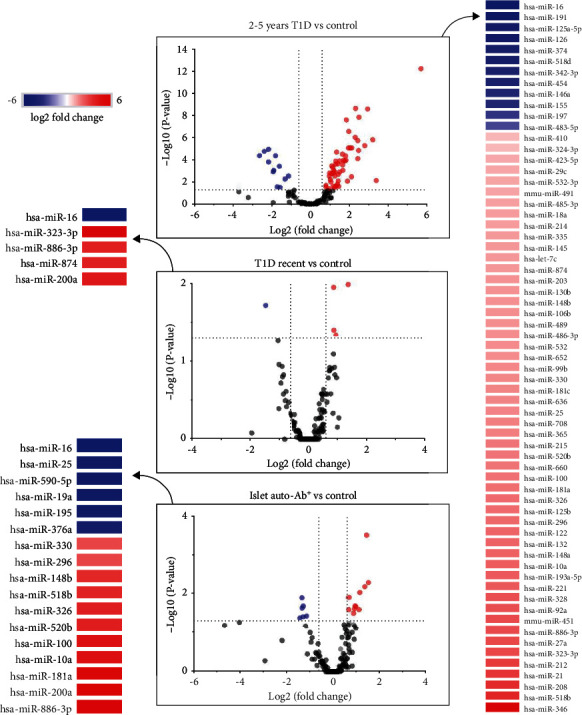
Volcano plots with changes in miRNAs levels between the AbP group (individuals with islet autoantibodies without diabetes; *n* = 25), recent T1D group (newly diagnosed T1D patients with duration ≤6 months; *n* = 30), and T1D 2-5y group (patients with T1D with 2 to 5 years of duration; *n* = 26) in comparison with health controls (*n* = 29).The log fold change is shown on the *X*-axis and log 2 of the *p* value on the *Y*-axis.

We performed miRNA profiling from 110 subjects divided into four groups (workflow in Figure 1). The number of miRNAs detected in each group sample was similar. The analysis of 135 miRNAs expressed in 20% or more of each group evidenced 69 DEMs across the recent T1D, AbP, and T1D 2-5y groups in comparison to control group.  Figure 2 shows a volcano plot representation of the DEMs comparing each group versus control, represented as blue dots for downregulation and red dots for upregulation. The T1D 2-5y group had the highest number of DEMs with 51 upregulated and 13 downregulated, followed by the AbP group (10 upregulated and 6 downregulated) and the recent T1D group (3 upregulated and 1 downregulated DEMs) (Table 2).

We observed two different profiles. The 18 miRNAs consistently deregulated in AbP or recent T1D groups (13 of them also deregulated in T1D 2-5y) (Table 2) comprising the cluster A (12 up- and 6 downregulated) were analyzed separately from the miRNAs deregulated only in the T1D 2-5y group (cluster B: 40 up- and 11 downregulated miRNAs) (Table 3 and Figure 2).

We observed that T1D progression leads to a predominance of miRNAs upregulation in serum when compared to controls. The most upregulated DEMs were miR-200a-3p, in both the AbP and recent T1D groups and miR-346 in T1D 2-5y group, respectively. The downregulated miR-16 was the only miRNA that differed from controls simultaneously in the three groups.

**Figure 3:**
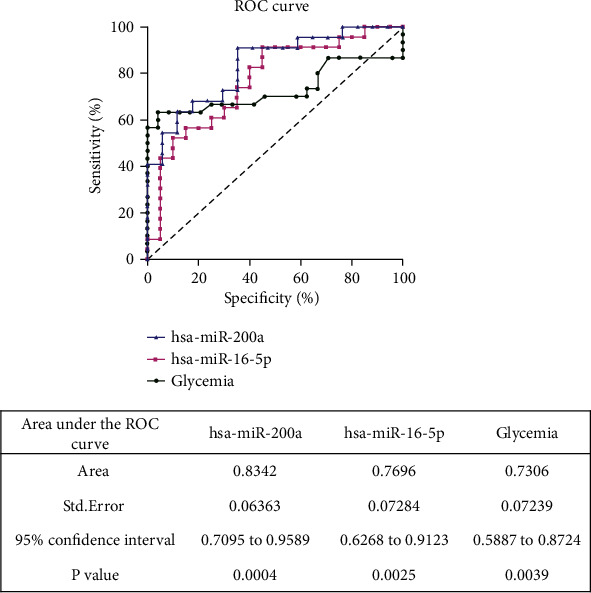
ROC curves of miR-200a-3p, miR-16-5p, and glucose levels.

Receiver operating characteristic (ROC) curve analysis revealed that miR-16-5p and miR-200a-3p can be used as T1D predictors (AUC = 0.7696, *p* < 0.0025 and AUC = 0.8342, *p* < 0.0004, respectively) presenting higher discriminating power, even when we compared to the glucose ROC curve (AUC = 0.7306, *p* < 0.0039)(Figure 3).

Five miRNAs (miR-195-5p, miR-19a-3p, miR376a-3p, miR-590-5p, and miR-25-3p) were downregulated only in the AbP group, whereas miR-323a-3p and miR-874-3p were upregulated both in the recent T1D and T1D 2-5y groups. Most of the other miRNAs from cluster A were increased only in the AbP and T1D 2-5y groups.

Fifty-nine (15.6%) of the 377 miRNAs evaluated were not expressed in any group (Suppl. 1). Only one miRNA (reclassified as Vault RNA; ncRNA-886) was upregulated in all three groups.

### 2.7. Correlations

IA2A, GADA, and Znt8A levels correlated positively with 13, 12, and 1 miRNAs, respectively. Negative correlations were found for IA2A levels with miR-19a-3p and for ZnT8A levels with miR-100-5p and miR-16-5p (Tables 4–6). Correlations with autoantibodies were more frequent in cluster A (10 of 18 miRNAs = 55.6%) than in cluster B, presenting longer diabetes duration (9 of 51 miRNAs = 17.6%; *p* = 0.0044; OR = 5.83; CI:1.799-18.910). The influence of glucose levels was suggested by both positive and negative correlations of glucose and/or HbA1c levels with several up and downregulated miRNAs. The correlations of miRNA with C-peptide levels and with age were usually negative in both clusters.

### 2.8. Target Prediction and Pathway Analysis

In both clusters, most pathways potentially regulated by DEMs were related to cancer, cell growth and metabolism (ErbB, MAPK, Wnt signaling pathway, and endocytosis), insulin production, and axon guidance/neurotrophin signaling pathways. Adherens junction and apoptosis pathways were enriched by DEMs' targets from the T1D 2-5y group.

The deregulated miRNAs were referred to several biological pathways by the miRWalk database, retrieving 206 and 140 pathways modulated by up- and downregulated miRNAs in cluster A (Suppl. 2) and 64 and 46 pathways in cluster B, respectively (Suppl. 3-4). In both clusters, most pathways potentially controlled by DEMs were related to cancer, growth, metabolism (ErbB, MAPK, Wnt signaling pathway, and endocytosis), insulin, and axon guidance/neurotrophin signaling pathways. Adherens junction and apoptosis pathways were enriched by up- and downregulated DEMs' targets mainly from the T1D 2-5y group. The more frequent DEM gene targets identified by Target Scan tool are presented in Suppl. 5-6.

**Figure 4:**
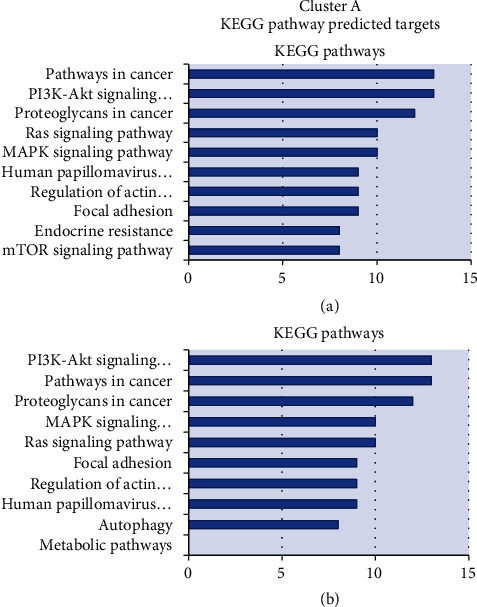
Distribution of enriched KEGG pathways of cluster A: 18 miRNAs consistently deregulated in AbP or recent T1D groups (13 of them being also deregulated in T1D 2-5y). The chart shows category rankings. The *X*-axis value represents the number of microRNA's targets in the pathway and the *Y*-axis the pathways. (a) Represents the potential genes from up-expressed microRNAs. (b) Represents the potential genes from down-expressed microRNAs in comparison with controls. AbP group (individuals without diabetes expressing islet autoantibody); recent T1D group (newly diagnosed T1D patients with duration ≤6 months); T1D 2-5y group (patients with T1D with 2 to 5 years of duration); IAA: insulin autoantibody; GADA: glutamic acid decarboxylase autoantibody; IA-2A: tyrosine phosphatase autoantibody; ZnT8A: zinc transport 8 autoantibody; only target genes identified by 20% or more of the DEMs with seed of conserved 7-8 mers were submitted to functional enrichment analysis.

**Figure 5:**
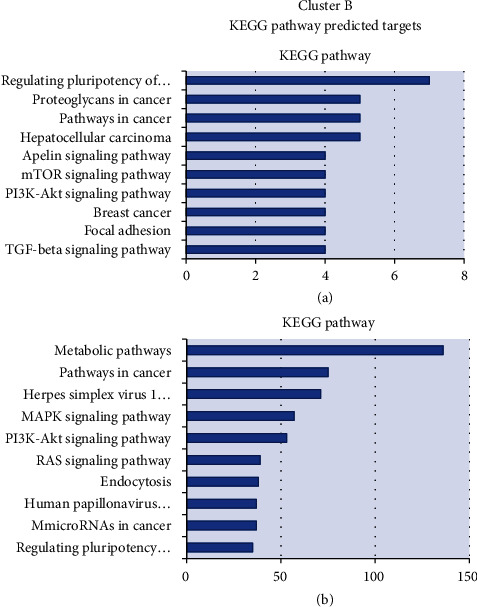
Distribution of enriched KEGG pathways of cluster B: 51 miRNAs deregulated only in the T1D 2-5y group. The chart shows category rankings. The *X*-axis value represents the number of microRNA's targets in the pathway and the *Y*-axis the pathways. (a) Represents the potential genes from up-expressed microRNAs. (b) Represents the potential genes from down-expressed microRNAs in comparison with controls. AbP group (individuals without diabetes expressing islet autoantibody), recent T1D group (newly diagnosed T1D patients with duration ≤6 months), and T1D 2-5y group (patients with T1D with 2 to 5 years of duration); health control group; Rq: relative expression represented by fold change in comparison to controls; *p* value: corrected *p* value. Only target genes identified by 20% or more of the DEMs with seed of conserved 7-8 mers were submitted to functional enrichment analysis.

Pathway enrichment analyses were performed using KEGG and IPA software. Figures 4 and 5 list the enriched pathways of the DEMs' targets from both cluster profiles using KEGG tool. Proliferative, metabolic, and immune responses were the top pathways identified for up- and downregulated miRNAs. Pathways related to survival (autophagy) and self-renewal capacity (regulating pluripotency of stem cells) were associated to downregulated miRNAs. More pathways were potentially modulated by downregulated DEMs in clusters A and B (216 and 321) than by upregulated DEMs (118 and 153 pathways), respectively.

Targeting and pathway analysis using IPA software identified highly predicted and/or experimentally observed targeting information from 10 DEMs (out of 16) in the AbP group with 359 targets: 1 DEM (out of 4) in the recent T1D group with 198 targets and 45 DEMs (out of 64) in the T1D 2-5y group with 1033 targets.

**Figure 6:**
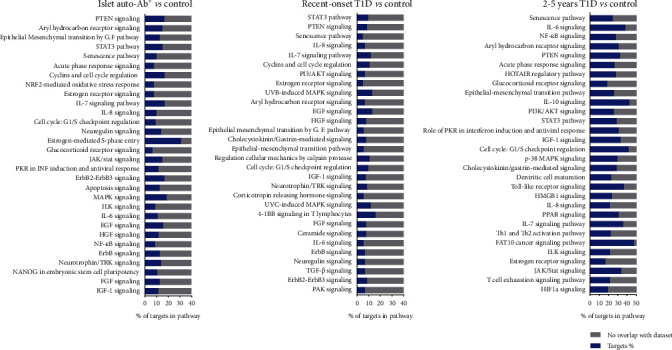
Ingenuity Pathway Analysis (IPA). Canonical pathways most significantly enriched by serum miRNAs from the AbP group (individuals with islet autoantibodies without diabetes; *n* = 25), recent-onset type 1 diabetes (T1D) group (newly diagnosed T1D patients with duration ≤6 months; *n* = 30), and T1D 2-5y group (patients with T1D with 2 to 5 years of duration; *n* = 26) in comparison with health controls. The stacked bar chart displays the percentage of target DEGs molecules present in each pathway. The Benjamini-Hochberg method was used to adjust the right-tailed Fischer's exact test *p* value, which was always <0.001.

After excluding cancer-related pathways, we identified a total of 366, 320, and 425 enriched canonical pathways related to DEMs' targets from the AbP, recent T1D, and T1D 2-5y groups, respectively (Suppl. 7). The top forty canonical pathways most enriched with DEMs' targets identified by IPA analysis for each one of the three groups compared to control were the PTEN (phosphatase and tensin homolog deleted from chromosome ten), aryl hydrocarbon receptor, STAT3 (signal transducer and activator of transcription 3), epithelial-mesenchymal transition, and senescence pathways (Figure 6). Pathways related to cell proliferation and immune response, e.g., cyclins/cell cycle regulation, and Interleukins IL-7 and IL-8, were overrepresented in the AbP and recent T1D groups. NF-KB (nuclear factor kappa B), IL-6, IL-10, acute phase response, and glucocorticoid receptor signaling pathways were overrepresented in the T1D 2-5y group. miRNAs from all groups were associated with a great number of pathways related to growth (IGF-1, FGF, HGF, EGF, ErbB, JAK/STAT, and PI3K/AKT signaling) and cell division (cyclins/cell cycle regulation). Inflammatory and defense pathways (ILK, neuregulin, dendritic cell maturation, toll-like receptor signaling, and Th1/Th2 activation pathways) were modulated. Apoptosis, virus response, and neuronal development/repair (neuroinflammation, PEDF, neuregulin, and axonal guidance signaling pathways) were also affected. In general, the DEMs from the T1D 2-5y group modulated most genes from all pathways, with a greater magnitude than the other groups, many of them expressing an innate and adaptive immune responses.

**Figure 7:**
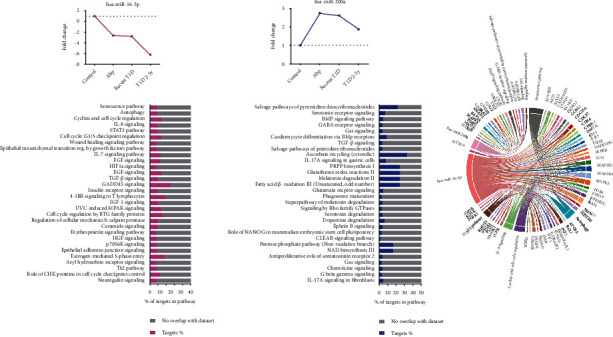
miR-16-5p and miR-200a-3p: serum levels expressed during T1D progression and canonical pathways most significantly enriched by serum miRNAs through Ingenuity Pathway Analysis (IPA). AbP group (individuals with islet autoantibodies without diabetes; *n* = 25), recent-onset type 1 diabetes (T1D) group (newly diagnosed T1D patients with duration ≤6 months; *n* = 30), and T1D 2-5y group (patients with T1D with 2 to 5 years of duration; *n* = 26) in comparison with health controls. The stacked bar chart displays the percentage of target DEGs molecules present in each pathway. The Benjamini-Hochberg method was used to adjust the right-tailed Fischer's exact test *p* value, which was always <0.001.

The miRNAs miR-16-3p and miR-200a-3p had a high number of identified targets. The miR-16-3p potentially regulates pathways related to GADD45 (growth arrest and DNA damage-inducible 45) signaling, cell cycle regulation by antiproliferative BTG family proteins, cell cycle checkpoint control, EGF signaling, senescence, and autophagy pathways. miR-200a-3p has the most targets related to metabolic pathways, e.g., ascorbate recycling, PRPP (phosphoribosyl pyrophosphate) biosynthesis I, glutathione redox reaction II, melatonin degradation II, fatty acid *β*-oxidation III, salvage pathways of pyrimidine deoxyribonucleotides, pentose phosphate, and NAD biosynthesis III pathways (Figure 7).

## 3. Discussion

We identified miRNA dysregulation during T1D evolution. By comparing patients at different stages of disease progression with healthy individuals, from the initial immunological manifestation (one to three autoantibodies in AbP group) until partial and strong beta cell damage in the recent T1D and T1D 2-5y groups, we have found 69 differentially expressed miRNAs. These miRNAs have predicted targets related to immune regulation, metabolism, glucose homeostasis, cell proliferative/survival mechanisms, and beta cell function. Dysregulated expression of miRNAs pointed to possible mechanisms underlying the pathophysiology of T1D. The AbP group could also provide the effects of miRNA dysregulation and pathways predicted to be activated during the onset of islet autoimmunity that are unrelated to blood glucose levels. The most enriched pathways potentially regulated by DEMs were related to immune cell activation, inflammation, and apoptosis.

The miR-200a-3p was the most highly expressed miRNA in AbP and recent T1D groups positively correlated to IA2A and GADA levels, suggesting a robust association with T1D pathogenesis. miR-200a was previously reported to be highly expressed in beta cells [11], being associated with their damage and apoptosis in vitro [12]. We hypothesize that its decrease to values similar to controls in the T1D 2-5y group could be linked to the scarcity of beta cells in this group or to hyperglycemia, although its expression was not correlated with glucose or HbA1c levels. Mechanistically, it was described as an antiapoptotic and stress-resistance miRNA, with targets that include the beta cell chaperone Dnajc3 and the caspase inhibitor XIAP and positively controls the activation of the tumor suppressor Trp53 [13].

Other important target of this miRNA is the thioredoxin-interacting protein (TXNIP). Its proapoptotic and diabetogenic function prevents beta cell function via induction of miR-200a in vitro. The TXNIP/miR-200/Zeb1/E-cadherin signaling pathway links miR-200 to beta cell apoptosis and diabetes and links TXNIP to inhibition of epithelial-mesenchymal transition (EMT), a process involved in beta cell expansion [12], predicting its decline [14]. Catabolic and oxidizing reactions related to NAD generation, antioxidant mechanisms, and synthesis of nucleotides, suggested by canonical pathways associated with miR-200a-3p, are probably related to cell lesion and repair (Figure 7).

Besides miR-200a-3p, other upregulated miRNAs (miR-181a and miR-323) were also described as inhibitors of EMT [14], which could worsen beta cell function precociously, in the phase of ongoing autoimmunity (AbP) or in recent T1D group. In accordance, the EMT pathway was an enriched pathway in all three groups (Figure 6).

Among the upregulated DEMs, we also observed miR-296, miR-874 (miR-Walk pathway), and miR-518, all previously associated with apoptosis signaling [15]. Others were negatively involved in beta cell formation/differentiation and survival, insulin processing/secretion, and glucose homeostasis like miR-518b, miR-330-3p, miR-148b-3p, miR-181a-5p, and miR-330-3p [14, 16–18]. Deleterious effects were still observed for upregulated miRNAs miR-520b, miR-326, and miR-181a-5p related to response to cell stress [19], to islet autoantibodies and Th17 pathway [20], or impairing regulatory T cells (Tregs) induction [21], respectively, probably further hampering the recovery of beta cells.

Tregs are fundamental in individual protection from autoimmunity and miRNAs reported as inhibitors of their differentiation, development, and immunological functions were upregulated in both clusters A (miR- 181a, miR-200a, miR-330-3p, and miR-326) [21, 22] and B (miR-27a, miR-92a, miR-193, and miR-181c) [22–24]. In addition, other miRNAs related to Treg cell-mediated immunological tolerance were downregulated in AbP (miR-195 and miR-16) [23–25] and in T1D-2-5y groups (miR-18a, miR-27, miR-155, miR-126, and miR-146a) [22, 25–27], limiting control of beta cell offensive attack. The deleterious role of upregulated miRNAs in autoimmune aggression was further suggested by their negative correlation with C-peptide levels and positive correlation with islet autoantibodies (Table 4). The correlations of miRNAs with autoantibody titers were more frequent in cluster A (55.5% of the miRNAs), which is expected greater immunological activation than in cluster B (17.6% of miRNAs) (*p* = 0.004), probably subjected to effects of longer diabetes duration, decreasing antibody titers, and to other stimuli such as metabolic disturbances. Exceptions to all trends in immune activation were observed for miR-100-5p, negatively correlated to ZnT8A (*r* = −0.358; *p* = 0.044)(Table 4), and opposed to inflammation [23] and for a few other upregulated miRNAs such as miR-10a-5p and miR-874, acting through stabilizing Tregs [25, 28], inhibiting NF-*κ*B, TNFalpha, IL-6, and IL-1*β* signaling [29].

Among cluster A downregulated miRNAs, miR-16-5p was the unique miRNA downregulated in all three groups in comparison to controls. It seems to favor Treg induction [22, 25], which is highly expressed in beta cells, and negatively regulates the protein Ptch1 (protein patched homolog 1) involved in the inhibition of beta cell proliferation [30]. miR-16 seems to protect from high glucose-induced pancreatic beta cell apoptosis by targeting CXCL10 [31] and from immune aggression as it was negatively correlated with ZnT8A levels in our cohort (*r* = −0.358; *p* = 0.044). The most enriched pathways for miR-16 predicted targets were related to cell cycle regulation and division, checkpoint control, and DNA repair (Figure 7).

The insulin resistance due to autoimmune aggression and the release of inflammatory cytokines might function decreasing the levels and the protective role of miR-16 [32]. miR-16 was downregulated already in the preclinical phase of diabetes, whereas hyperglycemia, which was correlated negatively with miR-16 levels (*r* = −0.289, *p* = 0.003), probably influenced its great decline in the T1D 2-5y group. Other five miRNAs downregulated only in the AbP group are involved in immune regulation (miR-195-5p and miR-590-5p) [33], cell proliferation, insulin transcription (miR-376a-3p and miR-19a-3p) [34, 35], and residual beta cell function: miR-25 [7]. Their downregulation could potentially prevent beta cell recovery and inflammation resolution and is mirroring the autoimmune aggression in progress, unrelated to glucose levels.

Therefore, parts of upregulated genes, including those in the AbP group, reveal a profile toward inflammation, apoptosis, and commitment of beta cell and insulin secretion, evidencing cluster A DEMs as relevant to autoantibody development, decreased C-peptide levels, T1D pathogenesis, and progression. Few miRNAs were associated with immunomodulation and anti-inflammatory effects.

The expression of most miRNAs of the cluster A followed a similar pattern in the three groups, although not always with statistical significance. The worsening of beta cell function, dysregulation of glucose levels, and insulin resistance probably accounted for the progressive upregulated trend of the expression of these miRNAs toward the T1D 2-5y group, particularly for miR-518b and miR-323a-3p, interfering negatively with beta cell expansion [14] and insulin secretion [36]. The exceptions were miR-200a-3p and miR-25-3p, associated with islet autoantibodies, in which the response of the T1D 2-5y group was opposite of the AbP and recent T1D groups, supporting its role in immunological aggression.

Thus, the altered expression of miRNAs from cluster A may be implicated in the cycle of damage regeneration of beta cells, inflammation, and metabolic disorders, contemplating insulin resistance and later, hyperglycemia. Cluster B (composed of DEMs expressed only in the T1D 2-5y group; *n* = 51) conferred similar results. Most upregulated miRNAs were present in human pancreatic beta cells [11].

Published studies link several DEMs from both clusters to T1D evolvement, metabolic disturbs, impairment of beta cell function [1, 2, 5–7, 12–14, 18, 20, 22, 30–32, 34–36], or taking part in other autoimmune diseases like systemic lupus erythematosus, rheumatoid arthritis, Crohn's disease, multiple sclerosis, and autoimmune thyroid disease [2, 27, 37, 38].

Nc886 (pre-miR-886), upregulated in the three groups, seems to activate the protein kinase RNA-activated; an interferon-inducible kinase maybe related to defense against viruses [39].

The analysis using miRWalk and KEGG databases reinforced the relevance of both clusters in the autoimmune process. The potential biological pathways enriched by DEMs targeted oncogenic/proliferative and metabolic pathways, processes related to cell differentiation, migration, survival, apoptosis, neural development, insulin signaling, and immune system pathways. Most of these pathways are essential for proper lymphocyte development and function, and their enrichment, which was greater in cluster A, may be a reflection of the autoimmune activity.

Interestingly, these pathways were enriched by up- and downregulated miRNAs from both clusters, although through different targets.

Pathway enrichment analyses were performed using KEGG and IPA software. Figures 4 and 5 list the enriched pathways of the DEMs' targets from both cluster profiles using KEGG tool. Proliferative, metabolic, and immune responses were the top pathways identified for up- and downregulated miRNAs. *Pathways related to survival (autophagy in cluster A) and self-renewal capacity (regulating pluripotency of stem cells in cluster B) were less modulated by downregulated miRNAs, perhaps in an attempt to contain the self-aggression.*

Genes related to B and T cell differentiation/function were targeted by upregulated miRNAs in cluster A (*HIPK1*) and B (*NAA50, NFAT5, OTUB7B*) and by downregulated miRNAs in clusters A (*BRWD1*) and B (*ABL2*, *FOSL2*, and *PDE7A*).

In a similar way, genes related to beta cell function were associated with upregulated miRNAs in clusters A (FZD5 and GATA6) and B (KCNJ6) and downregulated miRNAs in clusters A (PLAG1) and B (FBXO28). Importantly, one of the miRNAs' gene targets from cluster A was DYRK2 (dual specificity tyrosine-phosphorylation-regulated kinase 2), which is an autoantibody target antigen in T1D [40]. The DEMs targeting DYRK2 were miR-181a-5p, miR-326, and miR-874-3p (positively correlated to islet autoantibodies) and miR-148b-3p, all previously related to T1D and/or autoimmune diseases [1, 2, 17, 21, 22].

One of the most significantly enriched canonical pathways in the three groups (Figure 6), pathogenetically relevant in T1D, was the senescence pathway, a state of cellular arrest associated with inflammatory cytokines, growth factors, and matrix metalloproteinases triggered by several damaging factors present in the three groups (like immune attack and metabolic derangements). In the same direction, the STAT3 pathway, regulating Th17 cell differentiation and suppressing Treg generation [41], and the PTEN pathway, involved in T helper follicular cell precursor induction, autoantibody generation and triggering of islet autoimmunity [42, 43], and the regulation of EMT [14], all have their role in inflammation and reduction of beta cell function/survival. The IL-7 and IL-8 inflammatory signaling pathways were highly represented in the AbP and recent T1D groups whereas IL-6, NF-KB, and acute phase response pathways in the T1D 2-5y group.

On the opposite direction, there was the aryl hydrocarbon receptor pathway, enriched in all three groups. It modulates the development and functionality of immune cells and suppresses the expression of inflammatory cytokines during diabetes development [44]. DEMs from the T1D 2-5y group also target anti-inflammatory pathways like IL-10 A and glucocorticoid receptor signaling.

In general, DEMs from all groups target mechanisms related to growth, cell cycle regulation, apoptosis, inflammation, defense, and neuronal pathways, whereas few of them favored defense against an autoimmune offensive. The commitment of these pathways increased progressively from AbP to the recent T1D and then to T1D 2-5y group, which regulated the largest number of targets in most pathways, many of them expressing innate and adaptive immune responses. The metabolic dysregulation probably influenced these results.

Of note, similar pathways were enriched by both up- and downregulated miRNAs, triggered probably by different causal hits. Other possibility is the intense metabolic derangement due to glucose/lipotoxicity and inflammatory cytokines, eliciting miRNAs from inflammatory pathways. This can also explain some inconsistent results in the literature, where the same miRNA predisposed or protected from T1D or other autoimmune diseases. Diabetes duration, age of patients, and glucose levels could have acted in miRNA deregulation during disease progression and taken part in these inconsistencies, pointing to the importance of these variables in disease determinations. This can be further evidenced when considering the 69 miRNAs from clusters A to B, where 26 DEMs correlated with glycemic status, 19 with autoantibodies levels and ongoing islet autoimmunity, and 20 miRNAs correlated negatively with age. T1D duration influenced positively and negatively the levels of 19 miRNAs and their association with autoantibody titers, which are known to decrease with time. However, these results should be considered carefully, considering the sample size and low serum RNA yield.

Although the serum miRNA profile might not mirror the situation in the affected pancreas, it is notable that most of the DEMs of our cohort are miRNAs enriched in beta cells, suggesting they may correlate with the severity of beta cell injury. Ongoing destruction of beta cells could result in diffusion of islet-enriched miRNAs into serum, as observed during the autoimmune attack in animal models of diabetes [45]. Furthermore, the negative correlation of several upregulated miRNAs expression with C-peptide levels as well as with age, considering that the immune attack is usually more intense in the youngest, reinforces our results. Even more, previous reports of the participation of most miRNAs in the pathogenesis of T1D and other autoimmune manifestations also point to their role in the autoimmune process. Their expression in the different phases of the autoimmune lesion, starting with positivity to islet autoantibodies up to severe beta cell dysfunction, evidencing the influence of age, duration of diabetes, and glycemic control on their expression brings relevant information and suggests new immunological and metabolic influences.

In conclusion, our data suggested the potential role of miRNAs favoring the preponderance of pathways compromising beta cell function throughout diabetes progression like increased apoptosis, inhibition of EMT, impaired TREG function, inflammatory pathways like STAT3 and PTEN, and senescence probably being indirect signs of islet autoimmunity and metabolic derangements due to gluco-lipid toxicity. The higher discriminating power for T1D prediction for miR-200a-3p and miR-16-5p, distinguishing patients from the different stages of T1D, suggested both miRNAs as potential biomarkers and targets for beta cell recovery.

## Figures and Tables

**Table 1:** Expression of serum miRNAs of AbP, recent T1D, and T1D 2-5y groups in relation to the control group.

	Control group	AbP group	Recent T1D group	T1D 2-5y group	*p* value
Number	29	25	30	26	
Age (years)	14.5 (10.0-18.9)	11.7 (9.0-19.0)	13.0 (9.9-18.6)	13.3 (10.7-18.7)	0.5226
Ethnicity (white) %	68.9	62.5	86.2	76.9	0.2213
Female %	58.6	44	50	42.3	0.6150
Glucose levels (mg/dL)	81 (76.5-88.0)	86 (78.2-90.7) £	113.5 (82-193)#	189 (121-287.5) §	<0.0001
Age at diagnosis (years)			12.8 (9.0-18.2)	10.5 (6.7-15.2)	
Diabetes duration (years)			0.25 (0.09-0.33)	3.8 (2.9-4.6)	
HbA1c (mmol/mol)	33.3 (30.1-35.5)	32.2 (27.9-35.5) £¥	56.3 (49.7-76.0) #	66.1 (60.7-72.7) §	<0.0001
HbA1c (%)	5.2 (4.9-5.4)	5.1 (4.7-5.4) £¥	7.3 (6.7-9.1) #	8.2 (7.7-8.8) §	<0.0001
IAA (nU/mL)	0.0 (0.0-19.7)	69.5 (24.7-75.3)∗	—	—	<0.0001
GADA (Ul/mL)	0.0 (0.0-2.0)	64 (22.2-335)∗	161.5 (48-477)#	46.0 (11.3-642.5) §	<0.0001
Anti-ZnT8 (Ul/mL)	1.0 (0.0-6.0)	5.5 (3.0-412.6) ¥	645 (222-1.127) #	48 (5.50-520.0) §	<0.0001
IA2-A (Ul/mL)	0.0 (0.0-12.5)	12.0 (0.0-152.5) ¥£	812.0 (267.3-2.365) #	206 (34-638) §	<0.0001
C-peptide (ng/mL)	2.2 (1.8-3.0)	2.2 (1.3-3.0) £¥∗	0.65 (0.4-1.1) #	0.2 (0.1-0.4) §	<0.0001
HLA-DR3 or DR4 alleles (%)	10.3	57.1∗£¥	92.9 #	96 §	<0.0001
HLA-DQ2 or DQ8 alleles (%)	13.8	75∗	89.3 #	92 §	<0.0001

AbP group (individuals without diabetes expressing islet autoantibody), recent T1D group (newly diagnosed patients with type 1 diabetes with duration ≤6 months), T1D 2-5y group (patients with type 1 diabetes with 2 to 5 years of duration), and health control group, IAA: insulin autoantibody; GADA: glutamic acid decarboxylase antibody; IA-2A: tyrosine phosphatase autoantibody; ZnT8A: zinc transport 8 autoantibody; # (recent T1D × Control); § (T1D 2-5y × control); ∗(AbP × control); £ (AbP × T1D 2-5); ¥ (AbP × recent T1D).

**Table 2:** MicroRNA expression profile of cluster A in comparison to the control group.

ID MIRBASE	AbP group	*p* value	Recent T1D group	*p* value	T1D 2-5y group	*p* value
	Rq		Rq		Rq	
hsa-miR-100-5p	2.188	0.026	1.45	0.431	3.294	0,00E+00
hsa-miR-10a-5p	2.237	0.009	1.83	0.081	4.161	0,00E+00
hsa-miR-148b-3p	1.857	0.032	1.447	0.231	2.419	0.003
hsa-miR-181a-5p	2.588	0.007	1.4	0.725	3.415	0,00E+00
hsa-miR-200a-3p	2.728	0,00E+00	2.598	0.01	1.858	0.069
hsa-miR-296-5p	1.624	0.012	1.379	0.263	3.639	0,00E+00
hsa-miR-326	1.944	0.021	1.669	0.121	3.52	0,00E+00
hsa-miR-330-3p	1.614	0.026	1.39	0.263	2.675	0,00E+00
hsa-miR-518b	1.907	0.025	1.656	0.133	10.541	0.007
hsa-miR-520b-3p	1.986	0.021	1.625	0.164	3.179	0,00E+00
hsa-miR-323a-3p	1.455	0.193	1.847	0.039	5.677	0,00E+00
hsa-miR-874-3p	1.426	0.071	1.837	0.011	2.294	0.001
hsa-miR-16-5p	0.379	0.042	0.358	0.019	0.16	0,00E+00
hsa-miR-195-5p	0.421	0.021	0.527	0.116	0.548	0.089
hsa-miR-19a-3p	0.418	0.039	0.648	0.461	0.527	0.133
hsa-miR-376a-3p	0.463	0.037	0.821	0.906	0.829	0.902
hsa-miR-590-5p	0.406	0.023	0.754	0.775	0.749	0.669
hsa-miR-25-3p	0.405	0.013	0.717	0.671	2.763	0.024

AbP group (individuals without diabetes expressing islet autoantibody), recent T1D group (newly diagnosed patients with type 1 diabetes with duration ≤6 months), T1D 2-5y group (patients with type 1 diabetes with 2 to 5 years of duration), and health control group, Rq: relative expression represented by fold change in comparison to controls, *p* value: corrected *p* value.

**Table 3:** MicroRNA expression profile of cluster B in comparison to the control group.

ID MIRBASE	AbP group	*p* value	Recent T1D group	*p* value	T1D 2-5y group	*p* value
	Rq		Rq		Rq	
hsa-let-7c-5p	1.592	0.065	1.334	0.53	2.284	0.002
hsa-miR-18a-5p	1.404	0.063	1.17	0.671	2.171	0,00E+00
hsa-miR-346	1.922	0.069	1.754	0.125	51.456	0,00E+00
hsa-miR-708-5p	1.864	0.063	1.512	0.264	2.875	0.001
hsa-miR-491-5p	1.638	0.059	1.41	0.275	2.052	0.006
hsa-miR-106b-5p	1.434	0.53	1.064	1,00E+00	2.479	0.023
hsa-miR-122-5p	1.771	0.711	2.074	0.532	3.795	0.008
hsa-miR-125b-5p	1.592	0.207	1.876	0.12	3.591	0,00E+00
hsa-miR-130b-3p	0.966	1,00E+00	1.189	0.854	2.401	0.001
hsa-miR-132-3p	1.157	0.937	1.371	0.532	3.918	0,00E+00
hsa-miR-145-5p	0.842	0.988	1.071	1,00E+00	2.281	0.023
hsa-miR-148a-3p	1.414	0.374	1.372	0.464	3.96	0,00E+00
hsa-miR-181c-5p	1.634	0.11	1.405	0.418	2.707	0.002
hsa-miR-193a-5p	0.938	1,00E+00	1.234	1,00E+00	4.369	0.003
hsa-miR-203a-3p	1.638	0.323	1.965	0.163	2.396	0.031
hsa-miR-208a-3p	1.977	0.078	1.72	0.263	9.314	0,00E+00
hsa-miR-21-5p	1.058	1,00E+00	1.405	0.716	7.703	0,00E+00
hsa-miR-212-3p	1.052	1,00E+00	1.137	1,00E+00	6.975	0,00E+00
hsa-miR-214-3p	1.488	0.107	1.064	1,00E+00	2.178	0.002
hsa-miR-215-5p	1.537	0.138	1.677	0.132	3.164	0,00E+00
hsa-miR-221-3p	1.019	1,00E+00	0.982	1,00E+00	4.421	0,00E+00
hsa-miR-27a-3p	1.188	0.948	1.303	0.732	5.625	0,00E+00
hsa-miR-29c-3p	1.305	0.134	1.208	0.546	1.991	0.003
hsa-miR-324-3p	1.063	1,00E+00	1.294	0.539	1.764	0.019
hsa-miR-328-3p	0.837	0.906	0.836	0.977	4.999	0,00E+00
hsa-miR-335-5p	0.803	0.716	0.81	0.804	2.243	0,00E+00
hsa-miR-365a-3p	1.773	0.099	1.894	0.15	2.876	0,00E+00
hsa-miR-410-3p	1.284	0.447	1.186	0.766	1.749	0.026
hsa-miR-423-5p	1.352	0.532	1.04	1,00E+00	1.953	0.037
hsa-miR-485-3p	0.813	0.503	0.997	1,00E+00	2.106	0.009
hsa-miR-486-3p	1.441	0.081	1.16	0.804	2.5	0,00E+00
hsa-miR-489-3p	1.513	0.092	1.404	0.31	2.488	0,00E+00
hsa-miR-532-5p	1.003	1,00E+00	0.861	1,00E+00	2.541	0.021
hsa-miR-532-3p	1.362	0.345	1.408	0.264	2.011	0.002
hsa-miR-636	1.532	0.457	1.482	0.45	2.721	0.008
hsa-miR-652-3p	1.254	0.264	1.301	0.351	2.566	0,00E+00
hsa-miR-660-5p	1.002	1,00E+00	1.053	1,00E+00	3.203	0.001
hsa-miR-92a-3p	1.002	1,00E+00	1.124	1,00E+00	5.08	0,00E+00
hsa-miR-99b-5p	0.875	1,00E+00	1.274	0.804	2.646	0.015
hsa-miR-451a	1.319	0.78	1.2	0.977	5.451	0,00E+00
hsa-miR-125a-5p	0.954	1,00E+00	0.57	0.321	0.22	0,00E+00
hsa-miR-126-3p	0.938	1,00E+00	0.479	0.055	0.221	0,00E+00
hsa-miR-146a-5p	0.767	0.641	0.547	0.15	0.324	0,00E+00
hsa-miR-155-5p	0.536	0.481	0.986	1,00E+00	0.332	0.029
hsa-miR-191-5p	0.788	0.728	0.49	0.11	0.19	0,00E+00
hsa-miR-197-3p	0.676	0.4	0.734	0.61	0.394	0.005
hsa-miR-342-3p	1.209	0.717	0.815	0.82	0.282	0,00E+00
hsa-miR-374a-5p	0.614	0.345	0.654	0.484	0.259	0.001
hsa-miR-454-3p	0.507	0.1	0.752	0.796	0.299	0.026
hsa-miR-483-5p	1.577	0.134	1.324	0.418	0.448	0.003
hsa-miR-518d-3p	1.194	0.884	1.148	1,00E+00	0.264	0.001
Non-coding RNA-886						
hsa-miR-886-3p	2.883	0.005	1.938	0.045	5.455	0,00E+00

AbP group (individuals without diabetes expressing islet autoantibody); recent T1D group (newly diagnosed patients with type 1 diabetes with duration ≤6 months); T1D 2-5y group (patients with type 1 diabetes with 2 to 5 years of duration). Rq: relative expression represented by fold change in comparison to controls. *p* value: corrected *p* value.

**Table 4:** Correlations of miRNAs from cluster A.

miRNAs	IA2A		GADA		ZnT8A		Glucose		HbA1c		Age		C-peptide	
	*p*	*r*	*p*	*r*	*p*	*r*	*p*	*r*	*p*	*r*	*p*	*r*	*p*	*r*
*Upregulated*														
miR-181a-5p	0.033	0.255	0.005	0.341							0.023	-0.277	0.038	-0.342
miR-200a-3p	0.027	0.255	0.026	0.262									0.035	-0.33
miR-296-5p	0.038	0.338					0.016	0.392					0.005	-0.577
miR-326	0.034	0.402					0.001	0.572	0.009	0.49			0.03	-0.542
miR-874-3p					0.026	0.474								
miR-518b														
miR-323a-3p	0.022	0.255	0.008	0.298			0.002	0.34	0.032	0.246			0,000	-0.572
miR-100-5p					0.044	-0.358								
*Downregulated*														
miR-16-5p					0.007	-0.268			0.002	-0.304	0.005	-0.27		
miR-25-3p	0.003	0.236							0.044	0.261			0.003	-0.476
miR-195-5p											0.002	-0.309		
miR-19a-3p	0.037	-0.214												

IAA: insulin autoantibody; GADA: glutamic acid decarboxylase antibody; IA-2A: tyrosine phosphatase autoantibody; ZnT8A: zinc transport 8 autoantibody.

**Table 5:** Correlations of upregulated miRNAs from cluster B.

miRNAs	IA2A		GADA		Glucose		HbA1c		Age		C-peptide	
Upregulated	*p*	*r*	*p*	*r*	*p*	*r*	*p*	*r*	*p*	*r*	*p*	*r*
miR-106b-5p									0.049	-0.224		
miR-132-3p					0.037	0.287	0.015	0.337				
miR-145-5p	0.023	0.244	0.027	0.241							0.005	-0.407
miR-148a-3p	0.021	0.336	0.001	0.467							0.007	-0.49
miR-18a-5p												
miR-181c-5p			0.044	0.348								
miR-193a-5p					0.049	0.257					0.023	-0.406
miR-208a-3p	0.007	0.282	0.007	0.287	0.003	0.307	0,000	0.376			0.003	-0.441
miR-212-3p					0.05	0.263						
miR-214-3p									0.002	-0.495		
miR-215-5p	0.026	0.387	0.025	0.408								
miR-21-5p			0.049	0.208	0.002	0.314					0.03	-0.298
miR-346	0.035	0.274	0.049	0.261	0.001	0.425	0.001	0.447			0.004	-0.515
miR-221-3p					0.02	0.335			0.044	-0.293		
miR-130b-3p									0.039	-0.293		
miR-27a-3p												
miR-29c-3p												
miR-328-3p					0.003	0.297					0.003	-0.407
miR-410-3p									0.01	-0.404		
miR-423-5p									0.008	-0.267		
miR-451a					0.046	0.204			0.003	-0.303	0,000	-0.483
miR-485-3p												
miR-486-3p									0.006	-0.274		
miR-489-3p									0.016	-0.531		
miR-532-5p									0.016	-0.384	0.023	-0.481
miR-636	0.001	0.378	0.005	0.326							0.009	-0.398
miR-660-5p											0.002	-0.541
miR-886-3p												
miR-92a-3p					0.016	0.231					0.007	-0.351
miR-99b-5p	0.012	0.291	0.006	0.325	0.025	0.264					0.049	-0.313

IAA: insulin autoantibody; GADA: glutamic acid decarboxylase antibody; IA-2A: tyrosine phosphatase autoantibody; ZnT8A: zinc transport 8 autoantibody.

**Table 6:** Correlations of downregulated miRNAs from cluster B.

miRNAs	Glucose		HbA1c		Age	
*Downregulated*						
miR-125a-5p	0.014	-0.279	0.014	-0.284	0.03	-0.251
miR-126-3p	0.044	-0.196	0	-0.373	0.028	-0.217
miR-146a-5p			0.005	-0.277	0.018	-0.233
miR-155-5p					0.01	-0.3
miR-191-5p			0,000	-0.354		
miT-197-3p			0.033	-0.211	0.021	-0.228
miR-342-3p			0.001	-0.319	0.013	-0.245
miR-374a-5p			0.006	-0.291		
miR-483-5p			0.015	-0.242	0.035	-0.209
miR-518d-3p	0.021	-0.222	0.016	-0.235		

IAA: insulin autoantibody; GADA: glutamic acid decarboxylase antibody; IA-2A: tyrosine phosphatase autoantibody; ZnT8A: zinc transport 8 autoantibody.

## Data Availability

All data generated or analyzed during this study are included in this published article (and its supplementary information files, Supplementary Tables 1 to 7).

## Supplementary Materials

Supplementary Materials Supplementary Table 1S: miRNAs without expression in serum samples. Supplementary Table 2S: pathways related to up-and downregulated miRNAs of cluster A predicted by the miRWalk platform. Supplementary Table 3S: pathways related to upregulated miRNAs of cluster B predicted by the miRWalk platform. Supplementary Table 4S: pathways related to downregulated miRNAs of cluster B predicted by the miRWalk platform. Supplementary Table 5S: most frequent target genes of miRNAs from cluster A of TargetScan. Supplementary Table 6S: most frequent target genes of miRNAs from cluster B by TargetScan. Supplementary Table 7S: ingenuity canonical pathways related to differentially expressed miRNAs' targets. Supplementary Table 8S: reporting guidelines: STREGA.

## References

[1] Zheng Y., Wang Z., Zhou Z. (2017). miRNAs: novel regulators of autoimmunity-mediated pancreatic *β*-cell destruction in type 1 diabetes. *Cellular & Molecular Immunology*.

[2] Zhang L., Wu H., Zhao M., Lu Q. (2020). Identifying the differentially expressed microRNAs in autoimmunity: a systemic review and meta-analysis. *Autoimmunity*.

[3] Erener S., Marwaha A., Tan R., Panagiotopoulos C., Kieffer T. J. (2017). Profiling of circulating microRNAs in children with recent onset of type 1 diabetes. *JCI Insight*.

[4] Santos A. S., Cunha-Neto E., Fukui R. T., Ferreira L. R. P., Silva M. E. R. (2019). Increased expression of circulating microRNA 101-3p in type 1 diabetes patients: new insights into miRNA-regulated pathophysiological pathways for type 1 diabetes. *Frontiers in Immunology*.

[5] Assmann T. S., Recamonde-Mendoza M., de Souza B. M., Bauer A. C., Crispim D. (2018). MicroRNAs and diabetic kidney disease: systematic review and bioinformatic analysis. *Molecular and Cellular Endocrinology*.

[6] Takahashi P., Xavier D. J., Evangelista A. F. (2014). MicroRNA expression profiling and functional annotation analysis of their targets in patients with type 1 diabetes mellitus. *Gene*.

[7] Nielsen L. B., Wang C., Sørensen K. (2012). Circulating levels of microRNA from children with newly diagnosed type 1 diabetes and healthy controls: evidence that miR-25 associates to residual beta-cell function and glycaemic control during disease progression. *Experimental Diabetes Research*.

[8] Gomes K. F., Santos A. S., Semzezem C. (2017). The influence of population stratification on genetic markers associated with type 1 diabetes. *Scientific Reports*.

[9] American Diabetes Association (2021). Standards of medical care in diabetes. *Diabetes Care*.

[10] Schmittgen T. D., Livak K. J. (2008). Analyzing real-time PCR data by the comparative _C_ _T_ method. *Nature Protocols*.

[11] Klein D., Misawa R., Bravo-Egana V. (2013). MicroRNA expression in alpha and beta cells of human pancreatic islets. *PLoS One*.

[12] Filios S. R., Xu G., Chen J., Hong K., Jing G., Shalev A. (2014). MicroRNA-200 is induced by thioredoxin-interacting protein and regulates Zeb1 protein signaling and beta cell apoptosis. *The Journal of Biological Chemistry*.

[13] Belgardt B. F., Ahmed K., Spranger M. (2015). The microRNA-200 family regulates pancreatic beta cell survival in type 2 diabetes. *Nature Medicine*.

[14] Belongie K. J., Ferrannini E., Johnson K., Andrade-Gordon P., Hansen M. K., Petrie J. R. (2017). Identification of novel biomarkers to monitor *β*-cell function and enable early detection of type 2 diabetes risk. *PLoS One*.

[15] Feng C., Xian Q., Liu S. (2018). Micro RNA-518 inhibits gastric cancer cell growth by inducing apoptosis via targeting MDM2. *Biomedicine & Pharmacotherapy*.

[16] Sebastiani G., Guarino E., Grieco G. E., Formichi C., Delli P. C. (2017). Circulating microRNA (miRNA) expression profiling in plasma of patients with gestational diabetes mellitus reveals upregulation of miRNA miR-330-3p. *Frontiers in Endocrinology*.

[17] Azizi M., Teimoori-Toolabi L., Arzanani M. K., Azadmanesh K., Fard-Esfahani P., Zeinali S. (2014). MicroRNA-148b and microRNA-152 reactivate tumor suppressor genes through suppression of DNA methyltransferase-1 gene in pancreatic cancer cell lines. *Cancer Biology & Therapy*.

[18] Zhou B., Li C., Qi W. (2012). Downregulation of miR-181a upregulates sirtuin-1 (SIRT1) and improves hepatic insulin sensitivity. *Diabetologia*.

[19] Gaither K. A., Watson C. J. W., Madarampalli B., Lazarus P. (2020). Expression of activating transcription factor 5 (ATF5) is mediated by microRNA-520b-3p under diverse cellular stress in cancer cells. *PLoS One*.

[20] Sebastiani G., Grieco F. A., Spagnuolo I., Galleri L., Cataldo D., Dotta F. (2011). Increased expression of microRNA miR-326 in type 1 diabetic patients with ongoing islet autoimmunity. *Diabetes/Metabolism Research and Reviews*.

[21] Li Q. J., Chau J., Ebert P. J. (2007). miR-181a is an intrinsic modulator of T cell sensitivity and selection. *Cell*.

[22] Scherm M. G., Daniel C. (2020). miRNA regulation of T Cells in islet autoimmunity and type 1 diabetes. *Current Diabetes Reports*.

[23] Warth S. C., Hoefig K. P., Hiekel A. (2015). Induced miR-99a expression represses Mtor cooperatively with miR-150 to promote regulatory T-cell differentiation. *The EMBO Journal*.

[24] Scherm M., Serr I., Kaestner K. H., Daniel C. (2019). The role of T cell miRNAs for regulatory T cell induction in islet autoimmunity. *Molecular Metabolism*.

[25] Liu C., Li N., Liu G. (2020). The role of microRNAs in regulatory T cells. *Journal of Immunology Research*.

[26] Shu Y., Hu Q., Long H., Chang C., Lu Q., Xiao R. (2017). Epigenetic variability of CD4^+^CD25^+^ Tregs contributes to the pathogenesis of autoimmune diseases. *Clinical Reviews in Allergy and Immunology*.

[27] Mohammadnia-Afrouzi M., Hosseini A. Z., Khalili A. (2016). Altered microRNA expression and immunosuppressive cytokine production by regulatory T cells of ulcerative colitis patients. *Immunological Investigations*.

[28] Jeker L. T., Zhou X., Gershberg K. (2012). MicroRNA 10a marks regulatory T cells. *PLoS One*.

[29] Yao T., Zha D., Gao P., Shui H., Wu X. (2018). MiR-874 alleviates renal injury and inflammatory response in diabetic nephropathy through targeting toll-like receptor-4. *Journal of Cellular Physiology*.

[30] Jalabert A., Vial G., Guay C. (2016). Exosome-like vesicles released from lipid-induced insulin-resistant muscles modulate gene expression and proliferation of beta recipient cells in mice. *Diabetologia*.

[31] Gao X., Zhao S. (2020). miRNA-16-5p inhibits the apoptosis of high glucose-induced pancreatic *β* cells via targeting of CXCL10: potential biomarkers in type 1 diabetes mellitus. *Endokrynologia Polska*.

[32] Esteves J. V., Enguita F. J., Machado U. F. (2017). MicroRNAs-mediated regulation of skeletal muscle GLUT4 expression and translocation in insulin resistance. *Journal Diabetes Research*.

[33] Bras J. P., Silva A. M., Calin G. A., Barbosa M. A., Santos S. G., Almeida M. I. (2017). miR-195 inhibits macrophages pro-inflammatory profile and impacts the crosstalk with smooth muscle cells. *PLoS One*.

[34] Joglekar M. V., Joglekar V. M., Hardikar A. A. (2009). Expression of islet-specific microRNAs during human pancreatic development. *Gene Expression Patterns*.

[35] Li Y., Luo T., Wang L., Wu J., Guo S. (2016). MicroRNA-19a-3p enhances the proliferation and insulin secretion, while it inhibits the apoptosis of pancreatic *β* cells via the inhibition of SOCS3. *International Journal of Molecular Medicine*.

[36] Sebastiani G., Valentini M., Grieco G. E. (2017). MicroRNA expression profiles of human iPSCs differentiation into insulin-producing cells. *Acta Diabetologica*.

[37] Fenoglio C., Ridolfi E., Galimberti D., Scarpini E. (2012). MicroRNAs as active players in the pathogenesis of multiple sclerosis. *International Journal of Molecular Sciences*.

[38] Yamada H., Itoh M., Hiratsuka I., Hashimoto S. (2014). Circulating microRNAs in autoimmune thyroid diseases. *Clinical Endocrinology*.

[39] Jeon S. H., Lee K., Lee K. S. (2012). Characterization of the direct physical interaction of nc 886, a cellular non-coding RNA, and PKR. *FEBS Letters*.

[40] Miersch S., Bian X., Wallstrom G. (2013). Serological autoantibody profiling of type 1 diabetes by protein arrays. *Journal of Proteomics*.

[41] Hillmer E. J., Zhang H., Li H. S., Watowich S. S. (2016). STAT3 signaling in immunity. *Cytokine & Growth Factor Reviews*.

[42] Serr I., Scherm M. G., Zahm A. M. (2018). A miRNA181a/NFAT5 axis links impaired T cell tolerance induction with autoimmune type 1 diabetes. *Science Translational Medicine*.

[43] Serr I., Fürst R. W., Ott V. B. (2016). miRNA92a targets KLF2 and the phosphatase PTEN signaling to promote human T follicular helper precursors in T1D islet autoimmunity. *Proceedings of the National Academy of Sciences of the United States of America*.

[44] Yue T., Sun F., Yang C. (2020). The AHR signaling attenuates autoimmune responses during the development of type 1 diabetes. *Frontiers in Immunology*.

[45] Roggli E., Britan A., Gattesco S., Lin-Marq N., Abderrahmani A., Regazzi R. (2010). Involvement of microRNAs in the cytotoxic effects exerted by proinflammatory cytokines on pancreatic beta-cells. *Diabetes*.

