# Supplementary material for: Progression of Type 1 Diabetes: Circulating MicroRNA Expression Profiles Changes from Preclinical to Overt Disease
Source: J Immunol Res. 2022 Jul 19;2022:2734490. doi: 10.1155/2022/2734490 (PMC9325579; doi:10.1155/2022/2734490)
Supplement: Supplementary Materials — Supplementary Table 1S: miRNAs without expression in serum samples. Supplementary Table 2S: pathways related to up-and downregulated miRNAs of cluster A predicted by the miRWalk platform. Supplementary Table 3S: pathways related to upregulated miRNAs of cluster B predicted by the miRWalk platform. Supplementary Table 4S: pathways related to downregulated miRNAs of cluster B predicted by the miRWalk platform. Supplementary Table 5S: most frequent target genes of miRNAs from cluster A of TargetScan. Supplementary Table 6S: most frequent target genes of miRNAs from cluster B by TargetScan. Supplementary Table 7S: ingenuity canonical pathways related to differentially expressed miRNAs' targets. Supplementary Table 8S: reporting guidelines: STREGA. [file 2734490.f1.zip › STREGA (1).docx]

**STrengthening the REporting of Genetic Association studies (STREGA) reporting recommendations, extended from STROBE Statement**

| **Item** | **Item no** | **STROBE Guideline** | **Extension for Genetic Association Studies (STREGA)** | **Page no** |
| --- | --- | --- | --- | --- |
| **Title and Abstract** | 1 | (a) Circulating microRNAs (miRNAs) in the progression of type 1 diabetes (T1D) | ***microRNAs*** |  |
|  |  | (b) potential influence of microRNAs on type 1 diabetes progression, from the initial autoimmune lesion up to severe beta cell dysfunction | hsa-miR-16 and -miR-200a-3p as biomarkers of T1D progression |  |
| **Introduction** |  |  |  |  |
| *Background rationale* | 2 | miRNAs are regulators of gene expression that can be released during tissue damage, been used as biomarkers of destruction or regeneration of beta-cells and of the altered immunological activity. There are still contradictory data comparing miRNA profiles from individuals at different stages of diabetes, from the pre-clinical to recent and long duration type 1 diabetes. There is no clear definition about the effects of age, diabetes duration and glucose levels, considering that metabolic derangements caused by gluco-lipo-toxicity and inflammatory cytokines can change miRNA milieu and interfere with the results |  |  |
| *Objectives* | 3 | We investigated whether there is a differential profile of serum miRNAs at different stages of type 1 diabetes which could suggest their participation in its pathogenesis. | ***the study is the first report of a genetic association and a replication effort,*** |  |
| **Methods** |  |  |  |  |
| *Study design* | 4 | We covered the phases of highly active autoimmune process and those subject to gluco-lipo-toxicity effects, e.g., from the first autoimmune manifestations (islet autoantibodies) without diabetes to recent and long duration type 1 diabetes. |  |  |
| *Setting* | 5 | We analyzed serum levels of 377 miRNAs of 110 individuals divided into four groups: Individuals with islet autoantibodies without diabetes (AbP group;n=25) ; newly diagnosed patients with type 1 diabetes with duration ≤6 months (recent T1D group;n=30); patients with type 1 diabetes with 2 to 5 years of duration (T1D 2-5y group;n=26) and islet autoantibody negative healthy individuals (Control group;n=29). |  |  |
| *Participants* | 6 | 1. (a) **Cohort study –** Give the eligibility criteria, and the sources and methods of selection of participants. Describe methods of follow-up.   **Case–control study –** Groups were defined according to ADA criteria for T1D. Exclusion criteria comprised other types of diabetes, use of medications except insulin, a febrile state within 10 days prior to blood collection, individuals with liver, kidney, thyroid, inflammatory/autoimmune diseases.  Give the eligibility criteria, and the sources and methods of case ascertainment and control selection. Give the rationale for the choice of cases and controls.  **Cross-sectional study –** Give the eligibility criteria, and the sources and methods of selection of participants. | ***Give information on the criteria and methods for selection of subsets of participants from a larger study, when relevant.*** |  |
|  |  | **(b) Cohort study** – For matched studies, give matching criteria and number of exposed and unexposed.  **Case–control study –** Demographic characteristics, such as age, self-reported skin color and sex were similar between groups |  |  |
| *Variables* | 7 | Diagnosis of diabetes was defined by ADA criteria, analyzing glucose ant islet autoantibodies | ***(b) Clearly define genetic exposures (genetic variants) using a widely –used nomenclature system. Identify variables likely to be associated with population stratification (confounding by ethnic origin).*** |  |
| *Data sources measurement* | 8* | RNA/miRNAs were isolated from 200 µL serum samples stored at -80oC using the miRNeasy Serum/Plasma kit (Qiagen, Hilden, Germany) and the reverse transcription reaction was performed using Megaplex™ RT Human Pool A (Thermofisher,USA), TaqMan® MicroRNA Reverse Transcription Kit (Applied Biosystems, Foster City, California, USA). The RT products were pre-amplified according to manufacturer's protocol - Megaplex™ pools for microRNA Expression Analysis (Thermofisher,USA). Real-Time RT-PCR was performed using the TLDA TaqMan® Low Density Array-Card A v2.0 for humans(384 microRNAs), according to manufacturer’s instructions, on the QuantStudio12K Flex (Applied Biosystems, Foster City, California,USA). The potential target of the differentially expressed miRNAs (DEMs) were predicted using miRWalk 2.0, TargetScan Human 7.2 and IPA software (Ingenuity® Pathways Analysis-5.0; Ingenuity Systems-Qiagen,USA). Pathway enrichment analysis was performed using IPA and KEGG database considering only experimentally validated differentially expressed miRNAs’ targets and prioritizing targets related to beta-cell function and autoimmune manifestation. | ***(b) Describe laboratory methods, including source and storage of DNA, genotyping methods and platforms (including the allele calling algorithm used, and its version), error rates and call rates. State the laboratory /centre where genotyping was done. Describe comparability of laboratory methods if there is more than one group. Specify whether genotypes were assigned using all of the data from the study simultaneously or in smaller batches.*** |  |
| *Bias* | 9 | Exclusion criteria comprised other types of diabetes, use of medications except insulin, a febrile state within 10 days prior to blood collection, individuals with liver, kidney, thyroid, inflammatory/autoimmune diseases. | ***(b) For quantitative outcome variables, specify if any investigation of potential bias resulting from pharmacotherapy was undertaken. If relevant, describe the nature and magnitude of the potential bias, and explain what approach was used to deal with this.*** |  |
| *Study size* | 10 | We use the data in the literature. |  |  |
| *Quantitative variables* | 11 | We covered the phases of highly active autoimmune process and those subject to gluco-lipo-toxicity effects, e.g., from the first autoimmune manifestations (islet autoantibodies) without diabetes to recent and long duration type 1 diabetes. | ***If applicable, describe how effects of treatment were dealt with.*** |  |
| *Statistical methods* | 12 | The analysis of miRNAs expression used the Cloud program (Thermo Fisher Scientific-Waltham, MA-USA)- software of the Cτ comparative method. The criteria for the validation of qRT-PCR reactions were exponential and plateau amplification curves. miRNAs with Ct up to 35 were selected. The relative expression of miRNAs was obtained by the comparative method of Ct (2^-ΔΔCt^), using global normalization and Benjamini and Hochberg’s false discovery rate method, and represented as fold change (FC) in relation to Controls.. Variable distributions were verified by the Shapiro–Wilk normality test. Numerical variables with parametric and non-parametric distribution were analyzed by ANOVA and Kruskal–Wallis with Tukey’s or Dunn’s multiple comparisons post-test, respectively. Correlations were performed using the Spearman correlation test. Qualitative variables were compared using chi-square test or Fisher’s exact test (statistical package GraphPad Prism, La Jolla,CA, USA). Data were considered significant at p<0.05. Fisher exact test and the Benjamini and Hochberg’s false discovery rate method were applied to obtain the target pathways in IPA analysis. | ***State software version used and options (or settings) chosen.*** |  |
|  |  | Fasting glucose levels were determined by enzymatic colorimetric assay (LABTEST GOD-ANA, SP, Brazil), HbA1c by HPLC and C-peptide levels by radioimmunoassay (HCP20K, Millipore Corporation, Billerica, MA, USA; normal values>0.5 ng/mL; intra-and inter-assay CVs: 4.5% and 9.3%, respectively). IAA, GADA and IA2A levels were determined by radioimmunoassay (RSR limited, High Bentham, Lancaster, UK; CV<7%). The normal values for 700 healthy controls (3 SD) were <100nU/mL, <25IU/mL and <125 IU/mL respectively. ZnT8A levels were measured by ELISA (KR770-96; Kronus, Boise, Idaho, USA; CV<7%). The normal value in 321 healthy controls was ≤16 IU/mL(3 SD). |  |  |
|  |  | (c) Explain how missing data were addressed. Fifty-nine (15.6%) of the 377 miRNAs evaluated were not expressed in any group and were excluded from analysis |  |  |
|  |  | (d) **Cohort study –** If applicable, explain how loss to follow-up was addressed.  **Case–control study –**Variable distributions were verified by the Shapiro–Wilk normality test. Numerical variables with parametric and non-parametric distribution were analyzed by ANOVA and Kruskal–Wallis with Tukey’s or Dunn’s multiple comparisons post-test, respectively  **Cross-sectional study –** If applicable, describe analytical methods taking account of sampling strategy. |  |  |
|  |  | (e) Describe any sensitivity analyses.not applicable |  |  |
|  |  | Not applicable | ***(f) State whether Hardy- Weinberg equilibrium was considered and, if so, how.*** |  |
|  |  | \|  \| Not applicable \| \| --- \| --- \| | ***(g) Describe any methods used for inferring genotypes or haplotypes.*** |  |
|  |  | Not applicable | ***(h) Describe any methods used to assess or address population stratification.*** |  |
|  |  | the Benjamini and Hochberg’s false discovery rate method | ***(i) Describe any methods used to address multiple comparisons or to control risk of false positive findings.*** |  |
|  |  | Not applicable | ***(j) Describe any methods used to address and correct for relatedness among subjects.*** |  |
| **Results** |  |  |  |  |
| *Participants* | 13* | (a) We analyzed serum levels of 377 miRNAs of 110 individuals divided into four groups: Individuals with islet autoantibodies without diabetes (AbP group;n=25) ; newly diagnosed patients with type 1 diabetes with duration ≤6 months (recent T1D group;n=30); patients with type 1 diabetes with 2 to 5 years of duration (T1D 2-5y group;n=26) and islet autoantibody negative healthy individuals (Control group;n=29). | ***Report numbers of individuals in whom genotyping was attempted and numbers of individuals in whom genotyping was successful.*** |  |
|  |  | (b) Give reasons for non-participation at each stage.-not applicable |  |  |
|  |  | (c) Consider use of a flow diagram. |  |  |
| *Descriptive data* | 14* | Demographic characteristics, such as age, self-reported skin color and sex were similar between groups (Table 1). Patients with type 1 diabetes (both recent and lasting 2-5 years) were similar to each other. Differed from the control group by higher values ​​of glucose, HbA1c, islet autoantibody and lower C-peptide levels (p<0.05). AbP group presented intermediate characteristics: lower HbA1c and IA2A and higher C-peptide levels than both groups with type 1 diabetes. Differed from the Controls by higher IAA and GADA levels. HLA high risk alleles for diabetes (DR3/DR4;DQ2/DQ8) were less frequent in Control group(p<0.05). | ***Consider giving information by genotype.*** |  |
|  |  | (b) Indicate the number of participants with missing data for each variable of interest. Not applicable |  |  |
|  |  | (c) **Cohort study –** Summarize follow-up time, e.g. average and total amount. Not applicable |  |  |
| *Outcome data* | 15* | **Cohort study –** Report numbers of outcome events or summary measures over time. Not applicable | ***Report outcomes***  ***(phenotypes) for each genotype category over time*** |  |
|  |  | **Case–control study –** Report numbers in each exposure category, or summary measures of exposure. Not applicable | ***Report numbers in each genotype category*** |  |
|  |  | **Cross-sectional study –** Report numbers of outcome events or summary measures. Not applicable | ***Report outcomes (phenotypes) for each genotype category*** |  |
| *Main results* | 16 | (a) miRNA expression change from the initial autoimmune lesion (autoantibodies without diabetes) up to severe beta-cell dysfunction. Several miRNAs were correlated with islet autoantibodies, age and C-peptide levels (pointing these miRNAs as relevant to type 1 diabetes pathogenesis and progression) and to glucose control. miRNAs’ gene targets were related to inflammation, metabolic derangements and decreased immunomodulation. ROC curve analysis suggested miR-16-5p and miR-200a-3p as type 1 diabetes predictors, with higher discriminating power than glycemia |  |  |
|  |  | (b) Report category boundaries when continuous variables were categorized. Not applicable |  |  |
|  |  | (c) If relevant, consider translating estimates of relative risk into absolute risk for a meaningful time period. |  |  |
|  |  |  | ***(d) Report results of any adjustments for multiple comparisons.*** |  |
| *Other analyses* | 17 | (a) correlations with clinical and laboratory data |  |  |
|  |  |  | ***(b) If numerous genetic exposures (genetic variants) were examined, summarize results from all analyses undertaken.*** |  |
|  |  |  | ***(c) If detailed results are available elsewhere, state how they can be accessed.*** |  |
| **Discussion** |  |  |  |  |
| *Key results* | 18 | The results revealed 69 differentially expressed miRNAs (DEMs) in relation to controls. Several miRNAs were correlated with islet autoantibodies (IA2A, GADA and Znt8A), age, and C peptide levels, mainly from AbP and recent T1D groups pointing these miRNAs as relevant to type 1 diabetes pathogenesis and progression. Several miRNAs were related to metabolic derangements, inflammatory pathways and several other autoimmune diseases. Pathway analysis for DEMs targets revealed an enrichment in pathways related to metabolic syndrome, inflammatory response , apoptosis and insulin signaling pathways.Pathway analysis of DEM targets revealed an enrichment in pathways related to inflammatory response, apoptosis, insulin signaling pathways, metabolic derangements and decreased immunomodulation. One of the miRNAs’ gene targets was DYRK2 (dual specificity tyrosine-phosphorylation-regulated kinase 2) which is an autoantibody targeting an antigen in type 1 diabetes. ROC curve analysis showed hsa-miR-16 and hsa-miR-200a-3p with AUCs greater than for the glucose levels, with discriminating power for type 1 diabetes prediction greater then glucose levels |  |  |
| *Limitations* | 19 | low serum RNA yield. |  |  |
| *Interpretation* | 20 | our data suggests potential influence of miRNAs on disease progression, from the initial autoimmune lesion up to severe beta cell dysfunction . As special role of miRNAs: hsa-miR-16 and -miR-200a-3p was suggested as biomarkers of T1D progression, |  |  |
| *Generalizability* | 21 | miRNAs can be potential biomarker and targets for beta cell recovery |  |  |
| **Other information** |  |  |  |  |
| *Funding* | 22 | This research was funded by São Paulo Research Foundation (FAPESP- Process number 2019/06664-4), and Fundação de Pesquisa do Estado de Minas Gerais (FAPEMIG) and Brazilian National Institute of Science and Technology for Vaccines – all non-profit foundations. The funders had no role in the design of the study; in the collection, analyses, or interpretation of data; in the writing of the manuscript, or in the decision to publish the results |  |  |

STROBE: STtrengthening the Reporting of Observational Studies in Epidemiology

*Give information separately for cases and controls in case–control studies and, if applicable, for exposed and unexposed groups in cohort and cross-sectional studies.
