# Supplementary material for: Progression of Type 1 Diabetes: Circulating MicroRNA Expression Profiles Changes from Preclinical to Overt Disease
Source: J Immunol Res. 2022 Jul 19;2022:2734490. doi: 10.1155/2022/2734490 (PMC9325579; doi:10.1155/2022/2734490)
Supplement: Supplementary Materials — Supplementary Table 1S: miRNAs without expression in serum samples. Supplementary Table 2S: pathways related to up-and downregulated miRNAs of cluster A predicted by the miRWalk platform. Supplementary Table 3S: pathways related to upregulated miRNAs of cluster B predicted by the miRWalk platform. Supplementary Table 4S: pathways related to downregulated miRNAs of cluster B predicted by the miRWalk platform. Supplementary Table 5S: most frequent target genes of miRNAs from cluster A of TargetScan. Supplementary Table 6S: most frequent target genes of miRNAs from cluster B by TargetScan. Supplementary Table 7S: ingenuity canonical pathways related to differentially expressed miRNAs' targets. Supplementary Table 8S: reporting guidelines: STREGA. [file 2734490.f1.zip › Suppl 1 miRNAs without expression in serum samples.pdf]

Supplementary Table 1S: miRNAs without expression in serum samples

Assay ID	Assay Name	Pool Card	Control Group 29	recent T1D Group 31	T1D 2-5y Group 26	AbP Group 25
000398	hsa-miR-22	A	0	0	0	0
002085	hsa-miR-33b	A	0	0	0	0
001184	mmu-miR-129-3p	A	0	0	0	0
001129	mmu-miR-137	A	0	0	0	0
000477	hsa-miR-154	A	0	0	0	0
002106	hsa-miR-188-3p	A	0	0	0	0
002250	hsa-miR-193a-3p	A	0	0	0	0
002290	hsa-miR-208b	A	0	0	0	0
002220	hsa-miR-216a	A	0	0	0	0
002326	hsa-miR-216b	A	0	0	0	0
002337	hsa-miR-217	A	0	0	0	0
001101	hsa-miR-329	A	0	0	0	0
002230	hsa-miR-330-5p	A	0	0	0	0
002233	hsa-miR-331-5p	A	0	0	0	0
000557	hsa-miR-369-3p	A	0	0	0	0
001021	hsa-miR-369-5p	A	0	0	0	0
002124	hsa-miR-371-3p	A	0	0	0	0
000560	hsa-miR-372	A	0	0	0	0
001102	hsa-miR-376b	A	0	0	0	0
000566	hsa-miR-377	A	0	0	0	0
000569	hsa-miR-380-3p	A	0	0	0	0
001608	hsa-miR-449b	A	0	0	0	0
002303	hsa-miR-450a	A	0	0	0	0
002208	hsa-miR-450b-3p	A	0	0	0	0
002318	hsa-miR-453	A	0	0	0	0
001051	hsa-miR-507	A	0	0	0	0
002092	hsa-miR-508-5p	A	0	0	0	0
002241	hsa-miR-510	A	0	0	0	0
001145	hsa-miR-512-5p	A	0	0	0	0
002090	hsa-miR-513-5p	A	0	0	0	0
002416	hsa-miR-516a-5p	A	0	0	0	0
002401	hsa-miR-518c	A	0	0	0	0
002389	hsa-miR-518d-5p	A	0	0	0	0
002395	hsa-miR-518e	A	0	0	0	0
001168	hsa-miR-520a#	A	0	0	0	0
001982	hsa-miR-524-5p	A	0	0	0	0
002385	hsa-miR-525-3p	A	0	0	0	0
002412	hsa-miR-548a-5p	A	0	0	0	0
001541	hsa-miR-548b	A	0	0	0	0
002345	hsa-miR-556-3p	A	0	0	0	0
002344	hsa-miR-556-5p	A	0	0	0	0
002350	hsa-miR-576-5p	A	0	0	0	0
001983	hsa-miR-582-5p	A	0	0	0	0
002430	hsa-miR-624	A	0	0	0	0
002292	hsa-miR-653	A	0	0	0	0
002239	hsa-miR-654-3p	A	0	0	0	0
002205	hsa-miR-876-5p	A	0	0	0	0
002195	hsa-miR-892a	A	0	0	0	0
002095	hsa-miR-219-1-3p	A	0	0	0	0
002390	hsa-miR-219-2-3p	A	0	0	0	0
000523	hsa-miR-220	A	0	0	0	0
002211	hsa-miR-220c	A	0	0	0	0
000540	hsa-miR-325	A	0	0	0	0
000574	hsa-miR-384	A	0	0	0	0
001029	hsa-miR-448	A	0	0	0	0
001039	hsa-miR-492	A	0	0	0	0
002155	hsa-miR-509-3-5p	A	0	0	0	0
001163	hsa-miR-519c	A	0	0	0	0
001119	hsa-miR-520e	A	0	0	0	0

AbP Group (individuals without diabetes expressing islet autoantibody);
Recent T1D Group- (newly diagnosed patients with type 1 diabetes with duration ≤ 6 months)
T1D 2-5y Group- (patients with type 1 diabetes with 2 to 5 years of duration and health Control Group)
