# Supplementary material for: Progression of Type 1 Diabetes: Circulating MicroRNA Expression Profiles Changes from Preclinical to Overt Disease
Source: J Immunol Res. 2022 Jul 19;2022:2734490. doi: 10.1155/2022/2734490 (PMC9325579; doi:10.1155/2022/2734490)
Supplement: Supplementary Materials — Supplementary Table 1S: miRNAs without expression in serum samples. Supplementary Table 2S: pathways related to up-and downregulated miRNAs of cluster A predicted by the miRWalk platform. Supplementary Table 3S: pathways related to upregulated miRNAs of cluster B predicted by the miRWalk platform. Supplementary Table 4S: pathways related to downregulated miRNAs of cluster B predicted by the miRWalk platform. Supplementary Table 5S: most frequent target genes of miRNAs from cluster A of TargetScan. Supplementary Table 6S: most frequent target genes of miRNAs from cluster B by TargetScan. Supplementary Table 7S: ingenuity canonical pathways related to differentially expressed miRNAs' targets. Supplementary Table 8S: reporting guidelines: STREGA. [file 2734490.f1.zip › Suppl 2 MiRWalk pathway Cluster A.pdf]

Supplementary Table 2S: **Pathways related to up and down-regulated miRNAs of Cluster A predicted at miRWalk platform**

miRNAs	miRNA	PathName	PathFg	PathBg	GenomeFG	GenomeBG	Fischer exact test	BH false discovery rate
up-regulated	hsa-miR-148b-3p	Acute myeloid leukemia	33	58	6712	19747	0.000285321978653082	0.0447955506485338
	hsa-miR-296-5p	Acute myeloid leukemia	36	58	7558	19747	0.000202594692899396	0.0330229349426015
	hsa-miR-874-3p	Acute myeloid leukemia	37	58	7993	19747	0.000279113831917186	0.0457746684344186
	hsa-miR-148b-3p	Adherens junction	43	76	6712	19747	4,37E+09	0.00734140859202926
	hsa-miR-181a-5p	Adherens junction	51	76	8649	19747	3,42E+09	0.00602252004514624
	hsa-miR-200a-3p	Adherens junction	44	76	6375	19747	3,66E+08	0.00068069000805142
	hsa-miR-323a-3p	Adherens junction	49	76	7613	19747	4,14E+07	0.000777504373420887
	hsa-miR-326	Adherens junction	50	76	8828	19747	0.000167207811404726	0.0280909123159939
	hsa-miR-330-3p	Adherens junction	55	76	9087	19747	2,79E+08	0.000496287963766962
	hsa-miR-181a-5p	Adipocytokine signaling pathway	46	70	8649	19747	0.000176221912627448	0.0301339470592937
	hsa-miR-296-5p	Adipocytokine signaling pathway	42	70	7558	19747	0.000184318259767953	0.0302281946019443
	hsa-miR-200a-3p	Aldosterone regulated sodium reabsorption	27	42	6375	19747	2,05E+08	0.00375126517911573
	hsa-miR-323a-3p	Aldosterone regulated sodium reabsorption	28	42	7613	19747	0.000206409941785475	0.0357089199288872
	hsa-miR-296-5p	Apoptosis	50	87	7558	19747	0.000213575656291668	0.0345992563192502
	hsa-miR-874-3p	Apoptosis	56	87	7993	19747	5,62E+08	0.00101794294378252
	hsa-miR-10a-5p	Axon guidance	66	129	6252	19747	3,10E+08	0.00059167423294286
	hsa-miR-148b-3p	Axon guidance	67	129	6712	19747	1,96E+09	0.00340077945517526
	hsa-miR-181a-5p	Axon guidance	82	129	8649	19747	4,58E+08	0.00083744744068186
	hsa-miR-200a-3p	Axon guidance	67	129	6375	19747	2,82E+08	0.000523804547378212
	hsa-miR-296-5p	Axon guidance	77	129	7558	19747	6,39E+07	0.000119578405828185
	hsa-miR-323a-3p	Axon guidance	71	129	7613	19747	0.00010261879733185	0.0180609083304056
	hsa-miR-326	Axon guidance	83	129	8828	19747	5,25E+08	0.000981986349579498
	hsa-miR-330-3p	Axon guidance	86	129	9087	19747	1,69E+08	0.000304289223830338
	hsa-miR-874-3p	Axon guidance	88	129	7993	19747	1,59E+04	3,09E+06
	hsa-miR-874-3p	B cell receptor signaling pathway	46	75	7993	19747	0.000208595599439451	0.0344182739075094
	hsa-miR-518b	Basal cell carcinoma	22	55	3755	19747	0.000247807952755696	0.0431185837794912
	hsa-miR-874-3p	Basal cell carcinoma	36	55	7993	19747	0.000157082494016766	0.0262327765007999
	hsa-miR-148b-3p	Calcium signaling pathway	83	178	6712	19747	0.000307153428072526	0.0482230882073866
	hsa-miR-296-5p	Calcium signaling pathway	101	178	7558	19747	4,11E+07	7,73E+09
	hsa-miR-326	Calcium signaling pathway	114	178	8828	19747	1,44E+06	2,77E+09
	hsa-miR-874-3p	Calcium signaling pathway	101	178	7993	19747	7,97E+08	0.00144221627003443
	hsa-miR-148b-3p	Cell adhesion molecules CAMs	73	133	6712	19747	5,72E+07	0.000105296907884733
	hsa-miR-326	Cell adhesion molecules CAMs	89	133	8828	19747	1,84E+07	3,52E+09
	hsa-miR-518b	Cell cycle	42	124	3755	19747	6,16E+09	0.0112719271275947
	hsa-miR-296-5p	Chemokine signaling pathway	98	189	7558	19747	9,55E+08	0.016048813612924
	hsa-miR-323a-3p	Chemokine signaling pathway	101	189	7613	19747	2,16E+09	0.00390426576915299
	hsa-miR-296-5p	Chondroitin sulfate biosynthesis	17	22	7558	19747	0.00022754649233914	0.0366349852666015
	hsa-miR-10a-5p	Chronic myeloid leukemia	41	75	6252	19747	3,03E+09	0.0055512957471532
	hsa-miR-148b-3p	Chronic myeloid leukemia	51	75	6712	19747	1,84E+05	3,51E+07
	hsa-miR-181a-5p	Chronic myeloid leukemia	56	75	8649	19747	5,35E+06	1,02E+09
	hsa-miR-200a-3p	Chronic myeloid leukemia	45	75	6375	19747	7,14E+07	0.000134280870679786
	hsa-miR-296-5p	Chronic myeloid leukemia	51	75	7558	19747	1,70E+07	3,23E+09
	hsa-miR-323a-3p	Chronic myeloid leukemia	53	75	7613	19747	1,66E+05	3,18E+08
	hsa-miR-330-3p	Chronic myeloid leukemia	58	75	9087	19747	2,92E+06	5,54E+08
	hsa-miR-874-3p	Chronic myeloid leukemia	52	75	7993	19747	3,93E+07	7,35E+08
	hsa-miR-10a-5p	Colorectal cancer	46	86	6252	19747	2,16E+09	0.00395941475191675
	hsa-miR-148b-3p	Colorectal cancer	48	86	6712	19747	2,62E+09	0.00450490698679465
	hsa-miR-181a-5p	Colorectal cancer	57	86	8649	19747	2,12E+09	0.00374721368720921
	hsa-miR-200a-3p	Colorectal cancer	47	86	6375	19747	1,47E+09	0.00269621174986488
	hsa-miR-296-5p	Colorectal cancer	50	86	7558	19747	0.000144053551114382	0.0240569430361017
	hsa-miR-323a-3p	Colorectal cancer	56	86	7613	19747	5,35E+07	0.000102097454417187
	hsa-miR-326	Colorectal cancer	56	86	8828	19747	0.000106165882434942	0.018154365896375
	hsa-miR-330-3p	Colorectal cancer	64	86	9087	19747	7,66E+05	1,45E+08
	hsa-miR-518b	Colorectal cancer	34	86	3755	19747	7,82E+08	0.0014701549981444
	hsa-miR-874-3p	Colorectal cancer	57	86	7993	19747	1,15E+08	0.000212132522388711
	hsa-miR-10a-5p	Endocytosis	87	187	6252	19747	1,41E+09	0.00259531252280087
	hsa-miR-148b-3p	Endocytosis	91	187	6712	19747	2,24E+09	0.00387897987769692
	hsa-miR-181a-5p	Endocytosis	111	187	8649	19747	1,25E+09	0.00221712268364503
	hsa-miR-296-5p	Endocytosis	108	187	7558	19747	4,72E+06	9,07E+08
	hsa-miR-323a-3p	Endocytosis	99	187	7613	19747	4,25E+09	0.00755730554978099
	hsa-miR-326	Endocytosis	113	187	8828	19747	1,03E+09	0.00189750617134004
	hsa-miR-330-3p	Endocytosis	119	187	9087	19747	8,44E+07	0.000155303796537319
	hsa-miR-874-3p	Endocytosis	105	187	7993	19747	1,00E+09	0.00180198880654956
	hsa-miR-296-5p	Endometrial cancer	35	52	7558	19747	2,07E+09	0.00365883659149937
	hsa-miR-323a-3p	Endometrial cancer	34	52	7613	19747	7,98E+09	0.0140518329641817
	hsa-miR-326	Endometrial cancer	39	52	8828	19747	8,68E+08	0.00161358061496216
	hsa-miR-330-3p	Endometrial cancer	40	52	9087	19747	5,34E+08	0.000944666499558501

hsa-miR-874-3p	Endometrial cancer	34	52	7993	19747	0.000245462363950473	0.0402558276878776
hsa-miR-10a-5p	ErbB signaling pathway	47	89	6252	19747	2,74E+09	0.00501467880362049
hsa-miR-148b-3p	ErbB signaling pathway	52	89	6712	19747	1,96E+08	0.000357196916789751
hsa-miR-181a-5p	ErbB signaling pathway	65	89	8649	19747	2,04E+06	3,91E+08
hsa-miR-200a-3p	ErbB signaling pathway	53	89	6375	19747	1,07E+07	2,05E+09
hsa-miR-296-5p	ErbB signaling pathway	53	89	7558	19747	3,74E+09	0.00643852223263768
hsa-miR-323a-3p	ErbB signaling pathway	55	89	7613	19747	7,37E+07	0.00136338254977881
hsa-miR-326	ErbB signaling pathway	61	89	8828	19747	4,63E+08	0.000870885953942753
hsa-miR-330-3p	ErbB signaling pathway	66	89	9087	19747	6,06E+06	1,15E+09
hsa-miR-518b	ErbB signaling pathway	33	89	3755	19747	4,96E+09	0.00912196117337061
hsa-miR-874-3p	ErbB signaling pathway	61	89	7993	19747	7,69E+06	1,46E+09
hsa-miR-148b-3p	Fc gamma R mediated phagocytosis	52	97	6712	19747	5,42E+09	0.00904838401552313
hsa-miR-326	Fc gamma R mediated phagocytosis	61	97	8828	19747	0.000231364660766659	0.0384065336872654
hsa-miR-10a-5p	Focal adhesion	90	203	6252	19747	9,56E+09	0.0172155350711822
hsa-miR-148b-3p	Focal adhesion	94	203	6712	19747	0.000174873930788744	0.0278049549954102
hsa-miR-181a-5p	Focal adhesion	118	203	8649	19747	2,60E+09	0.00458468297621582
hsa-miR-323a-3p	Focal adhesion	105	203	7613	19747	8,75E+09	0.0154051941927141
hsa-miR-326	Focal adhesion	119	203	8828	19747	4,34E+09	0.00776791899730148
hsa-miR-148b-3p	Gap junction	48	90	6712	19747	0.000122040593190416	0.0197705760968474
hsa-miR-10a-5p	Glioma	39	65	6252	19747	2,27E+08	0.00043434516720938
hsa-miR-148b-3p	Glioma	48	65	6712	19747	5,77E+03	1,11E+06
hsa-miR-181a-5p	Glioma	49	65	8649	19747	2,20E+06	4,17E+09
hsa-miR-200a-3p	Glioma	38	65	6375	19747	1,21E+09	0.00222332605376092
hsa-miR-296-5p	Glioma	46	65	7558	19747	1,06E+07	2,01E+09
hsa-miR-323a-3p	Glioma	42	65	7613	19747	1,84E+09	0.00334164601536715
hsa-miR-326	Glioma	46	65	8828	19747	1,87E+09	0.00341382017337973
hsa-miR-330-3p	Glioma	48	65	9087	19747	4,70E+08	0.000832350347416637
hsa-miR-518b	Glioma	26	65	3755	19747	7,02E+09	0.0127779599839795
hsa-miR-874-3p	Glioma	47	65	7993	19747	1,93E+07	3,65E+08
hsa-miR-148b-3p	GnRH signaling pathway	55	105	6712	19747	7,78E+09	0.0127520909591378
hsa-miR-181a-5p	GnRH signaling pathway	67	105	8649	19747	2,76E+09	0.00485942381756834
hsa-miR-296-5p	GnRH signaling pathway	61	105	7558	19747	2,91E+09	0.00503673404228545
hsa-miR-326	GnRH signaling pathway	67	105	8828	19747	6,05E+09	0.0106487026912501
hsa-miR-330-3p	GnRH signaling pathway	67	105	9087	19747	0.000176841130248246	0.0295324687514571
hsa-miR-874-3p	GnRH signaling pathway	61	105	7993	19747	0.000193908757393555	0.0321888537273302
hsa-miR-10a-5p	Insulin signaling pathway	68	139	6252	19747	1,58E+09	0.00290515973342576
hsa-miR-148b-3p	Insulin signaling pathway	74	139	6712	19747	2,27E+08	0.000412851070962132
hsa-miR-200a-3p	Insulin signaling pathway	67	139	6375	19747	6,53E+09	0.0117488331588134
hsa-miR-296-5p	Insulin signaling pathway	79	139	7558	19747	6,62E+08	0.00120396409401159
hsa-miR-323a-3p	Insulin signaling pathway	78	139	7613	19747	1,92E+07	0.00350216809322562
hsa-miR-330-3p	Insulin signaling pathway	93	139	9087	19747	5,07E+07	9,37E+08
hsa-miR-874-3p	Insulin signaling pathway	91	139	7993	19747	2,14E+04	4,08E+07
hsa-miR-148b-3p	Leukocyte transendothelial migration	59	116	6712	19747	0.00012863245733034	0.0207098256301847
hsa-miR-148b-3p	Long term potentiation	41	71	6712	19747	3,41E+09	0.0058034570789362
hsa-miR-296-5p	Long term potentiation	43	71	7558	19747	0.000114680488458761	0.019151641572613
hsa-miR-323a-3p	Long term potentiation	46	71	7613	19747	6,67E+08	0.00124015663744594
hsa-miR-326	Long term potentiation	49	71	8828	19747	2,96E+09	0.0053619800958715
hsa-miR-330-3p	Long term potentiation	50	71	9087	19747	2,68E+09	0.00469533378091935
hsa-miR-874-3p	Long term potentiation	44	71	7993	19747	0.000203443310594397	0.0335681462480756
hsa-miR-296-5p	Lysosome	68	121	7558	19747	4,61E+09	0.00783235045116317
hsa-miR-100-5p	MAPK signaling pathway	53	272	2341	19747	0.000174902938959609	0.0318323348906489
hsa-miR-148b-3p	MAPK signaling pathway	121	272	6712	19747	0.000192107079953319	0.0305450257125777
hsa-miR-181a-5p	MAPK signaling pathway	166	272	8649	19747	6,66E+05	1,29E+08
hsa-miR-200a-3p	MAPK signaling pathway	117	272	6375	19747	0.000122356176811222	0.0219017556492088
hsa-miR-296-5p	MAPK signaling pathway	149	272	7558	19747	2,05E+06	3,96E+08
hsa-miR-326	MAPK signaling pathway	159	272	8828	19747	3,14E+08	0.000593161113504099
hsa-miR-874-3p	MAPK signaling pathway	159	272	7993	19747	1,34E+05	2,57E+07
hsa-miR-148b-3p	Melanogenesis	53	102	6712	19747	0.000136812966587511	0.0220268876205892
hsa-miR-296-5p	Melanogenesis	63	102	7558	19747	1,26E+08	0.000234569664481463
hsa-miR-330-3p	Melanogenesis	70	102	9087	19747	3,17E+07	0.000564109670148666
hsa-miR-518b	Melanogenesis	36	102	3755	19747	7,71E+09	0.0139498259206678
hsa-miR-874-3p	Melanogenesis	62	102	7993	19747	2,66E+08	0.00473042401678753
hsa-miR-148b-3p	Melanoma	47	71	6712	19747	2,95E+06	5,54E+08
hsa-miR-181a-5p	Melanoma	46	71	8649	19747	0.000288895951975274	0.0488234158838212
hsa-miR-296-5p	Melanoma	43	71	7558	19747	0.000114680488458761	0.019151641572613
hsa-miR-10a-5p	mTOR signaling pathway	30	53	6252	19747	0.000149370880602196	0.0267373876277931
hsa-miR-148b-3p	mTOR signaling pathway	32	53	6712	19747	7,47E+09	0.0122564656056605
hsa-miR-326	mTOR signaling pathway	38	53	8828	19747	6,25E+09	0.0109296630440125
hsa-miR-10a-5p	Neurotrophin signaling pathway	73	129	6252	19747	4,03E+05	7,82E+07

hsa-miR-148b-3p	Neurotrophin signaling pathway	71	129	6712	19747	7,07E+06	0.000130080356258607
hsa-miR-181a-5p	Neurotrophin signaling pathway	84	129	8649	19747	8,14E+07	0.000153783367055407
hsa-miR-200a-3p	Neurotrophin signaling pathway	72	129	6375	19747	2,81E+06	5,40E+08
hsa-miR-296-5p	Neurotrophin signaling pathway	73	129	7558	19747	1,79E+09	0.00318180637589007
hsa-miR-323a-3p	Neurotrophin signaling pathway	82	129	7613	19747	7,26E+05	1,40E+08
hsa-miR-326	Neurotrophin signaling pathway	79	129	8828	19747	0.000111440942756793	0.0190564012114116
hsa-miR-330-3p	Neurotrophin signaling pathway	94	129	9087	19747	4,97E+04	9,55E+06
hsa-miR-518b	Neurotrophin signaling pathway	45	129	3755	19747	1,48E+09	0.00278210339561635
hsa-miR-874-3p	Neurotrophin signaling pathway	79	129	7993	19747	1,47E+08	0.000271198965501805
hsa-miR-10a-5p	Non small cell lung cancer	35	54	6252	19747	5,54E+07	0.00010688884587335
hsa-miR-148b-3p	Non small cell lung cancer	39	54	6712	19747	1,06E+06	2,00E+08
hsa-miR-181a-5p	Non small cell lung cancer	39	54	8649	19747	2,16E+09	0.00382186698717631
hsa-miR-296-5p	Non small cell lung cancer	37	54	7558	19747	6,35E+08	0.00115634206815017
hsa-miR-326	Non small cell lung cancer	38	54	8828	19747	0.000120038061856215	0.0204064705155566
hsa-miR-330-3p	Non small cell lung cancer	41	54	9087	19747	7,25E+08	0.00128312563707357
hsa-miR-874-3p	Non small cell lung cancer	38	54	7993	19747	8,29E+08	0.00149971612832384
hsa-miR-148b-3p	Pancreatic cancer	48	75	6712	19747	1,03E+07	1,93E+09
hsa-miR-296-5p	Pancreatic cancer	48	75	7558	19747	5,55E+08	0.00100977293598756
hsa-miR-323a-3p	Pancreatic cancer	45	75	7613	19747	0.000134098789962	0.02346728824335
hsa-miR-330-3p	Pancreatic cancer	56	75	9087	19747	4,12E+07	7,66E+09
hsa-miR-874-3p	Pancreatic cancer	46	75	7993	19747	0.000208595599439451	0.0344182739075094
hsa-miR-10a-5p	Pathways in cancer	140	330	6252	19747	2,20E+09	0.00401701312095859
hsa-miR-148b-3p	Pathways in cancer	169	330	6712	19747	6,60E+03	1,27E+06
hsa-miR-181a-5p	Pathways in cancer	201	330	8649	19747	2,26E+04	4,38E+06
hsa-miR-200a-3p	Pathways in cancer	148	330	6375	19747	1,04E+08	0.000196208333384185
hsa-miR-296-5p	Pathways in cancer	168	330	7558	19747	1,74E+08	0.000321837196522258
hsa-miR-323a-3p	Pathways in cancer	159	330	7613	19747	0.000208372932320788	0.0360485172914964
hsa-miR-326	Pathways in cancer	196	330	8828	19747	4,65E+06	9,02E+08
hsa-miR-330-3p	Pathways in cancer	213	330	9087	19747	6,66E+02	1,29E+05
hsa-miR-518b	Pathways in cancer	108	330	3755	19747	1,65E+05	3,13E+07
hsa-miR-874-3p	Pathways in cancer	186	330	7993	19747	3,20E+05	6,11E+07
hsa-miR-148b-3p	Phosphatidylinositol signaling system	47	76	6712	19747	6,22E+07	0.000114511779911665
hsa-miR-181a-5p	Phosphatidylinositol signaling system	51	76	8649	19747	3,42E+09	0.00602252004514624
hsa-miR-296-5p	Phosphatidylinositol signaling system	45	76	7558	19747	0.000170111699048163	0.028068430342947
hsa-miR-323a-3p	Phosphatidylinositol signaling system	47	76	7613	19747	3,25E+09	0.00585170438566875
hsa-miR-148b-3p	Progesterone mediated oocyte maturation	48	88	6712	19747	5,80E+09	0.00963583040917686
hsa-miR-330-3p	Progesterone mediated oocyte maturation	59	88	9087	19747	5,41E+09	0.00941654091545895
hsa-miR-10a-5p	Prostate cancer	45	89	6252	19747	0.000156155368987277	0.0279518110487226
hsa-miR-148b-3p	Prostate cancer	56	89	6712	19747	2,23E+06	4,19E+08
hsa-miR-181a-5p	Prostate cancer	59	89	8649	19747	1,51E+09	0.00269053656475528
hsa-miR-200a-3p	Prostate cancer	47	89	6375	19747	4,76E+09	0.00861537560086126
hsa-miR-323a-3p	Prostate cancer	53	89	7613	19747	4,71E+09	0.00838772579560752
hsa-miR-330-3p	Prostate cancer	62	89	9087	19747	5,32E+08	0.0009413235058902
hsa-miR-518b	Prostate cancer	32	89	3755	19747	0.000126398064754896	0.0224988555263714
hsa-miR-874-3p	Prostate cancer	54	89	7993	19747	9,22E+09	0.0156691562226247
hsa-miR-874-3p	Regulation of actin cytoskeleton	111	212	7993	19747	0.000290433413936188	0.0476310798855348
hsa-miR-10a-5p	Renal cell carcinoma	39	71	6252	19747	4,07E+09	0.00740697451753858
hsa-miR-148b-3p	Renal cell carcinoma	40	71	6712	19747	9,08E+09	0.0148065447442863
hsa-miR-181a-5p	Renal cell carcinoma	49	71	8649	19747	1,52E+09	0.00270797756118686
hsa-miR-330-3p	Renal cell carcinoma	53	71	9087	19747	8,51E+07	0.000156668677241968
hsa-miR-181a-5p	Small cell lung cancer	55	84	8649	19747	4,94E+09	0.00869675528600013
hsa-miR-330-3p	Small cell lung cancer	57	84	9087	19747	4,25E+09	0.00740042849180159
hsa-miR-10a-5p	T cell receptor signaling pathway	53	110	6252	19747	0.000213033653275851	0.0374939229765499
hsa-miR-874-3p	T cell receptor signaling pathway	66	110	7993	19747	2,68E+09	0.00477676594997608
hsa-miR-148b-3p	Type II diabetes mellitus	29	49	6712	19747	0.000262890454397964	0.0412738013404803

	hsa-miR-181a-5p	Ubiquitin mediated proteolysis	85	134	8649	19747	3,49E+08	0.000646284436032731
	hsa-miR-330-3p	Ubiquitin mediated proteolysis	87	134	9087	19747	7,57E+08	0.00134036133344036
	hsa-miR-181a-5p	Vascular smooth muscle contraction	74	116	8649	19747	1,09E+09	0.00195012963607873
	hsa-miR-296-5p	VEGF signaling pathway	49	78	7558	19747	9,53E+08	0.00171607347493294
	hsa-miR-874-3p	VEGF signaling pathway	48	78	7993	19747	0.000136253814144846	0.0228906407763341
	hsa-miR-10a-5p	Wnt signaling pathway	72	152	6252	19747	3,59E+09	0.00656496674227214
	hsa-miR-148b-3p	Wnt signaling pathway	74	152	6712	19747	0.00012268786334757	0.0198754338623064
	hsa-miR-200a-3p	Wnt signaling pathway	79	152	6375	19747	3,65E+07	6,90E+09
	hsa-miR-296-5p	Wnt signaling pathway	80	152	7558	19747	0.000215115565454356	0.0348487216036057
	hsa-miR-323a-3p	Wnt signaling pathway	84	152	7613	19747	2,05E+09	0.00372990929875818
	hsa-miR-326	Wnt signaling pathway	93	152	8828	19747	3,04E+09	0.00546358726866268
	hsa-miR-330-3p	Wnt signaling pathway	111	152	9087	19747	1,09E+03	2,11E+05
	hsa-miR-518b	Wnt signaling pathway	61	152	3755	19747	1,20E+05	2,28E+07
	hsa-miR-874-3p	Wnt signaling pathway	91	152	7993	19747	1,01E+08	0.000187554566941811
**Down regulated**	hsa-miR-16-5p	Acute myeloid leukemia	42	58	7756	19747	3,09E+07	5,53E+09
	hsa-miR-195-5p	Acute myeloid leukemia	42	58	8372	19747	3,46E+08	0.00059797124965737
	hsa-miR-590-5p	Adherens junction	47	76	7481	19747	1,92E+09	0.00365068601263296
	hsa-miR-195-5p	Adipocytokine signaling pathway	50	70	8372	19747	8,12E+07	0.000143730007506957
	hsa-miR-25-3p	Adipocytokine signaling pathway	49	70	8526	19747	5,05E+07	0.000909537815281193
	hsa-miR-376a-3p	Adipocytokine signaling pathway	34	70	5595	19747	0.000259110631594339	0.0445670286342263
	hsa-miR-16-5p	Aldosterone regulated sodium reabsorption	28	42	7756	19747	0.000298235902995239	0.0450336213522811
	hsa-miR-376a-3p	Aldosterone regulated sodium reabsorption	23	42	5595	19747	0.000284059348503713	0.0485741485941349
	hsa-miR-25-3p	Amyotrophic lateral sclerosis ALS	37	55	8526	19747	0.000263433010015178	0.0437298796625196
	hsa-miR-16-5p	Apoptosis	53	87	7756	19747	3,53E+09	0.00583025197847097
	hsa-miR-195-5p	Apoptosis	60	87	8372	19747	4,79E+07	8,53E+09
	hsa-miR-25-3p	Apoptosis	62	87	8526	19747	1,02E+07	1,96E+09
	hsa-miR-16-5p	Axon guidance	85	129	7756	19747	8,09E+04	1,55E+07
	hsa-miR-195-5p	Axon guidance	84	129	8372	19747	1,53E+07	2,78E+09
	hsa-miR-19a-3p	Axon guidance	65	129	6553	19747	3,81E+09	0.00700563498143501
	hsa-miR-25-3p	Axon guidance	78	129	8526	19747	5,51E+07	0.00953164333655268
	hsa-miR-376a-3p	Axon guidance	59	129	5595	19747	1,85E+09	0.00332531482677645
	hsa-miR-16-5p	B cell receptor signaling pathway	48	75	7756	19747	1,26E+09	0.00214543606285859
	hsa-miR-25-3p	B cell receptor signaling pathway	51	75	8526	19747	1,21E+09	0.00215334684690487
	hsa-miR-16-5p	Basal cell carcinoma	36	55	7756	19747	7,57E+09	0.0121154036490838
	hsa-miR-195-5p	Calcium signaling pathway	112	178	8372	19747	2,46E+06	4,58E+07
	hsa-miR-19a-3p	Calcium signaling pathway	88	178	6553	19747	4,87E+07	0.00092135208295363
	hsa-miR-25-3p	Calcium signaling pathway	105	178	8526	19747	1,47E+09	0.0025978306363483
	hsa-miR-590-5p	Calcium signaling pathway	93	178	7481	19747	6,38E+09	0.011865075224554
	hsa-miR-25-3p	Chemokine signaling pathway	106	189	8526	19747	0.000227187516970498	0.0379403153340731
	hsa-miR-16-5p	Chronic myeloid leukemia	51	75	7756	19747	4,38E+07	7,79E+09
	hsa-miR-195-5p	Chronic myeloid leukemia	52	75	8372	19747	2,14E+08	0.00037231428006851
	hsa-miR-19a-3p	Chronic myeloid leukemia	43	75	6553	19747	1,45E+09	0.00269824736778531
	hsa-miR-25-3p	Chronic myeloid leukemia	54	75	8526	19747	3,93E+07	7,44E+09
	hsa-miR-376a-3p	Chronic myeloid leukemia	43	75	5595	19747	1,35E+07	2,58E+09
	hsa-miR-590-5p	Chronic myeloid leukemia	47	75	7481	19747	1,16E+09	0.0021980764645095
	hsa-miR-16-5p	Colorectal cancer	55	86	7756	19747	3,11E+08	0.000534992078139792
	hsa-miR-195-5p	Colorectal cancer	59	86	8372	19747	7,88E+07	0.000139473777870939
	hsa-miR-19a-3p	Colorectal cancer	47	86	6553	19747	3,28E+09	0.00606694254649142
	hsa-miR-25-3p	Colorectal cancer	59	86	8526	19747	1,63E+08	0.000299568910335733
	hsa-miR-376a-3p	Colorectal cancer	43	86	5595	19747	1,72E+09	0.00309869983202704
	hsa-miR-590-5p	Colorectal cancer	50	86	7481	19747	0.000106925064311295	0.0196742118332782
	hsa-miR-16-5p	Endocytosis	111	187	7756	19747	1,99E+06	3,70E+08
	hsa-miR-195-5p	Endocytosis	117	187	8372	19747	1,94E+06	3,62E+07
	hsa-miR-19a-3p	Endocytosis	102	187	6553	19747	1,31E+05	2,55E+07
	hsa-miR-25-3p	Endocytosis	115	187	8526	19747	3,16E+07	6,00E+09
	hsa-miR-376a-3p	Endocytosis	80	187	5595	19747	1,54E+09	0.00276556684619012
	hsa-miR-590-5p	Endocytosis	101	187	7481	19747	4,99E+08	0.00095887954000356
	hsa-miR-16-5p	Endometrial cancer	35	52	7756	19747	3,94E+08	0.00646138283909611
	hsa-miR-195-5p	Endometrial cancer	35	52	8372	19747	0.000248009610654232	0.0376974608194433
	hsa-miR-16-5p	ErbB signaling pathway	56	89	7756	19747	5,32E+07	0.000910309375262143
	hsa-miR-195-5p	ErbB signaling pathway	56	89	8372	19747	7,39E+09	0.0117472811082527
	hsa-miR-19a-3p	ErbB signaling pathway	46	89	6553	19747	0.000232851130463537	0.0395846921788013
	hsa-miR-25-3p	ErbB signaling pathway	59	89	8526	19747	8,88E+08	0.00159039266883306

hsa-miR-376a-3p	ErbB signaling pathway	49	89	5595	19747	1,07E+06	2,04E+09
hsa-miR-590-5p	ErbB signaling pathway	52	89	7481	19747	6,55E+09	0.012187084222578
hsa-miR-16-5p	Ether lipid metabolism	26	36	7756	19747	6,22E+09	0.0100145596455349
hsa-miR-16-5p	Fc epsilon RI signaling pathway	49	82	7756	19747	0.000136770571000737	0.0214729796471157
hsa-miR-16-5p	Gap junction	56	90	7756	19747	8,72E+08	0.00148233104920495
hsa-miR-195-5p	Gap junction	57	90	8372	19747	4,88E+09	0.00803529920081946
hsa-miR-16-5p	Glioma	45	65	7756	19747	9,49E+07	0.000166144113465711
hsa-miR-195-5p	Glioma	48	65	8372	19747	2,61E+07	4,70E+09
hsa-miR-25-3p	Glioma	52	65	8526	19747	1,32E+05	2,55E+07
hsa-miR-376a-3p	Glioma	38	65	5595	19747	3,60E+07	6,81E+09
hsa-miR-16-5p	GnRH signaling pathway	67	105	7756	19747	3,07E+07	5,49E+09
hsa-miR-195-5p	GnRH signaling pathway	67	105	8372	19747	7,60E+08	0.00130042052909104
hsa-miR-16-5p	Hedgehog signaling pathway	40	56	7756	19747	1,06E+08	0.000186005291409584
hsa-miR-195-5p	Hedgehog signaling pathway	38	56	8372	19747	0.000103937992111909	0.0162143267694578
hsa-miR-195-5p	Inositol phosphate metabolism	38	54	8372	19747	2,95E+09	0.00489422274188577
hsa-miR-16-5p	Insulin signaling pathway	89	139	7756	19747	2,90E+05	5,51E+07
hsa-miR-195-5p	Insulin signaling pathway	97	139	8372	19747	5,47E+03	1,04E+06
hsa-miR-19a-3p	Insulin signaling pathway	67	139	6553	19747	0.000164832550399785	0.0281863661183632
hsa-miR-25-3p	Insulin signaling pathway	88	139	8526	19747	1,29E+08	0.000238220057873534
hsa-miR-376a-3p	Insulin signaling pathway	70	139	5595	19747	3,19E+06	6,16E+07
hsa-miR-590-5p	Insulin signaling pathway	74	139	7481	19747	0.000159451307779625	0.0290201380158917
hsa-miR-590-5p	Leukocyte transendothelial migration	63	116	7481	19747	0.000229095003774531	0.0411376912780356
hsa-miR-16-5p	Long term depression	49	73	7756	19747	1,33E+08	0.000231688587233329
hsa-miR-195-5p	Long term depression	46	73	8372	19747	0.000301324321661288	0.0454999725708544
hsa-miR-16-5p	Long term potentiation	50	71	7756	19747	1,01E+07	1,85E+09
hsa-miR-195-5p	Long term potentiation	54	71	8372	19747	7,99E+05	1,51E+08
hsa-miR-25-3p	Long term potentiation	48	71	8526	19747	2,75E+09	0.00484572821541164
hsa-miR-376a-3p	Long term potentiation	36	71	5595	19747	5,57E+09	0.00997769712958558
hsa-miR-16-5p	MAPK signaling pathway	152	272	7756	19747	1,84E+06	3,42E+08
hsa-miR-195-5p	MAPK signaling pathway	154	272	8372	19747	1,41E+08	0.000247333538414114
hsa-miR-19a-3p	MAPK signaling pathway	125	272	6553	19747	7,19E+08	0.00135901424277507
hsa-miR-25-3p	MAPK signaling pathway	155	272	8526	19747	2,79E+08	0.000508264953770591
hsa-miR-376a-3p	MAPK signaling pathway	111	272	5595	19747	5,84E+08	0.00106856635898644
hsa-miR-16-5p	Melanogenesis	66	102	7756	19747	1,74E+07	3,14E+08
hsa-miR-195-5p	Melanogenesis	69	102	8372	19747	2,20E+07	3,95E+09
hsa-miR-16-5p	Melanoma	43	71	7756	19747	0.000226711921617821	0.0346869240075266
hsa-miR-195-5p	Melanoma	45	71	8372	19747	0.000288476174127238	0.0435599022932129
hsa-miR-25-3p	Melanoma	48	71	8526	19747	2,75E+09	0.00484572821541164
hsa-miR-376a-3p	Melanoma	37	71	5595	19747	2,02E+09	0.00362941335807191
hsa-miR-16-5p	mTOR signaling pathway	35	53	7756	19747	7,23E+08	0.0115620965596043
hsa-miR-195-5p	mTOR signaling pathway	42	53	8372	19747	4,64E+06	8,62E+08
hsa-miR-16-5p	Neurotrophin signaling pathway	77	129	7756	19747	2,07E+08	0.000358742367655563
hsa-miR-195-5p	Neurotrophin signaling pathway	87	129	8372	19747	7,56E+05	1,43E+08
hsa-miR-19a-3p	Neurotrophin signaling pathway	63	129	6553	19747	0.000159660008236973	0.0273018614085223
hsa-miR-25-3p	Neurotrophin signaling pathway	82	129	8526	19747	2,32E+08	0.000424674493567217
hsa-miR-376a-3p	Neurotrophin signaling pathway	62	129	5595	19747	1,52E+08	0.000283691841335365
hsa-miR-590-5p	Neurotrophin signaling pathway	69	129	7481	19747	0.000217416626533721	0.0391349927760697
hsa-miR-16-5p	Non small cell lung cancer	38	54	7756	19747	3,56E+08	0.000612701812897092
hsa-miR-195-5p	Non small cell lung cancer	38	54	8372	19747	2,95E+09	0.00489422274188577
hsa-miR-25-3p	Non small cell lung cancer	40	54	8526	19747	3,96E+08	0.000717360961876676
hsa-miR-376a-3p	Non small cell lung cancer	30	54	5595	19747	2,43E+09	0.00437091845948433
hsa-miR-16-5p	p53 signaling pathway	42	68	7756	19747	0.000143677426946433	0.02255735603059
hsa-miR-195-5p	p53 signaling pathway	44	68	8372	19747	0.000168528131394991	0.0257848041034336
hsa-miR-16-5p	Pancreatic cancer	51	75	7756	19747	4,38E+07	7,79E+09
hsa-miR-195-5p	Pancreatic cancer	53	75	8372	19747	6,63E+07	0.000117955194013138
hsa-miR-19a-3p	Pancreatic cancer	42	75	6553	19747	3,93E+09	0.00723930035156986
hsa-miR-25-3p	Pancreatic cancer	53	75	8526	19747	1,31E+08	0.000241126704432296
hsa-miR-376a-3p	Pancreatic cancer	39	75	5595	19747	1,30E+09	0.0023375462184097
hsa-miR-16-5p	Pathways in cancer	193	330	7756	19747	9,44E+01	1,82E+04
hsa-miR-195-5p	Pathways in cancer	199	330	8372	19747	3,20E+03	6,14E+05

hsa-miR-19a-3p	Pathways in cancer	152	330	6553	19747	6,64E+07	0.000127448995016266
hsa-miR-25-3p	Pathways in cancer	192	330	8526	19747	2,36E+05	4,56E+08
hsa-miR-376a-3p	Pathways in cancer	146	330	5595	19747	3,85E+04	7,48E+06
hsa-miR-590-5p	Pathways in cancer	173	330	7481	19747	4,46E+06	8,65E+08
hsa-miR-16-5p	Phosphatidylinositol signaling system	46	76	7756	19747	0.000141686455895795	0.0222447735756398
hsa-miR-195-5p	Phosphatidylinositol signaling system	52	76	8372	19747	3,99E+08	0.000685681829752934
hsa-miR-16-5p	Prostate cancer	61	89	7756	19747	2,09E+06	3,88E+08
hsa-miR-195-5p	Prostate cancer	61	89	8372	19747	5,42E+07	9,64E+09
hsa-miR-19a-3p	Prostate cancer	46	89	6553	19747	0.000232851130463537	0.0395846921788013
hsa-miR-25-3p	Prostate cancer	56	89	8526	19747	0.000134012943030935	0.0227822003152589
hsa-miR-195-5p	Regulation of actin cytoskeleton	118	212	8372	19747	6,35E+09	0.0102239269613309
hsa-miR-376a-3p	Renal cell carcinoma	39	71	5595	19747	2,24E+08	0.000419682162660338
hsa-miR-16-5p	Small cell lung cancer	49	84	7756	19747	0.000308981219150898	0.0466561640917856
hsa-miR-25-3p	Small cell lung cancer	53	84	8526	19747	0.000181068776332986	0.0306006232002746
hsa-miR-19a-3p	SNARE interactions in vesicular transport	24	39	6553	19747	0.000261096411396454	0.0441252935260008
hsa-miR-16-5p	T cell receptor signaling pathway	72	110	7756	19747	2,38E+06	4,43E+08
hsa-miR-195-5p	T cell receptor signaling pathway	67	110	8372	19747	6,77E+09	0.0108281975924561
hsa-miR-19a-3p	T cell receptor signaling pathway	56	110	6553	19747	8,87E+09	0.0155275849165313
hsa-miR-25-3p	T cell receptor signaling pathway	72	110	8526	19747	1,95E+08	0.000356828847245027
hsa-miR-19a-3p	Ubiquitin mediated proteolysis	70	134	6553	19747	3,92E+08	0.00074049219252587
hsa-miR-16-5p	Vascular smooth muscle contraction	65	116	7756	19747	0.000183617172614241	0.0282770445825931
hsa-miR-195-5p	Vascular smooth muscle contraction	73	116	8372	19747	6,34E+08	0.00109050835134864
hsa-miR-16-5p	VEGF signaling pathway	52	78	7756	19747	8,79E+07	0.000154727466685428
hsa-miR-195-5p	VEGF signaling pathway	52	78	8372	19747	1,28E+09	0.00215682893474645
hsa-miR-25-3p	VEGF signaling pathway	50	78	8526	19747	0.000154110755637263	0.0261988284583348
hsa-miR-16-5p	Wnt signaling pathway	95	152	7756	19747	5,47E+05	1,03E+08
hsa-miR-195-5p	Wnt signaling pathway	104	152	8372	19747	7,23E+03	1,38E+06
hsa-miR-19a-3p	Wnt signaling pathway	83	152	6553	19747	4,10E+06	7,90E+08
hsa-miR-25-3p	Wnt signaling pathway	90	152	8526	19747	4,77E+09	0.00829783026781591
hsa-miR-376a-3p	Wnt signaling pathway	72	152	5595	19747	4,59E+07	8,67E+09
hsa-miR-590-5p	Wnt signaling pathway	85	152	7481	19747	4,52E+08	0.00086828893664623

Cluster A: 18 miRNAs consistently deregulated in AbP or recent T1D groups (13 of them being also deregulated in T1D 2-5y)
