# Supplementary material for: Progression of Type 1 Diabetes: Circulating MicroRNA Expression Profiles Changes from Preclinical to Overt Disease
Source: J Immunol Res. 2022 Jul 19;2022:2734490. doi: 10.1155/2022/2734490 (PMC9325579; doi:10.1155/2022/2734490)
Supplement: Supplementary Materials — Supplementary Table 1S: miRNAs without expression in serum samples. Supplementary Table 2S: pathways related to up-and downregulated miRNAs of cluster A predicted by the miRWalk platform. Supplementary Table 3S: pathways related to upregulated miRNAs of cluster B predicted by the miRWalk platform. Supplementary Table 4S: pathways related to downregulated miRNAs of cluster B predicted by the miRWalk platform. Supplementary Table 5S: most frequent target genes of miRNAs from cluster A of TargetScan. Supplementary Table 6S: most frequent target genes of miRNAs from cluster B by TargetScan. Supplementary Table 7S: ingenuity canonical pathways related to differentially expressed miRNAs' targets. Supplementary Table 8S: reporting guidelines: STREGA. [file 2734490.f1.zip › Suppl 3 miRWalk pathway Cluster B up-regulated miRNAs.pdf]

Suppementary Table 3S: **Pathways related to up-regulated miRNAs of Cluster B predicted at miRWalk platform**

miRNA	PathName	PathFg	PathBg	GenomeFG	GenomeBG	Fischer exact test	BH false discovery rate
hsa-miR-130b-3p	Acute myeloid leukemia	37	58	7949	19747	0.000244819176540473	0.0384366107168543
hsa-miR-132-3p	Acute myeloid leukemia	33	58	6440	19747	0.000117860867600675	0.0202720692273161
hsa-miR-491-5p	Acute myeloid leukemia	45	58	10672	19747	0.000174298922710136	0.0289336211698827
hsa-miR-324-3p	Acute myeloid leukemia	41	58	8351	19747	1,12E+09	0.00199812198332241
hsa-miR-328-3p	Acute myeloid leukemia	40	58	7326	19747	8,21E+06	0.000147691180541331
hsa-miR-335-5p	Acute myeloid leukemia	33	58	5890	19747	1,60E+09	0.00276003682177895
hsa-miR-486-3p	Acute myeloid leukemia	50	58	11653	19747	6,70E+08	0.00108504640476253
hsa-let-7c-5p	Adherens junction	59	76	10808	19747	2,81E+09	0.00499425859569112
hsa-miR-106b-5p	Adherens junction	52	76	8499	19747	6,76E+08	0.00122328682278677
hsa-miR-130b-3p	Adherens junction	53	76	7949	19747	1,83E+07	3,38E+09
hsa-miR-132-3p	Adherens junction	40	76	6440	19747	0.000237544531801563	0.0399074813426626
hsa-miR-145-5p	Adherens junction	57	76	9602	19747	2,38E+08	0.000439686530597515
hsa-miR-148a-3p	Adherens junction	49	76	6669	19747	4,28E+06	8,14E+08
hsa-miR-18a-5p	Adherens junction	51	76	8742	19747	4,84E+09	0.00885929200024005
hsa-miR-346	Adherens junction	48	76	8052	19747	6,63E+09	0.0117419208205449
hsa-miR-491-5p	Adherens junction	62	76	10672	19747	4,45E+07	8,36E+09
hsa-miR-708-5p	Adherens junction	56	76	9879	19747	2,16E+09	0.00412160296869877
hsa-miR-181c-5p	Adherens junction	50	76	8188	19747	1,57E+09	0.00291762348094325
hsa-miR-193a-5p	Adherens junction	63	76	11509	19747	3,98E+08	0.000729214916580499
hsa-miR-212-3p	Adherens junction	47	76	6789	19747	9,04E+07	0.000168149505526547
hsa-miR-215-5p	Adherens junction	33	76	4762	19747	0.000165744543327628	0.0311599741455941
hsa-miR-221-3p	Adherens junction	55	76	9076	19747	2,66E+08	0.000508536302372289
hsa-miR-27a-3p	Adherens junction	57	76	9729	19747	4,01E+08	0.000761394155305357
hsa-miR-410-3p	Adherens junction	46	76	7692	19747	0.000112475673956276	0.0208079996819111
hsa-miR-423-5p	Adherens junction	63	76	12169	19747	4,81E+09	0.00812995859327178
hsa-miR-485-3p	Adherens junction	45	76	6269	19747	7,14E+07	0.000135701328253421
hsa-miR-486-3p	Adherens junction	62	76	11653	19747	2,29E+09	0.00358984184783953
hsa-miR-489-3p	Adherens junction	43	76	6768	19747	5,46E+09	0.0102176474189569
hsa-miR-532-3p	Adherens junction	53	76	8784	19747	7,35E+08	0.0012869265216549
hsa-miR-532-5p	Adherens junction	47	76	8143	19747	0.000226989041475521	0.0381341589678876
hsa-miR-636	Adherens junction	54	76	8717	19747	1,82E+08	0.000346447742282912
hsa-miR-92a-3p	Adherens junction	47	76	8069	19747	0.000175765110595711	0.0300558339118666
hsa-miR-130b-3p	Adipocytokine signaling pathway	46	70	7949	19747	1,45E+09	0.00250000532142752
hsa-miR-18a-5p	Adipocytokine signaling pathway	46	70	8742	19747	0.000238472824679277	0.0426866356175906

hsa-miR-491-5p	Adipocytokine signaling pathway	53	70	10672	19747	0.000148750889145047	0.0249152246716898
hsa-miR-181c-5p	Adipocytokine signaling pathway	46	70	8188	19747	3,55E+09	0.00653844459709216
hsa-miR-221-3p	Adipocytokine signaling pathway	48	70	9076	19747	0.000108786905923643	0.019472856160332
hsa-miR-324-3p	Adipocytokine signaling pathway	46	70	8351	19747	6,38E+09	0.0108533509929681
hsa-miR-335-5p	Adipocytokine signaling pathway	37	70	5890	19747	4,62E+09	0.00780507611010947
hsa-miR-410-3p	Adipocytokine signaling pathway	42	70	7692	19747	0.000288482627774357	0.0507729424882869
hsa-miR-486-3p	Adipocytokine signaling pathway	58	70	11653	19747	1,68E+09	0.00264908350062362
hsa-miR-532-3p	Adipocytokine signaling pathway	46	70	8784	19747	0.000272785599402796	0.0441912671032529
hsa-miR-660-5p	Adipocytokine signaling pathway	38	70	5834	19747	1,30E+09	0.00233117915049232
hsa-miR-92a-3p	Adipocytokine signaling pathway	44	70	8069	19747	0.0001641955507087	0.0282416347218964
hsa-miR-132-3p	Aldosterone regulated sodium reabsorption	26	42	6440	19747	9,12E+09	0.015782563051612
hsa-miR-491-5p	Aldosterone regulated sodium reabsorption	36	42	10672	19747	1,42E+09	0.00255906326516625
hsa-miR-335-5p	Aldosterone regulated sodium reabsorption	25	42	5890	19747	6,09E+09	0.0102229063623819
hsa-miR-486-3p	Aldosterone regulated sodium reabsorption	37	42	11653	19747	3,96E+09	0.00617674006071216
hsa-miR-652-3p	Aldosterone regulated sodium reabsorption	26	42	6694	19747	0.000187638741148173	0.0343378896301156
hsa-miR-660-5p	Aldosterone regulated sodium reabsorption	25	42	5834	19747	5,11E+09	0.00884973910583337
hsa-miR-92a-3p	Amyotrophic lateral sclerosis ALS	36	55	8069	19747	0.00019688311587883	0.03366701281528
hsa-let-7c-5p	Apoptosis	66	87	10808	19747	3,62E+08	0.00640059309564595
hsa-miR-106b-5p	Apoptosis	58	87	8499	19747	7,21E+07	0.00130449612982968
hsa-miR-122-5p	Apoptosis	66	87	10891	19747	4,98E+09	0.00911092116547369
hsa-miR-132-3p	Apoptosis	47	87	6440	19747	2,96E+09	0.00521534339132987
hsa-miR-208a-3p	Apoptosis	35	87	4498	19747	0.000197627892186247	0.0341896253482208
hsa-miR-212-3p	Apoptosis	51	87	6789	19747	3,13E+08	0.000569799419240842
hsa-miR-214-3p	Apoptosis	61	87	10069	19747	0.000219431197826711	0.0362061476414073
hsa-miR-27a-3p	Apoptosis	63	87	9729	19747	9,20E+08	0.00172074661605946

hsa-miR-29c-3p	Apoptosis	47	87	6309	19747	1,63E+09	0.00286859202296747
hsa-miR-324-3p	Apoptosis	53	87	8351	19747	0.000346585052801699	0.0554536084482718
hsa-miR-328-3p	Apoptosis	51	87	7326	19747	3,59E+09	0.0060987636569973
hsa-miR-335-5p	Apoptosis	42	87	5890	19747	0.000219267151396974	0.0350827442235158
hsa-miR-365a-3p	Apoptosis	39	87	4746	19747	1,67E+08	0.0029950169554589
hsa-miR-410-3p	Apoptosis	51	87	7692	19747	0.000155854080900768	0.0285212968048406
hsa-miR-486-3p	Apoptosis	75	87	11653	19747	3,19E+06	5,78E+08
hsa-miR-532-3p	Apoptosis	59	87	8784	19747	9,18E+08	0.00160608547858007
hsa-miR-532-5p	Apoptosis	58	87	8143	19747	1,42E+08	0.000269988107409157
hsa-miR-636	Apoptosis	59	87	8717	19747	6,87E+08	0.0012911888303255
hsa-miR-660-5p	Apoptosis	49	87	5834	19747	1,69E+07	3,19E+09
hsa-miR-92a-3p	Apoptosis	58	87	8069	19747	9,97E+07	0.000189445262606298
hsa-miR-145-5p	Arrhythmogenic right ventricular cardiomyopathy ARVC	54	74	9602	19747	1,73E+09	0.00305734707548562
hsa-miR-193a-5p	Arrhythmogenic right ventricular cardiomyopathy ARVC	58	74	11509	19747	0.000218723538143536	0.0367455544081141
hsa-let-7c-5p	Axon guidance	100	129	10808	19747	5,81E+06	1,12E+09
hsa-miR-106b-5p	Axon guidance	94	129	8499	19747	5,59E+02	1,08E+05
hsa-miR-122-5p	Axon guidance	102	129	10891	19747	1,09E+06	2,10E+08
hsa-miR-125b-5p	Axon guidance	78	129	7799	19747	1,12E+08	0.000212304391550373
hsa-miR-130b-3p	Axon guidance	74	129	7949	19747	6,14E+09	0.00995290085509444
hsa-miR-132-3p	Axon guidance	68	129	6440	19747	1,79E+08	0.000335976957843441
hsa-miR-145-5p	Axon guidance	97	129	9602	19747	5,86E+04	1,13E+06
hsa-miR-148a-3p	Axon guidance	70	129	6669	19747	1,30E+08	0.000241145820353463
hsa-miR-18a-5p	Axon guidance	85	129	8742	19747	5,69E+07	0.000108031226473923
hsa-miR-346	Axon guidance	87	129	8052	19747	7,78E+04	1,50E+07
hsa-miR-491-5p	Axon guidance	104	129	10672	19747	2,21E+03	4,25E+06
hsa-miR-708-5p	Axon guidance	91	129	9879	19747	1,66E+08	0.000319058904067476
hsa-miR-181c-5p	Axon guidance	79	129	8188	19747	4,42E+08	0.000835783845070745
hsa-miR-193a-5p	Axon guidance	106	129	11509	19747	5,59E+05	1,08E+08
hsa-miR-208a-3p	Axon guidance	51	129	4498	19747	1,40E+09	0.00257492465959075
hsa-miR-212-3p	Axon guidance	68	129	6789	19747	1,38E+09	0.00247647692847044
hsa-miR-214-3p	Axon guidance	93	129	10069	19747	7,77E+07	0.000143774788205373
hsa-miR-21-5p	Axon guidance	77	129	7952	19747	6,24E+08	0.00116136576064068
hsa-miR-221-3p	Axon guidance	87	129	9076	19747	6,49E+07	0.000124666483122391
hsa-miR-27a-3p	Axon guidance	98	129	9729	19747	4,41E+04	8,51E+06

hsa-miR-29c-3p	Axon guidance	70	129	6309	19747	1,24E+07	2,32E+09
hsa-miR-324-3p	Axon guidance	89	129	8351	19747	7,36E+04	1,42E+06
hsa-miR-328-3p	Axon guidance	74	129	7326	19747	2,18E+08	0.000386411002875886
hsa-miR-365a-3p	Axon guidance	53	129	4746	19747	1,32E+09	0.00238655668008603
hsa-miR-423-5p	Axon guidance	112	129	12169	19747	1,99E+04	3,85E+06
hsa-miR-485-3p	Axon guidance	69	129	6269	19747	2,42E+06	4,64E+09
sa-miR-486-3p	Axon guidance	114	129	11653	19747	1,88E+00	3,65E+02
hsa-miR-489-3p	Axon guidance	65	129	6768	19747	0.000114094210286749	0.0209933346927618
hsa-miR-532-3p	Axon guidance	78	129	8784	19747	0.000182013195268937	0.0296681508288367
hsa-miR-636	Axon guidance	89	129	8717	19747	9,57E+05	1,86E+08
hsa-miR-652-3p	Axon guidance	67	129	6694	19747	1,78E+09	0.0033790324644466
hsa-miR-660-5p	Axon guidance	59	129	5834	19747	7,01E+09	0.0120629994736805
hsa-miR-92a-3p	Axon guidance	77	129	8069	19747	1,17E+09	0.00217918780691389
hsa-let-7c-5p	B cell receptor signaling pathway	57	75	10808	19747	0.000111635872230908	0.0190897341514853
hsa-miR-132-3p	B cell receptor signaling pathway	41	75	6440	19747	6,52E+09	0.0113516536651965
hsa-miR-491-5p	B cell receptor signaling pathway	56	75	10672	19747	0.000186466900673037	0.0307670386110512
hsa-miR-193a-5p	B cell receptor signaling pathway	64	75	11509	19747	4,00E+07	7,53E+09
hsa-miR-29c-3p	B cell receptor signaling pathway	39	75	6309	19747	0.000243847436125654	0.0412102167052356
hsa-miR-324-3p	B cell receptor signaling pathway	47	75	8351	19747	0.000292487310562415	0.0467979696899864
hsa-miR-328-3p	B cell receptor signaling pathway	45	75	7326	19747	4,60E+09	0.0077814508765141
hsa-miR-365a-3p	B cell receptor signaling pathway	33	75	4746	19747	0.000113576850024222	0.0194216413541419
hsa-miR-423-5p	B cell receptor signaling pathway	64	75	12169	19747	5,97E+08	0.00105725539142632
hsa-miR-486-3p	B cell receptor signaling pathway	66	75	11653	19747	3,66E+06	6,63E+08
hsa-miR-532-5p	B cell receptor signaling pathway	49	75	8143	19747	2,12E+09	0.00378812238732683
hsa-miR-92a-3p	B cell receptor signaling pathway	46	75	8069	19747	0.000270157346302029	0.0459267488713449
hsa-miR-145-5p	Basal cell carcinoma	40	55	9602	19747	0.000239208760278346	0.0397086542062055
hsa-miR-423-5p	Basal cell carcinoma	47	55	12169	19747	9,92E+09	0.0164649609806437
hsa-miR-532-3p	Basal cell carcinoma	39	55	8784	19747	6,53E+09	0.0108417785570601
hsa-miR-328-3p	Bladder cancer	28	43	7326	19747	0.000176513040326892	0.0291246516539372

hsa-let-7c-5p	Calcium signaling pathway	125	178	10808	19747	1,55E+08	0.0027994495842719
hsa-miR-106b-5p	Calcium signaling pathway	104	178	8499	19747	2,41E+09	0.00428474660038043
hsa-miR-122-5p	Calcium signaling pathway	127	178	10891	19747	6,13E+08	0.00116518464404106
hsa-miR-130b-3p	Calcium signaling pathway	96	178	7949	19747	0.0001459102099851	0.0230538131776458
hsa-miR-145-5p	Calcium signaling pathway	114	178	9602	19747	2,27E+09	0.00401361372330948
hsa-miR-148a-3p	Calcium signaling pathway	85	178	6669	19747	7,42E+09	0.0126878003884893
hsa-miR-346	Calcium signaling pathway	100	178	8052	19747	2,24E+09	0.00404560161993861
hsa-miR-491-5p	Calcium signaling pathway	122	178	10672	19747	5,30E+09	0.00916789300651792
hsa-miR-193a-5p	Calcium signaling pathway	131	178	11509	19747	1,41E+09	0.00253150845022894
hsa-miR-214-3p	Calcium signaling pathway	120	178	10069	19747	6,09E+08	0.00110252979803466
hsa-miR-21-5p	Calcium signaling pathway	96	178	7952	19747	0.00014830258852343	0.0268427685227409
hsa-miR-27a-3p	Calcium signaling pathway	115	178	9729	19747	2,45E+09	0.00451250708656505
hsa-miR-324-3p	Calcium signaling pathway	105	178	8351	19747	4,92E+08	0.000885677401261265
hsa-miR-328-3p	Calcium signaling pathway	91	178	7326	19747	8,84E+09	0.014827095387459
hsa-miR-335-5p	Calcium signaling pathway	77	178	5890	19747	9,34E+09	0.0154189864970639
hsa-miR-365a-3p	Calcium signaling pathway	71	178	4746	19747	1,83E+08	0.000343408730729688
hsa-miR-423-5p	Calcium signaling pathway	133	178	12169	19747	0.000144429212710244	0.0235419616717698
hsa-miR-485-3p	Calcium signaling pathway	83	178	6269	19747	2,21E+09	0.00406929998199512
hsa-miR-486-3p	Calcium signaling pathway	136	178	11653	19747	7,31E+07	0.000127178724815086
hsa-miR-532-3p	Calcium signaling pathway	110	178	8784	19747	2,31E+08	0.000408404593124646
hsa-miR-532-5p	Calcium signaling pathway	97	178	8143	19747	0.000230909600519875	0.0387928128873389
hsa-miR-636	Calcium signaling pathway	112	178	8717	19747	3,21E+07	6,17E+09

hsa-miR-652-3p	Calcium signaling pathway	87	178	6694	19747	2,44E+09	0.00463032363659854
hsa-miR-660-5p	Calcium signaling pathway	77	178	5834	19747	6,61E+09	0.0113673914717074
hsa-miR-92a-3p	Calcium signaling pathway	97	178	8069	19747	0.000155902314958664	0.0268151981728901
hsa-let-7c-5p	Cell adhesion molecules CAMs	95	133	10808	19747	5,52E+09	0.0096556479124811
hsa-miR-125b-5p	Cell adhesion molecules CAMs	80	133	7799	19747	1,11E+08	0.000209600620519741
hsa-miR-130b-3p	Cell adhesion molecules CAMs	78	133	7949	19747	1,33E+09	0.00231973901828869
hsa-miR-145-5p	Cell adhesion molecules CAMs	86	133	9602	19747	0.000134241288172684	0.0226867777011835
hsa-miR-148a-3p	Cell adhesion molecules CAMs	74	133	6669	19747	1,74E+07	3,29E+08
hsa-miR-491-5p	Cell adhesion molecules CAMs	92	133	10672	19747	0.000254606077533117	0.0414406287906677
hsa-miR-708-5p	Cell adhesion molecules CAMs	89	133	9879	19747	5,78E+09	0.0108579806041837
hsa-miR-193a-5p	Cell adhesion molecules CAMs	99	133	11509	19747	7,21E+09	0.0126091066234996
hsa-miR-423-5p	Cell adhesion molecules CAMs	102	133	12169	19747	0.00015453166295118	0.0250341293980912
hsa-miR-486-3p	Cell adhesion molecules CAMs	104	133	11653	19747	2,19E+08	0.00037222623167571
hsa-miR-491-5p	Chemokine signaling pathway	130	189	10672	19747	2,34E+09	0.00417265321076891
hsa-miR-335-5p	Chemokine signaling pathway	81	189	5890	19747	9,17E+09	0.0152250218617758
hsa-miR-486-3p	Chemokine signaling pathway	147	189	11653	19747	3,51E+06	6,36E+08
hsa-miR-532-5p	Chemokine signaling pathway	103	189	8143	19747	0.000150303675435386	0.0255516248240157
hsa-let-7c-5p	Chronic myeloid leukemia	59	75	10808	19747	1,35E+09	0.00243527558256295
hsa-miR-106b-5p	Chronic myeloid leukemia	52	75	8499	19747	3,67E+08	0.000670875118192454
hsa-miR-122-5p	Chronic myeloid leukemia	58	75	10891	19747	5,39E+09	0.00986944900855594
hsa-miR-125b-5p	Chronic myeloid leukemia	46	75	7799	19747	0.000105276981529105	0.0182129178045352
hsa-miR-130b-3p	Chronic myeloid leukemia	57	75	7949	19747	3,13E+04	6,01E+06

hsa-miR-132-3p	Chronic myeloid leukemia	47	75	6440	19747	8,76E+05	1,68E+09
hsa-miR-145-5p	Chronic myeloid leukemia	55	75	9602	19747	1,13E+09	0.00202905145651873
hsa-miR-148a-3p	Chronic myeloid leukemia	48	75	6669	19747	8,27E+06	1,57E+08
hsa-miR-18a-5p	Chronic myeloid leukemia	53	75	8742	19747	3,29E+08	0.000618848276988812
hsa-miR-491-5p	Chronic myeloid leukemia	57	75	10672	19747	7,00E+09	0.0120467968377094
hsa-miR-181c-5p	Chronic myeloid leukemia	50	75	8188	19747	8,97E+08	0.00167757853522381
hsa-miR-193a-5p	Chronic myeloid leukemia	61	75	11509	19747	1,88E+09	0.00337102842432011
hsa-miR-208a-3p	Chronic myeloid leukemia	37	75	4498	19747	4,20E+07	7,94E+09
hsa-miR-212-3p	Chronic myeloid leukemia	45	75	6789	19747	4,97E+08	0.00089867507037469
hsa-miR-214-3p	Chronic myeloid leukemia	56	75	10069	19747	2,27E+09	0.00403831044354454
hsa-miR-21-5p	Chronic myeloid leukemia	50	75	7952	19747	3,35E+08	0.000633599244625422
hsa-miR-29c-3p	Chronic myeloid leukemia	44	75	6309	19747	1,64E+07	0.000297730146543248
hsa-miR-324-3p	Chronic myeloid leukemia	55	75	8351	19747	4,75E+06	9,10E+08
hsa-miR-328-3p	Chronic myeloid leukemia	56	75	7326	19747	3,74E+03	7,18E+05
hsa-miR-335-5p	Chronic myeloid leukemia	43	75	5890	19747	6,51E+06	0.000120364547889923
hsa-miR-365a-3p	Chronic myeloid leukemia	33	75	4746	19747	0.000113576850024222	0.0194216413541419
hsa-miR-410-3p	Chronic myeloid leukemia	47	75	7692	19747	2,70E+09	0.00510817879394599
hsa-miR-423-5p	Chronic myeloid leukemia	63	75	12169	19747	2,07E+09	0.00353715938834847
hsa-miR-485-3p	Chronic myeloid leukemia	40	75	6269	19747	8,49E+09	0.0152810773584856
hsa-miR-486-3p	Chronic myeloid leukemia	69	75	11653	19747	1,59E+04	3,05E+06
hsa-miR-532-3p	Chronic myeloid leukemia	57	75	8784	19747	2,65E+06	5,04E+07
hsa-miR-532-5p	Chronic myeloid leukemia	55	75	8143	19747	1,67E+06	3,23E+07

hsa-miR-636	Chronic myeloid leukemia	52	75	8717	19747	8,94E+08	0.00168024796174128
hsa-miR-660-5p	Chronic myeloid leukemia	47	75	5834	19747	2,75E+05	5,30E+07
hsa-miR-92a-3p	Chronic myeloid leukemia	47	75	8069	19747	0.000110754473882535	0.019271278455561
hsa-miR-99b-5p	Chronic myeloid leukemia	23	75	2577	19747	5,32E+09	0.00962405667124111
hsa-let-7c-5p	Colorectal cancer	66	86	10808	19747	1,87E+09	0.0033730454040937
hsa-miR-106b-5p	Colorectal cancer	60	86	8499	19747	4,74E+07	9,05E+09
hsa-miR-122-5p	Colorectal cancer	64	86	10891	19747	0.000173218002518379	0.0311792404533082
hsa-miR-125b-5p	Colorectal cancer	53	86	7799	19747	2,67E+09	0.0048068313041558
hsa-miR-130b-3p	Colorectal cancer	62	86	7949	19747	2,07E+05	3,93E+07
hsa-miR-132-3p	Colorectal cancer	50	86	6440	19747	9,58E+07	0.000181996436655332
hsa-miR-145-5p	Colorectal cancer	64	86	9602	19747	9,26E+07	0.000172185971813093
hsa-miR-148a-3p	Colorectal cancer	47	86	6669	19747	5,40E+08	0.00929359099484989
hsa-miR-18a-5p	Colorectal cancer	55	86	8742	19747	0.000181019439417763	0.0325834990951973
hsa-miR-346	Colorectal cancer	55	86	8052	19747	1,17E+08	0.00215300448091028
hsa-miR-491-5p	Colorectal cancer	66	86	10672	19747	1,08E+09	0.00195705281226272
hsa-miR-708-5p	Colorectal cancer	60	86	9879	19747	0.000156938983832306	0.0288767730251444
hsa-miR-193a-5p	Colorectal cancer	66	86	11509	19747	0.000253957211512395	0.0424108543225699
hsa-miR-208a-3p	Colorectal cancer	40	86	4498	19747	1,03E+08	0.000193820639470488
hsa-miR-212-3p	Colorectal cancer	51	86	6789	19747	1,94E+07	0.000355407516155259
hsa-miR-214-3p	Colorectal cancer	63	86	10069	19747	2,00E+09	0.00356632077495945
hsa-miR-215-5p	Colorectal cancer	36	86	4762	19747	0.000209323832448427	0.0393528805003043
hsa-miR-21-5p	Colorectal cancer	57	86	7952	19747	9,41E+07	0.000179819700381897
hsa-miR-29c-3p	Colorectal cancer	51	86	6309	19747	1,55E+07	2,89E+09
hsa-miR-324-3p	Colorectal cancer	58	86	8351	19747	2,11E+08	0.000386325159148691
hsa-miR-328-3p	Colorectal cancer	59	86	7326	19747	2,87E+05	5,49E+07
hsa-miR-335-5p	Colorectal cancer	51	86	5890	19747	1,29E+06	2,46E+08
hsa-miR-365a-3p	Colorectal cancer	37	86	4746	19747	8,05E+09	0.0139259800782651
hsa-miR-410-3p	Colorectal cancer	51	86	7692	19747	0.000103415840960185	0.0191319305776343
hsa-miR-423-5p	Colorectal cancer	72	86	12169	19747	6,80E+08	0.00119666299465989
hsa-miR-485-3p	Colorectal cancer	44	86	6269	19747	0.000138990679944313	0.0247403410300878
hsa-miR-486-3p	Colorectal cancer	71	86	11653	19747	2,48E+07	0.000419667164349283
hsa-miR-532-3p	Colorectal cancer	67	86	8784	19747	2,41E+04	4,64E+06
hsa-miR-532-5p	Colorectal cancer	55	86	8143	19747	1,73E+09	0.00310648390582958
hsa-miR-92a-3p	Colorectal cancer	56	86	8069	19747	4,66E+08	0.000872110507128782
hsa-miR-99b-5p	Colorectal cancer	29	86	2577	19747	6,82E+06	0.000125507401004477

hsa-miR-486-3p	Dilated cardiomyopathy	74	94	11653	19747	4,16E+09	0.00648187031939425
hsa-miR-660-5p	Dilated cardiomyopathy	45	94	5834	19747	0.000133217222808346	0.0222472762089938
hsa-let-7c-5p	Endocytosis	133	187	10808	19747	2,87E+08	0.000534186690675423
hsa-miR-122-5p	Endocytosis	146	187	10891	19747	4,45E+03	8,63E+05
hsa-miR-125b-5p	Endocytosis	104	187	7799	19747	5,49E+08	0.00102075940974808
hsa-miR-130b-3p	Endocytosis	113	187	7949	19747	1,81E+06	3,42E+07
hsa-miR-145-5p	Endocytosis	125	187	9602	19747	3,32E+07	6,24E+09
hsa-miR-18a-5p	Endocytosis	114	187	8742	19747	2,97E+08	0.000558857323422618
hsa-miR-346	Endocytosis	111	187	8052	19747	2,04E+07	3,90E+09
hsa-miR-491-5p	Endocytosis	146	187	10672	19747	6,00E+02	1,16E+04
hsa-miR-708-5p	Endocytosis	129	187	9879	19747	1,02E+07	1,96E+09
hsa-miR-181c-5p	Endocytosis	107	187	8188	19747	9,35E+08	0.00174788019683886
hsa-miR-193a-5p	Endocytosis	144	187	11509	19747	5,15E+06	9,89E+08
hsa-miR-208a-3p	Endocytosis	72	187	4498	19747	8,81E+07	0.000165611182430414
hsa-miR-212-3p	Endocytosis	91	187	6789	19747	3,67E+09	0.00649635936188597
hsa-miR-214-3p	Endocytosis	129	187	10069	19747	4,01E+07	7,51E+09
hsa-miR-21-5p	Endocytosis	117	187	7952	19747	5,40E+04	1,05E+07
hsa-miR-27a-3p	Endocytosis	137	187	9729	19747	1,64E+03	3,18E+04
hsa-miR-29c-3p	Endocytosis	83	187	6309	19747	0.00023284077529695	0.0393500910251845
hsa-miR-324-3p	Endocytosis	109	187	8351	19747	7,04E+08	0.00126771223520321
hsa-miR-335-5p	Endocytosis	81	187	5890	19747	5,91E+09	0.00992726321483328
hsa-miR-365a-3p	Endocytosis	68	187	4746	19747	0.000100760004816847	0.0173307208284976
hsa-miR-423-5p	Endocytosis	155	187	12169	19747	1,79E+04	3,46E+06
hsa-miR-485-3p	Endocytosis	95	187	6269	19747	4,30E+06	8,35E+08
hsa-miR-486-3p	Endocytosis	143	187	11653	19747	3,49E+07	6,20E+09
hsa-miR-489-3p	Endocytosis	90	187	6768	19747	5,95E+09	0.0110754228907177
hsa-miR-532-3p	Endocytosis	109	187	8784	19747	9,57E+09	0.0157980239336699
hsa-miR-532-5p	Endocytosis	101	187	8143	19747	0.000269364179328732	0.0449838179478982
hsa-miR-636	Endocytosis	115	187	8717	19747	1,23E+08	0.000233301281691151
hsa-miR-652-3p	Endocytosis	93	187	6694	19747	5,29E+07	0.00102006234425966
hsa-miR-92a-3p	Endocytosis	106	187	8069	19747	8,43E+08	0.0015678518492925
hsa-let-7c-5p	Endometrial cancer	41	52	10808	19747	0.000258328505220575	0.0428223428022159
hsa-miR-130b-3p	Endometrial cancer	38	52	7949	19747	1,59E+08	0.000291483307757672
hsa-miR-132-3p	Endometrial cancer	30	52	6440	19747	0.000169446546334146	0.0288059128768048
hsa-miR-145-5p	Endometrial cancer	39	52	9602	19747	9,42E+09	0.0161089393721467
hsa-miR-193a-5p	Endometrial cancer	44	52	11509	19747	4,29E+09	0.00754686385259498
hsa-miR-208a-3p	Endometrial cancer	24	52	4498	19747	0.000168020067704888	0.0290674717129457
hsa-miR-214-3p	Endometrial cancer	40	52	10069	19747	0.000109547725243025	0.0185135655660712

hsa-miR-29c-3p	Endometrial cancer	30	52	6309	19747	0.000111746906716608	0.0193322148619731
hsa-miR-328-3p	Endometrial cancer	34	52	7326	19747	3,20E+09	0.00544179039930775
hsa-miR-335-5p	Endometrial cancer	29	52	5890	19747	8,48E+09	0.0140799951349717
hsa-miR-486-3p	Endometrial cancer	45	52	11653	19747	1,59E+09	0.00250671861327572
hsa-miR-532-3p	Endometrial cancer	39	52	8784	19747	7,50E+08	0.00131203972347544
hsa-let-7c-5p	ErbB signaling pathway	66	89	10808	19747	0.000121651948843818	0.0208024832522928
hsa-miR-106b-5p	ErbB signaling pathway	57	89	8499	19747	5,10E+09	0.00902973464012311
hsa-miR-122-5p	ErbB signaling pathway	67	89	10891	19747	6,67E+09	0.0121418238670078
hsa-miR-125b-5p	ErbB signaling pathway	55	89	7799	19747	1,69E+09	0.00307423537428417
hsa-miR-130b-3p	ErbB signaling pathway	53	89	7949	19747	0.000178782559724598	0.0282476444364865
hsa-miR-132-3p	ErbB signaling pathway	47	89	6440	19747	6,32E+09	0.0109962469654898
hsa-miR-145-5p	ErbB signaling pathway	67	89	9602	19747	2,46E+07	4,65E+09
hsa-miR-148a-3p	ErbB signaling pathway	47	89	6669	19747	0.000164095201141526	0.0269116129872103
hsa-miR-18a-5p	ErbB signaling pathway	62	89	8742	19747	1,09E+08	0.000207606736175877
hsa-miR-346	ErbB signaling pathway	57	89	8052	19747	7,65E+08	0.00141522481884576
hsa-miR-491-5p	ErbB signaling pathway	71	89	10672	19747	3,56E+07	6,70E+09
hsa-miR-181c-5p	ErbB signaling pathway	59	89	8188	19747	1,90E+08	0.00036536602567227
hsa-miR-193a-5p	ErbB signaling pathway	69	89	11509	19747	0.000105672550472159	0.0182813512316834
hsa-miR-208a-3p	ErbB signaling pathway	38	89	4498	19747	2,26E+09	0.00413808968977847
hsa-miR-212-3p	ErbB signaling pathway	49	89	6789	19747	4,93E+09	0.00872745046398471
hsa-miR-21-5p	ErbB signaling pathway	56	89	7952	19747	1,29E+09	0.00236502986864026
hsa-miR-221-3p	ErbB signaling pathway	59	89	9076	19747	8,51E+09	0.0153215506622363
hsa-miR-27a-3p	ErbB signaling pathway	61	89	9729	19747	0.000177185670994081	0.0315390494369465
hsa-miR-29c-3p	ErbB signaling pathway	50	89	6309	19747	1,96E+08	0.000355130102223712
hsa-miR-324-3p	ErbB signaling pathway	59	89	8351	19747	4,06E+08	0.000735131332533805
hsa-miR-328-3p	ErbB signaling pathway	53	89	7326	19747	1,36E+09	0.00234658170103451
hsa-miR-335-5p	ErbB signaling pathway	45	89	5890	19747	3,20E+08	0.00544045391222231
hsa-miR-365a-3p	ErbB signaling pathway	39	89	4746	19747	3,17E+09	0.00557797261635606
hsa-miR-423-5p	ErbB signaling pathway	74	89	12169	19747	8,48E+08	0.00147569244347282
hsa-miR-485-3p	ErbB signaling pathway	47	89	6269	19747	2,96E+08	0.00544873966017641
hsa-miR-486-3p	ErbB signaling pathway	78	89	11653	19747	3,16E+05	5,91E+07
hsa-miR-489-3p	ErbB signaling pathway	51	89	6768	19747	7,03E+08	0.00135630249701439
hsa-miR-532-3p	ErbB signaling pathway	61	89	8784	19747	3,80E+08	0.000668955399706658
hsa-miR-532-5p	ErbB signaling pathway	55	89	8143	19747	7,08E+08	0.0122514012978326
hsa-miR-660-5p	ErbB signaling pathway	49	89	5834	19747	4,34E+07	8,11E+09
hsa-miR-92a-3p	ErbB signaling pathway	53	89	8069	19747	0.000279506175320285	0.0472365436291282
hsa-miR-660-5p	Ether lipid metabolism	21	36	5834	19747	0.000300946052126652	0.048452314392391

hsa-miR-125b-5p	Fc epsilon RI signaling pathway	49	82	7799	19747	0.000160369965095176	0.0274232640312752
hsa-miR-328-3p	Fc epsilon RI signaling pathway	47	82	7326	19747	0.000152768490327716	0.0252625395232133
hsa-miR-99b-5p	Fc epsilon RI signaling pathway	23	82	2577	19747	0.00023813985927279	0.0419126152320111
hsa-miR-122-5p	Fc gamma R mediated phagocytosis	73	97	10891	19747	3,24E+09	0.00599893123134428
hsa-miR-145-5p	Fc gamma R mediated phagocytosis	68	97	9602	19747	1,42E+07	0.00253340171130394
hsa-miR-423-5p	Fc gamma R mediated phagocytosis	81	97	12169	19747	2,24E+08	0.000405566471731173
hsa-miR-486-3p	Fc gamma R mediated phagocytosis	76	97	11653	19747	4,44E+09	0.0069250230738725
hsa-miR-148a-3p	Focal adhesion	94	203	6669	19747	0.000134794159807772	0.0223758305280901
hsa-miR-346	Focal adhesion	110	203	8052	19747	7,25E+09	0.0128336520221668
hsa-miR-491-5p	Focal adhesion	140	203	10672	19747	9,33E+07	0.00168823931181989
hsa-miR-193a-5p	Focal adhesion	153	203	11509	19747	2,35E+07	4,41E+09
hsa-miR-212-3p	Focal adhesion	100	203	6789	19747	8,11E+08	0.00145932416644845
hsa-miR-29c-3p	Focal adhesion	107	203	6309	19747	6,26E+04	1,19E+06
hsa-miR-335-5p	Focal adhesion	86	203	5890	19747	9,29E+09	0.0153343931495045
hsa-miR-423-5p	Focal adhesion	158	203	12169	19747	5,43E+07	0.000100973037970855
hsa-miR-486-3p	Focal adhesion	154	203	11653	19747	3,06E+07	5,45E+09
hsa-miR-532-3p	Focal adhesion	118	203	8784	19747	5,96E+09	0.00995806622660956
hsa-miR-148a-3p	Gap junction	48	90	6669	19747	0.000102149310717432	0.0171610842005285
hsa-miR-18a-5p	Gap junction	57	90	8742	19747	0.000204513241813132	0.0366078702845506
hsa-miR-214-3p	Gap junction	68	90	10069	19747	1,48E+08	0.000271967572458856
hsa-miR-365a-3p	Gap junction	40	90	4746	19747	1,68E+09	0.00301252715370393
hsa-let-7c-5p	Glioma	53	65	10808	19747	5,23E+08	0.000967912279811253
hsa-miR-106b-5p	Glioma	47	65	8499	19747	1,63E+08	0.000305623187829552
hsa-miR-125b-5p	Glioma	41	65	7799	19747	0.000100844030056704	0.0174460171998098
hsa-miR-130b-3p	Glioma	47	65	7949	19747	1,59E+07	2,93E+09
hsa-miR-132-3p	Glioma	37	65	6440	19747	4,58E+09	0.0080152473146577
hsa-miR-145-5p	Glioma	48	65	9602	19747	3,00E+09	0.00528271842436351
hsa-miR-148a-3p	Glioma	44	65	6669	19747	2,36E+06	4,51E+08
hsa-miR-18a-5p	Glioma	46	65	8742	19747	1,37E+09	0.00253120249449136
hsa-miR-346	Glioma	43	65	8052	19747	3,09E+09	0.0055695863675911
hsa-miR-491-5p	Glioma	53	65	10672	19747	3,17E+07	0.000586700361120989
hsa-miR-181c-5p	Glioma	43	65	8188	19747	4,99E+09	0.00918601742638116
hsa-miR-193a-5p	Glioma	54	65	11509	19747	1,73E+09	0.0031188332826586

hsa-miR-208a-3p	Glioma	30	65	4498	19747	2,70E+09	0.0049086636530522
hsa-miR-214-3p	Glioma	52	65	10069	19747	1,22E+08	0.000225706195347431
hsa-miR-21-5p	Glioma	43	65	7952	19747	2,16E+09	0.00394575899208744
hsa-miR-221-3p	Glioma	45	65	9076	19747	0.000124572432346924	0.0222984653900993
hsa-miR-29c-3p	Glioma	40	65	6309	19747	8,65E+07	0.00015923257248459
hsa-miR-324-3p	Glioma	47	65	8351	19747	8,94E+07	0.000168043301221628
hsa-miR-328-3p	Glioma	51	65	7326	19747	1,11E+03	2,14E+05
hsa-miR-335-5p	Glioma	40	65	5890	19747	1,14E+07	2,15E+09
hsa-miR-365a-3p	Glioma	36	65	4746	19747	5,54E+06	1,06E+09
hsa-miR-423-5p	Glioma	58	65	12169	19747	6,46E+07	0.000120173299253171
hsa-miR-486-3p	Glioma	59	65	11653	19747	1,33E+06	2,44E+08
hsa-miR-532-3p	Glioma	50	65	8784	19747	9,70E+06	1,80E+09
hsa-miR-532-5p	Glioma	48	65	8143	19747	9,50E+06	1,82E+08
hsa-miR-660-5p	Glioma	41	65	5834	19747	2,14E+06	4,08E+08
hsa-miR-92a-3p	Glioma	47	65	8069	19747	2,70E+07	5,18E+09
hsa-let-7c-5p	Glycerophospholipid metabolism	54	70	10808	19747	8,33E+09	0.0144127485505426
hsa-miR-423-5p	Glycerophospholipid metabolism	60	70	12169	19747	8,67E+08	0.00150859153726278
hsa-let-7c-5p	GnRH signaling pathway	78	105	10808	19747	2,73E+09	0.00485266169527943
hsa-miR-125b-5p	GnRH signaling pathway	64	105	7799	19747	6,88E+07	0.00127266189086473
hsa-miR-346	GnRH signaling pathway	61	105	8052	19747	0.000246231400420671	0.0421055694719347
hsa-miR-491-5p	GnRH signaling pathway	75	105	10672	19747	0.000192773154248205	0.0318075704509538
hsa-miR-214-3p	GnRH signaling pathway	76	105	10069	19747	6,10E+08	0.00110364457278208
hsa-miR-365a-3p	GnRH signaling pathway	43	105	4746	19747	9,05E+09	0.0155712418630718
hsa-miR-423-5p	GnRH signaling pathway	84	105	12169	19747	4,00E+09	0.00675836922586396
hsa-miR-486-3p	GnRH signaling pathway	82	105	11653	19747	2,79E+09	0.00435468859023034
hsa-miR-346	Hedgehog signaling pathway	37	56	8052	19747	0.000113907761329715	0.0199338582327002
hsa-miR-532-3p	Hedgehog signaling pathway	39	56	8784	19747	0.000122942134432622	0.02016251004695
hsa-miR-532-5p	Hedgehog signaling pathway	40	56	8143	19747	4,55E+08	0.000846368813507935

hsa-miR-486-3p	Hypertrophic cardiomyopathy HCM	70	86	11653	19747	7,94E+08	0.00127909282543971
hsa-miR-660-5p	Hypertrophic cardiomyopathy HCM	41	86	5834	19747	0.000290082258520785	0.0467032436218464
hsa-miR-130b-3p	Inositol phosphate metabolism	36	54	7949	19747	7,75E+09	0.0124730140588347
hsa-miR-145-5p	Inositol phosphate metabolism	42	54	9602	19747	1,07E+09	0.00191635733016458
hsa-miR-215-5p	Inositol phosphate metabolism	26	54	4762	19747	0.000103429329945004	0.0195481433596058
hsa-miR-485-3p	Inositol phosphate metabolism	32	54	6269	19747	2,77E+09	0.0050892437533356
hsa-miR-486-3p	Inositol phosphate metabolism	45	54	11653	19747	0.000114860879468062	0.0178034363175496
hsa-miR-532-5p	Inositol phosphate metabolism	36	54	8143	19747	0.000139718578748769	0.0237521583872908
hsa-let-7c-5p	Insulin signaling pathway	102	139	10808	19747	4,32E+08	0.000802904623297297
hsa-miR-125b-5p	Insulin signaling pathway	82	139	7799	19747	2,47E+08	0.000462268299250126
hsa-miR-130b-3p	Insulin signaling pathway	80	139	7949	19747	2,69E+09	0.00453952633954071
hsa-miR-132-3p	Insulin signaling pathway	72	139	6440	19747	2,11E+08	0.000394830658472681
hsa-miR-145-5p	Insulin signaling pathway	91	139	9602	19747	4,35E+09	0.0076061686720977
hsa-miR-148a-3p	Insulin signaling pathway	70	139	6669	19747	3,82E+09	0.0066909532523744
hsa-miR-18a-5p	Insulin signaling pathway	83	139	8742	19747	0.000172036171731546	0.0309665109116782
hsa-miR-346	Insulin signaling pathway	83	139	8052	19747	4,84E+08	0.000900410789909162
hsa-miR-491-5p	Insulin signaling pathway	108	139	10672	19747	5,49E+05	1,05E+08
hsa-miR-708-5p	Insulin signaling pathway	90	139	9879	19747	0.000312895843538583	0.0560083559934064
hsa-miR-193a-5p	Insulin signaling pathway	106	139	11509	19747	6,45E+08	0.00116802999418971
hsa-miR-212-3p	Insulin signaling pathway	68	139	6789	19747	0.000274733268255001	0.0442320561890551
hsa-miR-214-3p	Insulin signaling pathway	102	139	10069	19747	5,25E+06	1,00E+09
hsa-miR-21-5p	Insulin signaling pathway	80	139	7952	19747	2,73E+09	0.0049941158882415

hsa-miR-221-3p	Insulin signaling pathway	87	139	9076	19747	5,61E+09	0.0101485714583737
hsa-miR-27a-3p	Insulin signaling pathway	92	139	9729	19747	3,95E+09	0.00727320897302096
hsa-miR-29c-3p	Insulin signaling pathway	72	139	6309	19747	9,01E+07	0.000165714354952539
hsa-miR-324-3p	Insulin signaling pathway	85	139	8351	19747	5,44E+08	0.0009788860052078
hsa-miR-328-3p	Insulin signaling pathway	79	139	7326	19747	1,66E+08	0.000296119254649243
hsa-miR-335-5p	Insulin signaling pathway	72	139	5890	19747	4,58E+06	8,69E+08
hsa-miR-365a-3p	Insulin signaling pathway	58	139	4746	19747	2,96E+08	0.000552928301281952
hsa-miR-423-5p	Insulin signaling pathway	113	139	12169	19747	4,10E+06	7,63E+09
hsa-miR-451a	Insulin signaling pathway	29	139	1963	19747	8,89E+09	0.0166206950421471
hsa-miR-486-3p	Insulin signaling pathway	115	139	11653	19747	1,53E+05	2,88E+07
hsa-miR-489-3p	Insulin signaling pathway	68	139	6768	19747	0.000248261384259041	0.0451835719351454
hsa-miR-532-3p	Insulin signaling pathway	94	139	8784	19747	2,84E+06	5,40E+08
hsa-miR-532-5p	Insulin signaling pathway	86	139	8143	19747	6,96E+07	0.000132943658911327
hsa-miR-660-5p	Insulin signaling pathway	71	139	5834	19747	7,71E+06	1,46E+09
hsa-miR-92a-3p	Insulin signaling pathway	83	139	8069	19747	5,33E+08	0.00099754783314666
hsa-miR-27a-3p	Keratan sulfate biosynthesis	15	15	9729	19747	2,43E+08	0.00447655950578362
hsa-miR-125b-5p	Leukocyte transendothelial migration	68	116	7799	19747	2,31E+09	0.00415781849855753
hsa-miR-130b-3p	Long term depression	45	73	7949	19747	0.000177545772729403	0.0280522320912457
hsa-miR-132-3p	Long term depression	43	73	6440	19747	3,38E+07	0.000628510372007501
hsa-miR-212-3p	Long term depression	45	73	6789	19747	1,75E+08	0.00032112493894482
hsa-miR-214-3p	Long term depression	54	73	10069	19747	4,94E+09	0.00865333742157033
hsa-let-7c-5p	Long term potentiation	58	71	10808	19747	1,66E+08	0.000312832748489529
hsa-miR-130b-3p	Long term potentiation	46	71	7949	19747	2,51E+09	0.00425019113001135
hsa-miR-132-3p	Long term potentiation	40	71	6440	19747	3,17E+09	0.00557793533225043
hsa-miR-148a-3p	Long term potentiation	40	71	6669	19747	7,73E+09	0.0132207404298169

hsa-miR-212-3p	Long term potentiation	43	71	6789	19747	5,71E+08	0.00103273096699323
hsa-miR-214-3p	Long term potentiation	59	71	10069	19747	1,62E+06	3,12E+08
hsa-miR-221-3p	Long term potentiation	51	71	9076	19747	8,71E+08	0.00163717957101565
hsa-miR-324-3p	Long term potentiation	48	71	8351	19747	1,42E+09	0.00251927653370422
hsa-miR-328-3p	Long term potentiation	43	71	7326	19747	4,93E+09	0.00833170338950688
hsa-miR-365a-3p	Long term potentiation	32	71	4746	19747	8,19E+09	0.0141646598512706
hsa-miR-423-5p	Long term potentiation	61	71	12169	19747	6,18E+08	0.00108731410312022
hsa-miR-485-3p	Long term potentiation	39	71	6269	19747	4,36E+09	0.00797164152468054
hsa-miR-486-3p	Long term potentiation	63	71	11653	19747	3,64E+06	6,58E+07
hsa-miR-532-3p	Long term potentiation	47	71	8784	19747	0.000180699113001072	0.0294539554191748
hsa-miR-636	Long term potentiation	47	71	8717	19747	0.000144645936648675	0.0261809145334101
hsa-miR-652-3p	Long term potentiation	41	71	6694	19747	3,18E+09	0.00604080960143702
hsa-miR-130b-3p	Lysosome	68	121	7949	19747	0.000275012367236029	0.0431769416560565
hsa-let-7c-5p	MAPK signaling pathway	189	272	10808	19747	3,90E+07	7,44E+09
hsa-miR-106b-5p	MAPK signaling pathway	152	272	8499	19747	1,22E+09	0.00219403424876339
hsa-miR-125b-5p	MAPK signaling pathway	156	272	7799	19747	1,60E+05	3,11E+07
hsa-miR-130b-3p	MAPK signaling pathway	145	272	7949	19747	8,08E+08	0.00143798895207376
hsa-miR-132-3p	MAPK signaling pathway	123	272	6440	19747	8,65E+08	0.00158348970165867
hsa-miR-145-5p	MAPK signaling pathway	167	272	9602	19747	1,37E+09	0.00243436825785025
hsa-miR-148a-3p	MAPK signaling pathway	123	272	6669	19747	5,28E+09	0.00907513562369466
hsa-miR-18a-5p	MAPK signaling pathway	154	272	8742	19747	2,56E+09	0.00470345214684365
hsa-miR-346	MAPK signaling pathway	149	272	8052	19747	1,89E+08	0.000357356146119708
hsa-miR-491-5p	MAPK signaling pathway	181	272	10672	19747	1,66E+09	0.00296322595415982
hsa-miR-708-5p	MAPK signaling pathway	166	272	9879	19747	0.000155385511762709	0.0285909341643385
hsa-miR-181c-5p	MAPK signaling pathway	148	272	8188	19747	9,97E+08	0.00186431833798245
hsa-miR-193a-5p	MAPK signaling pathway	199	272	11509	19747	1,98E+07	3,73E+09
hsa-miR-212-3p	MAPK signaling pathway	136	272	6789	19747	6,93E+05	1,32E+09

hsa-miR-214-3p	MAPK signaling pathway	181	272	10069	19747	1,26E+07	2,40E+09
hsa-miR-21-5p	MAPK signaling pathway	145	272	7952	19747	8,27E+08	0.00153017372899776
hsa-miR-27a-3p	MAPK signaling pathway	167	272	9729	19747	3,43E+09	0.00630583344550976
hsa-miR-324-3p	MAPK signaling pathway	158	272	8351	19747	9,48E+06	1,81E+08
hsa-miR-328-3p	MAPK signaling pathway	145	272	7326	19747	3,21E+06	6,09E+07
hsa-miR-335-5p	MAPK signaling pathway	110	272	5890	19747	0.000111490500405376	0.0182844420664816
hsa-miR-365a-3p	MAPK signaling pathway	99	272	4746	19747	2,94E+08	0.000549544127069201
hsa-miR-423-5p	MAPK signaling pathway	214	272	12169	19747	1,02E+05	1,94E+07
hsa-miR-485-3p	MAPK signaling pathway	124	272	6269	19747	1,08E+08	0.000204278671164237
hsa-miR-486-3p	MAPK signaling pathway	207	272	11653	19747	1,87E+05	3,49E+07
hsa-miR-489-3p	MAPK signaling pathway	131	272	6768	19747	1,38E+08	0.000267072986048777
hsa-miR-532-3p	MAPK signaling pathway	164	272	8784	19747	9,66E+06	1,80E+09
hsa-miR-532-5p	MAPK signaling pathway	148	272	8143	19747	7,02E+08	0.00129153403925789
hsa-miR-636	MAPK signaling pathway	152	272	8717	19747	5,95E+09	0.010886009662172
hsa-miR-652-3p	MAPK signaling pathway	122	272	6694	19747	0.000104552638540367	0.0195513434070487
hsa-miR-660-5p	MAPK signaling pathway	119	272	5834	19747	4,01E+07	7,50E+09
hsa-miR-92a-3p	MAPK signaling pathway	150	272	8069	19747	1,20E+08	0.000228541509148601
hsa-miR-99b-5p	MAPK signaling pathway	57	272	2577	19747	0.000175433151962223	0.0312271010492757
hsa-miR-125b-5p	Melanogenesis	60	102	7799	19747	5,94E+09	0.0104537888963757
hsa-miR-130b-3p	Melanogenesis	61	102	7949	19747	5,05E+09	0.00827681468905295
hsa-miR-145-5p	Melanogenesis	68	102	9602	19747	0.000172025442691565	0.028900274372183
hsa-miR-148a-3p	Melanogenesis	52	102	6669	19747	0.000244139937262184	0.0395506698364739
hsa-miR-346	Melanogenesis	61	102	8052	19747	7,87E+09	0.0138497405316457
hsa-miR-491-5p	Melanogenesis	77	102	10672	19747	6,12E+08	0.00111446036262965
hsa-miR-214-3p	Melanogenesis	75	102	10069	19747	2,63E+08	0.00047948006365639

hsa-miR-324-3p	Melanogenesis	68	102	8351	19747	5,57E+07	0.000104779156427483
hsa-miR-328-3p	Melanogenesis	61	102	7326	19747	2,58E+08	0.000457161214934527
hsa-miR-423-5p	Melanogenesis	85	102	12169	19747	1,50E+08	0.000275088224080034
hsa-miR-486-3p	Melanogenesis	81	102	11653	19747	9,89E+07	0.00158248344797178
hsa-miR-532-3p	Melanogenesis	68	102	8784	19747	4,94E+08	0.00086469973570403
hsa-miR-106b-5p	Melanoma	47	71	8499	19747	6,84E+09	0.0121089322914697
hsa-miR-130b-3p	Melanoma	45	71	7949	19747	6,82E+09	0.0110561024331037
hsa-miR-148a-3p	Melanoma	44	71	6669	19747	1,07E+08	0.000197045203505266
hsa-miR-208a-3p	Melanoma	30	71	4498	19747	0.000199031080602374	0.0344323769442108
hsa-miR-324-3p	Melanoma	47	71	8351	19747	4,03E+08	0.00692396541792366
hsa-miR-328-3p	Melanoma	46	71	7326	19747	1,98E+08	0.000350017972836946
hsa-miR-335-5p	Melanoma	41	71	5890	19747	9,08E+07	0.000168064060732455
hsa-miR-365a-3p	Melanoma	34	71	4746	19747	1,01E+09	0.00185634247650845
hsa-miR-532-3p	Melanoma	47	71	8784	19747	0.000180699113001072	0.0294539554191748
hsa-miR-532-5p	Melanoma	46	71	8143	19747	5,14E+08	0.0090005088602933
hsa-miR-708-5p	mTOR signaling pathway	40	53	9879	19747	0.000133889936772226	0.0247696383028618
hsa-miR-29c-3p	mTOR signaling pathway	33	53	6309	19747	5,41E+08	0.000973476483924731
hsa-miR-324-3p	mTOR signaling pathway	37	53	8351	19747	4,64E+09	0.00798031049984938
hsa-miR-328-3p	mTOR signaling pathway	38	53	7326	19747	3,20E+07	5,91E+09
hsa-miR-365a-3p	mTOR signaling pathway	29	53	4746	19747	1,51E+08	0.000283487893082829
hsa-let-7c-5p	Neurotrophin signaling pathway	92	129	10808	19747	7,90E+09	0.0136732809365003
hsa-miR-106b-5p	Neurotrophin signaling pathway	90	129	8499	19747	6,98E+04	1,34E+07
hsa-miR-122-5p	Neurotrophin signaling pathway	93	129	10891	19747	5,43E+09	0.00993213946174396
hsa-miR-125b-5p	Neurotrophin signaling pathway	72	129	7799	19747	0.000123361190179247	0.0212181247108305
hsa-miR-130b-3p	Neurotrophin signaling pathway	77	129	7949	19747	6,14E+08	0.00109946098932277
hsa-miR-132-3p	Neurotrophin signaling pathway	67	129	6440	19747	4,16E+08	0.000774617861411769
hsa-miR-145-5p	Neurotrophin signaling pathway	89	129	9602	19747	2,12E+08	0.00039276120599038
hsa-miR-148a-3p	Neurotrophin signaling pathway	70	129	6669	19747	1,30E+08	0.000241145820353463

hsa-miR-18a-5p	Neurotrophin signaling pathway	85	129	8742	19747	5,69E+07	0.000108031226473923
hsa-miR-346	Neurotrophin signaling pathway	75	129	8052	19747	4,92E+09	0.00879816530250184
hsa-miR-491-5p	Neurotrophin signaling pathway	96	129	10672	19747	1,40E+08	0.000262025246912301
hsa-miR-181c-5p	Neurotrophin signaling pathway	74	129	8188	19747	0.000188305783789304	0.0338950410820747
hsa-miR-193a-5p	Neurotrophin signaling pathway	101	129	11509	19747	1,26E+08	0.000233702669849076
hsa-miR-208a-3p	Neurotrophin signaling pathway	48	129	4498	19747	0.000146224502472592	0.0254430634302309
hsa-miR-212-3p	Neurotrophin signaling pathway	70	129	6789	19747	2,70E+07	0.000494325673569598
hsa-miR-214-3p	Neurotrophin signaling pathway	93	129	10069	19747	7,77E+07	0.000143774788205373
hsa-miR-221-3p	Neurotrophin signaling pathway	83	129	9076	19747	1,91E+08	0.00354700763489317
hsa-miR-27a-3p	Neurotrophin signaling pathway	92	129	9729	19747	2,79E+07	5,35E+08
hsa-miR-29c-3p	Neurotrophin signaling pathway	66	129	6309	19747	4,37E+08	0.000786917436035729
hsa-miR-324-3p	Neurotrophin signaling pathway	78	129	8351	19747	2,32E+09	0.00403819445295394
hsa-miR-328-3p	Neurotrophin signaling pathway	77	129	7326	19747	1,48E+07	2,79E+08
hsa-miR-335-5p	Neurotrophin signaling pathway	65	129	5890	19747	7,40E+07	0.000136943811526956
hsa-miR-365a-3p	Neurotrophin signaling pathway	61	129	4746	19747	6,87E+05	1,33E+08
hsa-miR-410-3p	Neurotrophin signaling pathway	72	129	7692	19747	7,40E+09	0.0137698718261168
hsa-miR-423-5p	Neurotrophin signaling pathway	102	129	12169	19747	1,60E+09	0.00275806346269027
hsa-miR-485-3p	Neurotrophin signaling pathway	65	129	6269	19747	7,84E+08	0.0014654553605142
hsa-miR-486-3p	Neurotrophin signaling pathway	108	129	11653	19747	1,22E+05	2,28E+06
hsa-miR-532-3p	Neurotrophin signaling pathway	84	129	8784	19747	1,76E+08	0.000315784991147318
hsa-miR-532-5p	Neurotrophin signaling pathway	79	129	8143	19747	3,45E+08	0.000644277831502583
hsa-miR-660-5p	Neurotrophin signaling pathway	60	129	5834	19747	3,33E+08	0.00585236114320686

hsa-miR-92a-3p	Neurotrophin signaling pathway	82	129	8069	19747	1,53E+07	2,93E+09
hsa-miR-99b-5p	Neurotrophin signaling pathway	34	129	2577	19747	3,68E+09	0.00666056138770621
hsa-miR-106b-5p	Non small cell lung cancer	39	54	8499	19747	1,32E+08	0.00237217711283513
hsa-miR-125b-5p	Non small cell lung cancer	35	54	7799	19747	0.000145752280504699	0.0249236399663036
hsa-miR-130b-3p	Non small cell lung cancer	41	54	7949	19747	1,04E+07	1,92E+09
hsa-miR-148a-3p	Non small cell lung cancer	34	54	6669	19747	1,07E+09	0.00195631188083357
hsa-miR-491-5p	Non small cell lung cancer	43	54	10672	19747	8,13E+09	0.0139089003540398
hsa-miR-193a-5p	Non small cell lung cancer	47	54	11509	19747	4,52E+08	0.000826353052515074
hsa-miR-21-5p	Non small cell lung cancer	35	54	7952	19747	0.000229038790460666	0.0409979434924593
hsa-miR-29c-3p	Non small cell lung cancer	32	54	6309	19747	3,19E+09	0.00561336088749214
hsa-miR-324-3p	Non small cell lung cancer	39	54	8351	19747	7,98E+08	0.00142887272862188
hsa-miR-328-3p	Non small cell lung cancer	39	54	7326	19747	1,63E+06	3,04E+09
hsa-miR-335-5p	Non small cell lung cancer	32	54	5890	19747	6,65E+07	0.00119050908422581
hsa-miR-365a-3p	Non small cell lung cancer	27	54	4746	19747	3,09E+09	0.00543400853622877
hsa-miR-423-5p	Non small cell lung cancer	47	54	12169	19747	3,60E+08	0.00612212788130467
hsa-miR-485-3p	Non small cell lung cancer	30	54	6269	19747	0.000247690695217988	0.0435935623583658
hsa-miR-486-3p	Non small cell lung cancer	48	54	11653	19747	1,45E+06	0.000249886960689084
hsa-miR-532-3p	Non small cell lung cancer	43	54	8784	19747	1,32E+07	2,44E+09
hsa-miR-532-5p	Non small cell lung cancer	38	54	8143	19747	1,39E+09	0.00250729833240173
hsa-miR-660-5p	Non small cell lung cancer	33	54	5834	19747	1,44E+08	0.000268502131403106
hsa-miR-92a-3p	Non small cell lung cancer	36	54	8069	19747	0.00011190475609124	0.0194714275598758
hsa-miR-324-3p	Notch signaling pathway	33	47	8351	19747	9,96E+09	0.0166337560273588

hsa-miR-423-5p	Notch signaling pathway	42	47	12169	19747	2,26E+09	0.00385702819176824
hsa-miR-486-3p	Notch signaling pathway	42	47	11653	19747	4,96E+08	0.000813838181758521
hsa-miR-130b-3p	p53 signaling pathway	42	68	7949	19747	0.000271649792095939	0.0426490173590625
hsa-miR-221-3p	p53 signaling pathway	49	68	9076	19747	1,13E+09	0.00211384040423837
hsa-miR-29c-3p	p53 signaling pathway	36	68	6309	19747	0.000263629686948291	0.0445534170942611
hsa-miR-636	p53 signaling pathway	47	68	8717	19747	2,72E+09	0.00503592625906459
hsa-miR-106b-5p	Pancreatic cancer	53	75	8499	19747	1,16E+08	0.000218944506316483
hsa-miR-122-5p	Pancreatic cancer	57	75	10891	19747	0.000147456459021973	0.0265421626239552
hsa-miR-125b-5p	Pancreatic cancer	47	75	7799	19747	4,09E+09	0.0072358806698931
hsa-miR-130b-3p	Pancreatic cancer	56	75	7949	19747	1,43E+05	2,71E+06
hsa-miR-132-3p	Pancreatic cancer	41	75	6440	19747	6,52E+09	0.0113516536651965
hsa-miR-148a-3p	Pancreatic cancer	46	75	6669	19747	9,33E+07	0.000172678555941122
hsa-miR-346	Pancreatic cancer	46	75	8052	19747	0.000255087007807125	0.0436198783350184
hsa-miR-491-5p	Pancreatic cancer	57	75	10672	19747	7,00E+09	0.0120467968377094
hsa-miR-193a-5p	Pancreatic cancer	60	75	11509	19747	5,72E+09	0.0100043454395511
hsa-miR-208a-3p	Pancreatic cancer	34	75	4498	19747	1,30E+09	0.00240439124463254
hsa-miR-21-5p	Pancreatic cancer	48	75	7952	19747	2,75E+08	0.00503410322590078
hsa-miR-29c-3p	Pancreatic cancer	43	75	6309	19747	4,94E+08	0.000888418260622003
hsa-miR-324-3p	Pancreatic cancer	53	75	8351	19747	6,03E+07	0.00011333180445753
hsa-miR-328-3p	Pancreatic cancer	48	75	7326	19747	2,01E+08	0.000355962369759287
hsa-miR-335-5p	Pancreatic cancer	42	75	5890	19747	2,05E+08	0.000375483613653101
hsa-miR-365a-3p	Pancreatic cancer	32	75	4746	19747	0.000285691059146997	0.0474247158184016
hsa-miR-485-3p	Pancreatic cancer	39	75	6269	19747	0.000210355026076009	0.0372328396154536
hsa-miR-486-3p	Pancreatic cancer	63	75	11653	19747	2,84E+08	0.000477269240312648
hsa-miR-532-3p	Pancreatic cancer	59	75	8784	19747	1,44E+05	2,77E+07
hsa-miR-532-5p	Pancreatic cancer	48	75	8143	19747	5,67E+09	0.00986858676811251
hsa-miR-660-5p	Pancreatic cancer	41	75	5834	19747	4,73E+08	0.000864782014018591
hsa-miR-92a-3p	Pancreatic cancer	47	75	8069	19747	0.000110754473882535	0.019271278455561
hsa-let-7c-5p	Pathways in cancer	230	330	10808	19747	1,48E+06	2,87E+08
hsa-miR-106b-5p	Pathways in cancer	196	330	8499	19747	1,27E+04	2,44E+07
hsa-miR-122-5p	Pathways in cancer	230	330	10891	19747	3,49E+06	6,69E+08
hsa-miR-125b-5p	Pathways in cancer	174	330	7799	19747	6,40E+07	0.000120874524466646
hsa-miR-130b-3p	Pathways in cancer	201	330	7949	19747	1,95E+00	3,78E+02
hsa-miR-132-3p	Pathways in cancer	156	330	6440	19747	1,73E+06	3,33E+08
hsa-miR-145-5p	Pathways in cancer	216	330	9602	19747	3,86E+03	7,44E+06
hsa-miR-148a-3p	Pathways in cancer	165	330	6669	19747	6,66E+04	1,29E+07

hsa-miR-18a-5p	Pathways in cancer	201	330	8742	19747	6,79E+04	1,32E+07
hsa-miR-346	Pathways in cancer	186	330	8052	19747	6,23E+05	1,20E+08
hsa-miR-491-5p	Pathways in cancer	234	330	10672	19747	1,93E+04	3,70E+06
hsa-miR-708-5p	Pathways in cancer	215	330	9879	19747	1,64E+05	3,17E+08
hsa-miR-181c-5p	Pathways in cancer	183	330	8188	19747	1,70E+07	3,30E+09
hsa-miR-193a-5p	Pathways in cancer	243	330	11509	19747	3,42E+05	6,59E+06
hsa-miR-208a-3p	Pathways in cancer	117	330	4498	19747	9,51E+06	1,82E+08
hsa-miR-212-3p	Pathways in cancer	167	330	6789	19747	7,44E+04	1,42E+06
hsa-miR-214-3p	Pathways in cancer	216	330	10069	19747	6,11E+06	1,17E+08
hsa-miR-215-5p	Pathways in cancer	114	330	4762	19747	1,12E+09	0.00214786402434441
hsa-miR-21-5p	Pathways in cancer	184	330	7952	19747	7,48E+05	1,44E+08
hsa-miR-221-3p	Pathways in cancer	202	330	9076	19747	1,45E+06	2,79E+08
hsa-miR-27a-3p	Pathways in cancer	203	330	9729	19747	4,34E+08	0.000824443679402642
hsa-miR-29c-3p	Pathways in cancer	170	330	6309	19747	9,15E+00	1,76E+03
hsa-miR-324-3p	Pathways in cancer	195	330	8351	19747	4,47E+04	8,63E+06
hsa-miR-328-3p	Pathways in cancer	187	330	7326	19747	2,77E+01	5,37E+03
hsa-miR-335-5p	Pathways in cancer	147	330	5890	19747	8,67E+05	1,66E+07
hsa-miR-365a-3p	Pathways in cancer	115	330	4746	19747	5,50E+08	0.00101741918513087
sa-miR-410-3p	Pathways in cancer	180	330	7692	19747	5,40E+05	1,04E+08
hsa-miR-423-5p	Pathways in cancer	254	330	12169	19747	1,57E+05	3,01E+07
hsa-miR-451a	Pathways in cancer	58	330	1963	19747	1,23E+09	0.00233202732370519
hsa-miR-485-3p	Pathways in cancer	149	330	6269	19747	1,93E+07	3,71E+09
hsa-miR-486-3p	Pathways in cancer	249	330	11653	19747	1,97E+04	3,77E+06
hsa-miR-489-3p	Pathways in cancer	148	330	6768	19747	3,88E+07	0.0072973637089766
hsa-miR-532-3p	Pathways in cancer	215	330	8784	19747	2,07E+00	4,02E+02
hsa-miR-532-5p	Pathways in cancer	190	330	8143	19747	1,22E+05	2,37E+07
hsa-miR-636	Pathways in cancer	194	330	8717	19747	5,05E+06	9,75E+08
hsa-miR-660-5p	Pathways in cancer	145	330	5834	19747	1,63E+06	3,12E+08
hsa-miR-92a-3p	Pathways in cancer	181	330	8069	19747	1,66E+07	3,19E+09
hsa-miR-99b-5p	Pathways in cancer	81	330	2577	19747	8,64E+05	1,60E+07
hsa-miR-489-3p	Pentose and glucuronate interconversions	20	28	6768	19747	6,62E+09	0.0123107999596767
hsa-let-7c-5p	Phosphatidylinositol signaling system	61	76	10808	19747	2,83E+08	0.000525732378273309
hsa-miR-122-5p	Phosphatidylinositol signaling system	59	76	10891	19747	3,79E+09	0.0069821397051294
hsa-miR-130b-3p	Phosphatidylinositol signaling system	52	76	7949	19747	6,17E+07	0.000113533714926217

hsa-miR-145-5p	Phosphatidylinositol signaling system	58	76	9602	19747	7,13E+07	0.000133371552748095
hsa-miR-148a-3p	Phosphatidylinositol signaling system	43	76	6669	19747	3,67E+09	0.00642628088261692
hsa-miR-346	Phosphatidylinositol signaling system	47	76	8052	19747	0.000165620442848693	0.028652336612824
hsa-miR-491-5p	Phosphatidylinositol signaling system	57	76	10672	19747	0.000132869585144046	0.0224549598893437
hsa-miR-212-3p	Phosphatidylinositol signaling system	42	76	6789	19747	0.000148565705742392	0.0248104728589794
hsa-miR-214-3p	Phosphatidylinositol signaling system	54	76	10069	19747	0.000290659554852816	0.0473775074410091
hsa-miR-221-3p	Phosphatidylinositol signaling system	52	76	9076	19747	6,23E+09	0.0112847667648139
hsa-miR-27a-3p	Phosphatidylinositol signaling system	53	76	9729	19747	0.000234043318612097	0.0414256673943412
hsa-miR-324-3p	Phosphatidylinositol signaling system	49	76	8351	19747	7,77E+09	0.0131028472135113
hsa-miR-335-5p	Phosphatidylinositol signaling system	40	76	5890	19747	2,63E+09	0.00449844353649673
hsa-miR-423-5p	Phosphatidylinositol signaling system	63	76	12169	19747	4,81E+09	0.00812995859327178
hsa-miR-485-3p	Phosphatidylinositol signaling system	42	76	6269	19747	1,84E+09	0.00340796523027464
hsa-miR-486-3p	Phosphatidylinositol signaling system	66	76	11653	19747	1,17E+07	2,10E+09
hsa-miR-532-3p	Phosphatidylinositol signaling system	56	76	8784	19747	2,20E+07	4,07E+09
hsa-miR-532-5p	Phosphatidylinositol signaling system	49	76	8143	19747	3,58E+09	0.00633533195275275
hsa-miR-636	Phosphatidylinositol signaling system	51	76	8717	19747	4,41E+09	0.00812034270099557
hsa-miR-652-3p	Phosphatidylinositol signaling system	41	76	6694	19747	0.000252167518262152	0.0456423208054495
hsa-miR-335-5p	Progesterone mediated oocyte maturation	46	88	5890	19747	8,55E+08	0.00152182599611379
hsa-let-7c-5p	Prostate cancer	65	89	10808	19747	0.000288577946387408	0.0473267832075349
hsa-miR-106b-5p	Prostate cancer	59	89	8499	19747	7,89E+08	0.00142816038638304
hsa-miR-130b-3p	Prostate cancer	61	89	7949	19747	6,07E+06	1,13E+09
hsa-miR-132-3p	Prostate cancer	47	89	6440	19747	6,32E+09	0.0109962469654898
hsa-miR-145-5p	Prostate cancer	62	89	9602	19747	4,57E+09	0.0079909418024209
hsa-miR-148a-3p	Prostate cancer	50	89	6669	19747	1,15E+09	0.00208582444335348
hsa-miR-193a-5p	Prostate cancer	69	89	11509	19747	0.000105672550472159	0.0182813512316834

hsa-miR-208a-3p	Prostate cancer	37	89	4498	19747	5,78E+07	0.0102964426920193
hsa-miR-214-3p	Prostate cancer	66	89	10069	19747	6,40E+08	0.00115809210654087
hsa-miR-221-3p	Prostate cancer	59	89	9076	19747	8,51E+09	0.0153215506622363
hsa-miR-29c-3p	Prostate cancer	53	89	6309	19747	7,30E+05	1,37E+09
hsa-miR-328-3p	Prostate cancer	56	89	7326	19747	6,59E+07	0.000119256685760116
hsa-miR-335-5p	Prostate cancer	50	89	5890	19747	1,97E+07	3,70E+09
hsa-miR-365a-3p	Prostate cancer	38	89	4746	19747	7,92E+09	0.0136931674990255
hsa-miR-486-3p	Prostate cancer	72	89	11653	19747	9,02E+08	0.0014433888658689
hsa-miR-532-3p	Prostate cancer	61	89	8784	19747	3,80E+08	0.000668955399706658
hsa-miR-532-5p	Prostate cancer	57	89	8143	19747	1,15E+09	0.00207260789621706
hsa-miR-660-5p	Prostate cancer	44	89	5834	19747	6,07E+09	0.0104347506356288
hsa-miR-92a-3p	Prostate cancer	53	89	8069	19747	0.000279506175320285	0.0472365436291282
hsa-miR-125b-5p	Regulation of actin cytoskeleton	119	212	7799	19747	6,32E+07	0.000119379000069116
hsa-miR-130b-3p	Regulation of actin cytoskeleton	111	212	7949	19747	0.000226000764629747	0.0354821200468702
hsa-miR-145-5p	Regulation of actin cytoskeleton	138	212	9602	19747	8,69E+07	0.000161692694021296
hsa-miR-148a-3p	Regulation of actin cytoskeleton	96	212	6669	19747	0.000310508428511325	0.0496813485618119
hsa-miR-346	Regulation of actin cytoskeleton	118	212	8052	19747	7,84E+08	0.00145114025746149
hsa-miR-193a-5p	Regulation of actin cytoskeleton	155	212	11509	19747	4,55E+08	0.000832357211691079
hsa-miR-214-3p	Regulation of actin cytoskeleton	134	212	10069	19747	0.000208167838919234	0.0343476934216737
hsa-miR-328-3p	Regulation of actin cytoskeleton	111	212	7326	19747	3,87E+08	0.000681869988742886
hsa-miR-335-5p	Regulation of actin cytoskeleton	87	212	5890	19747	0.000311846895446166	0.0495836563759404
hsa-miR-423-5p	Regulation of actin cytoskeleton	158	212	12169	19747	4,54E+09	0.00766833435469284
hsa-miR-486-3p	Regulation of actin cytoskeleton	154	212	11653	19747	2,28E+09	0.00357457750963939
hsa-miR-532-3p	Regulation of actin cytoskeleton	126	212	8784	19747	7,80E+08	0.00136423577161286
hsa-miR-106b-5p	Renal cell carcinoma	49	71	8499	19747	8,53E+08	0.00154409975165512
hsa-miR-132-3p	Renal cell carcinoma	39	71	6440	19747	8,48E+09	0.0146647645114278
hsa-miR-148a-3p	Renal cell carcinoma	39	71	6669	19747	0.000196686238324257	0.0320598568468539
hsa-miR-18a-5p	Renal cell carcinoma	49	71	8742	19747	2,16E+09	0.00399170072540255
hsa-miR-208a-3p	Renal cell carcinoma	30	71	4498	19747	0.000199031080602374	0.0344323769442108

hsa-miR-29c-3p	Renal cell carcinoma	40	71	6309	19747	1,85E+09	0.00326273801755925
hsa-miR-324-3p	Renal cell carcinoma	46	71	8351	19747	0.000107244477097077	0.0179098276752119
hsa-miR-328-3p	Renal cell carcinoma	46	71	7326	19747	1,98E+08	0.000350017972836946
hsa-miR-335-5p	Renal cell carcinoma	40	71	5890	19747	2,91E+08	0.000529536288562586
hsa-miR-410-3p	Renal cell carcinoma	44	71	7692	19747	7,14E+09	0.0132742121907547
hsa-miR-423-5p	Renal cell carcinoma	58	71	12169	19747	0.000215009409546296	0.0341864961178611
hsa-miR-486-3p	Renal cell carcinoma	59	71	11653	19747	1,18E+09	0.00187871757007912
hsa-miR-532-3p	Renal cell carcinoma	50	71	8784	19747	8,58E+08	0.00150110885800276
hsa-miR-660-5p	Renal cell carcinoma	39	71	5834	19747	6,86E+08	0.00124894702887157
hsa-miR-106b-5p	Small cell lung cancer	54	84	8499	19747	6,86E+09	0.0121421269095395
hsa-miR-122-5p	Small cell lung cancer	63	84	10891	19747	0.000132181458542993	0.0239248439962818
hsa-miR-130b-3p	Small cell lung cancer	52	84	7949	19747	4,88E+09	0.00800181414317739
hsa-miR-181c-5p	Small cell lung cancer	53	84	8188	19747	5,06E+09	0.00930728039800068
hsa-miR-208a-3p	Small cell lung cancer	34	84	4498	19747	0.000210960508870817	0.0364961680346513
hsa-miR-212-3p	Small cell lung cancer	45	84	6789	19747	0.000230348497617942	0.0375468051117246
hsa-miR-221-3p	Small cell lung cancer	55	84	9076	19747	0.000239039309015484	0.0425489970047561
hsa-miR-29c-3p	Small cell lung cancer	54	84	6309	19747	1,12E+05	2,12E+07
hsa-miR-335-5p	Small cell lung cancer	43	84	5890	19747	3,24E+09	0.00550867911462605
hsa-miR-486-3p	Small cell lung cancer	65	84	11653	19747	0.000301651417701395	0.0452477126552093
hsa-miR-532-3p	Small cell lung cancer	55	84	8784	19747	8,28E+09	0.0136651272674349
hsa-miR-532-5p	Small cell lung cancer	52	84	8143	19747	0.000103566251734075	0.0177098290465269
hsa-miR-636	Small cell lung cancer	54	84	8717	19747	0.000154717757328096	0.0278491963190573
hsa-miR-660-5p	Small cell lung cancer	43	84	5834	19747	2,51E+09	0.00444564603256873
hsa-miR-130b-3p	SNARE interactions in vesicular transport	28	39	7949	19747	6,52E+09	0.010563500056598
hsa-miR-148a-3p	SNARE interactions in vesicular transport	25	39	6669	19747	0.000103269784166448	0.0173493237399633
hsa-miR-130b-3p	T cell receptor signaling pathway	64	110	7949	19747	0.00010637124736645	0.017019399578632
hsa-miR-148a-3p	T cell receptor signaling pathway	55	110	6669	19747	0.000312002122366706	0.0499203395786729
hsa-miR-212-3p	T cell receptor signaling pathway	58	110	6789	19747	5,67E+09	0.00992428623095152
hsa-miR-214-3p	T cell receptor signaling pathway	76	110	10069	19747	8,55E+09	0.0146159220124725
hsa-miR-221-3p	T cell receptor signaling pathway	70	110	9076	19747	0.000138224055083733	0.0246038818049046
hsa-miR-27a-3p	T cell receptor signaling pathway	73	110	9729	19747	0.000210932332820651	0.0375459552420759

hsa-miR-29c-3p	T cell receptor signaling pathway	56	110	6309	19747	2,71E+09	0.00477079759122916
hsa-miR-328-3p	T cell receptor signaling pathway	64	110	7326	19747	5,45E+08	0.000958932236268154
hsa-miR-486-3p	T cell receptor signaling pathway	96	110	11653	19747	8,06E+03	1,55E+05
hsa-miR-532-5p	T cell receptor signaling pathway	66	110	8143	19747	5,35E+09	0.00935919358849223
hsa-miR-92a-3p	T cell receptor signaling pathway	65	110	8069	19747	8,39E+09	0.0146881497840589
hsa-miR-130b-3p	TGF beta signaling pathway	55	86	7949	19747	7,46E+07	0.00132758846439629
hsa-miR-132-3p	TGF beta signaling pathway	48	86	6440	19747	7,56E+08	0.00139043476427043
hsa-miR-212-3p	TGF beta signaling pathway	51	86	6789	19747	1,94E+07	0.000355407516155259
hsa-miR-130b-3p	Thyroid cancer	22	29	7949	19747	0.000105615524961996	0.0168984839939194
hsa-miR-208a-3p	Tight junction	50	132	4498	19747	6,36E+09	0.0112596204388357
hsa-miR-130b-3p	Type II diabetes mellitus	33	49	7949	19747	0.000113455195052502	0.0180393760133479
hsa-miR-221-3p	Type II diabetes mellitus	35	49	9076	19747	0.000269051764693386	0.0476221623507293
hsa-miR-486-3p	Type II diabetes mellitus	42	49	11653	19747	5,06E+09	0.0078985868822121
hsa-miR-181c-5p	Ubiquitin mediated proteolysis	82	134	8188	19747	3,06E+08	0.000581913418613326
hsa-miR-214-3p	Ubiquitin mediated proteolysis	91	134	10069	19747	5,07E+09	0.00888103336142652
hsa-miR-21-5p	Ubiquitin mediated proteolysis	78	134	7952	19747	1,97E+09	0.00360445376288437
hsa-miR-221-3p	Ubiquitin mediated proteolysis	87	134	9076	19747	7,14E+08	0.00134207311818389
hsa-miR-335-5p	Ubiquitin mediated proteolysis	64	134	5890	19747	8,95E+08	0.00159303475447459
hsa-miR-410-3p	Ubiquitin mediated proteolysis	80	134	7692	19747	8,88E+07	0.000170487592541728
hsa-miR-489-3p	Ubiquitin mediated proteolysis	68	134	6768	19747	6,05E+09	0.0112598919347289
hsa-miR-214-3p	Vascular smooth muscle contraction	79	116	10069	19747	0.000133897949251347	0.0224948554742262
hsa-miR-324-3p	Vascular smooth muscle contraction	68	116	8351	19747	0.00027897086972431	0.0449143100256139
hsa-miR-423-5p	Vascular smooth muscle contraction	92	116	12169	19747	3,31E+09	0.00563375299582765
hsa-miR-486-3p	Vascular smooth muscle contraction	92	116	11653	19747	2,77E+08	0.000467974897474186

hsa-miR-532-3p	Vascular smooth muscle contraction	71	116	8784	19747	0.0002066366474092	0.0334751368802904
hsa-miR-660-5p	Vascular smooth muscle contraction	54	116	5834	19747	7,81E+09	0.0133472301484074
hsa-let-7c-5p	VEGF signaling pathway	58	78	10808	19747	0.000273760351696914	0.0451704580299907
hsa-miR-125b-5p	VEGF signaling pathway	48	78	7799	19747	6,65E+09	0.0117122447260963
hsa-miR-212-3p	VEGF signaling pathway	43	78	6789	19747	0.000134184677468026	0.0224088411371603
hsa-miR-324-3p	VEGF signaling pathway	50	78	8351	19747	8,22E+08	0.0138071078477949
hsa-miR-328-3p	VEGF signaling pathway	49	78	7326	19747	3,47E+07	0.000611029340715869
hsa-miR-365a-3p	VEGF signaling pathway	33	78	4746	19747	0.000279474394857759	0.0463927495463881
hsa-miR-423-5p	VEGF signaling pathway	68	78	12169	19747	5,45E+07	0.000101295671716036
hsa-miR-486-3p	VEGF signaling pathway	63	78	11653	19747	3,60E+09	0.00562218231381992
hsa-miR-489-3p	VEGF signaling pathway	43	78	6768	19747	0.000123879682444268	0.0227938615697453
hsa-miR-660-5p	VEGF signaling pathway	39	78	5834	19747	0.000114222940879927	0.0191894540678277
hsa-let-7c-5p	Wnt signaling pathway	114	152	10808	19747	1,76E+07	3,38E+09
hsa-miR-106b-5p	Wnt signaling pathway	94	152	8499	19747	2,19E+07	0.000405198390764093
hsa-miR-122-5p	Wnt signaling pathway	115	152	10891	19747	1,19E+07	2,26E+09
hsa-miR-125b-5p	Wnt signaling pathway	91	152	7799	19747	2,82E+07	5,40E+08
hsa-miR-130b-3p	Wnt signaling pathway	107	152	7949	19747	4,41E+00	8,52E+02
hsa-miR-132-3p	Wnt signaling pathway	75	152	6440	19747	1,31E+09	0.00235891322241269
hsa-miR-145-5p	Wnt signaling pathway	107	152	9602	19747	3,92E+06	7,52E+08
hsa-miR-148a-3p	Wnt signaling pathway	78	152	6669	19747	5,85E+08	0.00106415991644684
hsa-miR-18a-5p	Wnt signaling pathway	97	152	8742	19747	8,87E+07	0.000168441243851599
hsa-miR-346	Wnt signaling pathway	89	152	8052	19747	6,90E+08	0.00127662989585077
hsa-miR-491-5p	Wnt signaling pathway	114	152	10672	19747	7,22E+06	1,37E+09
hsa-miR-181c-5p	Wnt signaling pathway	85	152	8188	19747	0.000217195802317327	0.0388780486148016
hsa-miR-193a-5p	Wnt signaling pathway	118	152	11509	19747	3,77E+07	7,08E+08
hsa-miR-208a-3p	Wnt signaling pathway	54	152	4498	19747	0.000235366730792053	0.040483077696233
hsa-miR-212-3p	Wnt signaling pathway	89	152	6789	19747	8,63E+04	1,65E+07
hsa-miR-214-3p	Wnt signaling pathway	119	152	10069	19747	2,96E+02	5,73E+04
hsa-miR-21-5p	Wnt signaling pathway	90	152	7952	19747	1,74E+08	0.000330851067939793

hsa-miR-221-3p	Wnt signaling pathway	106	152	9076	19747	2,35E+05	4,56E+07
hsa-miR-27a-3p	Wnt signaling pathway	101	152	9729	19747	1,32E+09	0.00244825388667575
hsa-miR-324-3p	Wnt signaling pathway	92	152	8351	19747	4,23E+08	0.00076500949186476
hsa-miR-328-3p	Wnt signaling pathway	81	152	7326	19747	3,30E+09	0.0056096082141783
hsa-miR-410-3p	Wnt signaling pathway	82	152	7692	19747	0.000120398571512339	0.0222737357297827
hsa-miR-423-5p	Wnt signaling pathway	123	152	12169	19747	2,10E+07	3,95E+09
hsa-miR-451a	Wnt signaling pathway	31	152	1963	19747	8,10E+09	0.0151553600884793
hsa-miR-485-3p	Wnt signaling pathway	69	152	6269	19747	0.000281201064881387	0.0494913874191241
hsa-miR-486-3p	Wnt signaling pathway	121	152	11653	19747	5,22E+06	9,45E+08
hsa-miR-532-3p	Wnt signaling pathway	99	152	8784	19747	2,15E+07	3,97E+09
hsa-miR-532-5p	Wnt signaling pathway	88	152	8143	19747	2,39E+09	0.00425560374443665
hsa-miR-636	Wnt signaling pathway	92	152	8717	19747	3,35E+09	0.00616479075209161
hsa-miR-652-3p	Wnt signaling pathway	80	152	6694	19747	1,45E+08	0.000282142216778675
hsa-miR-660-5p	Wnt signaling pathway	70	152	5834	19747	1,17E+09	0.0021112456916117
hsa-miR-92a-3p	Wnt signaling pathway	85	152	8069	19747	0.000120485477474099	0.0208439876030192
hsa-miR-99b-5p	Wnt signaling pathway	39	152	2577	19747	2,01E+09	0.00365668265928942

Cluster B: 51 miRNAs deregulated only in the T1D 2-5y group
