# Supplementary material for: Progression of Type 1 Diabetes: Circulating MicroRNA Expression Profiles Changes from Preclinical to Overt Disease
Source: J Immunol Res. 2022 Jul 19;2022:2734490. doi: 10.1155/2022/2734490 (PMC9325579; doi:10.1155/2022/2734490)
Supplement: Supplementary Materials — Supplementary Table 1S: miRNAs without expression in serum samples. Supplementary Table 2S: pathways related to up-and downregulated miRNAs of cluster A predicted by the miRWalk platform. Supplementary Table 3S: pathways related to upregulated miRNAs of cluster B predicted by the miRWalk platform. Supplementary Table 4S: pathways related to downregulated miRNAs of cluster B predicted by the miRWalk platform. Supplementary Table 5S: most frequent target genes of miRNAs from cluster A of TargetScan. Supplementary Table 6S: most frequent target genes of miRNAs from cluster B by TargetScan. Supplementary Table 7S: ingenuity canonical pathways related to differentially expressed miRNAs' targets. Supplementary Table 8S: reporting guidelines: STREGA. [file 2734490.f1.zip › Suppl 4 miRWalk pathway Cluster B down-regulated miRNAs.pdf]

| Supplementary Table 4S: | Pathways related to down-regulated miRNAs of Cluster B predicted at miRWalk platform | | | | | | BH |
|---|---|---|---|---|---|---|---|
| **miRNA** | **PathName** | **PathFg** | **PathBg** | **GenomeFG** | **GenomeBG** | **Fisher** | 0.0287723918835209 |
| hsa-miR-125a-5p | Adherens junction | 47 | 76 | 8065 | 19747 | 0.000173327661948921 | 8,29E+08 |
| hsa-miR-146a-5p | Adherens junction | 51 | 76 | 7155 | 19747 | 4,29E+06 | 0.000215745806503982 |
| hsa-miR-155-5p | Adherens junction | 44 | 76 | 6132 | 19747 | 1,14E+08 | 0.0153106024351519 |
| hsa-miR-191-5p | Adherens junction | 26 | 76 | 3187 | 19747 | 8,70E+09 | 0.0106677096655376 |
| hsa-miR-197-3p | Adherens junction | 43 | 76 | 6790 | 19747 | 5,96E+09 | 0.000350552438344027 |
| hsa-miR-342-3p | Adherens junction | 52 | 76 | 8200 | 19747 | 1,91E+08 | 8,80E+08 |
| hsa-miR-454-3p | Adherens junction | 53 | 76 | 7671 | 19747 | 4,61E+06 | 0.000686085404804114 |
| hsa-miR-483-5p | Adherens junction | 45 | 76 | 6611 | 19747 | 3,67E+08 | 0.0263876437041632 |
| hsa-miR-518d-3p | Adherens junction | 30 | 76 | 4093 | 19747 | 0.00014419477433969 | 0.0168787215310301 |
| hsa-miR-342-3p | Adipocytokine signaling pathway | 45 | 70 | 8200 | 19747 | 9,99E+09 | 0.00699902995022397 |
| hsa-miR-454-3p | Adipocytokine signaling pathway | 44 | 70 | 7671 | 19747 | 4,02E+09 | 0.00407588144485782 |
| hsa-miR-126-3p | Aldosterone regulated sodium reabsorption | 15 | 42 | 2176 | 19747 | 2,22E+09 | 0.000870534395375211 |
| hsa-miR-126-3p | Apoptosis | 25 | 87 | 2176 | 19747 | 4,66E+07 | 0.00828904100028753 |
| hsa-miR-155-5p | Apoptosis | 45 | 87 | 6132 | 19747 | 4,63E+08 | 0.0528388986939548 |
| hsa-miR-454-3p | Apoptosis | 50 | 87 | 7671 | 19747 | 0.000324165022662299 | 0.00463769744453258 |
| hsa-miR-483-5p | Apoptosis | 48 | 87 | 6611 | 19747 | 2,52E+09 | 3,99E+08 |
| hsa-miR-125a-5p | Axon guidance | 84 | 129 | 8065 | 19747 | 2,09E+06 | 0.00142041038282159 |
| hsa-miR-126-3p | Axon guidance | 32 | 129 | 2176 | 19747 | 7,60E+07 | 6,08E+09 |
| hsa-miR-146a-5p | Axon guidance | 75 | 129 | 7155 | 19747 | 3,17E+05 | 0.00144476125968995 |
| hsa-miR-155-5p | Axon guidance | 64 | 129 | 6132 | 19747 | 7,89E+07 | 0.000221017283884815 |
| hsa-miR-197-3p | Axon guidance | 71 | 129 | 6790 | 19747 | 1,15E+08 | 2,55E+09 |
| hsa-miR-342-3p | Axon guidance | 83 | 129 | 8200 | 19747 | 1,35E+07 | 0.000554817375664576 |
| hsa-miR-454-3p | Axon guidance | 76 | 129 | 7671 | 19747 | 2,97E+08 | 5,02E+07 |
| hsa-miR-483-5p | Axon guidance | 76 | 129 | 6611 | 19747 | 2,61E+05 | 0.000401119047284683 |
| hsa-miR-155-5p | B cell receptor signaling pathway | 43 | 75 | 6132 | 19747 | 2,16E+08 | 0.0270333079384091 |

| | | | | | | | |
|---|---|---|---|---|---|---|---|
| hsa-miR-454-3p | B cell receptor signaling pathway | 45 | 75 | 7671 | 19747 | 0.000164837243526885 | 0.03229955242004 |
| hsa-miR-125a-5p | Basal cell carcinoma | 36 | 55 | 8065 | 19747 | 0.000194575616988193 | 0.019822532193274 |
| hsa-miR-146a-5p | Calcium signaling pathway | 89 | 178 | 7155 | 19747 | 0.00011074040331438 | 0.0463939944379279 |
| hsa-miR-197-3p | Calcium signaling pathway | 84 | 178 | 6790 | 19747 | 0.000266632151942114 | 0.0505596067198286 |
| hsa-miR-342-3p | Calcium signaling pathway | 97 | 178 | 8200 | 19747 | 0.000310181636317967 | 0.0528996821523457 |
| hsa-miR-454-3p | Calcium signaling pathway | 92 | 178 | 7671 | 19747 | 0.000324537927315005 | 0.0170679387849724 |
| hsa-miR-483-5p | Calcium signaling pathway | 84 | 178 | 6611 | 19747 | 9,70E+09 | 0.0470330910750882 |
| hsa-miR-518d-3p | Calcium signaling pathway | 57 | 178 | 4093 | 19747 | 0.000261294950417157 | 1,18E+09 |
| hsa-miR-125a-5p | Cell adhesion molecules CAMs | 85 | 133 | 8065 | 19747 | 6,20E+06 | 0.0184521228928031 |
| hsa-miR-125a-5p | Chronic myeloid leukemia | 47 | 75 | 8065 | 19747 | 0.000109184159129013 | 0.00737747189682358 |
| hsa-miR-126-3p | Chronic myeloid leukemia | 21 | 75 | 2176 | 19747 | 4,03E+09 | 0.000130432778108478 |
| hsa-miR-155-5p | Chronic myeloid leukemia | 44 | 75 | 6132 | 19747 | 6,86E+07 | 0.00419982287562615 |
| hsa-miR-191-5p | Chronic myeloid leukemia | 27 | 75 | 3187 | 19747 | 2,28E+09 | 0.0176373503866759 |
| hsa-miR-197-3p | Chronic myeloid leukemia | 42 | 75 | 6790 | 19747 | 9,96E+09 | 0.012099055168366 |
| hsa-miR-342-3p | Chronic myeloid leukemia | 48 | 75 | 8200 | 19747 | 6,99E+09 | 0.00163195308235571 |
| hsa-miR-454-3p | Chronic myeloid leukemia | 48 | 75 | 7671 | 19747 | 8,92E+08 | 0.00560759404255904 |
| hsa-miR-125a-5p | Colorectal cancer | 54 | 86 | 8065 | 19747 | 3,19E+09 | 0.0209338207814954 |
| hsa-miR-126-3p | Colorectal cancer | 22 | 86 | 2176 | 19747 | 0.000118270173906754 | 0.0125974357347424 |
| hsa-miR-146a-5p | Colorectal cancer | 49 | 86 | 7155 | 19747 | 6,92E+09 | 9,00E+07 |
| hsa-miR-155-5p | Colorectal cancer | 53 | 86 | 6132 | 19747 | 4,66E+05 | 0.00845996812849892 |
| hsa-miR-191-5p | Colorectal cancer | 29 | 86 | 3187 | 19747 | 4,73E+09 | 0.000374736393747273 |
| hsa-miR-197-3p | Colorectal cancer | 51 | 86 | 6790 | 19747 | 1,95E+08 | 0.00150883622587305 |
| hsa-miR-342-3p | Colorectal cancer | 56 | 86 | 8200 | 19747 | 8,34E+08 | 0.0106966429245265 |

| | | | | | | | |
|---|---|---|---|---|---|---|---|
| hsa-miR-374a-5p | Colorectal cancer | 45 | 86 | 6261 | 19747 | 5,63E+09 | 0.00013480071911852 |
| hsa-miR-454-3p | Colorectal cancer | 56 | 86 | 7671 | 19747 | 7,13E+06 | 0.040766154940216 |
| hsa-miR-483-5p | Colorectal cancer | 45 | 86 | 6611 | 19747 | 0.000235642514105295 | 0.0241307332552081 |
| hsa-miR-518d-3p | Colorectal cancer | 33 | 86 | 4093 | 19747 | 0.000131145289430479 | 0.000764040329134395 |
| hsa-miR-125a-5p | Endocytosis | 107 | 187 | 8065 | 19747 | 4,18E+08 | 0.0507787179774768 |
| hsa-miR-126-3p | Endocytosis | 37 | 187 | 2176 | 19747 | 0.000291831712514234 | 0.0121926136724414 |
| hsa-miR-197-3p | Endocytosis | 90 | 187 | 6790 | 19747 | 6,81E+09 | 0.0115024547901004 |
| hsa-miR-342-3p | Endocytosis | 104 | 187 | 8200 | 19747 | 6,65E+09 | 3,26E+07 |
| hsa-miR-454-3p | Endocytosis | 113 | 187 | 7671 | 19747 | 1,70E+05 | 0.0240235666388089 |
| hsa-miR-483-5p | Endocytosis | 87 | 187 | 6611 | 19747 | 0.000137277523650337 | 0.0173775196679673 |
| hsa-miR-125a-5p | Endometrial cancer | 35 | 52 | 8065 | 19747 | 0.00010222070392922 | 0.00100966269842149 |
| hsa-miR-155-5p | Endometrial cancer | 32 | 52 | 6132 | 19747 | 5,49E+08 | 0.0159258938422448 |
| hsa-miR-454-3p | Endometrial cancer | 34 | 52 | 7671 | 19747 | 9,54E+09 | 0.0111773541492556 |
| hsa-miR-454-3p | Epithelial cell signaling in Helicobacter pylori infection | 44 | 71 | 7671 | 19747 | 6,61E+09 | 0.000197036611778636 |
| hsa-miR-125a-5p | ErbB signaling pathway | 59 | 89 | 8065 | 19747 | 1,05E+08 | 0.0354475316166863 |
| hsa-miR-126-3p | ErbB signaling pathway | 22 | 89 | 2176 | 19747 | 0.000202557323523922 | 0.0172756709398722 |
| hsa-miR-146a-5p | ErbB signaling pathway | 50 | 89 | 7155 | 19747 | 9,60E+09 | 0.00285115755691688 |
| hsa-miR-155-5p | ErbB signaling pathway | 47 | 89 | 6132 | 19747 | 1,57E+09 | 0.00646108834376698 |
| hsa-miR-342-3p | ErbB signaling pathway | 56 | 89 | 8200 | 19747 | 3,69E+09 | 0.0299798076807052 |
| hsa-miR-374a-5p | ErbB signaling pathway | 45 | 89 | 6261 | 19747 | 0.000162053014490298 | 0.0230781153667625 |
| hsa-miR-454-3p | ErbB signaling pathway | 52 | 89 | 7671 | 19747 | 0.000139867365859167 | 0.0502541394763599 |
| hsa-miR-483-5p | ErbB signaling pathway | 46 | 89 | 6611 | 19747 | 0.00029217522951372 | 0.000228721087560121 |
| hsa-miR-342-3p | Focal adhesion | 118 | 203 | 8200 | 19747 | 1,24E+08 | 0.0349606093996101 |
| hsa-miR-454-3p | Focal adhesion | 104 | 203 | 7671 | 19747 | 0.000213174447558598 | 0.0247149399693983 |
| hsa-miR-146a-5p | Gap junction | 50 | 90 | 7155 | 19747 | 0.000140425795280672 | 0.0496802571129116 |
| hsa-miR-342-3p | Gap junction | 54 | 90 | 8200 | 19747 | 0.000304786853453446 | 0.00194368223657054 |
| hsa-miR-125a-5p | Glioma | 44 | 65 | 8065 | 19747 | 1,08E+09 | 0.0288081684586405 |

| | | | | | | | |
|---|---|---|---|---|---|---|---|
| hsa-miR-126-3p | Glioma | 18 | 65 | 2176 | 19747 | 0.000163682775333185 | 0.0366454384222118 |
| hsa-miR-146a-5p | Glioma | 38 | 65 | 7155 | 19747 | 0.000213054874547743 | 7,21E+08 |
| hsa-miR-155-5p | Glioma | 40 | 65 | 6132 | 19747 | 3,78E+07 | 0.0212427456563642 |
| hsa-miR-191-5p | Glioma | 23 | 65 | 3187 | 19747 | 0.000122084745151518 | 0.00382574398588028 |
| hsa-miR-197-3p | Glioma | 39 | 65 | 6790 | 19747 | 2,10E+09 | 0.000315280553205102 |
| hsa-miR-342-3p | Glioma | 46 | 65 | 8200 | 19747 | 1,70E+08 | 0.000429832758062455 |
| hsa-miR-454-3p | Glioma | 44 | 65 | 7671 | 19747 | 2,30E+08 | 0.0391841935904168 |
| hsa-miR-483-5p | Glioma | 36 | 65 | 6611 | 19747 | 0.00022649822884634 | 0.02054471809884 |
| hsa-miR-518d-3p | Glioma | 27 | 65 | 4093 | 19747 | 0.000111052530264 | 0.00210519431203355 |
| hsa-miR-197-3p | Glycosphingolipid biosynthesis lacto and neolacto series | 20 | 26 | 6790 | 19747 | 1,13E+09 | 0.00965089844203764 |
| hsa-miR-125a-5p | GnRH signaling pathway | 63 | 105 | 8065 | 19747 | 5,58E+09 | 0.0129286115879751 |
| hsa-miR-191-5p | GnRH signaling pathway | 33 | 105 | 3187 | 19747 | 7,30E+09 | 0.0398836451463501 |
| hsa-miR-483-5p | GnRH signaling pathway | 53 | 105 | 6611 | 19747 | 0.000230541301423989 | 0.0200353092950923 |
| hsa-miR-125a-5p | Hedgehog signaling pathway | 37 | 56 | 8065 | 19747 | 0.000118552126006463 | 0.0142963892095674 |
| hsa-miR-483-5p | Hedgehog signaling pathway | 33 | 56 | 6611 | 19747 | 8,12E+09 | 0.0281900249598004 |
| hsa-miR-191-5p | Inositol phosphate metabolism | 20 | 54 | 3187 | 19747 | 0.000162948121154916 | 0.00298550254803133 |
| hsa-miR-342-3p | Inositol phosphate metabolism | 38 | 54 | 8200 | 19747 | 1,68E+09 | 0.0444433063680304 |
| hsa-miR-483-5p | Inositol phosphate metabolism | 31 | 54 | 6611 | 19747 | 0.000256897724670696 | 0.002038648668654 |
| hsa-miR-125a-5p | Insulin signaling pathway | 82 | 139 | 8065 | 19747 | 1,13E+09 | 0.039447662724898 |
| hsa-miR-126-3p | Insulin signaling pathway | 30 | 139 | 2176 | 19747 | 0.000225415215570846 | 0.0215178394149849 |
| hsa-miR-146a-5p | Insulin signaling pathway | 72 | 139 | 7155 | 19747 | 0.000120886738286432 | 0.0249442161800886 |
| hsa-miR-155-5p | Insulin signaling pathway | 64 | 139 | 6132 | 19747 | 0.000141728501023231 | 0.000769324702867724 |
| hsa-miR-191-5p | Insulin signaling pathway | 44 | 139 | 3187 | 19747 | 4,09E+08 | 0.0064883855263985 |
| hsa-miR-197-3p | Insulin signaling pathway | 71 | 139 | 6790 | 19747 | 3,60E+09 | 5,00E+08 |

| | | | | | | | |
|---|---|---|---|---|---|---|---|
| hsa-miR-342-3p | Insulin signaling pathway | 90 | 139 | 8200 | 19747 | 2,59E+06 | 0.0262549616682783 |
| hsa-miR-374a-5p | Insulin signaling pathway | 65 | 139 | 6261 | 19747 | 0.00014115570789397 | 3,96E+09 |
| hsa-miR-483-5p | Insulin signaling pathway | 76 | 139 | 6611 | 19747 | 2,09E+07 | 0.0093321508366675 |
| hsa-miR-518d-3p | Insulin signaling pathway | 49 | 139 | 4093 | 19747 | 4,99E+09 | 0.00301606534170692 |
| hsa-miR-125a-5p | Leukocyte transendothelial migration | 70 | 116 | 8065 | 19747 | 1,68E+08 | 0.0308023131240282 |
| hsa-miR-146a-5p | Long term potentiation | 41 | 71 | 7155 | 19747 | 0.000177024788069128 | 0.00419120229865921 |
| hsa-miR-191-5p | Long term potentiation | 26 | 71 | 3187 | 19747 | 2,28E+09 | 4,14E+08 |
| hsa-miR-342-3p | Long term potentiation | 51 | 71 | 8200 | 19747 | 2,19E+07 | 0.0506205782623479 |
| hsa-miR-374a-5p | Long term potentiation | 37 | 71 | 6261 | 19747 | 0.000278135045397516 | 0.0277982607115056 |
| hsa-miR-483-5p | Long term potentiation | 39 | 71 | 6611 | 19747 | 0.000159760119031642 | 2,60E+06 |
| hsa-miR-125a-5p | MAPK signaling pathway | 163 | 272 | 8065 | 19747 | 1,34E+04 | 0.000214521988022473 |
| hsa-miR-146a-5p | MAPK signaling pathway | 137 | 272 | 7155 | 19747 | 1,12E+08 | 1,05E+09 |
| hsa-miR-191-5p | MAPK signaling pathway | 79 | 272 | 3187 | 19747 | 5,46E+06 | 0.00844279636970313 |
| hsa-miR-197-3p | MAPK signaling pathway | 125 | 272 | 6790 | 19747 | 4,69E+09 | 0.00556818859338512 |
| hsa-miR-342-3p | MAPK signaling pathway | 146 | 272 | 8200 | 19747 | 3,16E+09 | 0.00436940130041976 |
| hsa-miR-454-3p | MAPK signaling pathway | 139 | 272 | 7671 | 19747 | 2,43E+09 | 0.00617730528707843 |
| hsa-miR-483-5p | MAPK signaling pathway | 123 | 272 | 6611 | 19747 | 3,40E+09 | 0.0261373353994159 |
| hsa-miR-518d-3p | MAPK signaling pathway | 82 | 272 | 4093 | 19747 | 0.000142826969395715 | 0.0142192710352996 |
| hsa-miR-125a-5p | Melanogenesis | 61 | 102 | 8065 | 19747 | 8,32E+09 | 0.0274157959743549 |
| hsa-miR-454-3p | Melanogenesis | 58 | 102 | 7671 | 19747 | 0.000167169487648506 | 0.0154218412105665 |
| hsa-miR-483-5p | Melanogenesis | 53 | 102 | 6611 | 19747 | 8,76E+09 | 0.0120564894033045 |
| hsa-miR-155-5p | Melanoma | 38 | 71 | 6132 | 19747 | 6,77E+09 | 0.0506205782623479 |
| hsa-miR-374a-5p | Melanoma | 37 | 71 | 6261 | 19747 | 0.000278135045397516 | 0.0303873241777422 |
| hsa-miR-125a-5p | mTOR signaling pathway | 35 | 53 | 8065 | 19747 | 0.000183056169745435 | 0.0439131823201756 |
| hsa-miR-342-3p | mTOR signaling pathway | 35 | 53 | 8200 | 19747 | 0.000269406026504145 | 0.00440596043769794 |

| | | | | | | | |
|---|---|---|---|---|---|---|---|
| hsa-miR-125a-5p | Neurotrophin signaling pathway | 76 | 129 | 8065 | 19747 | 2,49E+09 | 0.0140116584705083 |
| hsa-miR-155-5p | Neurotrophin signaling pathway | 61 | 129 | 6132 | 19747 | 7,87E+09 | 0.00142865825925753 |
| hsa-miR-191-5p | Neurotrophin signaling pathway | 41 | 129 | 3187 | 19747 | 7,68E+08 | 0.00540199469544899 |
| hsa-miR-197-3p | Neurotrophin signaling pathway | 67 | 129 | 6790 | 19747 | 2,98E+09 | 9,80E+08 |
| hsa-miR-342-3p | Neurotrophin signaling pathway | 84 | 129 | 8200 | 19747 | 5,11E+06 | 0.0540277607525459 |
| hsa-miR-374a-5p | Neurotrophin signaling pathway | 60 | 129 | 6261 | 19747 | 0.000298495915759922 | 0.00124503362004464 |
| hsa-miR-454-3p | Neurotrophin signaling pathway | 75 | 129 | 7671 | 19747 | 6,77E+05 | 5,02E+07 |
| hsa-miR-483-5p | Neurotrophin signaling pathway | 76 | 129 | 6611 | 19747 | 2,61E+05 | 0.00192230023049846 |
| hsa-miR-125a-5p | Non small cell lung cancer | 38 | 54 | 8065 | 19747 | 1,06E+09 | 0.00771885102134273 |
| hsa-miR-126-3p | Non small cell lung cancer | 17 | 54 | 2176 | 19747 | 4,22E+09 | 0.000897795986535159 |
| hsa-miR-155-5p | Non small cell lung cancer | 33 | 54 | 6132 | 19747 | 4,88E+08 | 0.0281900249598004 |
| hsa-miR-191-5p | Non small cell lung cancer | 20 | 54 | 3187 | 19747 | 0.000162948121154916 | 0.027747078263956 |
| hsa-miR-197-3p | Non small cell lung cancer | 32 | 54 | 6790 | 19747 | 0.000158554732936892 | 0.00298550254803133 |
| hsa-miR-342-3p | Non small cell lung cancer | 38 | 54 | 8200 | 19747 | 1,68E+09 | 0.00563164684842405 |
| hsa-miR-454-3p | Non small cell lung cancer | 36 | 54 | 7671 | 19747 | 3,18E+09 | 0.00256463426046472 |
| hsa-miR-518d-3p | Oocyte meiosis | 43 | 112 | 4093 | 19747 | 1,34E+09 | 3,01E+08 |
| hsa-miR-125a-5p | Pancreatic cancer | 53 | 75 | 8065 | 19747 | 1,59E+07 | 0.00159563565610004 |
| hsa-miR-146a-5p | Pancreatic cancer | 46 | 75 | 7155 | 19747 | 8,40E+07 | 0.00136613771678651 |
| hsa-miR-191-5p | Pancreatic cancer | 28 | 75 | 3187 | 19747 | 7,31E+08 | 0.00262373608666599 |
| hsa-miR-197-3p | Pancreatic cancer | 44 | 75 | 6790 | 19747 | 1,43E+09 | 5,57E+09 |

| | | | | | | | |
|---|---|---|---|---|---|---|---|
| hsa-miR-454-3p | Pancreatic cancer | 51 | 75 | 7671 | 19747 | 2,93E+07 | 1,38E+08 |
| hsa-miR-125a-5p | Pathways in cancer | 186 | 330 | 8065 | 19747 | 7,21E+04 | 0.0214387669361645 |
| hsa-miR-126-3p | Pathways in cancer | 59 | 330 | 2176 | 19747 | 0.000121122977040478 | 1,54E+08 |
| hsa-miR-146a-5p | Pathways in cancer | 170 | 330 | 7155 | 19747 | 7,93E+05 | 7,45E+06 |
| hsa-miR-155-5p | Pathways in cancer | 156 | 330 | 6132 | 19747 | 3,84E+03 | 0.000125761220978404 |
| hsa-miR-191-5p | Pathways in cancer | 88 | 330 | 3187 | 19747 | 6,55E+07 | 4,65E+09 |
| hsa-miR-197-3p | Pathways in cancer | 158 | 330 | 6790 | 19747 | 2,40E+07 | 2,02E+09 |
| hsa-miR-342-3p | Pathways in cancer | 184 | 330 | 8200 | 19747 | 1,06E+07 | 0.0116805960557104 |
| hsa-miR-374a-5p | Pathways in cancer | 138 | 330 | 6261 | 19747 | 6,18E+09 | 1,07E+07 |
| hsa-miR-454-3p | Pathways in cancer | 183 | 330 | 7671 | 19747 | 5,57E+04 | 4,03E+09 |
| hsa-miR-483-5p | Pathways in cancer | 155 | 330 | 6611 | 19747 | 2,13E+07 | 0.0014380279214663 |
| hsa-miR-518d-3p | Pathways in cancer | 102 | 330 | 4093 | 19747 | 7,49E+08 | 0.0405772903291612 |
| hsa-miR-191-5p | Phosphatidylinositol signaling system | 25 | 76 | 3187 | 19747 | 0.000238689943112713 | 0.000350552438344027 |
| hsa-miR-342-3p | Phosphatidylinositol signaling system | 52 | 76 | 8200 | 19747 | 1,91E+08 | 0.0412204974035423 |
| hsa-miR-454-3p | Phosphatidylinositol signaling system | 45 | 76 | 7671 | 19747 | 0.000252886487138296 | 0.000227122389365353 |
| hsa-miR-483-5p | Phosphatidylinositol signaling system | 46 | 76 | 6611 | 19747 | 1,21E+08 | 0.0451722421136335 |
| hsa-miR-125a-5p | Prostate cancer | 53 | 89 | 8065 | 19747 | 0.000275440500692887 | 0.00110169881612874 |
| hsa-miR-155-5p | Prostate cancer | 48 | 89 | 6132 | 19747 | 5,99E+08 | 0.0415067715162865 |
| hsa-miR-191-5p | Prostate cancer | 28 | 89 | 3187 | 19747 | 0.000244157479507567 | 0.00438472168092128 |
| hsa-miR-454-3p | Prostate cancer | 54 | 89 | 7671 | 19747 | 2,45E+09 | 0.000113759773655651 |
| hsa-miR-125a-5p | Regulation of actin cytoskeleton | 122 | 212 | 8065 | 19747 | 6,08E+07 | 0.00376550149645128 |
| hsa-miR-342-3p | Regulation of actin cytoskeleton | 118 | 212 | 8200 | 19747 | 2,13E+08 | 0.0303275201995215 |

| | | | | | | | |
|---|---|---|---|---|---|---|---|
| hsa-miR-155-5p | Renal cell carcinoma | 37 | 71 | 6132 | 19747 | 0.000174296093100698 | 4,14E+08 |
| hsa-miR-342-3p | Renal cell carcinoma | 51 | 71 | 8200 | 19747 | 2,19E+07 | 0.027868911909151 |
| hsa-miR-454-3p | Renal cell carcinoma | 43 | 71 | 7671 | 19747 | 0.000169932389689945 | 0.00676424821180159 |
| hsa-miR-155-5p | Small cell lung cancer | 44 | 84 | 6132 | 19747 | 3,76E+09 | 0.0175311753135427 |
| hsa-miR-197-3p | Small cell lung cancer | 46 | 84 | 6790 | 19747 | 9,90E+09 | 0.0372711156929396 |
| hsa-miR-454-3p | Small cell lung cancer | 49 | 84 | 7671 | 19747 | 0.000227262900566705 | 0.0578865731632053 |
| hsa-miR-125a-5p | T cell receptor signaling pathway | 63 | 110 | 8065 | 19747 | 0.000357324525698798 | 0.00549246475699916 |
| hsa-miR-146a-5p | T cell receptor signaling pathway | 61 | 110 | 7155 | 19747 | 2,95E+08 | 0.00461301328049845 |
| hsa-miR-197-3p | T cell receptor signaling pathway | 59 | 110 | 6790 | 19747 | 2,55E+09 | 0.00239140557089447 |
| hsa-miR-454-3p | T cell receptor signaling pathway | 65 | 110 | 7671 | 19747 | 1,31E+09 | 0.00955630586358921 |
| hsa-miR-342-3p | TGF beta signaling pathway | 54 | 86 | 8200 | 19747 | 5,49E+09 | 0.00155477706695217 |
| hsa-miR-191-5p | Type II diabetes mellitus | 21 | 49 | 3187 | 19747 | 8,36E+08 | 0.0516925795335621 |
| hsa-miR-374a-5p | Ubiquitin mediated proteolysis | 62 | 134 | 6261 | 19747 | 0.000285594362063879 | 0.0441606610270351 |
| hsa-miR-191-5p | Vascular smooth muscle contraction | 34 | 116 | 3187 | 19747 | 0.000259768594276677 | 0.000648196602171507 |
| hsa-miR-125a-5p | VEGF signaling pathway | 52 | 78 | 8065 | 19747 | 3,52E+08 | 0.009697621173691 |
| hsa-miR-197-3p | VEGF signaling pathway | 44 | 78 | 6790 | 19747 | 5,42E+09 | 1,60E+08 |
| hsa-miR-125a-5p | Wnt signaling pathway | 97 | 152 | 8065 | 19747 | 8,34E+05 | 0.00470139772170649 |
| hsa-miR-146a-5p | Wnt signaling pathway | 80 | 152 | 7155 | 19747 | 2,51E+09 | 0.00153387586565945 |
| hsa-miR-155-5p | Wnt signaling pathway | 73 | 152 | 6132 | 19747 | 8,38E+08 | 0.00031490211266938 |
| hsa-miR-191-5p | Wnt signaling pathway | 48 | 152 | 3187 | 19747 | 1,65E+08 | 0.00227716334267629 |

| | | | | | | | |
|---|---|---|---|---|---|---|---|
| hsa-miR-197-3p | Wnt signaling pathway | 78 | 152 | 6790 | 19747 | 1,23E+09 | 0.000675877505055815 |
| hsa-miR-342-3p | Wnt signaling pathway | 91 | 152 | 8200 | 19747 | 3,67E+08 | 8,54E+05 |
| hsa-miR-454-3p | Wnt signaling pathway | 99 | 152 | 7671 | 19747 | 4,40E+03 | 1,21E+09 |
| hsa-miR-483-5p | Wnt signaling pathway | 83 | 152 | 6611 | 19747 | 6,36E+06 | 0.00315084208751404 |
| hsa-miR-518d-3p | Wnt signaling pathway | 54 | 152 | 4093 | 19747 | 1,65E+09 | |

Cluster B: 51 miRNAs deregulated only in the T1D 2-5y group
