# Supplementary material for: Progression of Type 1 Diabetes: Circulating MicroRNA Expression Profiles Changes from Preclinical to Overt Disease
Source: J Immunol Res. 2022 Jul 19;2022:2734490. doi: 10.1155/2022/2734490 (PMC9325579; doi:10.1155/2022/2734490)
Supplement: Supplementary Materials — Supplementary Table 1S: miRNAs without expression in serum samples. Supplementary Table 2S: pathways related to up-and downregulated miRNAs of cluster A predicted by the miRWalk platform. Supplementary Table 3S: pathways related to upregulated miRNAs of cluster B predicted by the miRWalk platform. Supplementary Table 4S: pathways related to downregulated miRNAs of cluster B predicted by the miRWalk platform. Supplementary Table 5S: most frequent target genes of miRNAs from cluster A of TargetScan. Supplementary Table 6S: most frequent target genes of miRNAs from cluster B by TargetScan. Supplementary Table 7S: ingenuity canonical pathways related to differentially expressed miRNAs' targets. Supplementary Table 8S: reporting guidelines: STREGA. [file 2734490.f1.zip › Suppl 5 Target Genes more frequent from miRNAs of Cluster A.pdf]

Supplementary Table 5S: Most frequent Target Genes of miRNAs from Cluster A by TargetScan

miRNAs Cluster A	Target genes	Gene names	Number of miRNAs	%
**up regulated**	BEND4	BEN domain containing 4	5	0,42
	CBX5	chromobox homolog 5	5	0,42
	DYRK2	dual-specificity tyrosine-(Y)-phosphorylation regulated kinase 2	4	0,33
	FZD5	frizzled family receptor 5	5	0,42
	GATA6	GATA binding protein 6	4	0,33
	GPCPD1	glycerophosphocholine phosphodiesterase GDE1 homolog (S. cerevisiae)	4	0,33
	HIPK1	homeodomain interacting protein kinase 1	4	0,33
	KCNC3	potassium voltage-gated channel, Shaw-related subfamily, member 3	4	0,33
	LCOR	ligand dependent nuclear receptor corepressor	4	0,33
	PALM2	paralemmin 2	4	0,33
	PPARGC1B	peroxisome proliferator-activated receptor gamma, coactivator 1 beta	4	0,33
	PPP1CB	protein phosphatase 1, catalytic subunit, beta isozyme	4	0,33
	RASL10B	RAS-like, family 10, member B	4	0,33
	SPRY3	sprouty homolog 3 (Drosophila)	4	0,33
	ST5	suppression of tumorigenicity 5	4	0,33
	STRN	striatin, calmodulin binding protein	4	0,33
	THRA	thyroid hormone receptor, alpha	4	0,33
	ZBTB20	zinc finger and BTB domain containing 20	4	0,33
	ZBTB7A	zinc finger and BTB domain containing 7A	4	0,33
	ZCCHC14	zinc finger, CCHC domain containing 14	4	0,33
**downregulated**	BRWD1	bromodomain and WD repeat domain containing 1	3	0,50
	C16orf52	chromosome 16 open reading frame 52	3	0,50
	PLAG1	pleiomorphic adenoma gene 1	3	0,50
	ZBTB10	zinc finger and BTB domain containing 10	3	0,50

Cluster A: 18 miRNAs consistently deregulated in AbP or recent T1D groups (13 of them being also deregulated in T1D 2-5y)
