# Supplementary material for: Progression of Type 1 Diabetes: Circulating MicroRNA Expression Profiles Changes from Preclinical to Overt Disease
Source: J Immunol Res. 2022 Jul 19;2022:2734490. doi: 10.1155/2022/2734490 (PMC9325579; doi:10.1155/2022/2734490)
Supplement: Supplementary Materials — Supplementary Table 1S: miRNAs without expression in serum samples. Supplementary Table 2S: pathways related to up-and downregulated miRNAs of cluster A predicted by the miRWalk platform. Supplementary Table 3S: pathways related to upregulated miRNAs of cluster B predicted by the miRWalk platform. Supplementary Table 4S: pathways related to downregulated miRNAs of cluster B predicted by the miRWalk platform. Supplementary Table 5S: most frequent target genes of miRNAs from cluster A of TargetScan. Supplementary Table 6S: most frequent target genes of miRNAs from cluster B by TargetScan. Supplementary Table 7S: ingenuity canonical pathways related to differentially expressed miRNAs' targets. Supplementary Table 8S: reporting guidelines: STREGA. [file 2734490.f1.zip › Suppl 6 Target Genes more frequent from miRNAs of Cluster B.pdf]

Supplementary Table 6S:	Most frequent Target Genes of miRNAs from Cluster B by TargetScan			
Cluster B miRNAs	**Target gene**	**Gene Names**	**number of miRNAs**	**%**
**Up regulated**	NFIB	nuclear factor I/B	16	41,03%
	NAA50	N(alpha)-acetyltransferase 50, NatE catalytic subunit	14	35,90%
	MMP16	matrix metallopeptidase 16 (membrane-inserted)	12	30,77%
	NUFIP2	nuclear fragile X mental retardation protein interacting protein 2	12	30,77%
	KCNJ6	potassium inwardly-rectifying channel, subfamily J, member 6	11	28,21%
	NFAT5	nuclear factor of activated T-cells 5, tonicity-responsive	11	28,21%
	OTUD7B	OTU domain containing 7B	11	28,21%
	SMARCD1	SWI/SNF related, matrix associated, actin dependent regulator of chromatin, subfamily d, member 1	11	28,21%
	AGO1	argonaute RISC catalytic component 1	10	25,64%
	KLF12	Kruppel-like factor 12	10	25,64%
	KLF7	Kruppel-like factor 7 (ubiquitous)	10	25,64%
	KLHL15	kelch-like family member 15	10	25,64%
	KPNA6	karyopherin alpha 6 (importin alpha 7)	10	25,64%
	MAPK1	mitogen-activated protein kinase 1	10	25,64%
	MECP2	methyl CpG binding protein 2 (Rett syndrome)	10	25,64%
	MEF2D	myocyte enhancer factor 2D	10	25,64%
	PRICKLE2	prickle homolog 2 (Drosophila)	10	25,64%
	SMAD2	SMAD family member 2	10	25,64%
	ACVR2B	activin A receptor, type IIB	9	23,08%
	AKIRIN1	akirin 1	9	23,08%
	ANKFY1	ankyrin repeat and FYVE domain containing 1	9	23,08%
	GXYLT1	glucoside xylosyltransferase 1	9	23,08%
	IGF1	insulin-like growth factor 1 (somatomedin C)	9	23,08%
	ITGA9	integrin, alpha 9	9	23,08%
	KPNA4	karyopherin alpha 4 (importin alpha 3)	9	23,08%
	PEG10	paternally expressed 10	9	23,08%
	PHF15	PHD finger protein 15	9	23,08%
	RAD51L3-RFFL	Uncharacterized protein	9	23,08%
	WNT2B	wingless-type MMTV integration site family, member 2B	9	23,08%
	ZBTB20	zinc finger and BTB domain containing 20	9	23,08%
	ACVR1C	activin A receptor, type IC	8	20,51%
	AKT3	v-akt murine thymoma viral oncogene homolog 3	8	20,51%
	ARHGAP32	Rho GTPase activating protein 32	8	20,51%
	ATXN1	ataxin 1	8	20,51%
	HIC2	hypermethylated in cancer 2	8	20,51%
	HNRNPA3	heterogeneous nuclear ribonucleoprotein A3	8	20,51%
	INO80	INO80 complex subunit	8	20,51%
	KDM5B	lysine (K)-specific demethylase 5B	8	20,51%
	KIAA0247	KIAA0247	8	20,51%
	KIAA1549	KIAA1549	8	20,51%
	KIRREL	kin of IRRE like (Drosophila)	8	20,51%
	KLF3	Kruppel-like factor 3 (basic)	8	20,51%
	LMTK2	lemur tyrosine kinase 2	8	20,51%
	LPP	LIM domain containing preferred translocation partner in lipoma	8	20,51%
	LRRC40	leucine rich repeat containing 40	8	20,51%
	MAP3K9	mitogen-activated protein kinase kinase kinase 9	8	20,51%

	MDGA1	MAM domain containing glycosylphosphatidylinositol anchor 1	8	20,51%
	MTF1	metal-regulatory transcription factor 1	8	20,51%
	MYO1D	myosin ID	8	20,51%
	NABP1	nucleic acid binding protein 1	8	20,51%
	NUDT3	nudix (nucleoside diphosphate linked moiety X)-type motif 3	8	20,51%
	ONECUT2	one cut homeobox 2	8	20,51%
	PKIA	protein kinase (cAMP-dependent, catalytic) inhibitor alpha	8	20,51%
	PLEKHA6	pleckstrin homology domain containing, family A member 6	8	20,51%
	PPARGC1B	peroxisome proliferator-activated receptor gamma, coactivator 1 beta	8	20,51%
	PURB	purine-rich element binding protein B	8	20,51%
	RFFL	ring finger and FYVE-like domain containing E3 ubiquitin protein ligase	8	20,51%
	RYBP	RING1 and YY1 binding protein	8	20,51%
	SRSF1	serine/arginine-rich splicing factor 1	8	20,51%
	SZRD1	SUZ RNA binding domain containing 1	8	20,51%
	UBE2D3	ubiquitin-conjugating enzyme E2D 3	8	20,51%
	ZNF704	zinc finger protein 704	8	20,51%
**Down regulated**	ABL2	c-abl oncogene 2, non-receptor tyrosine kinase	5	45,45%
	CREB1	cAMP responsive element binding protein 1	5	45,45%
	FBXO28	F-box protein 28	5	45,45%
	FOSL2	FOS-like antigen 2	5	45,45%
	PDE7A	phosphodiesterase 7A	5	45,45%
	QKI	QKI, KH domain containing, RNA binding	5	45,45%
	RPS6KA3	ribosomal protein S6 kinase, 90kDa, polypeptide 3	5	45,45%
	AAK1	AP2 associated kinase 1	4	36,36%
	ABCB11	ATP-binding cassette, sub-family B (MDR/TAP), member 11	4	36,36%
	ANKRD12	ankyrin repeat domain 12	4	36,36%
	ASXL2	additional sex combs like 2 (Drosophila)	4	36,36%
	ATXN1L	ataxin 1-like	4	36,36%
	BCAT1	branched chain amino-acid transaminase 1, cytosolic	4	36,36%
	BMPR2	bone morphogenetic protein receptor, type II (serine/threonine kinase)	4	36,36%
	CDC42BPG	CDC42 binding protein kinase gamma (DMPK-like)	4	36,36%
	CDS1	CDP-diacylglycerol synthase (phosphatidate cytidylyltransferase) 1	4	36,36%
	CNTNAP5	contactin associated protein-like 5	4	36,36%
	CXorf23	chromosome X open reading frame 23	4	36,36%
	DNAL1	dynein, axonemal, light chain 1	4	36,36%
	DSEL	dermatan sulfate epimerase-like	4	36,36%
	EDA	ectodysplasin A	4	36,36%
	EHF	ets homologous factor	4	36,36%
	FAHD2A	fumarylacetoacetate hydrolase domain containing 2A	4	36,36%
	FZD5	frizzled family receptor 5	4	36,36%
	GMEB1	glucocorticoid modulatory element binding protein 1	4	36,36%
	GPT2	glutamic pyruvate transaminase (alanine aminotransferase) 2	4	36,36%
	GRK5	G protein-coupled receptor kinase 5	4	36,36%
	HIC2	hypermethylated in cancer 2	4	36,36%
	HRK	harakiri, BCL2 interacting protein (contains only BH3 domain)	4	36,36%
	HUNK	hormonally up-regulated Neu-associated kinase	4	36,36%
	IKZF3	IKAROS family zinc finger 3 (Aiolos)	4	36,36%

JMY	junction mediating and regulatory protein, p53 cofactor	4	36,36%
KCMF1	potassium channel modulatory factor 1	4	36,36%
KCNA1	potassium voltage-gated channel, shaker-related subfamily, member 1 (episodic ataxia with myokymia)	4	36,36%
KIAA1468	KIAA1468	4	36,36%
KIF26B	kinesin family member 26B	4	36,36%
KLF7	Kruppel-like factor 7 (ubiquitous)	4	36,36%
MAPK1	mitogen-activated protein kinase 1	4	36,36%
METTL21A	methyltransferase like 21A	4	36,36%
MSI2	musashi RNA-binding protein 2	4	36,36%
NABP1	nucleic acid binding protein 1	4	36,36%
NACC2	NACC family member 2, BEN and BTB (POZ) domain containing	4	36,36%
NFIB	nuclear factor I/B	4	36,36%
NFIC	nuclear factor I/C (CCAAT-binding transcription factor)	4	36,36%
PHC2	polyhomeotic homolog 2 (Drosophila)	4	36,36%
PIK3CA	phosphatidylinositol-4,5-bisphosphate 3-kinase, catalytic subunit alpha	4	36,36%
PLCE1	phospholipase C, epsilon 1	4	36,36%
PLD5	phospholipase D family, member 5	4	36,36%
PLEKHM3	pleckstrin homology domain containing, family M, member 3	4	36,36%
POLH	polymerase (DNA directed), eta	4	36,36%
PPP1R3B	protein phosphatase 1, regulatory subunit 3B	4	36,36%
PROX1	prospero homeobox 1	4	36,36%
RAPGEF4	Rap guanine nucleotide exchange factor (GEF) 4	4	36,36%
RFX3	regulatory factor X, 3 (influences HLA class II expression)	4	36,36%
RNF11	ring finger protein 11	4	36,36%
SATB2	SATB homeobox 2	4	36,36%
SCN2B	sodium channel, voltage-gated, type II, beta subunit	4	36,36%
SEPT11	septin 11	4	36,36%
SGIP1	SH3-domain GRB2-like (endophilin) interacting protein 1	4	36,36%
SKI	v-ski avian sarcoma viral oncogene homolog	4	36,36%
SMTNL2	smoothelin-like 2	4	36,36%
SOCS6	suppressor of cytokine signaling 6	4	36,36%
SORT1	sortilin 1	4	36,36%
SOX11	SRY (sex determining region Y)-box 11	4	36,36%
SP1	Sp1 transcription factor	4	36,36%
STRN	striatin, calmodulin binding protein	4	36,36%
TCF4	transcription factor 4	4	36,36%
TEAD3	TEA domain family member 3	4	36,36%
TGOLN2	trans-golgi network protein 2	4	36,36%
TIA1	TIA1 cytotoxic granule-associated RNA binding protein	4	36,36%
TMEM120B	transmembrane protein 120B	4	36,36%
TTBK2	tau tubulin kinase 2	4	36,36%
UBE2W	ubiquitin-conjugating enzyme E2W (putative)	4	36,36%
UBN2	ubinuclein 2	4	36,36%
UGCG	UDP-glucose ceramide glucosyltransferase	4	36,36%
WDR37	WD repeat domain 37	4	36,36%
ZC3H12B	zinc finger CCCH-type containing 12B	4	36,36%
ZCCHC24	zinc finger, CCHC domain containing 24	4	36,36%
ZDHHC9	zinc finger, DHHC-type containing 9	4	36,36%

ZNF652	zinc finger protein 652	4	36,36%

Cluster B: 51 miRNAs deregulated only in the T1D 2-5y group
