# Supplementary material for: Progression of Type 1 Diabetes: Circulating MicroRNA Expression Profiles Changes from Preclinical to Overt Disease
Source: J Immunol Res. 2022 Jul 19;2022:2734490. doi: 10.1155/2022/2734490 (PMC9325579; doi:10.1155/2022/2734490)
Supplement: Supplementary Materials — Supplementary Table 1S: miRNAs without expression in serum samples. Supplementary Table 2S: pathways related to up-and downregulated miRNAs of cluster A predicted by the miRWalk platform. Supplementary Table 3S: pathways related to upregulated miRNAs of cluster B predicted by the miRWalk platform. Supplementary Table 4S: pathways related to downregulated miRNAs of cluster B predicted by the miRWalk platform. Supplementary Table 5S: most frequent target genes of miRNAs from cluster A of TargetScan. Supplementary Table 6S: most frequent target genes of miRNAs from cluster B by TargetScan. Supplementary Table 7S: ingenuity canonical pathways related to differentially expressed miRNAs' targets. Supplementary Table 8S: reporting guidelines: STREGA. [file 2734490.f1.zip › Suppl 7 Target genes of miRNAs predicted by IPA.pdf]

Supplementary Table 7S
GROUP
Antibody positive without diabetes

Canonical pthways

#	Ingenuity Canonical Pathways	Overlaps with dataset	No overlap with dataset	-log(p-value)	Ratio	Molecules
1	PTEN Signaling	22/126 (17%)	104/126 (83%)	16,30	0,18	BCL2,BMPR2,CCND1,CDKN1A,CDKN1B,EGFR,FASLG,FGFR1,FGFR3,GRB2,GSK3B,IGF1R,IGF2R,ITGA2,KRAS,MAP2K1,MAPK3,PIK3R1,PTEN,RAF1,RELA,TGFBR2
2	Aryl Hydrocarbon Receptor Signaling	22/143 (15%)	121/143 (85%)	15,10	0,15	APAF1,BAX,CCND1,CCND2,CCND3,CCNE1,CDK6,CDKN1A,CDKN1B,CHEK1,E2F1,ESR1,FAS,FASLG,GSTM4,HSP90B1,JUN,MAPK3,NFIA,NFIB,RELA,TNF
3	Epithelial Mesenchymal Transition By Growth Factors Pathway	24/188 (13%)	164/188 (87%)	14,50	0,13	EGFR,ETS1,FASLG,FGF16,FGF2,FGF7,FGFR1,FGFR3,GRB2,GSK3B,IL6R,JUN,KRAS,LATS2,MAP2K1,MAP2K4,MAPK3,MTOR,PIK3R1,RAF1,RELA,TGFBR2,TNF,TNFSF9
4	STAT3 Pathway	20/135 (15%)	115/135 (85%)	13,40	0,15	BCL2,BMPR2,CDC25A,CDKN1A,EGFR,FGF2,FGFR1,FGFR3,IGF1,IGF1R,IGF2R,IL6R,KRAS,MAP2K1,MAP2K4,MAPK3,RAF1,SOCS5,TGFBR2,VEGFA
5	Senescence Pathway	27/275 (10%)	248/275 (90%)	13,40	0,10	ASXL2,BMPR2,CCND1,CDC25A,CDK6,CDKN1A,CDKN1B,CHEK1,DMTF1,E2F1,E2F3,EIF4E,ETS1,JUN,KRAS,MAP2K1,MAP2K4,MAPK3,MTOR,NF1,PIK3R1,PPP2R5C,PTEN,RAF1,RPS6KA5,SQSTM1,TGFBR2
6	Acute Phase Response Signaling	15/179 (8%)	164/179 (92%)	6,79	0,08	F2,GRB2,HMOX1,IL6R,JUN,KRAS,MAP2K1,MAP2K4,MAPK3,MTOR,PIK3R1,RAF1,RELA,SOCS5,TNF
7	Cyclins and Cell Cycle Regulation	14/81 (17%)	67/81 (83%)	10,50	0,17	CCND1,CCND2,CCND3,CCNE1,CDC25A,CDK6,CDKN1A,CDKN1B,E2F1,E2F3,GSK3B,PPP2R5C,RAF1,WEE1
8	NRF2-mediated Oxidative Stress Response	15/189 (8%)	174/189 (92%)	6,48	0,08	ACTA2,DNAJB4,GSK3B,GSTM4,HERPUD1,HMOX1,JUN,KRAS,MAP2K1,MAP2K4,MAPK3,PIK3R1,RAF1,SOD3,SQSTM1
9	Estrogen Receptor Signaling	25/328 (8%)	303/328 (92%)	10,40	0,08	BCL2,CCND1,CDKN1A,CFL2,EGFR,EIF4E,ESR1,GRB2,GSK3B,HSP90B1,IGF1,IGF1R,IGF2R,JUN,KRAS,MAP2K1,MAPK3,MMP14,MTOR,PIK3R1,PRKACB,PTEN,RAF1,RELA,VEGFA
10	IL-7 Signaling Pathway	13/78 (17%)	65/78 (83%)	9,60	0,17	BAX,BCL2,CCND1,CCND3,CDC25A,CDKN1B,GRB2,GSK3B,IFNG,JUN,MAPK3,MCL1,PIK3R1
11	IL-8 Signaling	19/200 (10%)	181/200 (91%)	9,35	0,10	BAX,BCL2,CCND1,CCND2,CCND3,EGFR,HMOX1,JUN,KRAS,MAP2K1,MAP2K4,MAPK3,MTOR,PIK3R1,PTGS2,RAF1,RELA,RHOT1,VEGFA
12	Cell Cycle: G1/S Checkpoint Regulation	12/67 (18%)	55/67 (82%)	9,29	0,18	BMI1,CCND1,CCND2,CCND3,CCNE1,CDC25A,CDK6,CDKN1A,CDKN1B,E2F1,E2F3,GSK3B
13	Neuregulin Signaling	13/96 (14%)	83/96 (86%)	8,45	0,14	CDKN1B,EGFR,ERBB4,GRB2,HSP90B1,ITGA2,KRAS,MAP2K1,MAPK3,MTOR,PIK3R1,PTEN,RAF1
14	Estrogen-mediated S-phase Entry	8/26 (31%)	18/26 (69%)	8,38	0,31	CCND1,CCNE1,CDC25A,CDKN1A,CDKN1B,E2F1,E2F3,ESR1
15	Glucocorticoid Receptor Signaling	23/336 (7%)	313/336 (93%)	8,38	0,07	BCL2,CDKN1A,DUSP1,ESR1,GRB2,GTF2H1,HSP90B1,HSPA14,HSPA1A/HSPA1B,IFNG,JUN,KRAS,MAP2K1,MAP2K4,MAPK3,PIK3R1,PRKACB,PTGS2,RAF1,RELA,TGFBR2,TNF,TRA
16	JAK/Stat Signaling	12/80 (15%)	68/80 (85%)	8,36	0,15	CCKBR,CDKN1A,GRB2,JUN,KRAS,MAP2K1,MAPK3,MTOR,PIK3R1,RAF1,RELA,SOCS5
17	Role of PKR in Interferon Induction and Antiviral Response	14/117 (12%)	103/117 (88%)	8,34	0,12	APAF1,BAX,E2F1,FAS,FASLG,HSP90B1,HSPA14,HSPA1A/HSPA1B,IFNG,JUN,MAP2K4,MAPK3,RELA,TNF
18	ErbB2-ErbB3 Signaling	11/65 (17%)	54/65 (83%)	8,29	0,17	CCND1,CDKN1B,GRB2,GSK3B,JUN,KRAS,MAP2K1,MAPK3,PIK3R1,PTEN,RAF1
19	Apoptosis Signaling	13/99 (13%)	86/99 (87%)	8,28	0,13	APAF1,BAX,BCL2,FAS,FASLG,KRAS,MAP2K1,MAP2K4,MAPK3,MCL1,RAF1,RELA,TNF
20	MAPK Signaling	10/52 (19%)	42/52 (81%)	8,15	0,19	EGFR,EIF4E,H3-3A/H3-3B,JUN,MAP2K1,MAP2K4,MAPK3,MTOR,PIK3R1,RPS6KA5
21	ILK Signaling	17/190 (9%)	173/190 (91%)	8,04	0,09	ACTA2,CCND1,CFL2,GSK3B,JUN,MAP2K4,MAPK3,MTOR,PIK3R1,PPP2R5C,PTEN,PTGS2,RELA,RHOT1,RPS6KA5,TNF,VEGFA
22	IL-6 Signaling	14/125 (11%)	111/125 (89%)	7,96	0,11	ABCB1,GRB2,IL6R,JUN,KRAS,MAP2K1,MAP2K4,MAPK3,MCL1,PIK3R1,RAF1,RELA,TNF,VEGFA
23	EGF Signaling	9/55 (16%)	46/55 (84%)	6,75	0,16	EGFR,GRB2,JUN,MAP2K1,MAP2K4,MAPK3,MTOR,PIK3R1,RAF1
24	HGF Signaling	13/111 (12%)	98/111 (88%)	7,67	0,12	CCND1,CDKN1A,ETS1,GRB2,ITGA2,JUN,KRAS,MAP2K1,MAP2K4,MAPK3,PIK3R1,PTGS2,RAF1
25	NF-κB Signaling	16/179 (9%)	163/179 (91%)	7,59	0,09	BMPR2,EGFR,FGFR1,FGFR3,GSK3B,IGF1R,IGF2R,KRAS,PELI1,PIK3R1,PRKACB,RAF1,RELA,TGFBR2,TNF,TRA
26	ErbB Signaling	12/94 (13%)	82/94 (87%)	7,55	0,13	EGFR,ERBB4,GRB2,GSK3B,JUN,KRAS,MAP2K1,MAP2K4,MAPK3,MTOR,PIK3R1,RAF1
27	Neurotrophin/TRK Signaling	11/76 (14%)	65/76 (86%)	7,55	0,15	BDNF,GRB2,JUN,KRAS,MAP2K1,MAP2K4,MAPK3,PIK3R1,RAF1,SPRY1,SPRY2
28	Role of NANOG in Mammalian Embryonic Stem Cell Pluripotency	13/119 (11%)	106/119 (89%)	7,31	0,11	BMPR2,CDX2,GATA6,GRB2,GSK3B,KRAS,MAP2K1,MAPK3,PIK3R1,RAF1,SMO,TCL1A,WNT3A
29	FGF Signaling	11/84 (13%)	73/84 (87%)	7,09	0,13	FGF16,FGF2,FGF7,FGFR1,FGFR3,GRB2,MAP2K1,MAPK3,PIK3R1,RAF1,RPS6KA5
30	IGF-1 Signaling	12/104 (12%)	92/104 (88%)	7,05	0,12	GRB10,GRB2,IGF1,IGF1R,JUN,KRAS,MAP2K1,MAPK3,PIK3R1,PRKACB,RAF1,SOCS5
31	14-3-3-mediated Signaling	13/127 (10%)	114/127 (90%)	6,97	0,10	BAX,CDKN1B,GRB2,GSK3B,JUN,KRAS,MAP2K1,MAP2K4,MAPK3,PDCD6IP,PIK3R1,RAF1,TNF
32	Telomerase Signaling	12/107 (11%)	95/107 (89%)	6,91	0,11	CDKN1A,E2F1,EGFR,ETS1,GRB2,HSP90B1,KRAS,MAP2K1,MAPK3,PIK3R1,PPP2R5C,RAF1
33	Ceramide Signaling	11/88 (13%)	77/88 (88%)	6,88	0,13	BCL2,JUN,KRAS,MAP2K1,MAP2K4,MAPK3,PIK3R1,PPP2R5C,RAF1,RELA,TNF
34	Endocannabinoid Developing Neuron Pathway	12/115 (10%)	103/115 (90%)	6,57	0,10	CCND1,CDKN1B,GSK3B,KRAS,MAP2K1,MAP2K4,MAPK3,MTOR,PIK3R1,PRKACB,RAF1,RPTOR
35	Insulin Receptor Signaling	13/140 (9%)	127/140 (91%)	6,47	0,09	EIF4E,GRB10,GRB2,GSK3B,KRAS,MAP2K1,MAPK3,MTOR,PIK3R1,PRKACB,PTEN,RAF1,RPTOR
36	Cholecystokinin/Gastrin-mediated Signaling	12/119 (10%)	107/119 (90%)	6,41	0,10	CCKBR,EGFR,GRB2,JUN,KRAS,MAP2K1,MAP2K4,MAPK3,PTGS2,RAF1,RHOT1,TNF
37	Role of Osteoblasts, Osteoclasts and Chondrocytes in Rheumatoid Arthritis	16/220 (7%)	204/220 (93%)	6,35	0,07	BCL2,BMPR2,DKK1,GSK3B,IFNG,IGF1,ITGA2,JUN,MAP2K4,MAPK3,MMP14,PIK3R1,RELA,SMO,TNF,WNT3A
38	FLT3 Signaling in Hematopoietic Progenitor Cells	10/80 (13%)	70/80 (88%)	6,30	0,13	EIF4E,FLT3,GRB2,KRAS,MAP2K1,MAPK3,MTOR,PIK3R1,RAF1,RPS6KA5
39	Role of MAPK Signaling in the Pathogenesis of Influenza	10/80 (13%)	70/80 (88%)	6,30	0,13	BAX,BCL2,IFNG,KRAS,MAP2K1,MAP2K4,MAPK3,PTGS2,RAF1,TNF
40	Natural Killer Cell Signaling	15/197 (8%)	182/197 (92%)	6,25	0,08	CFL2,FASLG,GRB2,HSPA14,HSPA1A/HSPA1B,IFNG,KRAS,MAP2K1,MAPK3,MICA,MTOR,PIK3R1,RAF1,RELA,WIPF1
41	Coronavirus Pathogenesis Pathway	13/150 (9%)	137/150 (91%)	6,12	0,09	BAX,BCL2,CCND1,E2F1,E2F3,EIF4E,FASLG,JUN,MAP2K4,MAPK3,PTGS2,RELA,TGFBR2
42	Regulation of Cellular Mechanics by Calpain Protease	9/65 (14%)	56/65 (86%)	6,11	0,14	CCND1,CCNE1,CDK6,CDKN1B,EGFR,GRB2,ITGA2,KRAS,MAPK3
43	BMP signaling pathway	10/85 (12%)	75/85 (88%)	6,05	0,12	BMPR2,GRB2,JUN,KRAS,MAP2K1,MAP2K4,MAPK3,PRKACB,RAF1,RELA
44	Myc Mediated Apoptosis Signaling	8/50 (16%)	42/50 (84%)	5,99	0,16	APAF1,BAX,BCL2,FAS,FASLG,MCL1,PRKACB,TNF
45	Regulation of IL-2 Expression in Activated and Anergic T Lymphocytes	10/89 (11%)	79/89 (89%)	5,87	0,11	GRB2,JUN,KRAS,MAP2K1,MAP2K4,MAPK3,RAF1,RELA,TGFBR2,TRA
46	Neuroinflammation Signaling Pathway	18/299 (6%)	281/299 (94%)	5,87	0,06	APP,BCL2,BDNF,BMPR2,FAS,FASLG,GSK3B,HMOX1,IFNG,IL6R,JUN,MAP2K4,MAPK3,PIK3R1,PTGS2,RELA,TGFBR2,TNF
47	B Cell Receptor Signaling	14/185 (8%)	171/185 (92%)	5,84	0,08	CFL2,ETS1,GRB2,GSK3B,JUN,KRAS,MAP2K1,MAP2K4,MAPK3,MTOR,PIK3R1,PTEN,RAF1,RELA
48	Human Embryonic Stem Cell Pluripotency	12/135 (9%)	123/135 (91%)	5,82	0,09	BDNF,BMPR2,FGF2,FGFR1,FGFR3,GSK3B,LEFTY1,LEFTY2,PIK3R1,SMO,TGFBR2,WNT3A
49	Rac Signaling	11/112 (10%)	101/112 (90%)	5,81	0,10	CD44,CFL2,ITGA2,JUN,KRAS,MAP2K1,MAP2K4,MAPK3,PIK3R1,RAF1,RELA
50	IL-17A Signaling in Gastric Cells	6/25 (24%)	19/25 (76%)	5,70	0,24	EGFR,JUN,MAP2K4,MAPK3,RELA,TNF
51	Melanocyte Development and Pigmentation Signaling	10/94 (11%)	84/94 (89%)	5,65	0,11	BCL2,GRB2,KITLG,KRAS,MAP2K1,MAPK3,PIK3R1,PRKACB,RAF1,RPS6KA5
52	Role of Macrophages, Fibroblasts and Endothelial Cells in Rheumatoid Arthritis	18/312 (6%)	294/312 (94%)	5,61	0,06	CCND1,DKK1,FGF2,GSK3B,IL6R,JUN,KRAS,MAP2K1,MAP2K4,MAPK3,NLK,PIK3R1,RAF1,RELA,SMO,TNF,VEGFA,WNT3A
53	TGF-β Signaling	10/96 (10%)	86/96 (90%)	5,56	0,10	BCL2,BMPR2,GRB2,JUN,KRAS,MAP2K1,MAP2K4,MAPK3,RAF1,TGFBR2
54	CNTF Signaling	8/57 (14%)	49/57 (86%)	5,54	0,14	GRB2,KRAS,MAP2K1,MAPK3,MTOR,PIK3R1,RAF1,RPS6KA5
55	PAK Signaling	10/97 (10%)	87/97 (90%)	5,52	0,10	CFL2,GRB2,ITGA2,KRAS,MAP2K1,MAP2K4,MAPK3,PIK3R1,RAF1,TNF
56	Corticotropin Releasing Hormone Signaling	12/145 (8%)	133/145 (92%)	5,49	0,08	BDNF,CACNA2D1,FASLG,JUN,MAP2K1,MAPK3,NPR3,PRKACB,PTGS2,RAF1,SMO,VEGFA
57	Agrin Interactions at Neuromuscular Junction	9/78 (12%)	69/78 (88%)	5,43	0,12	ACTA2,EGFR,ERBB4,ITGA2,JUN,KRAS,LAMC1,MAP2K4,MAPK3
58	Sirtuin Signaling Pathway	17/291 (6%)	274/291 (94%)	5,40	0,06	APP,ATG9A,BAX,E2F1,GSK3B,H3-3A/H3-3B,JUN,MAPK3,MTOR,PPIF,RELA,RPTOR,SMARCA5,SOD3,TNF,TOMM34,UCP2
59	T Cell Exhaustion Signaling Pathway	13/175 (7%)	162/175 (93%)	5,37	0,07	BMPR2,IFNG,IL6R,JUN,KRAS,MAP2K4,MAPK3,MTOR,PIK3R1,PPP2R5C,TGFBR2,TRA,VEGFA
60	IL-17 Signaling	9/80 (11%)	71/80 (89%)	5,34	0,11	GSK3B,JUN,KRAS,MAP2K1,MAP2K4,MAPK3,PIK3R1,PTGS2,RELA
61	Induction of Apoptosis by HIV1	8/61 (13%)	53/61 (87%)	5,31	0,13	APAF1,BAX,BCL2,FAS,FASLG,MAP2K4,RELA,TNF
62	Mouse Embryonic Stem Cell Pluripotency	10/103 (10%)	93/103 (90%)	5,28	0,10	BMPR2,GRB2,GSK3B,KRAS,MAP2K1,MAPK3,PIK3R1,RAF1,SMO,WNT3A
63	PEDF Signaling	9/82 (11%)	73/82 (89%)	5,25	0,11	BCL2,BDNF,FAS,FASLG,KRAS,MAPK3,PIK3R1,RAF1,RELA
64	PPAR Signaling	10/104 (10%)	94/104 (90%)	5,25	0,10	GRB2,HSP90B1,JUN,KRAS,MAP2K1,MAPK3,PTGS2,RAF1,RELA,TNF
65	T Cell Receptor Signaling	10/105 (10%)	95/105 (90%)	5,21	0,10	GRB2,JUN,KRAS,MAP2K1,MAP2K4,MAPK3,PIK3R1,RAF1,RELA,TRA
66	Integrin Signaling	14/213 (7%)	199/213 (93%)	5,12	0,07	ACTA2,GRB2,GSK3B,ITGA2,KRAS,MAP2K1,MAP2K4,MAPK3,PIK3R1,PTEN,RAF1,RHOT1,WIPF1,ZYX
67	Sertoli Cell-Sertoli Cell Junction Signaling	13/185 (7%)	172/185 (93%)	5,11	0,07	ACTA2,CLDN12,GSK3B,ITGA2,JUN,KRAS,MAP2K1,MAP2K4,MAPK3,PRKACB,PTEN,RAF1,TNF
68	GADD45 Signaling	5/19 (26%)	14/19 (74%)	5,05	0,26	CCND1,CCND2,CCND3,CCNE1,CDKN1A
69	4-1BB Signaling in T Lymphocytes	6/32 (19%)	26/32 (81%)	5,04	0,19	JUN,MAP2K1,MAP2K4,MAPK3,RELA,TNFSF9
70	Type I Diabetes Mellitus Signaling	10/111 (9%)	101/111 (91%)	4,99	0,09	APAF1,BCL2,FAS,FASLG,IFNG,MAP2K4,RELA,SOCS5,TNF,TRA
71	Th2 Pathway	11/136 (8%)	125/136 (92%)	4,98	0,08	BMPR2,GRB2,IFNG,JAG1,JUN,NOTCH2,NOTCH4,PIK3R1,SPI1,TGFBR2,TRA
72	Crosstalk between Dendritic Cells and Natural Killer Cells	9/89 (10%)	80/89 (90%)	4,96	0,10	ACTA2,CD69,CD83,FAS,FASLG,IFNG,MICA,RELA,TNF
73	HMGB1 Signaling	12/165 (7%)	153/165 (93%)	4,91	0,07	FASLG,IFNG,JUN,KRAS,MAP2K1,MAP2K4,MAPK3,PIK3R1,RELA,RHOT1,TNF,TNFSF9
74	NGF Signaling	10/114 (9%)	104/114 (91%)	4,89	0,09	BAX,GRB2,KRAS,MAP2K1,MAP2K4,MAPK3,PIK3R1,RAF1,RELA,RPS6KA5
75	GM-CSF Signaling	8/70 (11%)	62/70 (89%)	4,86	0,11	CCND1,ETS1,GRB2,KRAS,MAP2K1,MAPK3,PIK3R1,RAF1
76	UVC-Induced MAPK Signaling	7/51 (14%)	44/51 (86%)	4,85	0,14	EGFR,JUN,KRAS,MAP2K1,MAP2K4,MAPK3,RAF1
77	Germ Cell-Sertoli Cell Junction Signaling	12/171 (7%)	159/171 (93%)	4,75	0,07	ACTA2,CFL2,ITGA2,KRAS,MAP2K1,MAP2K4,MAPK3,PIK3R1,RHOT1,TGFBR2,TNF,ZYX
78	Th1 and Th2 Activation Pathway	12/171 (7%)	159/171 (93%)	4,75	0,07	BMPR2,GRB2,IFNG,IL6R,JAG1,JUN,NOTCH2,NOTCH4,PIK3R1,SPI1,TGFBR2,TRA
79	Cell Cycle Regulation by BTG Family Proteins	6/37 (16%)	31/37 (84%)	4,66	0,16	BTG2,CCND1,CCNE1,E2F1,E2F3,PPP2R5C
80	IL-15 Signaling	8/75 (11%)	67/75 (89%)	4,64	0,11	KRAS,MAP2K1,MAPK3,MTOR,PIK3R1,RAF1,RELA,RPTOR
81	Systemic Lupus Erythematosus In T Cell Signaling Pathway	17/334 (5%)	317/334 (95%)	4,62	0,05	CD44,DNMT1,ESR1,FAS,FASLG,GRB2,JUN,KRAS,MAP2K1,MAP2K4,MAPK3,MTOR,PIK3R1,PPP2R5C,RHOT1,RPTOR,TRA
82	Erythropoietin Signaling	8/76 (11%)	68/76 (89%)	4,60	0,11	GRB2,JUN,KRAS,MAP2K1,MAPK3,PIK3R1,RAF1,RELA
83	GDNF Family Ligand-Receptor Interactions	8/76 (11%)	68/76 (89%)	4,60	0,11	GRB2,JUN,KRAS,MAP2K1,MAP2K4,MAPK3,PIK3R1,RAF1
84	Docosahexaenoic Acid (DHA) Signaling	6/38 (16%)	32/38 (84%)	4,59	0,16	APAF1,APP,BAX,BCL2,GSK3B,PIK3R1
85	VEGF Signaling	9/99 (9%)	90/99 (91%)	4,58	0,09	ACTA2,BCL2,GRB2,KRAS,MAP2K1,MAPK3,PIK3R1,RAF1,VEGFA
86	Role of CHK Proteins in Cell Cycle Checkpoint Control	7/57 (12%)	50/57 (88%)	4,52	0,12	CDC25A,CDKN1A,CHEK1,E2F1,E2F3,PLK1,PPP2R5C
87	Signaling by Rho Family GTPases	14/244 (6%)	230/244 (94%)	4,46	0,06	ACTA2,ARHGEF3,CFL2,ITGA2,JUN,MAP2K1,MAP2K4,MAPK3,PIK3R1,RAF1,RELA,RHOT1,SEPTIN2,WIPF1
88	Reelin Signaling in Neurons	10/129 (8%)	119/129 (92%)	4,42	0,08	APP,ARHGEF3,GSK3B,ITGA2,MAP2K1,MAP2K4,MAPK3,MTOR,PAFAH1B2,PIK3R1
89	p70S6K Signaling	10/129 (8%)	119/129 (92%)	4,42	0,08	EGFR,F2,GRB2,KRAS,MAP2K1,MAPK3,MTOR,PIK3R1,PPP2R5C,RAF1
90	Prolactin Signaling	8/81 (10%)	73/81 (90%)	4,39	0,10	GRB2,JUN,KRAS,MAP2K1,MAPK3,PIK3R1,RAF1,SOCS5
91	Actin Cytoskeleton Signaling	13/218 (6%)	205/218 (94%)	4,35	0,06	ACTA2,CFL2,F2,FGF16,FGF2,FGF7,GRB2,ITGA2,KRAS,MAP2K1,MAPK3,PIK3R1,RAF1
92	LPS-stimulated MAPK Signaling	8/82 (10%)	74/82 (90%)	4,35	0,10	JUN,KRAS,MAP2K1,MAP2K4,MAPK3,PIK3R1,RAF1,RELA
93	IL-2 Signaling	7/61 (11%)	54/61 (89%)	4,33	0,12	GRB2,JUN,KRAS,MAP2K1,MAPK3,PIK3R1,RAF1
94	Role of IL-17F in Allergic Inflammatory Airway Diseases	6/42 (14%)	36/42 (86%)	4,33	0,14	IGF1,MAP2K1,MAPK3,RAF1,RELA,RPS6KA5
95	PPARα/RXRα Activation	12/190 (6%)	178/190 (94%)	4,30	0,06	BMPR2,GRB2,HSP90B1,JUN,KRAS,MAP2K1,MAP2K4,MAPK3,PRKACB,RAF1,RELA,TGFBR2
96	CDK5 Signaling	9/108 (8%)	99/108 (92%)	4,28	0,08	BDNF,ITGA2,KRAS,LAMC1,MAP2K1,MAPK3,PPP2R5C,PRKACB,RAF1
97	Xenobiotic Metabolism Signaling	15/287 (5%)	272/287 (95%)	4,27	0,05	ABCB1,GSTM4,HMOX1,HSP90B1,KRAS,MAP2K1,MAP2K4,MAPK3,NR1I2,PIK3R1,PPP2R5C,RAF1,RELA,SOD3,TNF
98	Oncostatin M Signaling	6/43 (14%)	37/43 (86%)	4,27	0,14	GRB2,KRAS,MAP2K1,MAPK3,RAF1,TIMP3
99	Thrombopoietin Signaling	7/63 (11%)	56/63 (89%)	4,24	0,11	GRB2,JUN,KRAS,MAP2K1,MAPK3,PIK3R1,RAF1
100	EIF2 Signaling	13/224 (6%)	211/224 (94%)	4,23	0,06	ACTA2,BCL2,CCND1,EIF4E,GRB2,GSK3B,IGF1R,KRAS,MAP2K1,MAPK3,PIK3R1,RAF1,VEGFA
101	PDGF Signaling	8/86 (9%)	78/86 (91%)	4,20	0,09	GRB2,JUN,KRAS,MAP2K1,MAP2K4,MAPK3,PIK3R1,RAF1
102	Role of PI3K/AKT Signaling in the Pathogenesis of Influenza	7/64 (11%)	57/64 (89%)	4,19	0,11	GSK3B,IFNG,KPNA3,MAP2K1,MAPK3,PIK3R1,RELA
103	IL-17A Signaling in Airway Cells	7/64 (11%)	57/64 (89%)	4,19	0,11	GSK3B,MAP2K1,MAP2K4,MAPK3,PIK3R1,PTEN,RELA
104	CD40 Signaling	7/65 (11%)	58/65 (89%)	4,15	0,11	JUN,MAP2K1,MAP2K4,MAPK3,PIK3R1,PTGS2,RELA
105	Tight Junction Signaling	11/168 (7%)	157/168 (93%)	4,13	0,07	ACTA2,CLDN12,JUN,NAPG,PPP2R5C,PRKACB,PTEN,RELA,TGFBR2,TNF,VTI1B
106	Mitotic Roles of Polo-Like Kinase	7/66 (11%)	59/66 (89%)	4,11	0,11	CDC25A,HSP90B1,KIF23,PLK1,PPP2R5C,PRC1,WEE1
107	ErbB4 Signaling	7/67 (10%)	60/67 (90%)	4,07	0,10	ERBB4,GRB2,KRAS,MAP2K1,MAPK3,PIK3R1,RAF1
108	Axonal Guidance Signaling	20/484 (4%)	464/484 (96%)	4,04	0,04	ADAM9,BDNF,CFL2,EIF4E,GRB2,GSK3B,IGF1,ITGA2,KRAS,MAP2K1,MAPK3,MMP14,PIK3R1,PRKACB,RAF1,RTN4,SMO,VEGFA,WIPF1,WNT3A
109	Death Receptor Signaling	8/91 (9%)	83/91 (91%)	4,03	0,09	ACTA2,APAF1,BCL2,FAS,FASLG,MAP2K4,RELA,TNF
110	GNRH Signaling	11/173 (6%)	162/173 (94%)	4,02	0,06	CACNA2D1,EGFR,GRB2,JUN,KRAS,MAP2K1,MAP2K4,MAPK3,PRKACB,RAF1,RELA
111	Growth Hormone Signaling	7/71 (10%)	64/71 (90%)	3,90	0,10	CSHL1,IGF1,IGF1R,MAPK3,PIK3R1,RPS6KA5,SOCS5
112	Synaptogenesis Signaling Pathway	15/312 (5%)	297/312 (95%)	3,87	0,05	BDNF,CACNA2D1,CADM1,GRB2,GRIA2,GSK3B,KRAS,MAPK3,MARCKS,MTOR,NAPG,PIK3R1,PRKACB,RAF1,VTI1B
113	Epithelial Adherens Junction Signaling	10/152 (7%)	142/152 (93%)	3,82	0,07	ACTA2,BMPR2,EGFR,FGFR1,KRAS,NOTCH2,NOTCH4,PTEN,TGFBR2,ZYX
114	UVA-Induced MAPK Signaling	8/98 (8%)	90/98 (92%)	3,80	0,08	EGFR,JUN,KRAS,MAP2K4,MAPK3,MTOR,PIK3R1,RPS6KA5
115	Hepatic Cholestasis	11/185 (6%)	174/185 (94%)	3,76	0,06	ABCB1,ESR1,FASLG,IFNG,JUN,MAP2K4,NR1I2,PRKACB,RELA,TNF,TNFSF9
116	Cytotoxic T Lymphocyte-mediated Apoptosis of Target Cells	5/34 (15%)	29/34 (85%)	3,75	0,15	APAF1,BCL2,FAS,FASLG,TRA
117	Regulation of eIF4 and p70S6K Signaling	10/157 (6%)	147/157 (94%)	3,71	0,06	EIF4E,GRB2,ITGA2,KRAS,MAP2K1,MAPK3,MTOR,PIK3R1,PPP2R5C,RAF1
118	Role of IL-17A in Arthritis	6/54 (11%)	48/54 (89%)	3,71	0,11	MAP2K1,MAP2K4,MAPK3,PIK3R1,PTGS2,RELA
119	Production of Nitric Oxide and Reactive Oxygen Species in Macrophages	11/188 (6%)	177/188 (94%)	3,70	0,06	IFNG,JUN,MAP2K1,MAP2K4,MAPK3,PIK3R1,PPP2R5C,RELA,RHOT1,SPI1,TNF
120	Antiproliferative Role of Somatostatin Receptor 2	7/77 (9%)	70/77 (91%)	3,68	0,09	CDKN1A,CDKN1B,KRAS,MAP2K1,MAPK3,NPR3,PIK3R1
121	Gα12/13 Signaling	9/130 (7%)	121/130 (93%)	3,66	0,07	F2,JUN,KRAS,MAP2K1,MAP2K4,MAPK3,PIK3R1,RAF1,RELA
122	Sumoylation Pathway	8/103 (8%)	95/103 (92%)	3,65	0,08	ARHGDIA,ETS1,FAS,FASLG,JUN,MAP2K4,MYB,RHOT1
123	IL-3 Signaling	7/79 (9%)	72/79 (91%)	3,61	0,09	GRB2,JUN,KRAS,MAP2K1,MAPK3,PIK3R1,RAF1
124	IL-12 Signaling and Production in Macrophages	9/132 (7%)	123/132 (93%)	3,61	0,07	IFNG,JUN,MAP2K1,MAP2K4,MAPK3,PIK3R1,RELA,SPI1,TNF
125	Tec Kinase Signaling	10/164 (6%)	154/164 (94%)	3,56	0,06	ACTA2,FAS,FASLG,ITGA2,MAP2K4,PIK3R1,RELA,RHOT1,TNF,TRA
126	Gap Junction Signaling	11/198 (6%)	187/198 (94%)	3,51	0,06	ACTA2,EGFR,GRB2,GRIA2,KRAS,MAP2K1,MAPK3,NPR3,PIK3R1,PRKACB,RAF1
127	PI3K Signaling in B Lymphocytes	9/138 (7%)	129/138 (93%)	3,47	0,07	ATF6,JUN,KRAS,MAP2K1,MAPK3,PIK3R1,PTEN,RAF1,RELA
128	VEGF Family Ligand-Receptor Interactions	7/84 (8%)	77/84 (92%)	3,45	0,08	GRB2,KRAS,MAP2K1,MAPK3,PIK3R1,RAF1,VEGFA
129	Xenobiotic Metabolism General Signaling Pathway	9/143 (6%)	134/143 (94%)	3,35	0,06	GSTM4,HMOX1,KRAS,MAP2K1,MAP2K4,MAPK3,NR1I2,PIK3R1,RAF1
130	RANK Signaling in Osteoclasts	7/88 (8%)	81/88 (92%)	3,32	0,08	JUN,MAP2K1,MAP2K4,MAPK3,PIK3R1,RAF1,RELA
131	MIF Regulation of Innate Immunity	5/42 (12%)	37/42 (88%)	3,31	0,12	JUN,MAP2K4,MAPK3,PTGS2,RELA
132	Tumoricidal Function of Hepatic Natural Killer Cells	4/24 (17%)	20/24 (83%)	3,31	0,17	APAF1,BAX,FAS,FASLG
133	mTOR Signaling	11/210 (5%)	199/210 (95%)	3,29	0,05	EIF4E,HMOX1,KRAS,MAPK3,MTOR,PIK3R1,PPP2R5C,RHOT1,RPS6KA5,RPTOR,VEGFA
134	Fc Epsilon RI Signaling	8/117 (7%)	109/117 (93%)	3,28	0,07	GRB2,KRAS,MAP2K1,MAP2K4,MAPK3,PIK3R1,RAF1,TNF
135	p38 MAPK Signaling	8/118 (7%)	110/118 (93%)	3,25	0,07	DUSP1,FAS,FASLG,H3-3A/H3-3B,MAP2K4,RPS6KA5,TGFBR2,TNF
136	Role of NFAT in Regulation of the Immune Response	10/181 (6%)	171/181 (94%)	3,22	0,06	GRB2,GSK3B,JUN,KRAS,MAP2K1,MAPK3,PIK3R1,RAF1,RELA,TRA
137	IL-15 Production	8/121 (7%)	113/121 (93%)	3,18	0,07	EGFR,ERBB4,FGFR1,FGFR3,IGF1R,MAP2K1,MAP2K4,RELA
138	Apelin Liver Signaling Pathway	4/26 (15%)	22/26 (85%)	3,17	0,15	FAS,GSK3B,MAP2K4,TNF
139	Nur77 Signaling in T Lymphocytes	6/68 (9%)	62/68 (91%)	3,16	0,09	APAF1,BCL2,FASLG,MAP2K1,MAPK3,TRA
140	Inhibition of ARE-Mediated mRNA Degradation Pathway	8/122 (7%)	114/122 (93%)	3,16	0,07	CNOT6,FASLG,MAPK3,PPP2R5C,PRKACB,TIA1,TNF,TNFSF9
141	Role of Pattern Recognition Receptors in Recognition of Bacteria and Viruses	9/154 (6%)	145/154 (94%)	3,12	0,06	FASLG,IFNG,MAP2K4,MAPK3,PIK3R1,RELA,RNASEL,TNF,TNFSF9
142	PKCθ Signaling in T Lymphocytes	9/155 (6%)	146/155 (94%)	3,10	0,06	CACNA2D1,GRB2,JUN,KRAS,MAP2K4,MAPK3,PIK3R1,RELA,TRA
143	Phospholipase C Signaling	12/257 (5%)	245/257 (95%)	3,09	0,05	ARHGEF3,GRB2,HMOX1,ITGA2,KRAS,MAP2K1,MAPK3,MARCKS,RAF1,RELA,RHOT1,TRA
144	Synaptic Long Term Depression	10/189 (5%)	179/189 (95%)	3,07	0,05	CACNA2D1,GRIA2,IGF1,IGF1R,KRAS,MAP2K1,MAPK3,NPR3,PPP2R5C,RAF1
145	Amyotrophic Lateral Sclerosis Signaling	7/97 (7%)	90/97 (93%)	3,07	0,07	APAF1,BAX,BCL2,GRIA2,IGF1,PIK3R1,VEGFA
146	Graft-versus-Host Disease Signaling	5/48 (10%)	43/48 (90%)	3,04	0,10	FAS,FASLG,IFNG,TNF,TRA
147	Clathrin-mediated Endocytosis Signaling	10/193 (5%)	183/193 (95%)	3,00	0,05	ACTA2,F2,FGF16,FGF2,FGF7,GRB2,HGS,IGF1,PIK3R1,VEGFA
148	RAR Activation	10/194 (5%)	184/194 (95%)	2,99	0,05	DUSP1,GTF2H1,JUN,MAP2K1,MAP2K4,PIK3R1,PRKACB,PTEN,RELA,VEGFA
149	Semaphorin Neuronal Repulsive Signaling Pathway	8/130 (6%)	122/130 (94%)	2,98	0,06	CD44,CFL2,GSK3B,ITGA2,MAP2K1,MAP2K4,PIK3R1,PRKACB
150	TNFR1 Signaling	5/50 (10%)	45/50 (90%)	2,96	0,10	APAF1,JUN,MAP2K4,RELA,TNF
151	TNFR2 Signaling	4/30 (13%)	26/30 (87%)	2,92	0,13	JUN,MAP2K4,RELA,TNF
152	CD27 Signaling in Lymphocytes	5/53 (9%)	48/53 (91%)	2,84	0,09	APAF1,JUN,MAP2K1,MAP2K4,RELA
153	Protein Kinase A Signaling	15/399 (4%)	384/399 (96%)	2,76	0,04	CDC14B,CDC25A,DUSP1,GSK3B,H3-3A/H3-3B,MAP2K1,MAPK3,PHKB,PRKACB,PTEN,PTGS2,RAF1,RELA,SMO,TGFBR2
154	Unfolded protein response	5/56 (9%)	51/56 (91%)	2,74	0,09	ATF6,BCL2,HSP90B1,HSPA14,HSPA1A/HSPA1B
155	NF-κB Activation by Viruses	6/82 (7%)	76/82 (93%)	2,73	0,07	ITGA2,KRAS,MAPK3,PIK3R1,RAF1,RELA
156	Type II Diabetes Mellitus Signaling	8/142 (6%)	134/142 (94%)	2,73	0,06	CACNA2D1,MAP2K4,MAPK3,MTOR,PIK3R1,RELA,SOCS5,TNF
157	IL-17A Signaling in Fibroblasts	4/35 (11%)	31/35 (89%)	2,67	0,11	GSK3B,JUN,MAPK3,RELA
158	Ephrin Receptor Signaling	9/180 (5%)	171/180 (95%)	2,65	0,05	CFL2,GRB2,ITGA2,KRAS,MAP2K1,MAPK3,RAF1,VEGFA,WIPF1
159	Interferon Signaling	4/36 (11%)	32/36 (89%)	2,63	0,11	BAX,BCL2,IFNG,RELA
160	Relaxin Signaling	8/150 (5%)	142/150 (95%)	2,58	0,05	JUN,MAP2K1,MAPK3,NPR3,PIK3R1,PRKACB,RELA,VEGFA
161	FAT10 Signaling Pathway	3/18 (17%)	15/18 (83%)	2,58	0,17	IFNG,SQSTM1,TNF
162	Airway Pathology in Chronic Obstructive Pulmonary Disease	7/118 (6%)	111/118 (94%)	2,58	0,06	FASLG,FGF16,FGF2,FGF7,IFNG,TNF,TNFSF9
163	Sperm Motility	10/223 (4%)	213/223 (96%)	2,54	0,04	EGFR,ERBB4,FGFR1,FGFR3,IGF1R,MAP2K1,MAP2K4,PRKACB,SLC12A2,SLC16A10
164	CD28 Signaling in T Helper Cells	7/120 (6%)	113/120 (94%)	2,54	0,06	GRB2,JUN,MAP2K1,MAP2K4,PIK3R1,RELA,TRA
165	Altered T Cell and B Cell Signaling in Rheumatoid Arthritis	6/90 (7%)	84/90 (93%)	2,53	0,07	FAS,FASLG,IFNG,RELA,TNF,TRA
166	Th1 Pathway	7/121 (6%)	114/121 (94%)	2,52	0,06	GRB2,IFNG,IL6R,NOTCH2,NOTCH4,PIK3R1,TRA
167	Xenobiotic Metabolism CAR Signaling Pathway	9/189 (5%)	180/189 (95%)	2,51	0,05	ABCB1,EGFR,GSTM4,HSP90B1,MAP2K1,MAP2K4,MAPK3,PPP2R5C,SOD3
168	Th17 Activation Pathway	6/91 (7%)	85/91 (93%)	2,50	0,07	HSP90B1,IFNG,IL6R,MTOR,RELA,TRA
169	G Beta Gamma Signaling	7/122 (6%)	115/122 (94%)	2,50	0,06	CACNA2D1,EGFR,GRB2,KRAS,MAPK3,PRKACB,RAF1
170	Antiproliferative Role of TOB in T Cell Signaling	4/39 (10%)	35/39 (90%)	2,50	0,10	CCNE1,CDKN1B,TGFBR2,TRA
171	PXR/RXR Activation	5/65 (8%)	60/65 (92%)	2,45	0,08	ABCB1,NR1I2,PRKACB,RELA,TNF
172	Gαq Signaling	8/158 (5%)	150/158 (95%)	2,45	0,05	GSK3B,HMOX1,MAP2K1,MAPK3,PIK3R1,RAF1,RELA,RHOT1
173	CCR5 Signaling in Macrophages	6/94 (6%)	88/94 (94%)	2,44	0,06	CACNA2D1,FAS,FASLG,JUN,MAP2K4,TRA
174	Pyridoxal 5'-phosphate Salvage Pathway	5/66 (8%)	61/66 (92%)	2,42	0,08	CDK6,MAP2K1,MAP2K4,MAPK3,PLK1
175	G-Protein Coupled Receptor Signaling	11/272 (4%)	261/272 (96%)	2,40	0,04	DUSP1,GRB2,KRAS,LAMTOR3,MAP2K1,MAPK3,NPR3,PIK3R1,PRKACB,RAF1,RELA
176	Leukocyte Extravasation Signaling	9/197 (5%)	188/197 (95%)	2,39	0,05	ACTA2,CD44,CLDN12,ITGA2,MAP2K4,MMP14,PIK3R1,TIMP3,WIPF1
177	α-Adrenergic Signaling	6/96 (6%)	90/96 (94%)	2,39	0,06	KRAS,MAP2K1,MAPK3,PHKB,PRKACB,RAF1
178	ATM Signaling	6/97 (6%)	91/97 (94%)	2,37	0,06	CDC25A,CDKN1A,CHEK1,JUN,MAP2K4,PPP2R5C
179	Salvage Pathways of Pyrimidine Ribonucleotides	6/98 (6%)	92/98 (94%)	2,35	0,06	AICDA,CDK6,MAP2K1,MAP2K4,MAPK3,PLK1
180	IL-10 Signaling	5/69 (7%)	64/69 (93%)	2,34	0,07	HMOX1,JUN,MAP2K4,RELA,TNF
181	Apelin Pancreas Signaling Pathway	4/44 (9%)	40/44 (91%)	2,31	0,09	MAP2K4,PIK3R1,PRKACB,RELA
182	CXCR4 Signaling	8/167 (5%)	159/167 (95%)	2,30	0,05	JUN,KRAS,MAP2K1,MAP2K4,MAPK3,PIK3R1,RAF1,RHOT1
183	Cdc42 Signaling	8/167 (5%)	159/167 (95%)	2,30	0,05	CFL2,GSK3B,ITGA2,JUN,MAP2K4,RAF1,TRA,WIPF1
184	Neuropathic Pain Signaling In Dorsal Horn Neurons	6/101 (6%)	95/101 (94%)	2,28	0,06	BDNF,GRIA2,KCNN4,MAPK3,PIK3R1,PRKACB
185	Adipogenesis pathway	7/134 (5%)	127/134 (95%)	2,27	0,05	BMPR2,FGF2,FGFR1,FGFR3,GTF2H1,SMO,TNF
186	iNOS Signaling	4/45 (9%)	41/45 (91%)	2,27	0,09	HMGA1,IFNG,JUN,RELA

187	SAPK/JNK Signaling	6/102 (6%)	96/102 (94%)	2,26	0,06	GRB2,JUN,KRAS,MAP2K4,PIK3R1,TRA
188	Melatonin Signaling	5/72 (7%)	67/72 (93%)	2,26	0,07	MAP2K1,MAP2K4,MAPK3,PRKACB,RAF1
189	Actin Nucleation by ARP-WASP Complex	5/72 (7%)	67/72 (93%)	2,26	0,07	GRB2,ITGA2,KRAS,RHOT1,WIPF1
190	CREB Signaling in Neurons	9/207 (4%)	198/207 (96%)	2,25	0,04	CACNA2D1,GRB2,GRIA2,KRAS,MAP2K1,MAPK3,PIK3R1,PRKACB,RAF1
191	Androgen Signaling	7/136 (5%)	129/136 (95%)	2,24	0,05	CACNA2D1,CCND1,GTF2H1,JUN,MAPK3,PRKACB,RELA
192	T Helper Cell Differentiation	5/73 (7%)	68/73 (93%)	2,23	0,07	IFNG,IL6R,TGFBR2,TNF,TRA
193	Opioid Signaling Pathway	10/247 (4%)	237/247 (96%)	2,23	0,04	CACNA2D1,CDKN1B,GSK3B,KRAS,MAP2K1,MAP2K4,MAPK3,PRKACB,RAF1,RPS6KA5
194	Role of JAK1, JAK2 and TYK2 in Interferon Signaling	3/24 (13%)	21/24 (88%)	2,22	0,13	IFNG,RAF1,RELA
195	Leptin Signaling in Obesity	5/74 (7%)	69/74 (93%)	2,21	0,07	GRB2,MAP2K1,MAPK3,PIK3R1,PRKACB
196	TREM1 Signaling	5/75 (7%)	70/75 (93%)	2,19	0,07	CD83,GRB2,MAPK3,RELA,TNF
197	FcγRIIB Signaling in B Lymphocytes	5/75 (7%)	70/75 (93%)	2,19	0,07	CACNA2D1,GRB2,KRAS,MAP2K4,PIK3R1
198	Role of JAK family kinases in IL-6-type Cytokine Signaling	3/25 (12%)	22/25 (88%)	2,17	0,12	IL6R,MAP2K4,MAPK3
199	Paxillin Signaling	6/108 (6%)	102/108 (94%)	2,14	0,06	ACTA2,GRB2,ITGA2,KRAS,MAP2K4,PIK3R1
200	Cell Cycle: G2/M DNA Damage Checkpoint Regulation	4/49 (8%)	45/49 (92%)	2,14	0,08	CDKN1A,CHEK1,PLK1,WEE1
201	VDR/RXR Activation	5/78 (6%)	73/78 (94%)	2,11	0,06	CDKN1A,CDKN1B,IFNG,KLF4,WT1
202	RhoGDI Signaling	8/180 (4%)	172/180 (96%)	2,11	0,04	ACTA2,ARHGDIA,ARHGEF3,CD44,CFL2,ESR1,ITGA2,RHOT1
203	Amyloid Processing	4/50 (8%)	46/50 (92%)	2,11	0,08	APP,GSK3B,MAPK3,PRKACB
204	Chemokine Signaling	5/80 (6%)	75/80 (94%)	2,07	0,06	JUN,KRAS,MAP2K1,MAPK3,RAF1
205	Lymphotoxin β Receptor Signaling	4/53 (8%)	49/53 (92%)	2,02	0,08	APAF1,MAPK3,PIK3R1,RELA
206	fMLP Signaling in Neutrophils	6/116 (5%)	110/116 (95%)	2,00	0,05	KRAS,MAP2K1,MAPK3,PIK3R1,RAF1,RELA
207	Regulation Of The Epithelial Mesenchymal Transition In Development Pathway	5/84 (6%)	79/84 (94%)	1,98	0,06	GSK3B,JAG1,RELA,SMO,WNT3A
208	Systemic Lupus Erythematosus Signaling	9/229 (4%)	220/229 (96%)	1,98	0,04	GRB2,IL6R,JUN,KRAS,MAPK3,MTOR,PIK3R1,TNF,TRA
209	HIPPO signaling	5/85 (6%)	80/85 (94%)	1,96	0,06	CD44,LATS2,PPP2R5C,SCRIB,STK4
210	IL-4 Signaling	5/85 (6%)	80/85 (94%)	1,96	0,06	GRB2,HMGA1,KRAS,MTOR,PIK3R1
211	Allograft Rejection Signaling	5/86 (6%)	81/86 (94%)	1,94	0,06	FAS,FASLG,IFNG,TNF,TRA
212	Cell Cycle Control of Chromosomal Replication	4/56 (7%)	52/56 (93%)	1,94	0,07	CDK11B,CDK19,CDK6,PRIM1
213	MSP-RON Signaling Pathway	4/58 (7%)	54/58 (93%)	1,89	0,07	ACTA2,IFNG,PIK3R1,TNF
214	RhoA Signaling	6/123 (5%)	117/123 (95%)	1,88	0,05	ACTA2,ANLN,CFL2,IGF1,IGF1R,SEPTIN2
215	CCR3 Signaling in Eosinophils	6/124 (5%)	118/124 (95%)	1,86	0,05	CFL2,KRAS,MAP2K1,MAPK3,PIK3R1,RAF1
216	OX40 Signaling Pathway	5/90 (6%)	85/90 (94%)	1,86	0,06	BCL2,JUN,MAP2K4,RELA,TRA
217	Gαi Signaling	6/125 (5%)	119/125 (95%)	1,85	0,05	GRB2,KRAS,MAPK3,NPR3,PRKACB,RAF1
218	IL-9 Signaling	3/33 (9%)	30/33 (91%)	1,83	0,09	PIK3R1,RELA,TNF
219	Insulin Secretion Signaling Pathway	9/243 (4%)	234/243 (96%)	1,82	0,04	CSHL1,EIF4E,MAPK3,MTOR,NAPG,PIK3R1,PRKACB,RPS6KA5,VTI1B
220	Autophagy	4/61 (7%)	57/61 (93%)	1,81	0,07	ATG9A,BCL2,MTOR,SQSTM1
221	Xanthine and Xanthosine Salvage	1/1 (100%)	0/1 (0%)	1,80	1,00	PNP
222	MIF-mediated Glucocorticoid Regulation	3/34 (9%)	31/34 (91%)	1,80	0,09	MAPK3,PTGS2,RELA
223	Synaptic Long Term Potentiation	6/129 (5%)	123/129 (95%)	1,79	0,05	GRIA2,KRAS,MAP2K1,MAPK3,PRKACB,RAF1
224	Regulation of Actin-based Motility by Rho	5/94 (5%)	89/94 (95%)	1,79	0,05	ACTA2,ARHGDIA,ITGA2,RHOT1,WIPF1
225	Fcγ Receptor-mediated Phagocytosis in Macrophages and Monocytes	5/94 (5%)	89/94 (95%)	1,79	0,05	ACTA2,HMOX1,MAPK3,PIK3R1,PTEN
226	White Adipose Tissue Browning Pathway	6/129 (5%)	123/129 (95%)	1,79	0,05	BDNF,CACNA2D1,FGFR1,FGFR3,PRKACB,VEGFA
227	Calcium Signaling	8/206 (4%)	198/206 (96%)	1,78	0,04	ACTA2,CACNA2D1,GRIA2,MAPK3,PRKACB,TP63,TPM1,TPM3
228	Activation of IRF by Cytosolic Pattern Recognition Receptors	4/63 (6%)	59/63 (94%)	1,76	0,06	JUN,MAP2K4,RELA,TNF
229	DNA Methylation and Transcriptional Repression Signaling	3/35 (9%)	32/35 (91%)	1,76	0,09	DNMT1,DNMT3B,H3-3A/H3-3B
230	Colanic Acid Building Blocks Biosynthesis	2/14 (14%)	12/14 (86%)	1,71	0,14	UGDH,UGP2
231	AMPK Signaling	8/214 (4%)	206/214 (96%)	1,69	0,04	CCND1,CDKN1A,MTOR,PIK3R1,PPP2R5C,PRKACB,RAB9B,RPTOR
232	April Mediated Signaling	3/39 (8%)	36/39 (92%)	1,64	0,08	JUN,MAP2K4,RELA
233	Inhibition of Matrix Metalloproteases	3/39 (8%)	36/39 (92%)	1,64	0,08	MMP14,RECK,TIMP3
234	Role of JAK1 and JAK3 in γc Cytokine Signaling	4/69 (6%)	65/69 (94%)	1,63	0,06	GRB2,KRAS,MAPK3,PIK3R1
235	Granzyme B Signaling	2/16 (13%)	14/16 (88%)	1,59	0,13	APAF1,LMNB1
236	Dendritic Cell Maturation	7/183 (4%)	176/183 (96%)	1,59	0,04	CD83,MAP2K4,MAPK3,PIK3R1,RELA,TNF,TRA
237	B Cell Activating Factor Signaling	3/41 (7%)	38/41 (93%)	1,58	0,07	JUN,MAP2K4,RELA
238	Mechanisms of Viral Exit from Host Cells	3/41 (7%)	38/41 (93%)	1,58	0,07	ACTA2,LMNB1,PDCD6IP
239	RAN Signaling	2/17 (12%)	15/17 (88%)	1,54	0,12	KPNA2,KPNA3
240	Antioxidant Action of Vitamin C	5/109 (5%)	104/109 (95%)	1,54	0,05	HMOX1,MAP2K4,MAPK3,RELA,TNF
241	Protein Ubiquitination Pathway	9/273 (3%)	264/273 (97%)	1,53	0,03	DNAJB4,HSP90B1,HSPA14,HSPA1A/HSPA1B,IFNG,SMO,UBE2S,UBE4A,USP12
242	iCOS-iCOSL Signaling in T Helper Cells	5/111 (5%)	106/111 (95%)	1,51	0,05	GRB2,PIK3R1,PTEN,RELA,TRA
243	UDP-D-xylose and UDP-D-glucuronate Biosynthesis	1/2 (50%)	1/2 (50%)	1,51	0,50	UGDH
244	Guanine and Guanosine Salvage I	1/2 (50%)	1/2 (50%)	1,51	0,50	PNP
245	S-methyl-5'-thioadenosine Degradation II	1/2 (50%)	1/2 (50%)	1,51	0,50	MTAP
246	Adenine and Adenosine Salvage I	1/2 (50%)	1/2 (50%)	1,51	0,50	PNP
247	IL-23 Signaling Pathway	3/44 (7%)	41/44 (93%)	1,50	0,07	PIK3R1,RELA,TNF
248	Toll-like Receptor Signaling	4/76 (5%)	72/76 (95%)	1,50	0,05	JUN,MAP2K4,RELA,TNF
249	Role of Wnt/GSK-3β Signaling in the Pathogenesis of Influenza	4/78 (5%)	74/78 (95%)	1,46	0,05	GSK3B,IFNG,SMO,WNT3A
250	Role of Oct4 in Mammalian Embryonic Stem Cell Pluripotency	3/46 (7%)	43/46 (93%)	1,45	0,07	BMI1,CCNF,CDX2
251	Necroptosis Signaling Pathway	6/157 (4%)	151/157 (96%)	1,42	0,04	FAS,FASLG,PELI1,PPIF,TNF,TOMM34
252	Autoimmune Thyroid Disease Signaling	3/49 (6%)	46/49 (94%)	1,38	0,06	FAS,FASLG,TRA
253	Endoplasmic Reticulum Stress Pathway	2/21 (10%)	19/21 (90%)	1,37	0,10	ATF6,HSP90B1
254	TR/RXR Activation	4/84 (5%)	80/84 (95%)	1,36	0,05	MTOR,PIK3R1,SLC16A3,UCP2
255	Xenobiotic Metabolism AHR Signaling Pathway	4/85 (5%)	81/85 (95%)	1,35	0,05	GSTM4,HSP90B1,RELA,TNF
256	Differential Regulation of Cytokine Production in Intestinal Epithelial Cells by IL-17A and IL-17F	2/23 (9%)	21/23 (91%)	1,30	0,09	IFNG,TNF
257	Endocannabinoid Neuronal Synapse Pathway	5/128 (4%)	123/128 (96%)	1,29	0,04	CACNA2D1,GRIA2,MAPK3,PRKACB,PTGS2
258	CTLA4 Signaling in Cytotoxic T Lymphocytes	4/89 (4%)	85/89 (96%)	1,29	0,04	GRB2,PIK3R1,PPP2R5C,TRA
259	Transcriptional Regulatory Network in Embryonic Stem Cells	3/54 (6%)	51/54 (94%)	1,28	0,06	CDX2,GATA6,H3-3A/H3-3B
260	IL-22 Signaling	2/24 (8%)	22/24 (92%)	1,27	0,08	MAP2K4,MAPK3
261	IL-1 Signaling	4/91 (4%)	87/91 (96%)	1,26	0,04	JUN,MAP2K4,PRKACB,RELA
262	Airway Inflammation in Asthma	1/4 (25%)	3/4 (75%)	1,21	0,25	TNF
263	Arsenate Detoxification I (Glutaredoxin)	1/4 (25%)	3/4 (75%)	1,21	0,25	PNP
264	Heme Degradation	1/4 (25%)	3/4 (75%)	1,21	0,25	HMOX1
265	LPS/IL-1 Mediated Inhibition of RXR Function	7/224 (3%)	217/224 (97%)	1,19	0,03	ABCB1,GSTM4,JUN,MAP2K4,NR1I2,SOD3,TNF
266	Communication between Innate and Adaptive Immune Cells	4/96 (4%)	92/96 (96%)	1,19	0,04	CD83,IFNG,TNF,TRA
267	Iron homeostasis signaling pathway	5/137 (4%)	132/137 (96%)	1,19	0,04	BMPR2,EGFR,HMOX1,IL6R,MAPK3
268	Semaphorin Signaling in Neurons	3/60 (5%)	57/60 (95%)	1,17	0,05	CFL2,MAPK3,RHOT1
269	cAMP-mediated signaling	7/228 (3%)	221/228 (97%)	1,16	0,03	DUSP1,LAMTOR3,MAP2K1,MAPK3,NPR3,PRKACB,RAF1
270	Serine Biosynthesis	1/5 (20%)	4/5 (80%)	1,12	0,20	PSAT1
271	Virus Entry via Endocytic Pathways	4/107 (4%)	103/107 (96%)	1,05	0,04	ACTA2,ITGA2,KRAS,PIK3R1
272	Ceramide Biosynthesis	1/6 (17%)	5/6 (83%)	1,04	0,17	SPTLC1
273	Pentose Phosphate Pathway (Non-oxidative Branch)	1/6 (17%)	5/6 (83%)	1,04	0,17	RPIA
274	UDP-N-acetyl-D-glucosamine Biosynthesis II	1/6 (17%)	5/6 (83%)	1,04	0,17	GFPT1
275	Adenine and Adenosine Salvage III	1/6 (17%)	5/6 (83%)	1,04	0,17	PNP
276	Remodeling of Epithelial Adherens Junctions	3/68 (4%)	65/68 (96%)	1,04	0,04	ACTA2,HGS,ZYX
277	ERK5 Signaling	3/72 (4%)	69/72 (96%)	0,98	0,04	EGFR,KRAS,RPS6KA5
278	TWEAK Signaling	2/35 (6%)	33/35 (94%)	0,98	0,06	APAF1,RELA
279	Superpathway of Serine and Glycine Biosynthesis I	1/7 (14%)	6/7 (86%)	0,98	0,14	PSAT1
280	Purine Ribonucleosides Degradation to Ribose-1-phosphate	1/7 (14%)	6/7 (86%)	0,98	0,14	PNP
281	Glycogen Biosynthesis II (from UDP-D-Glucose)	1/7 (14%)	6/7 (86%)	0,98	0,14	UGP2
282	Caveolar-mediated Endocytosis Signaling	3/73 (4%)	70/73 (96%)	0,97	0,04	ACTA2,EGFR,ITGA2
283	Superoxide Radicals Degradation	1/8 (13%)	7/8 (88%)	0,92	0,13	SOD3
284	Salvage Pathways of Pyrimidine Deoxyribonucleotides	1/8 (13%)	7/8 (88%)	0,92	0,13	AICDA
285	tRNA Charging	2/39 (5%)	37/39 (95%)	0,90	0,05	HARS1,RARS1
286	Mitochondrial Dysfunction	5/171 (3%)	166/171 (97%)	0,88	0,03	APP,BCL2,MAP2K4,TXN2,UCP2
287	Sucrose Degradation V (Mammalian)	1/9 (11%)	8/9 (89%)	0,88	0,11	TPI1
288	Phagosome Formation	4/125 (3%)	121/125 (97%)	0,87	0,03	ITGA2,MARCKS,PIK3R1,RHOT1
289	Role of Hypercytokinemia/hyperchemokinemia in the Pathogenesis of Influenza	2/43 (5%)	41/43 (95%)	0,84	0,05	IFNG,TNF
290	Prostanoid Biosynthesis	1/10 (10%)	9/10 (90%)	0,84	0,10	PTGS2
291	Pentose Phosphate Pathway	1/10 (10%)	9/10 (90%)	0,84	0,10	RPIA
292	Purine Nucleotides De Novo Biosynthesis II	1/11 (9%)	10/11 (91%)	0,80	0,09	ADSS2
293	Ephrin A Signaling	2/47 (4%)	45/47 (96%)	0,78	0,04	CFL2,PIK3R1
294	Hematopoiesis from Multipotent Stem Cells	1/12 (8%)	11/12 (92%)	0,76	0,08	KITLG
295	Glycogen Degradation II	1/12 (8%)	11/12 (92%)	0,76	0,08	MTAP
296	Guanosine Nucleotides Degradation III	1/12 (8%)	11/12 (92%)	0,76	0,08	PNP
297	Hematopoiesis from Pluripotent Stem Cells	2/49 (4%)	47/49 (96%)	0,75	0,04	KITLG,TRA
298	Xenobiotic Metabolism PXR Signaling Pathway	5/192 (3%)	187/192 (97%)	0,73	0,03	ABCB1,GSTM4,HSP90B1,NR1I2,PRKACB
299	Urate Biosynthesis/Inosine 5'-phosphate Degradation	1/13 (8%)	12/13 (92%)	0,73	0,08	PNP
300	NAD Phosphorylation and Dephosphorylation	1/13 (8%)	12/13 (92%)	0,73	0,08	ACP2
301	Agranulocyte Adhesion and Diapedesis	5/193 (3%)	188/193 (97%)	0,73	0,03	ACTA2,CLDN12,ITGA2,MMP14,TNF
302	Leukotriene Biosynthesis	1/14 (7%)	13/14 (93%)	0,70	0,07	GSTM4
303	Glycogen Degradation III	1/14 (7%)	13/14 (93%)	0,70	0,07	MTAP
304	Role of Cytokines in Mediating Communication between Immune Cells	2/54 (4%)	52/54 (96%)	0,68	0,04	IFNG,TNF
305	Choline Biosynthesis III	1/15 (7%)	14/15 (93%)	0,68	0,07	HMOX1
306	Adenosine Nucleotides Degradation II	1/15 (7%)	14/15 (93%)	0,68	0,07	PNP
307	NER Pathway	3/103 (3%)	100/103 (97%)	0,66	0,03	GTF2H1,H3-3A/H3-3B,PRIM1
308	Mismatch Repair in Eukaryotes	1/16 (6%)	15/16 (94%)	0,65	0,06	MSH2
309	Chondroitin Sulfate Degradation (Metazoa)	1/16 (6%)	15/16 (94%)	0,65	0,06	HYAL3
310	Glutamate Receptor Signaling	2/57 (4%)	55/57 (96%)	0,65	0,04	GRIA2,SLC38A1
311	Ubiquinol-10 Biosynthesis (Eukaryotic)	1/17 (6%)	16/17 (94%)	0,63	0,06	ECHDC1
312	Dermatan Sulfate Degradation (Metazoa)	1/17 (6%)	16/17 (94%)	0,63	0,06	HYAL3
313	Gαs Signaling	3/107 (3%)	104/107 (97%)	0,63	0,03	MAP2K1,MAPK3,PRKACB
314	Retinoic acid Mediated Apoptosis Signaling	2/60 (3%)	58/60 (97%)	0,61	0,03	APAF1,IFNG
315	Differential Regulation of Cytokine Production in Macrophages and T Helper Cells by IL-17A and I	1/18 (6%)	17/18 (94%)	0,61	0,06	TNF
316	Purine Nucleotides Degradation II (Aerobic)	1/18 (6%)	17/18 (94%)	0,61	0,06	PNP
317	D-myo-inositol (1,3,4)-trisphosphate Biosynthesis	1/18 (6%)	17/18 (94%)	0,61	0,06	PTEN
318	DNA damage-induced 14-3-3σ Signaling	1/19 (5%)	18/19 (95%)	0,59	0,05	CCNE1
319	3-phosphoinositide Biosynthesis	4/166 (2%)	162/166 (98%)	0,58	0,02	CDC25A,DUSP1,PIK3R1,PTEN
320	CDP-diacylglycerol Biosynthesis I	1/20 (5%)	19/20 (95%)	0,57	0,05	GPAM
321	Fatty Acid α-oxidation	1/20 (5%)	19/20 (95%)	0,57	0,05	PTGS2
322	Inflammasome pathway	1/20 (5%)	19/20 (95%)	0,57	0,05	PANX1
323	Netrin Signaling	2/65 (3%)	63/65 (97%)	0,57	0,03	CACNA2D1,PRKACB
324	Neuroprotective Role of THOP1 in Alzheimer's Disease	3/116 (3%)	113/116 (97%)	0,56	0,03	APP,IFNG,PRKACB
325	Sphingosine-1-phosphate Signaling	3/117 (3%)	114/117 (97%)	0,55	0,03	MAPK3,PIK3R1,RHOT1
326	GP6 Signaling Pathway	3/119 (3%)	116/119 (97%)	0,54	0,03	GSK3B,LAMC1,PIK3R1
327	Phosphatidylglycerol Biosynthesis II (Non-plastidic)	1/22 (5%)	21/22 (95%)	0,53	0,05	GPAM
328	LXR/RXR Activation	3/121 (2%)	118/121 (98%)	0,53	0,02	PTGS2,RELA,TNF
329	Role of Lipids/Lipid Rafts in the Pathogenesis of Influenza	1/23 (4%)	22/23 (96%)	0,52	0,04	IFNG
330	Superpathway of D-myo-inositol (1,4,5)-trisphosphate Metabolism	1/23 (4%)	22/23 (96%)	0,52	0,04	PTEN
331	Ephrin B Signaling	2/72 (3%)	70/72 (97%)	0,50	0,03	CFL2,MAPK3
332	Granulocyte Adhesion and Diapedesis	4/180 (2%)	176/180 (98%)	0,50	0,02	CLDN12,ITGA2,MMP14,TNF
333	FXR/RXR Activation	3/126 (2%)	123/126 (98%)	0,50	0,02	MAP2K4,NR1I2,TNF
334	Macropinocytosis Signaling	2/76 (3%)	74/76 (97%)	0,47	0,03	KRAS,PIK3R1
335	Lipid Antigen Presentation by CD1	1/26 (4%)	25/26 (96%)	0,47	0,04	TRA
336	NAD Salvage Pathway II	1/26 (4%)	25/26 (96%)	0,47	0,04	ACP2
337	Glycolysis I	1/26 (4%)	25/26 (96%)	0,47	0,04	TPI1
338	Dopamine Receptor Signaling	2/77 (3%)	75/77 (97%)	0,47	0,03	PPP2R5C,PRKACB
339	Apelin Adipocyte Signaling Pathway	2/82 (2%)	80/82 (98%)	0,43	0,02	MAPK3,PRKACB
340	Superpathway of Inositol Phosphate Compounds	4/199 (2%)	195/199 (98%)	0,42	0,02	CDC25A,DUSP1,PIK3R1,PTEN
341	D-myo-inositol (1,4,5,6)-Tetrakisphosphate Biosynthesis	3/142 (2%)	139/142 (98%)	0,41	0,02	CDC25A,DUSP1,PTEN
342	D-myo-inositol (3,4,5,6)-tetrakisphosphate Biosynthesis	3/142 (2%)	139/142 (98%)	0,41	0,02	CDC25A,DUSP1,PTEN
343	Glutathione-mediated Detoxification	1/32 (3%)	31/32 (97%)	0,40	0,03	GSTM4
344	Role of JAK2 in Hormone-like Cytokine Signaling	1/34 (3%)	33/34 (97%)	0,38	0,03	SOCS5
345	Coagulation System	1/35 (3%)	34/35 (97%)	0,37	0,03	F2
346	Nucleotide Excision Repair Pathway	1/35 (3%)	34/35 (97%)	0,37	0,03	GTF2H1
347	Gustation Pathway	3/154 (2%)	151/154 (98%)	0,36	0,02	CACNA2D1,PANX1,PRKACB
348	GABA Receptor Signaling	2/95 (2%)	93/95 (98%)	0,36	0,02	CACNA2D1,KCNN4
349	Complement System	1/37 (3%)	36/37 (97%)	0,35	0,03	CD46
350	3-phosphoinositide Degradation	3/156 (2%)	153/156 (98%)	0,35	0,02	CDC25A,DUSP1,PTEN
351	D-myo-inositol-5-phosphate Metabolism	3/157 (2%)	154/157 (98%)	0,35	0,02	CDC25A,DUSP1,PTEN
352	Antigen Presentation Pathway	1/39 (3%)	38/39 (97%)	0,34	0,03	IFNG
353	Kinetochore Metaphase Signaling Pathway	2/101 (2%)	99/101 (98%)	0,33	0,02	PLK1,PPP2R5C
354	Triacylglycerol Biosynthesis	1/42 (2%)	41/42 (98%)	0,31	0,02	GPAM
355	Role of RIG1-like Receptors in Antiviral Innate Immunity	1/44 (2%)	43/44 (98%)	0,30	0,02	RELA
356	GPCR-Mediated Nutrient Sensing in Enteroendocrine Cells	2/112 (2%)	110/112 (98%)	0,28	0,02	CACNA2D1,PRKACB
357	Primary Immunodeficiency Signaling	1/50 (2%)	49/50 (98%)	0,26	0,02	AICDA
358	Assembly of RNA Polymerase II Complex	1/50 (2%)	49/50 (98%)	0,26	0,02	GTF2H1
359	Phototransduction Pathway	1/53 (2%)	52/53 (98%)	0,25	0,02	PRKACB
360	Maturity Onset Diabetes of Young (MODY) Signaling	1/59 (2%)	58/59 (98%)	0,22	0,02	UCP2
361	Calcium-induced T Lymphocyte Apoptosis	1/66 (2%)	65/66 (98%)	0,00	0,02	TRA
362	Dopamine-DARPP32 Feedback in cAMP Signaling	2/163 (1%)	161/163 (99%)	0,00	0,01	PPP2R5C,PRKACB
363	Phospholipases	1/64 (2%)	63/64 (98%)	0,00	0,02	HMOX1
364	GPCR-Mediated Integration of Enteroendocrine Signaling Exemplified by an L Cell	1/73 (1%)	72/73 (99%)	0,00	0,01	PRKACB
365	Phagosome Maturation	2/151 (1%)	149/151 (99%)	0,00	0,01	NAPG,VTI1B
366	Eicosanoid Signaling	1/66 (2%)	65/66 (98%)	0,00	0,02	PTGS2

Recent Type 1 diabetes Group

	Ingenuity Canonical Pathways	Overlaps with dataset	No overlap with dataset		Ratio	Molecules
**1**	STAT3 Pathway	13/135 (10%)	122/135 (90%)	7,43	0,10	BCL2,CDC25A,EGFR,FGF2,FGFR1,IGF1,IGF1R,IGF2R,MAP2K1,MAP2K4,MAPK3,RAF1,VEGFA
**2**	PTEN Signaling	11/126 (9%)	115/126 (91%)	6,04	0,09	BCL2,CCND1,EGFR,FGFR1,GRB2,IGF1R,IGF2R,ITGA2,MAP2K1,MAPK3,RAF1
**3**	Senescence Pathway	15/275 (5%)	260/275 (95%)	5,95	0,05	ASXL2,CCND1,CDC25A,CDK6,CHEK1,DMTF1,E2F3,EIF4E,JUN,MAP2K1,MAP2K4,MAPK3,PPP2R5C,RAF1,SQSTM1
**4**	IL-8 Signaling	13/200 (7%)	187/200 (94%)	5,95	0,07	BCL2,CCND1,CCND3,EGFR,HMOX1,JUN,MAP2K1,MAP2K4,MAPK3,PTGS2,RAF1,RHOT1,VEGFA
**5**	IL-7 Signaling Pathway	9/78 (12%)	69/78 (88%)	5,95	0,12	BCL2,CCND1,CCND3,CDC25A,GRB2,IFNG,JUN,MAPK3,MCL1
**6**	Cyclins and Cell Cycle Regulation	9/81 (11%)	72/81 (89%)	5,84	0,11	CCND1,CCND3,CCNE1,CDC25A,CDK6,E2F3,PPP2R5C,RAF1,WEE1

7	PI3K/AKT Signaling	12/175 (7%)	163/175 (93%)	5,79	0,07	BCL2,CCND1,EIF4E,GRB2,HSP90B1,ITGA2,MAP2K1,MAPK3,MCL1,PPP2R5C,PTGS2,RAF1
8	Estrogen Receptor Signaling	15/328 (5%)	313/328 (95%)	5,22	0,05	BCL2,CCND1,CFL2,EGFR,EIF4E,GRB2,HSP90B1,IGF1,IGF1R,IGF2R,JUN,MAP2K1,MAPK3,RAF1,VEGFA
9	UVB-Induced MAPK Signaling	7/52 (13%)	45/52 (87%)	5,03	0,14	EGFR,EIF4E,H3-3A/H3-3B,JUN,MAP2K1,MAP2K4,MAPK3
10	Aryl Hydrocarbon Receptor Signaling	10/143 (7%)	133/143 (93%)	4,95	0,07	CCND1,CCND3,CCNE1,CDK6,CHEK1,GSTM4,HSP90B1,JUN,MAPK3,NFIA
11	EGF Signaling	7/55 (13%)	48/55 (87%)	4,95	0,13	EGFR,GRB2,JUN,MAP2K1,MAP2K4,MAPK3,RAF1
12	HGF Signaling	9/111 (8%)	102/111 (92%)	4,94	0,08	CCND1,GRB2,ITGA2,JUN,MAP2K1,MAP2K4,MAPK3,PTGS2,RAF1
13	Regulation Of The Epithelial Mesenchymal Transition By Growth Factors Pathway	11/188 (6%)	177/188 (94%)	4,79	0,06	EGFR,FGF2,FGF7,FGFR1,GRB2,JUN,MAP2K1,MAP2K4,MAPK3,RAF1,TNFSF9
14	Cholecystokinin/Gastrin-mediated Signaling	9/119 (8%)	110/119 (92%)	4,74	0,08	EGFR,GRB2,JUN,MAP2K1,MAP2K4,MAPK3,PTGS2,RAF1,RHOT1
15	Regulation of the Epithelial-Mesenchymal Transition Pathway	11/192 (6%)	181/192 (94%)	4,74	0,06	EGFR,FGF2,FGF7,FGFR1,GRB2,MAP2K1,MAP2K4,MAPK3,NOTCH2,RAF1,WNT3A
16	Regulation of Cellular Mechanics by Calpain Protease	7/65 (11%)	58/65 (89%)	4,63	0,11	CCND1,CCNE1,CDK6,EGFR,GRB2,ITGA2,MAPK3
17	Cell Cycle: G1/S Checkpoint Regulation	7/67 (10%)	60/67 (90%)	4,57	0,10	BMI1,CCND1,CCND3,CCNE1,CDC25A,CDK6,E2F3
18	IGF-1 Signaling	8/104 (8%)	96/104 (92%)	4,34	0,08	GRB10,GRB2,IGF1,IGF1R,JUN,MAP2K1,MAPK3,RAF1
19	Neurotrophin/TRK Signaling	7/76 (9%)	69/76 (91%)	4,27	0,09	BDNF,GRB2,JUN,MAP2K1,MAP2K4,MAPK3,RAF1
20	Corticotropin Releasing Hormone Signaling	9/145 (6%)	136/145 (94%)	4,24	0,06	BDNF,CACNA2D1,JUN,MAP2K1,MAPK3,NPR3,PTGS2,RAF1,VEGFA
21	UVC-Induced MAPK Signaling	6/51 (12%)	45/51 (88%)	4,24	0,12	EGFR,JUN,MAP2K1,MAP2K4,MAPK3,RAF1
22	4-1BB Signaling in T Lymphocytes	5/32 (16%)	27/32 (84%)	4,08	0,16	JUN,MAP2K1,MAP2K4,MAPK3,TNFSF9
23	FGF Signaling	7/84 (8%)	77/84 (92%)	4,06	0,08	FGF2,FGF7,FGFR1,GRB2,MAP2K1,MAPK3,RAF1
24	Ceramide Signaling	7/88 (8%)	81/88 (92%)	3,94	0,08	BCL2,JUN,MAP2K1,MAP2K4,MAPK3,PPP2R5C,RAF1
25	IL-6 Signaling	8/125 (6%)	117/125 (94%)	3,88	0,06	GRB2,JUN,MAP2K1,MAP2K4,MAPK3,MCL1,RAF1,VEGFA
26	ErbB Signaling	7/94 (7%)	87/94 (93%)	3,77	0,07	EGFR,GRB2,JUN,MAP2K1,MAP2K4,MAPK3,RAF1
27	Neuregulin Signaling	7/96 (7%)	89/96 (93%)	3,73	0,07	EGFR,GRB2,HSP90B1,ITGA2,MAP2K1,MAPK3,RAF1
28	TGF-β Signaling	7/96 (7%)	89/96 (93%)	3,73	0,07	BCL2,GRB2,JUN,MAP2K1,MAP2K4,MAPK3,RAF1
29	ErbB2-ErbB3 Signaling	6/65 (9%)	59/65 (91%)	3,73	0,09	CCND1,GRB2,JUN,MAP2K1,MAPK3,RAF1
30	PAK Signaling	7/97 (7%)	90/97 (93%)	3,72	0,07	CFL2,GRB2,ITGA2,MAP2K1,MAP2K4,MAPK3,RAF1
31	Mitotic Roles of Polo-Like Kinase	6/66 (9%)	60/66 (91%)	3,71	0,09	CDC25A,HSP90B1,KIF23,PLK1,PPP2R5C,WEE1
32	PPAR Signaling	7/104 (7%)	97/104 (93%)	3,56	0,07	GRB2,HSP90B1,JUN,MAP2K1,MAPK3,PTGS2,RAF1
33	Glucocorticoid Receptor Signaling	12/336 (4%)	324/336 (96%)	3,51	0,04	BCL2,GRB2,GTF2H1,HSP90B1,HSPA1A/HSPA1B,IFNG,JUN,MAP2K1,MAP2K4,MAPK3,PTGS2,RAF1
34	NRF2-mediated Oxidative Stress Response	9/189 (5%)	180/189 (95%)	3,50	0,05	DNAJB4,GSTM4,HMOX1,JUN,MAP2K1,MAP2K4,MAPK3,RAF1,SQSTM1
35	Telomerase Signaling	7/107 (7%)	100/107 (93%)	3,50	0,07	EGFR,GRB2,HSP90B1,MAP2K1,MAPK3,PPP2R5C,RAF1
36	CDK5 Signaling	7/108 (6%)	101/108 (94%)	3,50	0,06	BDNF,ITGA2,LAMC1,MAP2K1,MAPK3,PPP2R5C,RAF1
37	ILK Signaling	9/190 (5%)	181/190 (95%)	3,50	0,05	CCND1,CFL2,JUN,MAP2K4,MAPK3,PPP2R5C,PTGS2,RHOT1,VEGFA
38	GDNF Family Ligand-Receptor Interactions	6/76 (8%)	70/76 (92%)	3,45	0,08	GRB2,JUN,MAP2K1,MAP2K4,MAPK3,RAF1
39	Rac Signaling	7/112 (6%)	105/112 (94%)	3,43	0,06	CFL2,ITGA2,JUN,MAP2K1,MAP2K4,MAPK3,RAF1
40	Agrin Interactions at Neuromuscular Junction	6/78 (8%)	72/78 (92%)	3,41	0,08	EGFR,ITGA2,JUN,LAMC1,MAP2K4,MAPK3
41	IL-17A Signaling in Gastric Cells	4/25 (16%)	21/25 (84%)	3,41	0,16	EGFR,JUN,MAP2K4,MAPK3
42	FLT3 Signaling in Hematopoietic Progenitor Cells	6/80 (8%)	74/80 (93%)	3,37	0,08	EIF4E,FLT3,GRB2,MAP2K1,MAPK3,RAF1
43	Estrogen-mediated S-phase Entry	4/26 (15%)	22/26 (85%)	3,37	0,15	CCND1,CCNE1,CDC25A,E2F3
44	BMP signaling pathway	6/85 (7%)	79/85 (93%)	3,24	0,07	GRB2,JUN,MAP2K1,MAP2K4,MAPK3,RAF1
45	Integrin Signaling	9/213 (4%)	204/213 (96%)	3,22	0,04	GRB2,ITGA2,MAP2K1,MAP2K4,MAPK3,RAF1,RHOT1,WIPF1,ZYX
46	PDGF Signaling	6/86 (7%)	80/86 (93%)	3,22	0,07	GRB2,JUN,MAP2K1,MAP2K4,MAPK3,RAF1
47	Actin Cytoskeleton Signaling	9/218 (4%)	209/218 (96%)	3,16	0,04	CFL2,F2,FGF2,FGF7,GRB2,ITGA2,MAP2K1,MAPK3,RAF1
48	14-3-3-mediated Signaling	7/127 (6%)	120/127 (94%)	3,16	0,06	GRB2,JUN,MAP2K1,MAP2K4,MAPK3,PDCD6IP,RAF1
49	Regulation of IL-2 Expression in Activated and Anergic T Lymphocytes	6/89 (7%)	83/89 (93%)	3,16	0,07	GRB2,JUN,MAP2K1,MAP2K4,MAPK3,RAF1
50	Role of CHK Proteins in Cell Cycle Checkpoint Control	5/57 (9%)	52/57 (91%)	3,14	0,09	CDC25A,CHEK1,E2F3,PLK1,PPP2R5C
51	p70S6K Signaling	7/129 (5%)	122/129 (95%)	3,14	0,05	EGFR,F2,GRB2,MAP2K1,MAPK3,PPP2R5C,RAF1
52	GNRH Signaling	8/173 (5%)	165/173 (95%)	3,14	0,05	CACNA2D1,EGFR,GRB2,JUN,MAP2K1,MAP2K4,MAPK3,RAF1
53	EIF2 Signaling	9/224 (4%)	215/224 (96%)	3,09	0,04	BCL2,CCND1,EIF4E,GRB2,IGF1R,MAP2K1,MAPK3,RAF1,VEGFA
54	Acute Phase Response Signaling	8/179 (4%)	171/179 (96%)	3,06	0,04	F2,GRB2,HMOX1,JUN,MAP2K1,MAP2K4,MAPK3,RAF1
55	Melanocyte Development and Pigmentation Signaling	6/94 (6%)	88/94 (94%)	3,06	0,06	BCL2,GRB2,KITLG,MAP2K1,MAPK3,RAF1
56	FAK Signaling	6/95 (6%)	89/95 (94%)	3,05	0,06	EGFR,GRB2,ITGA2,MAP2K1,MAPK3,RAF1
57	Ephrin Receptor Signaling	8/180 (4%)	172/180 (96%)	3,05	0,04	CFL2,GRB2,ITGA2,MAP2K1,MAPK3,RAF1,VEGFA,WIPF1
58	IL-2 Signaling	5/61 (8%)	56/61 (92%)	3,04	0,08	GRB2,JUN,MAP2K1,MAPK3,RAF1
59	Thrombopoietin Signaling	5/63 (8%)	58/63 (92%)	2,99	0,08	GRB2,JUN,MAP2K1,MAPK3,RAF1
60	Apoptosis Signaling	6/99 (6%)	93/99 (94%)	2,98	0,06	BCL2,MAP2K1,MAP2K4,MAPK3,MCL1,RAF1
61	VEGF Signaling	6/99 (6%)	93/99 (94%)	2,98	0,06	BCL2,GRB2,MAP2K1,MAPK3,RAF1,VEGFA
62	Production of Nitric Oxide and Reactive Oxygen Species in Macrophages	8/188 (4%)	180/188 (96%)	2,96	0,04	IFNG,JUN,MAP2K1,MAP2K4,MAPK3,PPP2R5C,RHOT1,SPI1
63	Synaptic Long Term Depression	8/189 (4%)	181/189 (96%)	2,95	0,04	CACNA2D1,IGF1,IGF1R,MAP2K1,MAPK3,NPR3,PPP2R5C,RAF1
64	CD40 Signaling	5/65 (8%)	60/65 (92%)	2,95	0,08	JUN,MAP2K1,MAP2K4,MAPK3,PTGS2
65	Pyridoxal 5'-phosphate Salvage Pathway	5/66 (8%)	61/66 (92%)	2,92	0,08	CDK6,MAP2K1,MAP2K4,MAPK3,PLK1
66	Cell Cycle Regulation by BTG Family Proteins	4/37 (11%)	33/37 (89%)	2,90	0,11	CCND1,CCNE1,E2F3,PPP2R5C
67	Signaling by Rho Family GTPases	9/244 (4%)	235/244 (96%)	2,90	0,04	CFL2,ITGA2,JUN,MAP2K1,MAP2K4,MAPK3,RAF1,RHOT1,WIPF1
68	T Cell Receptor Signaling	6/105 (6%)	99/105 (94%)	2,88	0,06	GRB2,JUN,MAP2K1,MAP2K4,MAPK3,RAF1
69	Natural Killer Cell Signaling	8/197 (4%)	189/197 (96%)	2,86	0,04	CFL2,GRB2,HSPA1A/HSPA1B,IFNG,MAP2K1,MAPK3,RAF1,WIPF1
70	Axonal Guidance Signaling	13/484 (3%)	471/484 (97%)	2,86	0,03	BDNF,CFL2,EIF4E,GRB2,IGF1,ITGA2,MAP2K1,MAPK3,RAF1,RTN4,VEGFA,WIPF1,WNT3A
71	Adrenomedullin signaling pathway	8/197 (4%)	189/197 (96%)	2,86	0,04	BCL2,GRB2,KCNN4,MAP2K1,MAP2K4,MAPK3,NPR3,RAF1
72	GM-CSF Signaling	5/70 (7%)	65/70 (93%)	2,84	0,07	CCND1,GRB2,MAP2K1,MAPK3,RAF1
73	Regulation of eIF4 and p70S6K Signaling	7/157 (4%)	150/157 (96%)	2,74	0,04	EIF4E,GRB2,ITGA2,MAP2K1,MAPK3,PPP2R5C,RAF1
74	MIF Regulation of Innate Immunity	4/42 (10%)	38/42 (90%)	2,74	0,10	JUN,MAP2K4,MAPK3,PTGS2
75	Erythropoietin Signaling	5/76 (7%)	71/76 (93%)	2,70	0,07	GRB2,JUN,MAP2K1,MAPK3,RAF1
76	Oncostatin M Signaling	4/43 (9%)	39/43 (91%)	2,70	0,09	GRB2,MAP2K1,MAPK3,RAF1
77	Role of PKR in Interferon Induction and Antiviral Response	6/117 (5%)	111/117 (95%)	2,68	0,05	HSP90B1,HSPA1A/HSPA1B,IFNG,JUN,MAP2K4,MAPK3
78	GADD45 Signaling	3/19 (16%)	16/19 (84%)	2,68	0,16	CCND1,CCND3,CCNE1
79	HMGB1 Signaling	7/165 (4%)	158/165 (96%)	2,66	0,04	IFNG,JUN,MAP2K1,MAP2K4,MAPK3,RHOT1,TNFSF9
80	IL-3 Signaling	5/79 (6%)	74/79 (94%)	2,65	0,06	GRB2,JUN,MAP2K1,MAPK3,RAF1
81	IL-17 Signaling	5/80 (6%)	75/80 (94%)	2,64	0,06	JUN,MAP2K1,MAP2K4,MAPK3,PTGS2
82	JAK/Stat Signaling	5/80 (6%)	75/80 (94%)	2,64	0,06	GRB2,JUN,MAP2K1,MAPK3,RAF1
83	PFKFB4 Signaling Pathway	4/46 (9%)	42/46 (91%)	2,63	0,09	FGF2,MAP2K1,MAP2K4,MAPK3
84	Prolactin Signaling	5/81 (6%)	76/81 (94%)	2,62	0,06	GRB2,JUN,MAP2K1,MAPK3,RAF1
85	LPS-stimulated MAPK Signaling	5/82 (6%)	77/82 (94%)	2,60	0,06	JUN,MAP2K1,MAP2K4,MAPK3,RAF1
86	Germ Cell-Sertoli Cell Junction Signaling	7/171 (4%)	164/171 (96%)	2,60	0,04	CFL2,ITGA2,MAP2K1,MAP2K4,MAPK3,RHOT1,ZYX
87	VEGF Family Ligand-Receptor Interactions	5/84 (6%)	79/84 (94%)	2,57	0,06	GRB2,MAP2K1,MAPK3,RAF1,VEGFA
88	Gα12/13 Signaling	6/130 (5%)	124/130 (95%)	2,51	0,05	F2,JUN,MAP2K1,MAP2K4,MAPK3,RAF1
89	IL-12 Signaling and Production in Macrophages	6/132 (5%)	126/132 (95%)	2,48	0,05	IFNG,JUN,MAP2K1,MAP2K4,MAPK3,SPI1
90	Sertoli Cell-Sertoli Cell Junction Signaling	7/185 (4%)	178/185 (96%)	2,43	0,04	CLDN12,ITGA2,JUN,MAP2K1,MAP2K4,MAPK3,RAF1
91	B Cell Receptor Signaling	7/185 (4%)	178/185 (96%)	2,43	0,04	CFL2,GRB2,JUN,MAP2K1,MAP2K4,MAPK3,RAF1
92	Xenobiotic Metabolism CAR Signaling Pathway	7/189 (4%)	182/189 (96%)	2,38	0,04	EGFR,GSTM4,HSP90B1,MAP2K1,MAP2K4,MAPK3,PPP2R5C
93	PPARα/RXRα Activation	7/190 (4%)	183/190 (96%)	2,37	0,04	GRB2,HSP90B1,JUN,MAP2K1,MAP2K4,MAPK3,RAF1
94	Unfolded protein response	4/56 (7%)	52/56 (93%)	2,37	0,07	ATF6,BCL2,HSP90B1,HSPA1A/HSPA1B
95	Insulin Receptor Signaling	6/140 (4%)	134/140 (96%)	2,37	0,04	EIF4E,GRB10,GRB2,MAP2K1,MAPK3,RAF1
96	CNTF Signaling	4/57 (7%)	53/57 (93%)	2,34	0,07	GRB2,MAP2K1,MAPK3,RAF1
97	ATM Signaling	5/97 (5%)	92/97 (95%)	2,33	0,05	CDC25A,CHEK1,JUN,MAP2K4,PPP2R5C
98	Xenobiotic Metabolism General Signaling Pathway	6/143 (4%)	137/143 (96%)	2,33	0,04	GSTM4,HMOX1,MAP2K1,MAP2K4,MAPK3,RAF1
99	Salvage Pathways of Pyrimidine Ribonucleotides	5/98 (5%)	93/98 (95%)	2,32	0,05	CDK6,MAP2K1,MAP2K4,MAPK3,PLK1
100	Mouse Embryonic Stem Cell Pluripotency	5/103 (5%)	98/103 (95%)	2,24	0,05	GRB2,MAP2K1,MAPK3,RAF1,WNT3A
101	Sumoylation Pathway	5/103 (5%)	98/103 (95%)	2,24	0,05	ARHGDIA,JUN,MAP2K4,MYB,RHOT1
102	ErbB4 Signaling	4/67 (6%)	63/67 (94%)	2,12	0,06	GRB2,MAP2K1,MAPK3,RAF1
103	NGF Signaling	5/114 (4%)	109/114 (96%)	2,06	0,04	GRB2,MAP2K1,MAP2K4,MAPK3,RAF1
104	Endocannabinoid Developing Neuron Pathway	5/115 (4%)	110/115 (96%)	2,05	0,04	CCND1,MAP2K1,MAP2K4,MAPK3,RAF1
105	CXCR4 Signaling	6/167 (4%)	161/167 (96%)	2,05	0,04	JUN,MAP2K1,MAP2K4,MAPK3,RAF1,RHOT1
106	Growth Hormone Signaling	4/71 (6%)	67/71 (94%)	2,05	0,06	CSHL1,IGF1,IGF1R,MAPK3
107	Cdc42 Signaling	6/167 (4%)	161/167 (96%)	2,05	0,04	CFL2,ITGA2,JUN,MAP2K4,RAF1,WIPF1
108	Fc Epsilon RI Signaling	5/117 (4%)	112/117 (96%)	2,04	0,04	GRB2,MAP2K1,MAP2K4,MAPK3,RAF1
109	Melatonin Signaling	4/72 (6%)	68/72 (94%)	2,04	0,06	MAP2K1,MAP2K4,MAPK3,RAF1
110	Actin Nucleation by ARP-WASP Complex	4/72 (6%)	68/72 (94%)	2,04	0,06	GRB2,ITGA2,RHOT1,WIPF1
111	Xenobiotic Metabolism Signaling	8/287 (3%)	279/287 (97%)	2,03	0,03	GSTM4,HMOX1,HSP90B1,MAP2K1,MAP2K4,MAPK3,PPP2R5C,RAF1
112	Role of NANOG in Mammalian Embryonic Stem Cell Pluripotency	5/119 (4%)	114/119 (96%)	2,01	0,04	GRB2,MAP2K1,MAPK3,RAF1,WNT3A
113	IL-15 Production	5/121 (4%)	116/121 (96%)	1,99	0,04	EGFR,FGFR1,IGF1R,MAP2K1,MAP2K4
114	G Beta Gamma Signaling	5/122 (4%)	117/122 (96%)	1,97	0,04	CACNA2D1,EGFR,GRB2,MAPK3,RAF1
115	T Cell Exhaustion Signaling Pathway	6/175 (3%)	169/175 (97%)	1,97	0,03	IFNG,JUN,MAP2K4,MAPK3,PPP2R5C,VEGFA
116	Chemokine Signaling	4/80 (5%)	76/80 (95%)	1,90	0,05	JUN,MAP2K1,MAPK3,RAF1
117	Reelin Signaling in Neurons	5/129 (4%)	124/129 (96%)	1,88	0,04	ITGA2,MAP2K1,MAP2K4,MAPK3,PAFAH1B2
118	PEDF Signaling	4/82 (5%)	78/82 (95%)	1,86	0,05	BCL2,BDNF,MAPK3,RAF1
119	Synaptogenesis Signaling Pathway	8/312 (3%)	304/312 (97%)	1,84	0,03	BDNF,CACNA2D1,CADM1,GRB2,MAPK3,NAPG,RAF1,VTI1B
120	Colanic Acid Building Blocks Biosynthesis	2/14 (14%)	12/14 (86%)	1,82	0,14	UGDH,UGP2
121	Androgen Signaling	5/136 (4%)	131/136 (96%)	1,80	0,04	CACNA2D1,CCND1,GTF2H1,JUN,MAPK3
122	Th2 Pathway	5/136 (4%)	131/136 (96%)	1,80	0,04	GRB2,IFNG,JUN,NOTCH2,SPI1
123	Clathrin-mediated Endocytosis Signaling	6/193 (3%)	187/193 (97%)	1,80	0,03	F2,FGF2,FGF7,GRB2,IGF1,VEGFA
124	iNOS Signaling	3/45 (7%)	42/45 (93%)	1,79	0,07	HMGA1,IFNG,JUN
125	PI3K Signaling in B Lymphocytes	5/138 (4%)	133/138 (96%)	1,79	0,04	ATF6,JUN,MAP2K1,MAPK3,RAF1
126	Phospholipase C Signaling	7/257 (3%)	250/257 (97%)	1,78	0,03	GRB2,HMOX1,ITGA2,MAP2K1,MAPK3,RAF1,RHOT1
127	Gap Junction Signaling	6/198 (3%)	192/198 (97%)	1,76	0,03	EGFR,GRB2,MAP2K1,MAPK3,NPR3,RAF1
128	Xanthine and Xanthosine Salvage	1/1 (100%)	0/1 (0%)	1,71	1,00	PNP
129	Cell Cycle: G2/M DNA Damage Checkpoint Regulation	3/49 (6%)	46/49 (94%)	1,70	0,06	CHEK1,PLK1,WEE1
130	Regulation of Actin-based Motility by Rho	4/94 (4%)	90/94 (96%)	1,69	0,04	ARHGDIA,ITGA2,RHOT1,WIPF1
131	α-Adrenergic Signaling	4/96 (4%)	92/96 (96%)	1,66	0,04	MAP2K1,MAPK3,PHKB,RAF1
132	Relaxin Signaling	5/150 (3%)	145/150 (97%)	1,66	0,03	JUN,MAP2K1,MAPK3,NPR3,VEGFA
133	mTOR Signaling	6/210 (3%)	204/210 (97%)	1,65	0,03	EIF4E,HMOX1,MAPK3,PPP2R5C,RHOT1,VEGFA
134	UVA-Induced MAPK Signaling	4/98 (4%)	94/98 (96%)	1,64	0,04	EGFR,JUN,MAP2K4,MAPK3
135	FAT10 Signaling Pathway	2/18 (11%)	16/18 (89%)	1,64	0,11	IFNG,SQSTM1
136	CD27 Signaling in Lymphocytes	3/53 (6%)	50/53 (94%)	1,63	0,06	JUN,MAP2K1,MAP2K4
137	Role of Pattern Recognition Receptors in Recognition of Bacteria and Viruses	5/154 (3%)	149/154 (97%)	1,62	0,03	IFNG,MAP2K4,MAPK3,RNASEL,TNFSF9
138	PKCθ Signaling in T Lymphocytes	5/155 (3%)	150/155 (97%)	1,61	0,03	CACNA2D1,GRB2,JUN,MAP2K4,MAPK3
139	Gαq Signaling	5/158 (3%)	153/158 (97%)	1,58	0,03	HMOX1,MAP2K1,MAPK3,RAF1,RHOT1
140	Sperm Motility	6/223 (3%)	217/223 (97%)	1,55	0,03	EGFR,FGFR1,IGF1R,MAP2K1,MAP2K4,SLC12A2
141	Sirtuin Signaling Pathway	7/291 (2%)	284/291 (98%)	1,54	0,02	ATG9A,H3-3A/H3-3B,JUN,MAPK3,PPIF,TOMM34,UCP2
142	Endoplasmic Reticulum Stress Pathway	2/21 (10%)	19/21 (90%)	1,53	0,10	ATF6,HSP90B1
143	Semaphorin Signaling in Neurons	3/60 (5%)	57/60 (95%)	1,50	0,05	CFL2,MAPK3,RHOT1
144	Tight Junction Signaling	5/168 (3%)	163/168 (97%)	1,49	0,03	CLDN12,JUN,NAPG,PPP2R5C,VTI1B
145	Autophagy	3/61 (5%)	58/61 (95%)	1,49	0,05	ATG9A,BCL2,SQSTM1
146	Th1 and Th2 Activation Pathway	5/171 (3%)	166/171 (97%)	1,47	0,03	GRB2,IFNG,JUN,NOTCH2,SPI1
147	UDP-D-xylose and UDP-D-glucuronate Biosynthesis	1/2 (50%)	1/2 (50%)	1,46	0,50	UGDH
148	Guanine and Guanosine Salvage I	1/2 (50%)	1/2 (50%)	1,46	0,50	PNP
149	Adenine and Adenosine Salvage I	1/2 (50%)	1/2 (50%)	1,46	0,50	PNP
150	IL-22 Signaling	2/24 (8%)	22/24 (92%)	1,44	0,08	MAP2K4,MAPK3
151	IL-17A Signaling in Airway Cells	3/64 (5%)	61/64 (95%)	1,44	0,05	MAP2K1,MAP2K4,MAPK3
152	Role of JAK1, JAK2 and TYK2 in Interferon Signaling	2/24 (8%)	22/24 (92%)	1,44	0,08	IFNG,RAF1
153	Role of JAK family kinases in IL-6-type Cytokine Signaling	2/25 (8%)	23/25 (92%)	1,41	0,08	MAP2K4,MAPK3
154	NF-κB Signaling	5/179 (3%)	174/179 (97%)	1,41	0,03	EGFR,FGFR1,IGF1R,IGF2R,RAF1
155	CD28 Signaling in T Helper Cells	4/120 (3%)	116/120 (97%)	1,40	0,03	GRB2,JUN,MAP2K1,MAP2K4
156	Role of NFAT in Regulation of the Immune Response	5/181 (3%)	176/181 (97%)	1,39	0,03	GRB2,JUN,MAP2K1,MAPK3,RAF1
157	Nur77 Signaling in T Lymphocytes	3/68 (4%)	65/68 (96%)	1,39	0,04	BCL2,MAP2K1,MAPK3
158	Inhibition of ARE-Mediated mRNA Degradation Pathway	4/122 (3%)	118/122 (97%)	1,38	0,03	MAPK3,PPP2R5C,TIA1,TNFSF9
159	IL-10 Signaling	3/69 (4%)	66/69 (96%)	1,38	0,04	HMOX1,JUN,MAP2K4
160	RhoA Signaling	4/123 (3%)	119/123 (97%)	1,37	0,03	ANLN,CFL2,IGF1,IGF1R
161	CCR3 Signaling in Eosinophils	4/124 (3%)	120/124 (97%)	1,36	0,03	CFL2,MAP2K1,MAPK3,RAF1
162	Protein Kinase A Signaling	8/399 (2%)	391/399 (98%)	1,36	0,02	CDC14B,CDC25A,H3-3A/H3-3B,MAP2K1,MAPK3,PHKB,PTGS2,RAF1
163	Gαi Signaling	4/125 (3%)	121/125 (97%)	1,36	0,03	GRB2,MAPK3,NPR3,RAF1
164	White Adipose Tissue Browning Pathway	4/129 (3%)	125/129 (97%)	1,32	0,03	BDNF,CACNA2D1,FGFR1,VEGFA
165	Semaphorin Neuronal Repulsive Signaling Pathway	4/130 (3%)	126/130 (97%)	1,31	0,03	CFL2,ITGA2,MAP2K1,MAP2K4
166	RAR Activation	5/194 (3%)	189/194 (97%)	1,31	0,03	GTF2H1,JUN,MAP2K1,MAP2K4,VEGFA
167	FcγRIIB Signaling in B Lymphocytes	3/75 (4%)	72/75 (96%)	1,31	0,04	CACNA2D1,GRB2,MAP2K4
168	IL-15 Signaling	3/75 (4%)	72/75 (96%)	1,31	0,04	MAP2K1,MAPK3,RAF1
169	TNFR2 Signaling	2/30 (7%)	28/30 (93%)	1,30	0,07	JUN,MAP2K4
170	Antiproliferative Role of Somatostatin Receptor 2	3/77 (4%)	74/77 (96%)	1,28	0,04	MAP2K1,MAPK3,NPR3
171	Human Embryonic Stem Cell Pluripotency	4/135 (3%)	131/135 (97%)	1,27	0,03	BDNF,FGF2,FGFR1,WNT3A
172	G-Protein Coupled Receptor Signaling	6/272 (2%)	266/272 (98%)	1,25	0,02	GRB2,LAMTOR3,MAP2K1,MAPK3,NPR3,RAF1
173	Protein Ubiquitination Pathway	6/273 (2%)	267/273 (98%)	1,25	0,02	DNAJB4,HSP90B1,HSPA1A/HSPA1B,IFNG,UBE2S,UBE4A
174	Arsenate Detoxification I (Glutaredoxin)	1/4 (25%)	3/4 (75%)	1,22	0,25	PNP
175	Heme Degradation	1/4 (25%)	3/4 (75%)	1,22	0,25	HMOX1
176	NF-κB Activation by Viruses	3/82 (4%)	79/82 (96%)	1,22	0,04	ITGA2,MAPK3,RAF1
177	CREB Signaling in Neurons	5/207 (2%)	202/207 (98%)	1,22	0,02	CACNA2D1,GRB2,MAP2K1,MAPK3,RAF1
178	MIF-mediated Glucocorticoid Regulation	2/34 (6%)	32/34 (94%)	1,22	0,06	MAPK3,PTGS2
179	IL-17A Signaling in Fibroblasts	2/35 (6%)	33/35 (94%)	1,20	0,06	JUN,MAPK3
180	Interferon Signaling	2/36 (6%)	34/36 (94%)	1,18	0,06	BCL2,IFNG
181	Serine Biosynthesis	1/5 (20%)	4/5 (80%)	1,14	0,20	PSAT1
182	OX40 Signaling Pathway	3/90 (3%)	87/90 (97%)	1,13	0,03	BCL2,JUN,MAP2K4
183	Epithelial Adherens Junction Signaling	4/152 (3%)	148/152 (97%)	1,13	0,03	EGFR,FGFR1,NOTCH2,ZYX
184	April Mediated Signaling	2/39 (5%)	37/39 (95%)	1,12	0,05	JUN,MAP2K4
185	tRNA Charging	2/39 (5%)	37/39 (95%)	1,12	0,05	HARS1,RARS1
186	CCR5 Signaling in Macrophages	3/94 (3%)	91/94 (97%)	1,09	0,03	CACNA2D1,JUN,MAP2K4
187	cAMP-mediated signaling	5/228 (2%)	223/228 (98%)	1,09	0,02	LAMTOR3,MAP2K1,MAPK3,NPR3,RAF1
188	B Cell Activating Factor Signaling	2/41 (5%)	39/41 (95%)	1,09	0,05	JUN,MAP2K4
189	Ceramide Biosynthesis	1/6 (17%)	5/6 (83%)	1,08	0,17	SPTLC1
190	UDP-N-acetyl-D-glucosamine Biosynthesis II	1/6 (17%)	5/6 (83%)	1,08	0,17	GFPT1
191	Adenine and Adenosine Salvage III	1/6 (17%)	5/6 (83%)	1,08	0,17	PNP
192	HOTAIR Regulatory Pathway	4/160 (3%)	156/160 (98%)	1,08	0,03	H3-3A/H3-3B,LAMTOR5,mir-9,WNT3A
193	Superpathway of Serine and Glycine Biosynthesis I	1/7 (14%)	6/7 (86%)	1,03	0,14	PSAT1
194	Purine Ribonucleosides Degradation to Ribose-1-phosphate	1/7 (14%)	6/7 (86%)	1,03	0,14	PNP

195	Glycogen Biosynthesis II (from UDP-D-Glucose)	1/7 (14%)	6/7 (86%)	1,03	0,14	UGP2
196	SAPK/JNK Signaling	3/102 (3%)	99/102 (97%)	1,03	0,03	GRB2,JUN,MAP2K4
197	Role of Oct4 in Mammalian Embryonic Stem Cell Pluripotency	2/46 (4%)	44/46 (96%)	1,02	0,04	BMI1,CCNF
198	NER Pathway	3/103 (3%)	100/103 (97%)	1,02	0,03	GTF2H1,H3-3A/H3-3B,PRIM1
199	Insulin Secretion Signaling Pathway	5/243 (2%)	238/243 (98%)	1,02	0,02	CSHL1,EIF4E,MAPK3,NAPG,VTI1B
200	Opioid Signaling Pathway	5/247 (2%)	242/247 (98%)	1,00	0,02	CACNA2D1,MAP2K1,MAP2K4,MAPK3,RAF1
201	Paxillin Signaling	3/108 (3%)	105/108 (97%)	0,98	0,03	GRB2,ITGA2,MAP2K4
202	Myc Mediated Apoptosis Signaling	2/50 (4%)	48/50 (96%)	0,98	0,04	BCL2,MCL1
203	TNFR1 Signaling	2/50 (4%)	48/50 (96%)	0,98	0,04	JUN,MAP2K4
204	Antioxidant Action of Vitamin C	3/109 (3%)	106/109 (97%)	0,98	0,03	HMOX1,MAP2K4,MAPK3
205	RhoGDI Signaling	4/180 (2%)	176/180 (98%)	0,96	0,02	ARHGDIA,CFL2,ITGA2,RHOT1
206	Sucrose Degradation V (Mammalian)	1/9 (11%)	8/9 (89%)	0,95	0,11	TPI1
207	fMLP Signaling in Neutrophils	3/116 (3%)	113/116 (97%)	0,92	0,03	MAP2K1,MAPK3,RAF1
208	Prostanoid Biosynthesis	1/10 (10%)	9/10 (90%)	0,91	0,10	PTGS2
209	Cell Cycle Control of Chromosomal Replication	2/56 (4%)	54/56 (96%)	0,90	0,04	CDK6,PRIM1
210	Th1 Pathway	3/121 (2%)	118/121 (98%)	0,89	0,02	GRB2,IFNG,NOTCH2
211	Purine Nucleotides De Novo Biosynthesis II	1/11 (9%)	10/11 (91%)	0,87	0,09	ADSS2
212	Leukocyte Extravasation Signaling	4/197 (2%)	193/197 (98%)	0,87	0,02	CLDN12,ITGA2,MAP2K4,WIPF1
213	Hematopoiesis from Multipotent Stem Cells	1/12 (8%)	11/12 (92%)	0,84	0,08	KITLG
214	Guanosine Nucleotides Degradation III	1/12 (8%)	11/12 (92%)	0,84	0,08	PNP
215	Endocannabinoid Neuronal Synapse Pathway	3/128 (2%)	125/128 (98%)	0,84	0,02	CACNA2D1,MAPK3,PTGS2
216	Synaptic Long Term Potentiation	3/129 (2%)	126/129 (98%)	0,83	0,02	MAP2K1,MAPK3,RAF1
217	Activation of IRF by Cytosolic Pattern Recognition Receptors	2/63 (3%)	61/63 (97%)	0,83	0,03	JUN,MAP2K4
218	Urate Biosynthesis/Inosine 5'-phosphate Degradation	1/13 (8%)	12/13 (92%)	0,82	0,08	PNP
219	NAD Phosphorylation and Dephosphorylation	1/13 (8%)	12/13 (92%)	0,82	0,08	ACP2
220	Adipogenesis pathway	3/134 (2%)	131/134 (98%)	0,80	0,02	FGF2,FGFR1,GTF2H1
221	Leukotriene Biosynthesis	1/14 (7%)	13/14 (93%)	0,79	0,07	GSTM4
222	Iron homeostasis signaling pathway	3/137 (2%)	134/137 (98%)	0,79	0,02	EGFR,HMOX1,MAPK3
223	Role of JAK1 and JAK3 in γc Cytokine Signaling	2/69 (3%)	67/69 (97%)	0,77	0,03	GRB2,MAPK3
224	Choline Biosynthesis III	1/15 (7%)	14/15 (93%)	0,77	0,07	HMOX1
225	Adenosine Nucleotides Degradation II	1/15 (7%)	14/15 (93%)	0,77	0,07	PNP
226	Mismatch Repair in Eukaryotes	1/16 (6%)	15/16 (94%)	0,75	0,06	MSH2
227	Ephrin B Signaling	2/72 (3%)	70/72 (97%)	0,75	0,03	CFL2,MAPK3
228	Chondroitin Sulfate Degradation (Metazoa)	1/16 (6%)	15/16 (94%)	0,75	0,06	HYAL3
229	Caveolar-mediated Endocytosis Signaling	2/73 (3%)	71/73 (97%)	0,74	0,03	EGFR,ITGA2
230	TREM1 Signaling	2/75 (3%)	73/75 (97%)	0,73	0,03	GRB2,MAPK3
231	RAN Signaling	1/17 (6%)	16/17 (94%)	0,73	0,06	KPNA3
232	Ubiquinol-10 Biosynthesis (Eukaryotic)	1/17 (6%)	16/17 (94%)	0,73	0,06	ECHDC1
233	Dermatan Sulfate Degradation (Metazoa)	1/17 (6%)	16/17 (94%)	0,73	0,06	HYAL3
234	Toll-like Receptor Signaling	2/76 (3%)	74/76 (97%)	0,72	0,03	JUN,MAP2K4
235	Purine Nucleotides Degradation II (Aerobic)	1/18 (6%)	17/18 (94%)	0,71	0,06	PNP
236	VDR/RXR Activation	2/78 (3%)	76/78 (97%)	0,71	0,03	IFNG,WT1
237	DNA damage-induced 14-3-3σ Signaling	1/19 (5%)	18/19 (95%)	0,70	0,05	CCNE1
238	CDP-diacylglycerol Biosynthesis I	1/20 (5%)	19/20 (95%)	0,68	0,05	GPAM
239	Fatty Acid α-oxidation	1/20 (5%)	19/20 (95%)	0,68	0,05	PTGS2
240	Inflammasome pathway	1/20 (5%)	19/20 (95%)	0,68	0,05	PANX1
241	TR/RXR Activation	2/84 (2%)	82/84 (98%)	0,67	0,02	SLC16A3,UCP2
242	IL-4 Signaling	2/85 (2%)	83/85 (98%)	0,66	0,02	GRB2,HMGA1
243	Xenobiotic Metabolism AHR Signaling Pathway	2/85 (2%)	83/85 (98%)	0,66	0,02	GSTM4,HSP90B1
244	Tec Kinase Signaling	3/164 (2%)	161/164 (98%)	0,66	0,02	ITGA2,MAP2K4,RHOT1
245	Phosphatidylglycerol Biosynthesis II (Non-plastidic)	1/22 (5%)	21/22 (95%)	0,65	0,05	GPAM
246	CTLA4 Signaling in Cytotoxic T Lymphocytes	2/89 (2%)	87/89 (98%)	0,64	0,02	GRB2,PPP2R5C
247	Differential Regulation of Cytokine Production in Intestinal Epithelial Cells by IL-17A and IL-17F	1/23 (4%)	22/23 (96%)	0,64	0,04	IFNG
248	IL-1 Signaling	2/91 (2%)	89/91 (98%)	0,63	0,02	JUN,MAP2K4
249	Death Receptor Signaling	2/91 (2%)	89/91 (98%)	0,63	0,02	BCL2,MAP2K4
250	Th17 Activation Pathway	2/91 (2%)	89/91 (98%)	0,63	0,02	HSP90B1,IFNG
251	Fcγ Receptor-mediated Phagocytosis in Macrophages and Monocytes	2/94 (2%)	92/94 (98%)	0,61	0,02	HMOX1,MAPK3
252	GABA Receptor Signaling	2/95 (2%)	93/95 (98%)	0,60	0,02	CACNA2D1,KCNN4
253	NAD Salvage Pathway II	1/26 (4%)	25/26 (96%)	0,60	0,04	ACP2
254	Glycolysis I	1/26 (4%)	25/26 (96%)	0,60	0,04	TPI1
255	Apelin Liver Signaling Pathway	1/26 (4%)	25/26 (96%)	0,60	0,04	MAP2K4
256	Kinetochore Metaphase Signaling Pathway	2/101 (2%)	99/101 (98%)	0,57	0,02	PLK1,PPP2R5C
257	PD-1, PD-L1 cancer immunotherapy pathway	2/106 (2%)	104/106 (98%)	0,54	0,02	IFNG,PDCD4
258	Gαs Signaling	2/107 (2%)	105/107 (98%)	0,54	0,02	MAP2K1,MAPK3
259	Glutathione-mediated Detoxification	1/32 (3%)	31/32 (97%)	0,52	0,03	GSTM4
260	Cytotoxic T Lymphocyte-mediated Apoptosis of Target Cells	1/34 (3%)	33/34 (97%)	0,50	0,03	BCL2
261	Coagulation System	1/35 (3%)	34/35 (97%)	0,50	0,03	F2
262	DNA Methylation and Transcriptional Repression Signaling	1/35 (3%)	34/35 (97%)	0,50	0,03	H3-3A/H3-3B
263	Nucleotide Excision Repair Pathway	1/35 (3%)	34/35 (97%)	0,50	0,03	GTF2H1
264	Calcium Signaling	3/206 (1%)	203/206 (99%)	0,50	0,01	CACNA2D1,MAPK3,TPM3
265	Sphingosine-1-phosphate Signaling	2/117 (2%)	115/117 (98%)	0,49	0,02	MAPK3,RHOT1
266	p38 MAPK Signaling	2/118 (2%)	116/118 (98%)	0,48	0,02	H3-3A/H3-3B,MAP2K4
267	Docosahexaenoic Acid (DHA) Signaling	1/38 (3%)	37/38 (97%)	0,47	0,03	BCL2
268	AMPK Signaling	3/214 (1%)	211/214 (99%)	0,47	0,01	CCND1,PPP2R5C,RAB9B
269	Antiproliferative Role of TOB in T Cell Signaling	1/39 (3%)	38/39 (97%)	0,47	0,03	CCNE1
270	Inhibition of Matrix Metalloproteases	1/39 (3%)	38/39 (97%)	0,47	0,03	RECK
271	Antigen Presentation Pathway	1/39 (3%)	38/39 (97%)	0,47	0,03	IFNG
272	Phagosome Formation	2/125 (2%)	123/125 (98%)	0,46	0,02	ITGA2,RHOT1
273	Mechanisms of Viral Exit from Host Cells	1/41 (2%)	40/41 (98%)	0,45	0,02	PDCD6IP
274	LPS/IL-1 Mediated Inhibition of RXR Function	3/224 (1%)	221/224 (99%)	0,45	0,01	GSTM4,JUN,MAP2K4
275	Triacylglycerol Biosynthesis	1/42 (2%)	41/42 (98%)	0,45	0,02	GPAM
276	Apelin Pancreas Signaling Pathway	1/44 (2%)	43/44 (98%)	0,44	0,02	MAP2K4
277	FAT10 Cancer Signaling Pathway	1/46 (2%)	45/46 (98%)	0,42	0,02	IFNG
278	Ephrin A Signaling	1/47 (2%)	46/47 (98%)	0,42	0,02	CFL2
279	Hematopoiesis from Pluripotent Stem Cells	1/49 (2%)	48/49 (98%)	0,40	0,02	KITLG
280	Assembly of RNA Polymerase II Complex	1/50 (2%)	49/50 (98%)	0,40	0,02	GTF2H1
281	Lymphotoxin β Receptor Signaling	1/53 (2%)	52/53 (98%)	0,38	0,02	MAPK3
282	Role of Cytokines in Mediating Communication between Immune Cells	1/54 (2%)	53/54 (98%)	0,38	0,02	IFNG
283	Transcriptional Regulatory Network in Embryonic Stem Cells	1/54 (2%)	53/54 (98%)	0,38	0,02	H3-3A/H3-3B
284	Phagosome Maturation	2/151 (1%)	149/151 (99%)	0,38	0,01	NAPG,VTI1B
285	Gustation Pathway	2/154 (1%)	152/154 (99%)	0,37	0,01	CACNA2D1,PANX1
286	Glutamate Receptor Signaling	1/57 (2%)	56/57 (98%)	0,36	0,02	SLC38A1
287	Necroptosis Signaling Pathway	2/157 (1%)	155/157 (99%)	0,36	0,01	PPIF,TOMM34
288	MSP-RON Signaling Pathway	1/58 (2%)	57/58 (98%)	0,36	0,02	IFNG
289	Retinoic acid Mediated Apoptosis Signaling	1/60 (2%)	59/60 (98%)	0,35	0,02	IFNG
290	Phospholipases	1/64 (2%)	63/64 (98%)	0,33	0,02	HMOX1
291	Netrin Signaling	1/65 (2%)	64/65 (98%)	0,32	0,02	CACNA2D1
292	Eicosanoid Signaling	1/66 (2%)	65/66 (98%)	0,32	0,02	PTGS2
293	Remodeling of Epithelial Adherens Junctions	1/68 (1%)	67/68 (99%)	0,31	0,01	ZYX
294	ERK5 Signaling	1/72 (1%)	71/72 (99%)	0,30	0,01	EGFR
295	Granulocyte Adhesion and Diapedesis	2/180 (1%)	178/180 (99%)	0,30	0,01	CLDN12,ITGA2
296	T Helper Cell Differentiation	1/73 (1%)	72/73 (99%)	0,29	0,01	IFNG
297	Dendritic Cell Maturation	2/183 (1%)	181/183 (99%)	0,29	0,01	MAP2K4,MAPK3
298	Angiopoietin Signaling	1/75 (1%)	74/75 (99%)	0,29	0,01	GRB2
299	Dopamine Receptor Signaling	1/77 (1%)	76/77 (99%)	0,28	0,01	PPP2R5C
300	Xenobiotic Metabolism PXR Signaling Pathway	2/192 (1%)	190/192 (99%)	0,28	0,01	GSTM4,HSP90B1
301	Agranulocyte Adhesion and Diapedesis	2/193 (1%)	191/193 (99%)	0,27	0,01	CLDN12,ITGA2
302	Apelin Adipocyte Signaling Pathway	1/82 (1%)	81/82 (99%)	0,27	0,01	MAPK3
303	Regulation Of The Epithelial Mesenchymal Transition In Development Pathway	1/84 (1%)	83/84 (99%)	0,26	0,01	WNT3A
304	HIPPO signaling	1/85 (1%)	84/85 (99%)	0,26	0,01	PPP2R5C
305	Crosstalk between Dendritic Cells and Natural Killer Cells	1/89 (1%)	88/89 (99%)	0,25	0,01	IFNG
306	Communication between Innate and Adaptive Immune Cells	1/96 (1%)	95/96 (99%)	0,23	0,01	IFNG
307	Virus Entry via Endocytic Pathways	1/107 (1%)	106/107 (99%)	0,20	0,01	ITGA2
308	iCOS-iCOSL Signaling in T Helper Cells	1/111 (1%)	110/111 (99%)	0,19	0,01	GRB2
309	GPCR-Mediated Nutrient Sensing in Enteroendocrine Cells	1/112 (1%)	111/112 (99%)	0,19	0,01	CACNA2D1
310	LXR/RXR Activation	1/121 (1%)	120/121 (99%)	0,00	0,01	PTGS2
311	FXR/RXR Activation	1/126 (1%)	125/126 (99%)	0,00	0,01	MAP2K4
312	Dopamine-DARPP32 Feedback in cAMP Signaling	1/163 (1%)	162/163 (99%)	0,00	0,01	PPP2R5C
313	D-myo-inositol-5-phosphate Metabolism	1/157 (1%)	156/157 (99%)	0,00	0,01	CDC25A
314	D-myo-inositol (1,4,5,6)-Tetrakisphosphate Biosynthesis	1/142 (1%)	141/142 (99%)	0,00	0,01	CDC25A
315	Superpathway of Inositol Phosphate Compounds	1/199 (1%)	198/199 (99%)	0,00	0,01	CDC25A
316	D-myo-inositol (3,4,5,6)-tetrakisphosphate Biosynthesis	1/142 (1%)	141/142 (99%)	0,00	0,01	CDC25A
317	3-phosphoinositide Degradation	1/156 (1%)	155/156 (99%)	0,00	0,01	CDC25A
318	3-phosphoinositide Biosynthesis	1/166 (1%)	165/166 (99%)	0,00	0,01	CDC25A
319	GP6 Signaling Pathway	1/119 (1%)	118/119 (99%)	0,00	0,01	LAMC1

Type 1 diabetes 2-5 years of duration

	Ingenuity Canonical Pathways	Overlaps with dataset	No overlap with dataset		Ratio	Molecules
**1**	Senescence Pathway	69/275 (25%)	206/275 (75%)	29,6	0,3	M2,MTOR,NF1,NFATC1,NRAS,PCGF1,PIK3R1,PIK3R2,PIK3R3,PPP2R2A,PPP2R5C,PPP3CA,PTEN,RAF1,RB1,RBL2,RPS6KA5,SMAD1,SMAD2,SMAD3,SMAD4,SMAD5,SOD2,SQSTM1,TBK1,TGFB3,TGFBR1,TGFBR2,TP53,TRAF6,ZFP36L1
**2**	IL-6 Signaling	47/125 (38%)	78/125 (62%)	28,4	0,4	CS3,SRF,STAT3,TNF,TRAF6,VEGFA
**3**	NF-κB Signaling	50/179 (28%)	129/179 (72%)	23,7	0,3	R1,TLR10,TLR3,TLR4,TLR7,TLR9,TNF,TNFAIP3,TRA,TRAF6
**4**	Aryl Hydrocarbon Receptor Signaling	44/143 (31%)	99/143 (69%)	22,8	0,3	TYR
**5**	PTEN Signaling	41/126 (33%)	85/126 (67%)	22,2	0,3	LA,TGFBR1,TGFBR2
**6**	Acute Phase Response Signaling	48/179 (27%)	131/179 (73%)	22,0	0,3	S3,SOCS5,SOD2,STAT3,TNF,TRAF6
**7**	HOTAIR Regulatory Pathway	45/160 (28%)	115/160 (72%)	21,6	0,3	9,MMP1,MMP13,MMP14,MMP16,MMP3,MMP9,MYC,PIK3R1,PIK3R2,PIK3R3,PTEN,RCOR1,RELA,SRF,STAT3,TCF7L2,TLR4,VIM,WNT1,WNT3A,WNT7B,YY1
**8**	Glucocorticoid Receptor Signaling	65/336 (19%)	271/336 (81%)	21,5	0,2	NOS2,NRAS,NRIP1,PIK3R1,PIK3R2,PIK3R3,POLR2C,PPP3CA,PRKACB,PTGS2,RAF1,RELA,SMAD2,SMAD3,SMAD4,STAT1,STAT3,TAF9B,TGFB3,TGFBR1,TGFBR2,TNF,TRA,TRAF6,UBE2I,VCAM1
**9**	Epithelial-Mesenchymal Transition Pathway	49/192 (26%)	143/192 (74%)	21,5	0,3	TCF7L2,TGFB3,TGFBR1,TGFBR2,WNT1,WNT3A,WNT7B,ZEB1,ZEB2
**10**	IL-10 Signaling	30/69 (43%)	39/69 (57%)	20,4	0,4	CCR5,CD14,CHUK,FOS,HMOX1,IKBKE,IL10,IL1F10,IL1R1,IL1RAP,IL1RAPL2,IL1RL2,IL1RN,IL36A,IL36B,IL36G,IL36RN,IL37,IL6,JAK1,JUN,LBP,MAP2K4,MAPK11,RELA,SOCS3,SP1,STAT3,TNF,TRAF6
**11**	PI3K/AKT Signaling	45/175 (26%)	130/175 (74%)	19,9	0,3	R5C,PTEN,PTGS2,RAF1,RELA,RHEB,TP53
**12**	STAT3 Pathway	39/135 (29%)	96/135 (71%)	19,2	0,3	GFA
**13**	Role of PKR in Interferon Induction and Antiviral Response	36/117 (31%)	81/117 (69%)	18,7	0,3	APAF1,BAX,CASP3,CHUK,E2F1,FADD,FAS,FASLG,FOS,HSP90B1,HSPA14,HSPA1A/HSPA1B,IFNA1/IFNA13,IFNB1,IFNG,IKBKE,JAK1,JUN,MAP2K4,MAP2K7,MAPK11,MAPK3,MYD88,PDGFA,PDGFB,RELA,SP1,STAT1,STAT3,TAB2,TLR3,TLR4,TLR9,TNF,TP53,TRAF6
**14**	IGF-1 Signaling	34/104 (33%)	70/104 (67%)	18,6	0,3	AKT3,CCN1,CCN2,FOS,FOXO1,FOXO3,GRB10,GRB2,IGF1,IGF1R,IGFBP3,IRS1,JAK1,JUN,KRAS,MAP2K1,MAPK3,NEDD4,NRAS,PDPK1,PIK3R1,PIK3R2,PIK3R3,PRKACB,PRKCI,PTPN11,PXN,RAF1,RASA1,SOCS1,SOCS3,SOCS5,SRF,STAT3
**15**	Cell Cycle: G1/S Checkpoint Regulation	28/67 (42%)	39/67 (58%)	18,6	0,4	ABL1,BMI1,CCND1,CCND2,CCND3,CCNE1,CCNE2,CDC25A,CDK4,CDK6,CDKN1A,CDKN1B,CDKN2A,E2F1,E2F2,E2F3,FOXO1,GSK3B,HDAC4,MDM2,MYC,PA2G4,RB1,RBL2,SMAD3,SMAD4,TGFB3,TP53
**16**	p38 MAPK Signaling	35/118 (30%)	83/118 (70%)	17,6	0,3	ATF4,CREB1,DUSP1,FADD,FAS,FASLG,H3-3A/H3-3B,IL1F10,IL1R1,IL1RAP,IL1RAPL2,IL1RL2,IL1RN,IL36A,IL36B,IL36G,IL36RN,IL37,IRAK1,IRAK2,MAP2K4,MAPK11,MEF2C,MEF2D,MYC,RPS6KA5,SRF,STAT1,TAB2,TGFB3,TGFBR1,TGFBR2,TNF,TP53,TRAF6
**17**	Cholecystokinin/Gastrin-mediated Signaling	35/119 (29%)	84/119 (71%)	17,5	0,3	CCKBR,CDC42,DIRAS3,EGFR,FOS,GNA13,GRB2,IL1F10,IL1RN,IL36A,IL36B,IL36G,IL36RN,IL37,JUN,KRAS,MAP2K1,MAP2K4,MAPK3,MAPK7,MEF2C,MEF2D,NRAS,PRKCA,PRKCI,PTGS2,PXN,RAF1,RHOA,RHOB,RHOG,RHOT1,SRC,SRF,TNF
**18**	Dendritic Cell Maturation	43/183 (23%)	140/183 (77%)	17,5	0,2	,TRA,TRAF6
**19**	Toll-like Receptor Signaling	28/76 (37%)	48/76 (63%)	16,9	0,4	CD14,CHUK,FOS,IL1F10,IL1RN,IL36A,IL36B,IL36G,IL36RN,IL37,IRAK1,IRAK2,JUN,LBP,MAP2K4,MAPK11,MYD88,RELA,TAB2,TLR1,TLR10,TLR3,TLR4,TLR7,TLR9,TNF,TNFAIP3,TRAF6
**20**	HMGB1 Signaling	40/165 (24%)	125/165 (76%)	16,8	0,2	AKT3,CD40LG,CDC42,CXCL8,DIRAS3,FASLG,FOS,ICAM1,IFNG,IL1F10,IL1R1,IL36A,IL36B,IL36G,IL37,IL6,JUN,KRAS,LIF,LTB,MAP2K1,MAP2K4,MAP2K7,MAPK11,MAPK3,NRAS,PIK3R1,PIK3R2,PIK3R3,RELA,RHOA,RHOB,RHOG,RHOT1,SP1,TGFB3,TLR4,TNF,TNFSF9,VCAM1
**21**	IL-8 Signaling	44/200 (22%)	156/200 (78%)	16,8	0,2	RAF6,VCAM1,VEGFA
**22**	PPAR Signaling	32/104 (31%)	72/104 (69%)	16,7	0,3	CHUK,FOS,GRB2,HSP90B1,IKBKE,IL1F10,IL1R1,IL1RAP,IL1RAPL2,IL1RL2,IL1RN,IL36A,IL36B,IL36G,IL36RN,IL37,JUN,KRAS,MAP2K1,MAPK3,NRAS,NRIP1,PDGFA,PDGFB,PDGFRA,PPARG,PTGS2,RAF1,RELA,RXRA,TNF,TRAF6
**23**	IL-7 Signaling Pathway	28/78 (36%)	50/78 (64%)	16,6	0,4	AKT3,BAK1,BAX,BCL2,CCND1,CCND3,CDC25A,CDKN1B,FOXO1,FOXO3,GRB2,GSK3B,IFNG,IL2RG,JAK1,JUN,MAPK11,MAPK3,MCL1,MET,MYC,NFATC1,PDPK1,PIK3R1,PIK3R2,PIK3R3,SOCS1,STAT1
**24**	Th1 and Th2 Activation Pathway	40/171 (23%)	131/171 (77%)	16,2	0,2	A
**25**	FAT10 Cancer Signaling Pathway	22/46 (48%)	24/46 (52%)	16,2	0,5	ACVR1,ACVR2A,AKT3,BMPR2,CHUK,CTNNB1,CXCR4,GSK3B,IFNG,IKBKE,IL6,MAD2L1,RELA,SMAD2,SMAD3,SMAD4,STAT3,TGFB3,TGFBR1,TGFBR2,TNF,TP53
**26**	ILK Signaling	42/190 (22%)	148/190 (78%)	16,1	0,2	VIM
**27**	Estrogen Receptor Signaling	55/328 (17%)	273/328 (83%)	15,5	0,2	PIK3R3,PRKACB,PRKCA,PRKCI,PTEN,RAF1,RELA,RHOA,RUNX2,SOD2,SP1,SRC,TP53,VEGFA
**28**	JAK/Stat Signaling	27/80 (34%)	53/80 (66%)	15,2	0,3	AKT3,BCL2L1,CCKBR,CDKN1A,CEBPB,FOS,GRB2,IL6,JAK1,JUN,KRAS,MAP2K1,MAPK3,MTOR,NRAS,PIAS3,PIK3R1,PIK3R2,PIK3R3,PTPN11,RAF1,RELA,SOCS1,SOCS3,SOCS5,STAT1,STAT3
**29**	T Cell Exhaustion Signaling Pathway	39/175 (22%)	136/175 (78%)	15,1	0,2	FA
30	HIF1α Signaling	42/205 (20%)	163/205 (80%)	14,9	0,2	3,VEGFA,VIM
31	HGF Signaling	31/111 (28%)	80/111 (72%)	14,9	0,3	AKT3,CCND1,CDC42,CDKN1A,CDKN2A,CRKL,ETS1,FOS,GRB2,IL6,ITGA2,ITGA5,JUN,KRAS,MAP2K1,MAP2K4,MAP2K7,MAP3K12,MAPK3,MET,NRAS,PIK3R1,PIK3R2,PIK3R3,PRKCA,PRKCI,PTGS2,PTPN11,PXN,RAF1,STAT3
32	Human Embryonic Stem Cell Pluripotency	34/135 (25%)	101/135 (75%)	14,9	0,3	ACVR1,AKT3,BDNF,BMPR2,CTNNB1,FGF2,FGFR1,FGFR3,FOXO1,GSK3B,LEFTY1,LEFTY2,NTRK3,PDGFA,PDGFB,PDGFRA,PDPK1,PIK3R1,PIK3R2,PIK3R3,S1PR1,SMAD1,SMAD2,SMAD3,SMAD4,SMAD5,SMO,TCF7L2,TGFB3,TGFBR1,TGFBR2,WNT1,WNT3A,WNT7B
33	PDGF Signaling	27/86 (31%)	59/86 (69%)	14,4	0,3	ABL1,ACP1,CRKL,FOS,GRB2,INPP5D,JAK1,JUN,KRAS,MAP2K1,MAP2K4,MAPK3,MYC,NRAS,PDGFA,PDGFB,PDGFRA,PIK3R1,PIK3R2,PIK3R3,PRKCA,RAF1,RASA1,SRC,SRF,STAT1,STAT3
34	IL-12 Signaling and Production in Macrophages	33/132 (25%)	99/132 (75%)	14,4	0,3	AKT3,CD40,CD40LG,CEBPB,CHUK,FOS,IFNA1/IFNA13,IFNG,IKBKE,IL10,IL12RB2,JUN,MAF,MAP2K1,MAP2K4,MAPK11,MAPK3,MYD88,NOS2,PIK3R1,PIK3R2,PIK3R3,PPARG,PRKCA,PRKCI,RELA,RXRA,SPI1,STAT1,TGFB3,TLR4,TNF,TRAF6
35	EGF Signaling	22/55 (40%)	33/55 (60%)	14,2	0,4	AKT3,EGFR,FOS,GRB2,JAK1,JUN,MAP2K1,MAP2K4,MAP2K7,MAPK11,MAPK3,MTOR,PIK3R1,PIK3R2,PIK3R3,PRKCA,RAF1,RASA1,SRC,SRF,STAT1,STAT3
36	Cyclins and Cell Cycle Regulation	26/81 (32%)	55/81 (68%)	14,1	0,3	ABL1,CCNA2,CCND1,CCND2,CCND3,CCNE1,CCNE2,CDC25A,CDK4,CDK6,CDKN1A,CDKN1B,CDKN2A,E2F1,E2F2,E2F3,GSK3B,HDAC4,PA2G4,PPP2R2A,PPP2R5C,RAF1,RB1,TGFB3,TP53,WEE1
37	Myc Mediated Apoptosis Signaling	21/50 (42%)	29/50 (58%)	14,1	0,4	APAF1,BAX,BBC3,BCL2,BCL2L1,BCL2L11,CASP3,CDKN2A,CFLAR,CHUK,FADD,FAS,FASLG,IKBKE,MCL1,MDM2,MYC,PMAIP1,PRKACB,TNF,TP53
38	Mouse Embryonic Stem Cell Pluripotency	29/103 (28%)	74/103 (72%)	14,1	0,3	AKT3,BMPR2,CTNNB1,GRB2,GSK3B,ID1,ID2,ID3,JAK1,KRAS,LIF,MAP2K1,MAPK11,MAPK3,MYC,NRAS,PIK3R1,PIK3R2,PIK3R3,PTPN11,RAF1,SMAD1,SMAD4,SMAD5,SMO,STAT3,TCF7L2,TP53,WNT3A
39	Neuregulin Signaling	28/96 (29%)	68/96 (71%)	14,1	0,3	ADAM17,AKT3,CDKN1B,CRKL,EGFR,ERBB2,ERBB3,ERBB4,GRB2,HSP90B1,ITGA2,ITGA5,KRAS,MAP2K1,MAPK3,MTOR,MYC,NRAS,PDPK1,PIK3R1,PIK3R2,PIK3R3,PRKCA,PRKCI,PTEN,PTPN11,RAF1,SRC
40	PEDF Signaling	26/82 (32%)	56/82 (68%)	14,0	0,3	AKT3,BCL2,BCL2L1,BDNF,CASP7,CFLAR,CHUK,FAS,FASLG,IKBKE,KRAS,MAPK11,MAPK3,NRAS,PIK3R1,PIK3R2,PIK3R3,PPARG,RAF1,RELA,RHOA,SOD2,SRF,TCF7L2,TP53,ZEB1
41	Role of Pattern Recognition Receptors in Recognition of Bacteria and Viruses	35/154 (23%)	119/154 (77%)	13,9	0,2	CD40LG,CREB1,CXCL8,FASLG,IFNA1/IFNA13,IFNB1,IFNG,IL10,IL1F10,IL36A,IL36B,IL36G,IL37,IL6,LIF,LTB,MAP2K4,MAPK3,MYD88,PIK3R1,PIK3R2,PIK3R3,PRKCA,PRKCI,RELA,RNASEL,TGFB3,TLR1,TLR3,TLR4,TLR7,TLR9,TNF,TNFSF9,TRAF6
42	Th2 Pathway	32/136 (24%)	104/136 (76%)	13,2	0,2	ACVR1,ACVR2A,BMPR2,CCR3,CCR5,CD40,CXCR4,GRB2,ICAM1,IFNG,IKZF1,IL10,IL12RB2,IL2RG,JAG1,JAK1,JUN,MAF,NOTCH1,NOTCH2,NOTCH4,PIK3R1,PIK3R2,PIK3R3,RUNX3,S1PR1,SOCS3,SPI1,TBX21,TGFBR1,TGFBR2,TRA
43	TGF-β Signaling	27/96 (28%)	69/96 (72%)	13,1	0,3	ACVR1,ACVR2A,BCL2,BMPR1B,BMPR2,CDC42,FOS,GRB2,JUN,KRAS,MAP2K1,MAP2K4,MAPK11,MAPK3,NRAS,RAF1,RUNX2,RUNX3,SMAD1,SMAD2,SMAD3,SMAD4,SMAD5,TGFB3,TGFBR1,TGFBR2,TRAF6
44	Apoptosis Signaling	27/99 (27%)	72/99 (73%)	12,8	0,3	APAF1,BAK1,BAX,BCL2,BCL2L1,BCL2L11,CAPN8,CASP3,CASP6,CASP7,CHUK,DFFA,FAS,FASLG,IKBKE,KRAS,MAP2K1,MAP2K4,MAP2K7,MAPK3,MCL1,NRAS,PRKCA,RAF1,RELA,TNF,TP53
45	Prolactin Signaling	24/81 (30%)	57/81 (70%)	12,2	0,3	CEBPB,FOS,GRB2,IRS1,JUN,KRAS,MAP2K1,MAPK3,MYC,NRAS,PDPK1,PIK3R1,PIK3R2,PIK3R3,PRKCA,PRKCI,PTPN11,RAF1,SOCS1,SOCS3,SOCS5,SP1,STAT1,STAT3
46	Leukocyte Extravasation Signaling	37/197 (19%)	160/197 (81%)	12,0	0,2	ABL1,ACTA2,CD44,CDC42,CDH5,CLDN1,CLDN12,CLDN18,CRKL,CTNNB1,CTNND1,CXCR4,F11R,ICAM1,ITGA2,ITGA5,ITGB3,MAP2K4,MAPK11,MMP1,MMP13,MMP14,MMP16,MMP3,MMP9,PIK3R1,PIK3R2,PIK3R3,PRKCA,PRKCI,PTPN11,PXN,RHOA,SRC,TIMP3,VCAM1,WIPF1
47	Adrenomedullin signaling pathway	37/197 (19%)	160/197 (81%)	12,0	0,2	AKT3,BAX,BCL2,CASP3,CEBPB,CFH,FOS,GPR37,GRB2,GSK3B,HIF1A,IL1F10,IL1RN,IL36A,IL36B,IL36G,IL36RN,IL37,KCNN4,KRAS,MAP2K1,MAP2K4,MAP2K7,MAPK11,MAPK3,MAPK7,NPR3,NRAS,PIK3R1,PIK3R2,PIK3R3,PPARG,PRKACB,RAF1,RELA,RXRA,TNF
48	PPARα/RXRα Activation	36/190 (19%)	154/190 (81%)	11,8	0,2	ACVR1,ACVR2A,BMPR2,CHUK,GPD1,GRB2,HSP90B1,IKBKE,IL1R1,IL1RAP,IL1RAPL2,IL1RL2,IL6,IRS1,JUN,KRAS,LPL,MAP2K1,MAP2K4,MAP2K7,MAPK3,MEF2C,NCOA3,NRAS,PRKACB,PRKCA,RAF1,RELA,RXRA,SMAD2,SMAD3,SMAD4,TGFB3,TGFBR1,TGFBR2,TRAF6
49	iNOS Signaling	18/45 (40%)	27/45 (60%)	11,7	0,4	CD14,CHUK,FOS,HMGA1,IFNG,IKBKE,IRAK1,IRAK2,JAK1,JUN,LBP,MAPK11,MYD88,NOS2,RELA,STAT1,TLR4,TRAF6
50	ErbB Signaling	25/94 (27%)	69/94 (73%)	11,6	0,3	CDC42,EGFR,ERBB2,ERBB3,ERBB4,FOS,FOXO1,GRB2,GSK3B,JUN,KRAS,MAP2K1,MAP2K4,MAPK11,MAPK3,MTOR,NRAS,PAK5,PDPK1,PIK3R1,PIK3R2,PIK3R3,PRKCA,PRKCI,RAF1
51	Role of NANOG in Mammalian Embryonic Stem Cell Pluripotency	28/119 (24%)	91/119 (76%)	11,6	0,2	AKT3,BMPR1B,BMPR2,CDX2,CTNNB1,GATA6,GRB2,GSK3B,JAK1,KRAS,LIF,MAP2K1,MAPK3,NRAS,PIK3R1,PIK3R2,PIK3R3,RAF1,SMAD1,SMAD4,SMAD5,SMO,STAT3,TCL1A,TP53,WNT1,WNT3A,WNT7B
52	ErbB2-ErbB3 Signaling	21/65 (32%)	44/65 (68%)	11,6	0,3	CCND1,CDKN1B,ERBB2,ERBB3,FOXO1,GRB2,GSK3B,JUN,KRAS,MAP2K1,MAPK3,MYC,NRAS,PDPK1,PIK3R1,PIK3R2,PIK3R3,PTEN,RAF1,SP1,STAT3
53	Axonal Guidance Signaling	61/484 (13%)	423/484 (87%)	11,6	0,1	B,PIK3R1,PIK3R2,PIK3R3,PLXND1,PPP3CA,PRKACB,PRKCA,PRKCI,PTPN11,PXN,RAF1,RASA1,RHOA,RTN4,SDCBP,SMO,TUBB2A,VEGFA,WIPF1,WNT1,WNT3A,WNT7B
54	B Cell Receptor Signaling	35/185 (19%)	150/185 (81%)	11,5	0,2	ABL1,AKT3,ATF4,BCL2L1,CDC42,CFL2,CHUK,CREB1,ETS1,FOXO1,GRB2,GSK3B,IKBKE,INPP5D,JUN,KRAS,MAP2K1,MAP2K4,MAP2K7,MAP3K12,MAPK11,MAPK3,MEF2C,MTOR,NFATC1,NRAS,PDPK1,PIK3R1,PIK3R2,PIK3R3,PPP3CA,PTEN,PTPN11,RAF1,RELA
55	Communication between Innate and Adaptive Immune Cells	25/96 (26%)	71/96 (74%)	11,4	0,3	Ccl9,CD40,CD40LG,CD83,CXCL8,IFNA1/IFNA13,IFNB1,IFNG,IL10,IL1F10,IL1RN,IL36A,IL36B,IL36G,IL36RN,IL37,IL6,TLR1,TLR10,TLR3,TLR4,TLR7,TLR9,TNF,TRA
56	Estrogen-mediated S-phase Entry	14/26 (54%)	12/26 (46%)	11,3	0,5	CCNA2,CCND1,CCNE1,CCNE2,CDC25A,CDK4,CDKN1A,CDKN1B,E2F1,E2F2,E2F3,ESR1,MYC,RB1
57	NGF Signaling	27/114 (24%)	87/114 (76%)	11,3	0,2	AKT3,ATF4,BAX,CDC42,CHUK,CREB1,GRB2,IKBKE,KRAS,MAP2K1,MAP2K4,MAP3K12,MAPK3,MAPK7,NRAS,PDPK1,PIK3R1,PIK3R2,PIK3R3,PTPN11,RAF1,RELA,RHOA,RHOG,RPS6KA5,TP53,TRAF6
58	LPS-stimulated MAPK Signaling	23/82 (28%)	59/82 (72%)	11,3	0,3	CD14,CDC42,CHUK,CREB1,FOS,IKBKE,JUN,KRAS,LBP,MAP2K1,MAP2K4,MAPK11,MAPK3,NRAS,PIK3R1,PIK3R2,PIK3R3,PRKCA,PRKCI,RAF1,RELA,SRF,TLR4
59	Germ Cell-Sertoli Cell Junction Signaling	33/171 (19%)	138/171 (81%)	11,1	0,2	ACTA2,CDC42,CFL2,CLINT1,CTNNB1,CTNND1,DIRAS3,ITGA2,KRAS,MAP2K1,MAP2K4,MAP2K7,MAP3K12,MAPK3,NRAS,PAK5,PDPK1,PIK3R1,PIK3R2,PIK3R3,PXN,RHOA,RHOB,RHOG,RHOT1,SRC,TGFB3,TGFBR1,TGFBR2,TJP1,TNF,TUBB2A,ZYX
60	Neurotrophin/TRK Signaling	22/76 (29%)	54/76 (71%)	11,1	0,3	ATF4,BDNF,CDC42,CREB1,FOS,GRB2,JUN,KRAS,MAP2K1,MAP2K4,MAP2K7,MAPK3,NRAS,NTRK3,PDPK1,PIK3R1,PIK3R2,PIK3R3,PTPN11,RAF1,SPRY1,SPRY2
61	Natural Killer Cell Signaling	35/197 (18%)	162/197 (82%)	10,7	0,2	AKT3,CDC42,CFL2,COL1A1,COL1A2,COL3A1,COL5A3,FASLG,GRB2,HSPA14,HSPA1A/HSPA1B,IFNG,IL12RB2,KIR2DL1/KIR2DL3,KIR2DL2,KRAS,MAP2K1,MAP3K12,MAPK11,MAPK3,MICA,MTOR,MYD88,NFATC1,NRAS,PAK5,PIK3R1,PIK3R2,PIK3R3,PTPN11,PXN,RAF1,RELA,TRAF6,WIPF1

62	Gα12/13 Signaling	28/130 (22%)	102/130 (78%)	10,7	0,2	AKT3,CDC42,CDH5,CHUK,CTNNB1,F2,GNA13,IKBKE,JUN,KRAS,MAP2K1,MAP2K4,MAP2K7,MAPK3,MAPK7,MEF2C,MEF2D,NRAS,PIK3R1,PIK3R2,PIK3R3,PXN,RAF1,RASA1,RELA,RHOA,SRC,TBXA2R
63	CD40 Signaling	20/65 (31%)	45/65 (69%)	10,6	0,3	CD40,CD40LG,CHUK,FOS,ICAM1,IKBKE,JUN,MAP2K1,MAP2K4,MAP2K7,MAPK11,MAPK3,PIK3R1,PIK3R2,PIK3R3,PTGS2,RELA,STAT3,TNFAIP3,TRAF6
64	IL-17 Signaling	22/80 (28%)	58/80 (73%)	10,6	0,3	AKT3,CEBPB,CRP,CXCL8,GSK3B,IL6,JAK1,JUN,KRAS,MAP2K1,MAP2K4,MAPK11,MAPK3,MMP3,NOS2,NRAS,PIK3R1,PIK3R2,PIK3R3,PTGS2,RELA,TRAF6
65	FLT3 Signaling in Hematopoietic Progenitor Cells	22/80 (28%)	58/80 (73%)	10,6	0,3	AKT3,ATF4,CREB1,EIF4E,FLT3,GRB2,INPP5D,KRAS,MAP2K1,MAPK11,MAPK3,MTOR,NRAS,PDPK1,PIK3R1,PIK3R2,PIK3R3,PTPN11,RAF1,RPS6KA5,STAT1,STAT3
66	Insulin Receptor Signaling	29/140 (21%)	111/140 (79%)	10,6	0,2	AKT3,CRKL,EIF4E,FOXO1,FOXO3,GRB10,GRB2,GSK3B,GYS1,INPP5D,IRS1,JAK1,KRAS,MAP2K1,MAPK3,MTOR,NRAS,PDPK1,PIK3R1,PIK3R2,PIK3R3,PPP1R7,PRKACB,PRKCI,PTEN,PTPN11,RAF1,RPTOR,SOCS3
67	Crosstalk between Dendritic Cells and Natural Killer Cells	23/89 (26%)	66/89 (74%)	10,5	0,3	ACTA2,CD40,CD40LG,CD69,CD83,FAS,FASLG,FSCN1,IFNA1/IFNA13,IFNB1,IFNG,IL2RG,IL6,KIR2DL1/KIR2DL3,KIR2DL2,LTB,MICA,RELA,TLR3,TLR4,TLR7,TLR9,TNF
68	Endocannabinoid Developing Neuron Pathway	26/115 (23%)	89/115 (77%)	10,4	0,2	AKT3,ATF4,BRCA1,CCND1,CDKN1B,CREB1,CTNNB1,GSK3B,KRAS,MAP2K1,MAP2K4,MAP2K7,MAPK11,MAPK3,MAPK7,MTOR,NRAS,PIK3R1,PIK3R2,PIK3R3,PRKACB,RAF1,RHOA,RPTOR,SRC,STAT3
69	Telomerase Signaling	25/107 (23%)	82/107 (77%)	10,4	0,2	ABL1,AKT3,CDKN1A,E2F1,EGFR,ETS1,GRB2,HDAC4,HSP90B1,IL2RG,KRAS,MAP2K1,MAPK3,MYC,NRAS,PDPK1,PIK3R1,PIK3R2,PIK3R3,PPP2R2A,PPP2R5C,RAF1,RB1,SP1,TP53
70	TREM1 Signaling	21/75 (28%)	54/75 (72%)	10,3	0,3	AKT3,CD40,CD83,CXCL8,GRB2,ICAM1,IL10,IL6,IRAK1,ITGA5,MAPK3,MYD88,RELA,STAT3,TLR1,TLR10,TLR3,TLR4,TLR7,TLR9,TNF
71	FGF Signaling	22/84 (26%)	62/84 (74%)	10,2	0,3	AKT3,ATF4,CREB1,CRKL,FGF16,FGF2,FGF7,FGFR1,FGFR3,GRB2,MAP2K1,MAPK11,MAPK3,MET,PIK3R1,PIK3R2,PIK3R3,PRKCA,PTPN11,RAF1,RPS6KA5,STAT3
72	PI3K Signaling in B Lymphocytes	28/138 (20%)	110/138 (80%)	10,0	0,2	ABL1,AKT3,ATF4,ATF6,CD40,CHUK,CREB1,FOS,FOXO3,IKBKE,INPP5D,IRS1,JUN,KRAS,MAP2K1,MAPK3,NFATC1,NRAS,PDPK1,PIK3R1,PIK3R2,PLEKHA4,PPP3CA,PRKCI,PTEN,RAF1,RELA,TLR4
73	Production of Nitric Oxide and Reactive Oxygen Species in Macrophages	33/188 (18%)	155/188 (82%)	10,0	0,2	AKT3,CDC42,CHUK,DIRAS3,FOS,IFNG,IKBKE,JAK1,JUN,MAP2K1,MAP2K4,MAP2K7,MAP3K12,MAPK11,MAPK3,NOS2,PIK3R1,PIK3R2,PIK3R3,PPP1R7,PPP2R2A,PPP2R5C,PRKCA,PRKCI,RELA,RHOA,RHOB,RHOG,RHOT1,SPI1,STAT1,TLR4,TNF
74	Sumoylation Pathway	24/103 (23%)	79/103 (77%)	10,0	0,2	AR,ARHGDIA,CDC42,DIRAS3,DNMT3A,ETS1,FAS,FASLG,FOS,JUN,MAP2K4,MDM2,MYB,RCOR1,RHOA,RHOB,RHOG,RHOT1,SMAD4,SP1,TDG,TP53,UBE2I,ZEB1
75	LXR/RXR Activation	26/121 (21%)	95/121 (79%)	9,9	0,2	CD14,CYP51A1,IL1F10,IL1R1,IL1RAP,IL1RAPL2,IL1RL2,IL1RN,IL36A,IL36B,IL36G,IL36RN,IL37,IL6,LBP,LPL,MMP9,MYLIP,NOS2,PTGS2,RELA,RXRA,SCD,TLR3,TLR4,TNF
76	IL-15 Production	26/121 (21%)	95/121 (79%)	9,9	0,2	ABL1,CSF1R,DDR1,DSTYK,EGFR,ERBB2,ERBB3,ERBB4,FGFR1,FGFR3,IFNA1/IFNA13,IFNB1,IGF1R,IL6,JAK1,KIT,MAP2K1,MAP2K4,MAP2K7,MET,NTRK3,PDGFRA,PRKCI,RELA,SRC,STAT1
77	Th1 Pathway	26/121 (21%)	95/121 (79%)	9,9	0,2	CCR5,CD40,CD40LG,GRB2,ICAM1,IFNA1/IFNA13,IFNG,IL10,IL12RB2,IL6,IL6R,JAK1,NFATC1,NOTCH1,NOTCH2,NOTCH4,PIK3R1,PIK3R2,PIK3R3,RUNX3,SOCS1,SOCS3,STAT1,STAT3,TBX21,TRA
78	Growth Hormone Signaling	20/71 (28%)	51/71 (72%)	9,9	0,3	CSHL1,FOS,IGF1,IGF1R,IGFBP3,IRS1,MAPK3,PDPK1,PIK3R1,PIK3R2,PIK3R3,PRKCA,PRKCI,RPS6KA5,SOCS1,SOCS3,SOCS5,SRF,STAT1,STAT3
79	IL-3 Signaling	21/79 (27%)	58/79 (73%)	9,9	0,3	AKT3,CRKL,FOS,FOXO1,GRB2,INPP5D,JAK1,JUN,KRAS,MAP2K1,MAPK3,NRAS,PIK3R1,PIK3R2,PIK3R3,PPP3CA,PRKCA,PRKCI,RAF1,STAT1,STAT3
80	Granulocyte Adhesion and Diapedesis	32/180 (18%)	148/180 (82%)	9,9	0,2	Ccl6,CCL8,Ccl9,CDH5,CLDN1,CLDN12,CLDN18,CXCL8,CXCR4,ICAM1,IL1F10,IL1R1,IL1RAP,IL1RAPL2,IL1RL2,IL1RN,IL36A,IL36B,IL36G,IL36RN,IL37,ITGA2,ITGA5,ITGB3,MMP1,MMP13,MMP14,MMP16,MMP3,MMP9,TNF,VCAM1
81	Integrin Signaling	35/213 (16%)	178/213 (84%)	9,8	0,2	ABL1,ACTA2,AKT3,ARPC3,CAPN8,CDC42,CRKL,DIRAS3,GIT1,GRB2,GSK3B,ITGA2,ITGA5,ITGB3,KRAS,MAP2K1,MAP2K4,MAPK3,NRAS,PAK5,PDGFB,PIK3R1,PIK3R2,PIK3R3,PTEN,PXN,RAF1,RHOA,RHOB,RHOG,RHOT1,SRC,TSPAN3,WIPF1,ZYX
82	Regulation of IL-2 Expression in Activated and Anergic T Lymphocytes	22/89 (25%)	67/89 (75%)	9,7	0,2	CHUK,FOS,GRB2,IKBKE,JUN,KRAS,MAP2K1,MAP2K4,MAP2K7,MAPK3,NFATC1,NRAS,PPP3CA,RAF1,RELA,SMAD2,SMAD3,SMAD4,TGFB3,TGFBR1,TGFBR2,TRA
83	Tec Kinase Signaling	30/164 (18%)	134/164 (82%)	9,6	0,2	ACTA2,CDC42,DIRAS3,FADD,FAS,FASLG,FOS,GNA13,ITGA2,ITGA5,JAK1,MAP2K4,PAK5,PIK3R1,PIK3R2,PIK3R3,PRKCA,PRKCI,RELA,RHOA,RHOB,RHOG,RHOT1,SRC,STAT1,STAT3,TLR4,TNF,TNFRSF10A,TRA
84	UVB-Induced MAPK Signaling	17/52 (33%)	35/52 (67%)	9,6	0,3	EGFR,EIF4E,FOS,H3-3A/H3-3B,JUN,MAP2K1,MAP2K4,MAPK11,MAPK3,MTOR,PIK3R1,PIK3R2,PIK3R3,PRKCA,PRKCI,RPS6KA5,TP53
85	Macropinocytosis Signaling	20/76 (26%)	56/76 (74%)	9,4	0,3	ANKFY1,CD14,CDC42,CSF1,CSF1R,ITGA5,ITGB3,KRAS,MET,NRAS,PDGFA,PDGFB,PIK3R1,PIK3R2,PIK3R3,PRKCA,PRKCI,RAB34,RHOA,SRC
86	Ephrin Receptor Signaling	31/180 (17%)	149/180 (83%)	9,3	0,2	ABL1,ACP1,AKT3,ARPC3,ATF4,CDC42,CFL2,CREB1,CRKL,CXCR4,GNA13,GRB2,ITGA2,ITGA5,KRAS,MAP2K1,MAPK3,NRAS,PAK5,PDGFA,PDGFB,PTPN11,PXN,RAF1,RASA1,RHOA,SDCBP,SRC,STAT3,VEGFA,WIPF1
87	VDR/RXR Activation	20/78 (26%)	58/78 (74%)	9,2	0,3	CAMP,CD14,CDKN1A,CDKN1B,CEBPB,COL13A1,FOXO1,IFNG,IGFBP3,KLF4,NCOA3,PDGFA,PRKCA,PRKCI,RUNX2,RXRA,SP1,TRPV6,WT1,YY1
88	Role of JAK family kinases in IL-6-type Cytokine Signaling	12/25 (48%)	13/25 (52%)	9,1	0,5	IL6,IL6R,JAK1,MAP2K4,MAPK11,MAPK3,OSMR,PTPN11,SOCS1,SOCS3,STAT1,STAT3
89	Sertoli Cell-Sertoli Cell Junction Signaling	31/185 (17%)	154/185 (83%)	9,0	0,2	ACTA2,AKT3,CDC42,CLDN1,CLDN12,CLDN18,CLINT1,CTNNB1,F11R,GSK3B,ITGA2,ITGA5,JUN,KRAS,MAP2K1,MAP2K4,MAP2K7,MAP3K12,MAPK11,MAPK3,NOS2,NRAS,PRKACB,PTEN,RAF1,SPTB,SRC,TGFB3,TJP1,TNF,TUBB2A
90	PAK Signaling	22/97 (23%)	75/97 (77%)	9,0	0,2	CASP3,CDC42,CFL2,GIT1,GRB2,ITGA2,ITGA5,KRAS,MAP2K1,MAP2K4,MAPK3,NRAS,PAK5,PDGFA,PDGFB,PDGFRA,PIK3R1,PIK3R2,PIK3R3,PXN,RAF1,TNF
91	TNFR1 Signaling	16/50 (32%)	34/50 (68%)	8,9	0,3	APAF1,CASP3,CASP6,CASP7,CDC42,CHUK,FADD,FOS,IKBKE,JUN,MAP2K4,PAK5,RELA,RIPK1,TNF,TNFAIP3
92	VEGF Signaling	22/99 (22%)	77/99 (78%)	8,8	0,2	ACTA2,AKT3,BCL2,BCL2L1,ELAVL1,FOXO1,FOXO3,GRB2,HIF1A,KRAS,MAP2K1,MAPK3,NRAS,PIK3R1,PIK3R2,PIK3R3,PRKCA,PTPN11,PXN,RAF1,SRC,VEGFA
93	Death Receptor Signaling	21/91 (23%)	70/91 (77%)	8,7	0,2	ACTA2,APAF1,BCL2,CASP3,CASP6,CASP7,CFLAR,CHUK,DFFA,FADD,FAS,FASLG,IKBKE,MAP2K4,MAP2K7,PARP8,RELA,RIPK1,TBK1,TNF,TNFRSF10A
94	IL-15 Signaling	19/75 (25%)	56/75 (75%)	8,6	0,3	AKT3,BCL2L1,CXCL8,IL2RG,JAK1,KRAS,MAP2K1,MAPK11,MAPK3,MTOR,MYC,NRAS,PIK3R1,PIK3R2,PIK3R3,RAF1,RELA,RPTOR,STAT3
95	Erythropoietin Signaling	19/76 (25%)	57/76 (75%)	8,5	0,3	AKT3,FOS,GRB2,JUN,KRAS,MAP2K1,MAPK3,NRAS,PDPK1,PIK3R1,PIK3R2,PIK3R3,PRKCA,PRKCI,RAF1,RELA,SOCS1,SOCS3,SRC
96	Xenobiotic Metabolism Signaling	39/287 (14%)	248/287 (86%)	8,5	0,1	B28,UGT8
97	RAR Activation	31/194 (16%)	163/194 (84%)	8,5	0,2	AKT3,ARID2,DUSP1,FOS,GTF2H1,IGFBP3,JUN,MAP2K1,MAP2K4,MAPK11,MMP1,NRIP1,PDPK1,PIK3R1,PIK3R2,PIK3R3,PRKACB,PRKCA,PRKCI,PTEN,RDH10,RELA,RXRA,SMAD1,SMAD2,SMAD3,SMAD4,SMAD5,SRC,TGFB3,VEGFA
98	Melanocyte Development and Pigmentation Signaling	21/94 (22%)	73/94 (78%)	8,5	0,2	ATF4,BCL2,CREB1,GRB2,KIT,KITLG,KRAS,MAP2K1,MAPK3,MITF,NRAS,PIK3R1,PIK3R2,PIK3R3,PRKACB,PTPN11,RAF1,RPS6KA5,SRC,TYR,TYRP1
99	Epithelial Adherens Junction Signaling	27/152 (18%)	125/152 (82%)	8,4	0,2	ACTA2,ACVR1,ACVR2A,AKT3,ARPC3,BMPR2,CDC42,CLINT1,CTNNB1,CTNND1,EGFR,FGFR1,KRAS,MET,NOTCH1,NOTCH2,NOTCH4,NRAS,PTEN,PTPRM,RHOA,SRC,TCF7L2,TGFBR1,TGFBR2,TUBB2A,ZYX
100	GM-CSF Signaling	18/70 (26%)	52/70 (74%)	8,3	0,3	AKT3,BCL2L1,CCND1,ETS1,GRB2,KRAS,MAP2K1,MAPK3,NRAS,PIK3R1,PIK3R2,PIK3R3,PPP3CA,PTPN11,RAF1,RUNX1,STAT1,STAT3
101	Thrombopoietin Signaling	17/63 (27%)	46/63 (73%)	8,2	0,3	FOS,GRB2,JUN,KRAS,MAP2K1,MAPK3,MYC,NRAS,PIK3R1,PIK3R2,PIK3R3,PRKCA,PRKCI,PTPN11,RAF1,STAT1,STAT3
102	NRF2-mediated Oxidative Stress Response	30/189 (16%)	159/189 (84%)	8,2	0,2	ACTA2,ATF4,BACH1,DNAJB1,DNAJB4,DNAJC19,FOS,GSK3B,GSTM4,HERPUD1,HMOX1,JUN,KRAS,MAF,MAP2K1,MAP2K4,MAP2K7,MAPK3,MAPK7,NRAS,PIK3R1,PIK3R2,PIK3R3,PRKCA,PRKCI,RAF1,SOD2,SOD3,SQSTM1,TXNRD1
103	IL-17A Signaling in Airway Cells	17/64 (27%)	47/64 (73%)	8,1	0,3	AKT3,CHUK,GSK3B,IKBKE,IL6,JAK1,MAP2K1,MAP2K4,MAPK11,MAPK3,PIK3R1,PIK3R2,PIK3R3,PTEN,RELA,STAT3,TRAF6
104	14-3-3-mediated Signaling	24/127 (19%)	103/127 (81%)	8,1	0,2	AKT3,BAX,CDKN1B,FOS,FOXO1,GRB2,GSK3B,JUN,KRAS,MAP2K1,MAP2K4,MAPK3,NRAS,PDCD6IP,PIK3R1,PIK3R2,PIK3R3,PRKCA,PRKCI,RAF1,SRC,TNF,TUBB2A,VIM
105	Regulation of Cellular Mechanics by Calpain Protease	17/65 (26%)	48/65 (74%)	8,0	0,3	CAPN8,CCNA2,CCND1,CCNE1,CDK4,CDK6,CDKN1B,EGFR,GRB2,ITGA2,ITGA5,KRAS,MAPK3,NRAS,PXN,RB1,SRC
106	Oncostatin M Signaling	14/43 (33%)	29/43 (67%)	8,0	0,3	GRB2,JAK1,KRAS,MAP2K1,MAPK3,MMP1,MMP13,MMP3,NRAS,OSMR,RAF1,STAT1,STAT3,TIMP3
107	GP6 Signaling Pathway	23/119 (19%)	96/119 (81%)	8,0	0,2	AKT3,COL13A1,COL15A1,COL1A1,COL1A2,COL3A1,COL4A1,COL4A2,COL4A5,COL5A1,COL5A2,COL5A3,COL8A1,GSK3B,ITGB3,LAMC1,PDPK1,PIK3R1,PIK3R2,PIK3R3,PRKCA,PRKCI,RHOG
108	Th17 Activation Pathway	20/91 (22%)	71/91 (78%)	8,0	0,2	HIF1A,HSP90B1,IFNG,IL10,IL12RB2,IL1R1,IL6,IL6R,IRAK1,IRAK2,JAK1,MTOR,MYD88,NFATC1,RELA,RUNX1,SOCS3,STAT3,TRA,TRAF6
109	Agranulocyte Adhesion and Diapedesis	30/193 (16%)	163/193 (84%)	8,0	0,2	ACTA2,Ccl6,CCL8,Ccl9,CDH5,CLDN1,CLDN12,CLDN18,CXCL8,CXCR4,ICAM1,IL1F10,IL1R1,IL1RN,IL36A,IL36B,IL36G,IL36RN,IL37,ITGA2,ITGA5,MMP1,MMP13,MMP14,MMP16,MMP3,MMP9,PODXL,TNF,VCAM1
110	Rac Signaling	22/112 (20%)	90/112 (80%)	7,8	0,2	ARFIP2,ARPC3,CD44,CDC42,CFL2,ITGA2,ITGA5,JUN,KRAS,MAP2K1,MAP2K4,MAP2K7,MAPK3,NRAS,PAK5,PIK3R1,PIK3R2,PIK3R3,PRKCI,RAF1,RELA,RHOA
111	Signaling by Rho Family GTPases	34/244 (14%)	210/244 (86%)	7,7	0,1	ACTA2,ARFIP2,ARHGEF3,ARPC3,CDC42,CDH5,CFL2,DIRAS3,FOS,GNA13,ITGA2,ITGA5,JUN,MAP2K1,MAP2K4,MAP2K7,MAP3K12,MAPK3,NEDD4,PAK5,PIK3R1,PIK3R2,PIK3R3,PRKCI,RAF1,RELA,RHOA,RHOB,RHOG,RHOT1,SEPTIN2,SEPTIN3,VIM,WIPF1
112	Docosahexaenoic Acid (DHA) Signaling	13/38 (34%)	25/38 (66%)	7,7	0,3	AKT3,APAF1,APP,BAX,BCL2,BCL2L1,CASP3,FOXO1,GSK3B,PDPK1,PIK3R1,PIK3R2,PIK3R3
113	Xenobiotic Metabolism General Signaling Pathway	25/143 (17%)	118/143 (83%)	7,7	0,2	AKT3,GSTM4,HMOX1,KRAS,MAF,MAP2K1,MAP2K4,MAP2K7,MAP3K12,MAPK11,MAPK3,MAPK7,NR1I2,NRAS,PIK3R1,PIK3R2,PIK3R3,PRKCA,PRKCI,RAF1,RXRA,UGT2B15,UGT2B17,UGT2B28,UGT8
114	FAK Signaling	20/95 (21%)	75/95 (79%)	7,6	0,2	ACTA2,AKT3,CAPN8,EGFR,GRB2,ITGA2,ITGA5,KRAS,MAP2K1,MAPK3,NRAS,PAK5,PDPK1,PIK3R1,PIK3R2,PIK3R3,PTEN,PXN,RAF1,SRC
115	Lymphotoxin β Receptor Signaling	15/53 (28%)	38/53 (72%)	7,6	0,3	AKT3,APAF1,BCL2L1,CASP3,CHUK,IKBKE,LTB,MAPK3,PDPK1,PIK3R1,PIK3R2,PIK3R3,RELA,TRAF6,VCAM1
116	IL-2 Signaling	16/61 (26%)	45/61 (74%)	7,6	0,3	AKT3,FOS,GRB2,IL2RG,JAK1,JUN,KRAS,MAP2K1,MAPK3,NRAS,PIK3R1,PIK3R2,PIK3R3,PTPN11,RAF1,SOCS1
117	Antiproliferative Role of TOB in T Cell Signaling	13/39 (33%)	26/39 (67%)	7,6	0,3	CCNA2,CCNE1,CCNE2,CDKN1B,DPP7,RB1,SMAD2,SMAD3,SMAD4,TGFB3,TGFBR1,TGFBR2,TRA
118	Agrin Interactions at Neuromuscular Junction	18/78 (23%)	60/78 (77%)	7,6	0,2	ACTA2,CDC42,EGFR,ERBB2,ERBB3,ERBB4,ITGA2,ITGA5,ITGB3,JUN,KRAS,LAMC1,MAP2K4,MAPK3,NRAS,PAK5,PXN,SRC
119	Iron homeostasis signaling pathway	24/137 (18%)	113/137 (82%)	7,5	0,2	ATP6AP1L,ATP6V0A1,ATP6V1C1,ATP6V1F,BMPR1B,BMPR2,CIAO2A,EGFR,HIF1A,HJV,HMOX1,IL6,IL6R,JAK1,MAPK3,PDGFA,PDGFB,PDGFRA,SMAD1,SMAD2,SMAD3,SMAD4,SMAD5,STAT3
120	Activation of IRF by Cytosolic Pattern Recognition Receptors	16/63 (25%)	47/63 (75%)	7,4	0,3	CD40,CHUK,FADD,IFNA1/IFNA13,IFNB1,IKBKE,IL10,IL6,JUN,MAP2K4,RELA,RIPK1,STAT1,TBK1,TNF,TRAF6
121	p70S6K Signaling	23/129 (18%)	106/129 (82%)	7,3	0,2	AGTR1,AKT3,EGFR,F2,GRB2,IL2RG,IRS1,JAK1,KRAS,MAP2K1,MAPK3,MTOR,NRAS,PDPK1,PIK3R1,PIK3R2,PIK3R3,PPP2R2A,PPP2R5C,PRKCA,PRKCI,RAF1,SRC
122	NF-κB Activation by Viruses	18/82 (22%)	64/82 (78%)	7,2	0,2	AKT3,CCR5,CHUK,IKBKE,ITGA2,ITGA5,ITGB3,KRAS,MAPK3,NRAS,PIK3R1,PIK3R2,PIK3R3,PRKCA,PRKCI,RAF1,RELA,RIPK1
123	IL-17A Signaling in Fibroblasts	12/35 (34%)	23/35 (66%)	7,2	0,3	CEBPB,CHUK,FOS,GSK3B,IKBKE,IL6,JUN,MAPK11,MAPK3,MMP1,RELA,TRAF6
124	CNTF Signaling	15/57 (26%)	42/57 (74%)	7,2	0,3	GRB2,JAK1,KRAS,MAP2K1,MAPK3,MTOR,NRAS,PIK3R1,PIK3R2,PIK3R3,PTPN11,RAF1,RPS6KA5,STAT1,STAT3
125	mTOR Signaling	30/210 (14%)	180/210 (86%)	7,1	0,1	AKT3,CDC42,DDIT4,DIRAS3,EIF3J,EIF4E,EIF4G2,HIF1A,HMOX1,IRS1,KRAS,MAPK3,MTOR,NRAS,PDPK1,PIK3R1,PIK3R2,PIK3R3,PPP2R2A,PPP2R5C,PRKCA,PRKCI,RHEB,RHOA,RHOB,RHOG,RHOT1,RPS6KA5,RPTOR,VEGFA
126	Adipogenesis pathway	23/134 (17%)	111/134 (83%)	7,0	0,2	BMPR2,CEBPB,CTNNB1,EGR2,FBXW7,FGF2,FGFR1,FGFR3,FOXO1,GTF2H1,HDAC4,HIF1A,KLF5,LPL,PPARG,RB1,SMAD1,SMAD3,SMAD5,SMO,SOX9,TNF,TP53
127	UVC-Induced MAPK Signaling	14/51 (27%)	37/51 (73%)	7,0	0,3	EGFR,FOS,JUN,KRAS,MAP2K1,MAP2K4,MAPK11,MAPK3,NRAS,PRKCA,PRKCI,RAF1,SRC,TP53
128	BMP signaling pathway	18/85 (21%)	67/85 (79%)	7,0	0,2	BMPR1B,BMPR2,CREB1,GRB2,JUN,KRAS,MAP2K1,MAP2K4,MAPK11,MAPK3,NRAS,PRKACB,RAF1,RELA,RUNX2,SMAD1,SMAD4,SMAD5
129	Tumoricidal Function of Hepatic Natural Killer Cells	10/24 (42%)	14/24 (58%)	7,0	0,4	APAF1,BAX,CASP3,CASP6,CASP7,DFFA,FADD,FAS,FASLG,ICAM1
130	Tight Junction Signaling	26/168 (15%)	142/168 (85%)	6,9	0,2	ACTA2,AKT3,CDC42,CDK4,CLDN1,CLDN12,CLDN18,CTNNB1,F11R,FOS,JUN,NAPG,PPP2R2A,PPP2R5C,PRKACB,PRKCI,PTEN,RELA,RHOA,TGFB3,TGFBR1,TGFBR2,TJP1,TNF,VAMP3,VTI1B
131	Necroptosis Signaling Pathway	25/157 (16%)	132/157 (84%)	6,9	0,2	CAPN8,CFLAR,CHUK,FADD,FAS,FASLG,IFNA1/IFNA13,IFNB1,JAK1,MDM2,PELI1,PPIF,PPP3CA,RB1,RBL2,RIPK1,SLC25A13,STAT1,TAB2,TLR3,TLR4,TNF,TNFRSF10A,TOMM34,TP53
132	T Cell Receptor Signaling	20/105 (19%)	85/105 (81%)	6,9	0,2	CHUK,CTLA4,FOS,GRB2,IKBKE,JUN,KRAS,MAP2K1,MAP2K4,MAPK3,NFATC1,NRAS,PIK3R1,PIK3R2,PIK3R3,PPP3CA,RAF1,RASA1,RELA,TRA
133	GADD45 Signaling	9/19 (47%)	10/19 (53%)	6,9	0,5	BRCA1,CCND1,CCND2,CCND3,CCNE1,CCNE2,CDK4,CDKN1A,TP53
134	Protein Kinase A Signaling	44/399 (11%)	355/399 (89%)	6,8	0,1	3B,MAP2K1,MAPK3,NFATC1,PDE3A,PHKB,PPP1R7,PPP3CA,PRKACB,PRKCA,PRKCI,PTEN,PTGS2,PTPN11,PTPRJ,PTPRM,PXN,RAF1,RELA,RHOA,SMAD3,SMAD4,SMO,TCF7L2,TGFB3,TGFBR1,TGFBR2
135	Ceramide Signaling	18/88 (20%)	70/88 (80%)	6,7	0,2	AKT3,BCL2,FOS,JUN,KRAS,MAP2K1,MAP2K4,MAPK3,NRAS,PIK3R1,PIK3R2,PIK3R3,PPP2R2A,PPP2R5C,RAF1,RELA,S1PR1,TNF
136	UVA-Induced MAPK Signaling	19/98 (19%)	79/98 (81%)	6,7	0,2	BCL2L1,CASP3,EGFR,FOS,JUN,KRAS,MAP2K4,MAPK11,MAPK3,MTOR,NRAS,PARP8,PIK3R1,PIK3R2,PIK3R3,PRKCA,RPS6KA5,STAT1,TP53
137	PFKFB4 Signaling Pathway	13/46 (28%)	33/46 (72%)	6,7	0,3	ATF4,CREB1,FGF2,HK2,MAP2K1,MAP2K4,MAP2K7,MAPK3,NCOA3,PRKACB,RPS6KA5,TGFB3,TP53
138	Role of Cytokines in Mediating Communication between Immune Cells	14/54 (26%)	40/54 (74%)	6,7	0,3	CXCL8,IFNA1/IFNA13,IFNB1,IFNG,IL10,IL1F10,IL1RN,IL36A,IL36B,IL36G,IL36RN,IL37,IL6,TNF
139	Apelin Liver Signaling Pathway	10/26 (38%)	16/26 (62%)	6,6	0,4	APLN,COL1A1,COL1A2,COL3A1,COL5A3,FAS,GSK3B,IRS1,MAP2K4,TNF
140	ERK5 Signaling	16/72 (22%)	56/72 (78%)	6,6	0,2	ATF4,CREB1,EGFR,FOS,FOXO3,GNA13,KRAS,LIF,MAPK7,MEF2C,MEF2D,MYC,NRAS,PTPN11,RPS6KA5,SRC
141	IL-9 Signaling	11/33 (33%)	22/33 (67%)	6,5	0,3	IL2RG,IRS1,JAK1,PIK3R1,PIK3R2,PIK3R3,RELA,SOCS3,STAT1,STAT3,TNF
142	SAPK/JNK Signaling	19/102 (19%)	83/102 (81%)	6,4	0,2	CDC42,CRKL,FADD,GNA13,GRB2,IRS1,JUN,KRAS,MAP2K4,MAP2K7,MAP3K12,NFATC1,NRAS,PIK3R1,PIK3R2,PIK3R3,RIPK1,TP53,TRA
143	CXCR4 Signaling	25/167 (15%)	142/167 (85%)	6,4	0,2	AKT3,CDC42,CXCR4,DIRAS3,FOS,GNA13,JUN,KRAS,MAP2K1,MAP2K4,MAPK3,NRAS,PAK5,PIK3R1,PIK3R2,PIK3R3,PRKCA,PRKCI,PXN,RAF1,RHOA,RHOB,RHOG,RHOT1,SRC
144	Clathrin-mediated Endocytosis Signaling	27/193 (14%)	166/193 (86%)	6,3	0,1	ACTA2,ARPC3,CDC42,F2,FGF16,FGF2,FGF7,GAK,GRB2,HGS,IGF1,ITGA5,ITGB3,MDM2,MET,MYO1E,PDGFA,PDGFB,PICALM,PIK3R1,PIK3R2,PIK3R3,PPP3CA,RAB5C,SH3BP4,SRC,VEGFA
145	Gαq Signaling	24/158 (15%)	134/158 (85%)	6,3	0,2	AGTR1,AKT3,CDC42,CHUK,DIRAS3,GSK3B,GYS1,HMOX1,IKBKE,MAP2K1,MAPK3,NFATC1,PIK3R1,PIK3R2,PIK3R3,PPP3CA,PRKCA,PRKCI,RAF1,RELA,RHOA,RHOB,RHOG,RHOT1
146	Actin Cytoskeleton Signaling	29/218 (13%)	189/218 (87%)	6,3	0,1	ACTA2,ARPC3,CD14,CDC42,CFL2,CRKL,F2,FGF16,FGF2,FGF7,GIT1,GNA13,GRB2,ITGA2,ITGA5,KRAS,LBP,MAP2K1,MAPK3,NRAS,PAK5,PDGFA,PDGFB,PIK3R1,PIK3R2,PIK3R3,PXN,RAF1,RHOA
147	GDNF Family Ligand-Receptor Interactions	16/76 (21%)	60/76 (79%)	6,2	0,2	CDC42,CREB1,FOS,GRB2,IRS1,JUN,KRAS,MAP2K1,MAP2K4,MAPK3,NRAS,PIK3R1,PIK3R2,PIK3R3,RAF1,RASA1
148	PD-1, PD-L1 cancer immunotherapy pathway	19/106 (18%)	87/106 (82%)	6,2	0,2	AKT3,BCL2L1,CDKN1B,GSK3B,IFNG,IL2RG,JAK1,LATS2,MR1,PDCD4,PIK3R1,PIK3R2,PIK3R3,PTEN,PTPN11,SMAD3,TGFB3,TNF,TRA
149	GNRH Signaling	25/173 (14%)	148/173 (86%)	6,1	0,1	ATF4,CACNA2D1,CDC42,CREB1,EGFR,FOS,GRB2,JUN,KRAS,MAP2K1,MAP2K4,MAP2K7,MAP3K12,MAPK11,MAPK3,MAPK7,NRAS,PAK5,PRKACB,PRKCA,PRKCI,PXN,RAF1,RELA,SRC
150	ATM Signaling	18/97 (19%)	79/97 (81%)	6,1	0,2	ABL1,ATF4,BRCA1,CDC25A,CDKN1A,CHEK1,CREB1,FANCD2,JUN,MAP2K4,MAPK11,MDM2,PPM1D,PPP2R2A,PPP2R5C,SMC1A,TP53,ZEB1
151	Reelin Signaling in Neurons	21/129 (16%)	108/129 (84%)	6,1	0,2	AKT3,APP,ARHGEF3,ARPC3,CDC42,CRKL,GSK3B,ITGA2,ITGA5,ITGB3,MAP2K1,MAP2K4,MAP2K7,MAPK3,MTOR,PAFAH1B2,PIK3R1,PIK3R2,PIK3R3,RHOA,SRC
152	Role of JAK1 and JAK3 in γc Cytokine Signaling	15/69 (22%)	54/69 (78%)	6,1	0,2	GRB2,IL2RG,IRS1,JAK1,KRAS,MAPK3,NRAS,PIK3R1,PIK3R2,PIK3R3,PTPN11,SOCS1,SOCS3,STAT1,STAT3
153	CD28 Signaling in T Helper Cells	20/120 (17%)	100/120 (83%)	6,0	0,2	AKT3,ARPC3,CDC42,CHUK,CTLA4,FOS,GRB2,IKBKE,JUN,MAP2K1,MAP2K4,NFATC1,PDPK1,PIK3R1,PIK3R2,PIK3R3,PPP3CA,PTPN11,RELA,TRA
154	Sirtuin Signaling Pathway	34/291 (12%)	257/291 (88%)	6,0	0,1	APP,ATG2B,ATG3,ATG9A,BAX,BCL2L11,CXCL8,E2F1,FOXO1,FOXO3,GSK3B,H3-3A/H3-3B,HIF1A,JUN,MAPK3,MAPK7,MTOR,MYC,NDUFA4,NEDD4,NOS2,PPARG,PPIF,RELA,RPTOR,SMARCA5,SOD2,SOD3,SP1,STAT3,TNF,TOMM34,TP53,UCP2
155	Cell Cycle Regulation by BTG Family Proteins	11/37 (30%)	26/37 (70%)	6,0	0,3	BTG2,CCND1,CCNE1,CCNE2,CDK4,E2F1,E2F2,E2F3,PPP2R2A,PPP2R5C,RB1
156	Role of JAK1, JAK2 and TYK2 in Interferon Signaling	9/24 (38%)	15/24 (63%)	5,9	0,4	IFNA1/IFNA13,IFNB1,IFNG,JAK1,RAF1,RELA,SOCS1,STAT1,STAT3
157	IL-1 Signaling	17/91 (19%)	74/91 (81%)	5,8	0,2	CHUK,FOS,GNA13,IKBKE,IL1R1,IL1RAP,IRAK1,IRAK2,JUN,MAP2K4,MAP2K7,MAPK11,MYD88,PRKACB,RELA,TAB2,TRAF6
158	Regulation of eIF4 and p70S6K Signaling	23/157 (15%)	134/157 (85%)	5,8	0,1	AGO4,AKT3,EIF3J,EIF4E,EIF4EBP2,EIF4G2,GRB2,IRS1,ITGA2,ITGA5,KRAS,MAP2K1,MAPK11,MAPK3,MTOR,NRAS,PDPK1,PIK3R1,PIK3R2,PIK3R3,PPP2R2A,PPP2R5C,RAF1
159	Phospholipase C Signaling	31/257 (12%)	226/257 (88%)	5,8	0,1	AHNAK,ARHGEF3,ATF4,CDC42,CREB1,DIRAS3,GNA13,GRB2,HDAC4,HMOX1,ITGA2,ITGA5,KRAS,MAP2K1,MAPK3,MARCKS,MEF2C,MEF2D,NFATC1,NRAS,PPP3CA,PRKCA,PRKCI,RAF1,RELA,RHOA,RHOB,RHOG,RHOT1,SRC,TRA
160	T Helper Cell Differentiation	15/73 (21%)	58/73 (79%)	5,7	0,2	CD40,CD40LG,IFNG,IL10,IL12RB2,IL2RG,IL6,IL6R,STAT1,STAT3,TBX21,TGFBR1,TGFBR2,TNF,TRA
161	Synaptogenesis Signaling Pathway	35/312 (11%)	277/312 (89%)	5,7	0,1	AKT3,ARPC3,ATF4,BDNF,CACNA2D1,CADM1,CDC42,CDH5,CREB1,CRKL,CTNNB1,CTNND1,EIF4EBP2,GRB2,GRIA2,GSK3B,KRAS,MAPK3,MARCKS,MTOR,NAPG,NLGN1,NRAS,PIK3R1,PIK3R2,PIK3R3,PRKACB,RAB5C,RAF1,RHOA,SRC,SYT1,THBS1,VAMP3,VTI1B
162	IL-17A Signaling in Gastric Cells	9/25 (36%)	16/25 (64%)	5,7	0,4	CXCL8,EGFR,FOS,JUN,MAP2K4,MAPK11,MAPK3,RELA,TNF
163	G-Protein Coupled Receptor Signaling	32/272 (12%)	240/272 (88%)	5,7	0,1	ADORA2B,AGTR1,AKT3,ATF4,CHUK,CREB1,DRD3,DUSP1,ENPP6,GRB2,IKBKE,KRAS,LAMTOR3,MAP2K1,MAPK3,NPR3,NRAS,PDE3A,PDPK1,PIK3R1,PIK3R2,PIK3R3,PRKACB,PRKCA,PTGIR,RAF1,RASA1,RELA,S1PR1,SRC,STAT3,TBXA2R
164	Phagosome Formation	20/125 (16%)	105/125 (84%)	5,7	0,2	CDC42,DIRAS3,ITGA2,ITGA5,MARCKS,PIK3R1,PIK3R2,PIK3R3,PRKCA,PRKCI,RHOA,RHOB,RHOG,RHOT1,TLR1,TLR10,TLR3,TLR4,TLR7,TLR9
165	Fcγ Receptor-mediated Phagocytosis in Macrophages and Monocytes	17/94 (18%)	77/94 (82%)	5,7	0,2	ACTA2,AKT3,ARPC3,CDC42,HMOX1,INPP5D,MAPK3,MYO5A,PIK3R1,PIK3R2,PIK3R3,PRKCA,PRKCI,PTEN,PXN,SRC,VAMP3
166	HIPPO signaling	16/85 (19%)	69/85 (81%)	5,6	0,2	AJUBA,CD44,CSNK1D,ITCH,LATS2,NF2,PPP1R7,PPP2R2A,PPP2R5C,SCRIB,SMAD1,SMAD2,SMAD3,SMAD4,SMAD5,STK4
167	IL-4 Signaling	16/85 (19%)	69/85 (81%)	5,6	0,2	AKT3,GRB2,HMGA1,IL13RA1,IL2RG,INPP5D,IRS1,JAK1,KRAS,MTOR,NFATC1,NRAS,PIK3R1,PIK3R2,PIK3R3,SOCS1
168	Fc Epsilon RI Signaling	19/117 (16%)	98/117 (84%)	5,6	0,2	AKT3,GRB2,INPP5D,KRAS,MAP2K1,MAP2K4,MAP2K7,MAPK11,MAPK3,NRAS,PDPK1,PIK3R1,PIK3R2,PIK3R3,PRKCA,PRKCI,PTPN11,RAF1,TNF
169	Sphingosine-1-phosphate Signaling	19/117 (16%)	98/117 (84%)	5,6	0,2	AKT3,CASP3,CASP6,CASP7,CDC42,DIRAS3,GNA13,MAPK3,PDGFA,PDGFB,PDGFRA,PIK3R1,PIK3R2,PIK3R3,RHOA,RHOB,RHOG,RHOT1,S1PR1
170	ErbB4 Signaling	14/67 (21%)	53/67 (79%)	5,5	0,2	ADAM17,ERBB4,GRB2,KRAS,MAP2K1,MAPK3,NRAS,PDPK1,PIK3R1,PIK3R2,PIK3R3,PRKCA,PRKCI,RAF1
171	Cytotoxic T Lymphocyte-mediated Apoptosis of Target Cells	10/34 (29%)	24/34 (71%)	5,4	0,3	APAF1,BCL2,CASP3,CASP6,CASP7,DFFA,FADD,FAS,FASLG,TRA
172	Nur77 Signaling in T Lymphocytes	14/68 (21%)	54/68 (79%)	5,4	0,2	APAF1,BCL2,CASP3,FASLG,MAP2K1,MAPK3,MAPK7,MEF2D,NFATC1,PPP3CA,PRKCA,PRKCI,RXRA,TRA
173	MIF Regulation of Innate Immunity	11/42 (26%)	31/42 (74%)	5,4	0,3	CD14,FOS,JUN,MAP2K4,MAPK3,MIF,NOS2,PTGS2,RELA,TLR4,TP53
174	Corticotropin Releasing Hormone Signaling	21/145 (14%)	124/145 (86%)	5,3	0,1	ATF4,BDNF,CACNA2D1,CREB1,FASLG,FOS,JUN,MAP2K1,MAPK11,MAPK3,MEF2C,MEF2D,NOS2,NPR3,PRKACB,PRKCA,PRKCI,PTGS2,RAF1,SMO,VEGFA
175	iCOS-iCOSL Signaling in T Helper Cells	18/111 (16%)	93/111 (84%)	5,3	0,2	AKT3,CD40,CD40LG,CHUK,GRB2,IKBKE,IL2RG,INPP5D,NFATC1,PDPK1,PIK3R1,PIK3R2,PIK3R3,PLEKHA4,PPP3CA,PTEN,RELA,TRA
176	Role of BRCA1 in DNA Damage Response	15/80 (19%)	65/80 (81%)	5,3	0,2	ARID2,BRCA1,CDKN1A,CHEK1,E2F1,E2F2,E2F3,FANCD2,IFNG,MSH2,PLK1,RB1,RBL2,STAT1,TP53
177	Interferon Signaling	10/36 (28%)	26/36 (72%)	5,2	0,3	BAK1,BAX,BCL2,IFNA1/IFNA13,IFNB1,IFNG,JAK1,RELA,SOCS1,STAT1
178	IL-23 Signaling Pathway	11/44 (25%)	33/44 (75%)	5,2	0,3	AKT3,HIF1A,IL12RB2,PIK3R1,PIK3R2,PIK3R3,RELA,RUNX1,SOCS3,STAT3,TNF
179	CD27 Signaling in Lymphocytes	12/53 (23%)	41/53 (77%)	5,2	0,2	APAF1,BCL2L1,CASP3,CHUK,FOS,IKBKE,JUN,MAP2K1,MAP2K4,MAP2K7,MAP3K12,RELA
180	Gap Junction Signaling	25/198 (13%)	173/198 (87%)	5,1	0,1	ACTA2,AKT3,CSNK1D,CTNNB1,EGFR,GRB2,GRIA2,KRAS,MAP2K1,MAPK3,MAPK7,NPR3,NRAS,PIK3R1,PIK3R2,PIK3R3,PPP3CA,PRKACB,PRKCA,PRKCI,RAF1,SP1,SRC,TJP1,TUBB2A
181	LPS/IL-1 Mediated Inhibition of RXR Function	27/224 (12%)	197/224 (88%)	5,1	0,1	ABCB1,CD14,GSTM4,IL1F10,IL1R1,IL1RAP,IL1RAPL2,IL1RL2,IL1RN,IL36A,IL36B,IL36G,IL36RN,IL37,IRAK1,JUN,LBP,MAP2K4,MAP2K7,MYD88,NR1I2,RXRA,SMOX,SOD3,TLR4,TNF,TRAF6
182	TNFR2 Signaling	9/30 (30%)	21/30 (70%)	5,0	0,3	CHUK,FOS,IKBKE,JUN,MAP2K4,RELA,TBK1,TNF,TNFAIP3
183	Angiopoietin Signaling	14/75 (19%)	61/75 (81%)	4,9	0,2	AKT3,CHUK,FOXO1,GRB2,IKBKE,KRAS,NRAS,PAK5,PIK3R1,PIK3R2,PIK3R3,PTPN11,RASA1,RELA
184	Semaphorin Neuronal Repulsive Signaling Pathway	19/130 (15%)	111/130 (85%)	4,9	0,1	AKT3,CD44,CFL2,ERBB2,GSK3B,ITGA2,ITGA5,MAP2K1,MAP2K4,MAP2K7,PAK5,PIK3R1,PIK3R2,PIK3R3,PLXND1,PRKACB,RHOA,TP53,VCAN
185	IL-22 Signaling	8/24 (33%)	16/24 (67%)	4,9	0,3	AKT3,JAK1,MAP2K4,MAPK11,MAPK3,SOCS3,STAT1,STAT3
186	April Mediated Signaling	10/39 (26%)	29/39 (74%)	4,9	0,3	CHUK,FOS,IKBKE,JUN,MAP2K4,MAP2K7,MAPK11,NFATC1,RELA,TRAF6
187	Paxillin Signaling	17/108 (16%)	91/108 (84%)	4,8	0,2	ACTA2,ARFIP2,CDC42,GRB2,ITGA2,ITGA5,ITGB3,KRAS,MAP2K4,MAPK11,NRAS,PAK5,PIK3R1,PIK3R2,PIK3R3,PXN,SRC
188	Role of NFAT in Regulation of the Immune Response	23/181 (13%)	158/181 (87%)	4,8	0,1	AKT3,CHUK,CSNK1D,FOS,GNA13,GRB2,GSK3B,IKBKE,JUN,KRAS,MAP2K1,MAPK3,MEF2C,MEF2D,NFATC1,NRAS,PIK3R1,PIK3R2,PIK3R3,PPP3CA,RAF1,RELA,TRA
189	4-1BB Signaling in T Lymphocytes	9/32 (28%)	23/32 (72%)	4,8	0,3	CHUK,IKBKE,JUN,MAP2K1,MAP2K4,MAPK11,MAPK3,RELA,TNFSF9
190	Cell Cycle: G2/M DNA Damage Checkpoint Regulation	11/49 (22%)	38/49 (78%)	4,7	0,2	ABL1,BRCA1,CDKN1A,CDKN2A,CHEK1,MDM2,PKMYT1,PLK1,PPM1D,TP53,WEE1
191	Inhibition of ARE-Mediated mRNA Degradation Pathway	18/122 (15%)	104/122 (85%)	4,7	0,1	AGO4,AKT3,CD40LG,CNOT6,CNOT8,DCP2,FASLG,LTB,MAPK11,MAPK3,MAPK7,PPP2R2A,PPP2R5C,PRKACB,TIA1,TNF,TNFSF9,ZFP36L1
192	B Cell Activating Factor Signaling	10/41 (24%)	31/41 (76%)	4,7	0,2	CHUK,FOS,IKBKE,JUN,MAP2K4,MAP2K7,MAPK11,NFATC1,RELA,TRAF6
193	Chemokine Signaling	14/80 (18%)	66/80 (83%)	4,6	0,2	CCR3,CCR5,CXCR4,FOS,JUN,KRAS,MAP2K1,MAPK11,MAPK3,NRAS,PRKCA,RAF1,RHOA,SRC
194	AMPK Signaling	25/214 (12%)	189/214 (88%)	4,6	0,1	AKT3,ARID2,ATF4,CCNA2,CCND1,CDKN1A,CREB1,ELAVL1,FOXO1,FOXO3,GYS1,IRS1,MAPK11,MTOR,PIK3R1,PIK3R2,PIK3R3,PPM1D,PPP2R2A,PPP2R5C,PRKACB,RAB27A,RAB6A,RAB9B,RPTOR
195	Actin Nucleation by ARP-WASP Complex	13/72 (18%)	59/72 (82%)	4,4	0,2	ARPC3,CDC42,DIRAS3,GRB2,ITGA2,ITGA5,KRAS,NRAS,RHOA,RHOB,RHOG,RHOT1,WIPF1
196	TWEAK Signaling	9/35 (26%)	26/35 (74%)	4,4	0,3	APAF1,CASP3,CASP6,CASP7,CHUK,FADD,IKBKE,RELA,RIPK1
197	VEGF Family Ligand-Receptor Interactions	14/84 (17%)	70/84 (83%)	4,4	0,2	AKT3,FOS,GRB2,KRAS,MAP2K1,MAPK3,NRAS,PIK3R1,PIK3R2,PIK3R3,PRKCA,PRKCI,RAF1,VEGFA
198	Hypoxia Signaling in the Cardiovascular System	13/74 (18%)	61/74 (82%)	4,3	0,2	ATF4,CREB1,CSNK1D,HIF1A,HSP90B1,JUN,MDM2,PTEN,TP53,UBE2I,UBE2J1,UBE2S,VEGFA
199	Sperm Motility	25/223 (11%)	198/223 (89%)	4,3	0,1	ABL1,CSF1R,DDR1,DSTYK,EGFR,ERBB2,ERBB3,ERBB4,FGFR1,FGFR3,IGF1R,JAK1,KIT,MAP2K1,MAP2K4,MAP2K7,MET,NTRK3,PDGFRA,PRKACB,PRKCA,PRKCI,SLC12A2,SLC16A10,SRC
200	Role of p14/p19ARF in Tumor Suppression	8/29 (28%)	21/29 (72%)	4,2	0,3	CDKN2A,E2F1,MDM2,PIK3R1,PIK3R2,PIK3R3,RB1,TP53
201	Role of Oct4 in Mammalian Embryonic Stem Cell Pluripotency	10/46 (22%)	36/46 (78%)	4,2	0,2	BMI1,BRCA1,CASP6,CCNF,CDX2,IGF2BP1,JARID2,PHB,RB1,TP53
202	Unfolded protein response	11/56 (20%)	45/56 (80%)	4,2	0,2	ATF4,ATF6,BCL2,CEBPB,CEBPG,HSP90B1,HSPA14,HSPA1A/HSPA1B,INSIG1,MAP2K7,PPARG
203	Role of CHK Proteins in Cell Cycle Checkpoint Control	11/57 (19%)	46/57 (81%)	4,1	0,2	BRCA1,CDC25A,CDKN1A,CHEK1,E2F1,E2F2,E2F3,PLK1,PPP2R2A,PPP2R5C,TP53
204	Inhibition of Matrix Metalloproteases	9/39 (23%)	30/39 (77%)	4,0	0,2	ADAM17,MMP1,MMP13,MMP14,MMP16,MMP3,MMP9,RECK,TIMP3
205	Remodeling of Epithelial Adherens Junctions	12/68 (18%)	56/68 (82%)	4,0	0,2	ACTA2,ARPC3,CTNNB1,CTNND1,HGS,MAPRE1,MAPRE2,MET,RAB5C,SRC,TUBB2A,ZYX
206	Xenobiotic Metabolism CAR Signaling Pathway	22/189 (12%)	167/189 (88%)	4,0	0,1	ABCB1,CYP1A1,EGFR,GSTM4,HSP90B1,MAP2K1,MAP2K4,MAP2K7,MAPK3,NOS2,NRIP1,PPP2R2A,PPP2R5C,PRKCA,PRKCI,RXRA,SOD3,SRC,UGT2B15,UGT2B17,UGT2B28,UGT8
207	Opioid Signaling Pathway	26/247 (11%)	221/247 (89%)	4,0	0,1	AKT3,ATF4,CACNA2D1,CDC42,CDKN1B,CREB1,CTNNB1,FOS,GSK3B,KRAS,MAP2K1,MAP2K4,MAP2K7,MAPK3,MAPK7,MYC,NRAS,PPP3CA,PRKACB,PRKCA,PRKCI,RAF1,RPS6KA5,SIGMAR1,SRC,SRF
208	3-phosphoinositide Biosynthesis	20/166 (12%)	146/166 (88%)	3,9	0,1	ACP1,ATP1A2,CDC25A,CDIPT,DUSP1,DUSP12,DUSP2,DUSP23,PIK3R1,PIK3R2,PIK3R3,PPP1R7,PPP3CA,PPP5C,PTEN,PTPN11,PTPRJ,PTPRM,RASA1,SOCS3
209	TR/RXR Activation	13/84 (15%)	71/84 (85%)	3,7	0,2	AKT3,DIO3,HIF1A,MDM2,MTOR,NCOA3,PIK3R1,PIK3R2,PIK3R3,RXRA,SLC16A3,THRB,UCP2
210	Regulation Of The Epithelial Mesenchymal Transition In Development Pathway	13/84 (15%)	71/84 (85%)	3,7	0,2	ADAM17,BRCA1,CTNNB1,GSK3B,HIF1A,JAG1,NOTCH1,RELA,SMO,TCF7L2,WNT1,WNT3A,WNT7B
211	CDK5 Signaling	15/108 (14%)	93/108 (86%)	3,7	0,1	ABL1,BDNF,ITGA2,KRAS,LAMC1,MAP2K1,MAPK11,MAPK3,MAPK7,NRAS,PPP1R7,PPP2R2A,PPP2R5C,PRKACB,RAF1
212	Role of JAK2 in Hormone-like Cytokine Signaling	8/34 (24%)	26/34 (76%)	3,7	0,2	IRS1,JAK1,PTPN11,SOCS1,SOCS3,SOCS5,STAT1,STAT3
213	Insulin Secretion Signaling Pathway	25/243 (10%)	218/243 (90%)	3,7	0,1	AGO4,ATF4,CREB1,CSHL1,EIF4E,EIF4EBP2,EIF4G2,JAK1,MAPK11,MAPK3,MTOR,NAPG,PIK3R1,PIK3R2,PIK3R3,PRKACB,PRKCA,PRKCI,RPS6KA5,SPCS3,SRC,STAT1,STAT3,VAMP3,VTI1B
214	G Beta Gamma Signaling	16/122 (13%)	106/122 (87%)	3,6	0,1	AKT3,CACNA2D1,CAV2,CDC42,EGFR,GNA13,GRB2,KRAS,MAPK3,NRAS,PDPK1,PRKACB,PRKCA,PRKCI,RAF1,SRC
215	Role of RIG1-like Receptors in Antiviral Innate Immunity	9/44 (20%)	35/44 (80%)	3,6	0,2	CHUK,FADD,IFNA1/IFNA13,IFNB1,IKBKE,RELA,RIPK1,TBK1,TRAF6
216	PXR/RXR Activation	11/65 (17%)	54/65 (83%)	3,6	0,2	ABCB1,AKT3,FOXO1,FOXO3,IL6,NR1I2,PRKACB,RELA,RXRA,SCD,TNF
217	Relaxin Signaling	18/150 (12%)	132/150 (88%)	3,6	0,1	AKT3,CREB1,ENPP6,FOS,GNA13,JUN,MAP2K1,MAPK3,MMP9,NOS2,NPR3,PDE3A,PIK3R1,PIK3R2,PIK3R3,PRKACB,RELA,VEGFA
218	Mitotic Roles of Polo-Like Kinase	11/66 (17%)	55/66 (83%)	3,5	0,2	ANAPC1,CDC25A,HSP90B1,KIF23,PKMYT1,PLK1,PPP2R2A,PPP2R5C,PRC1,SMC1A,WEE1
219	Antiproliferative Role of Somatostatin Receptor 2	12/77 (16%)	65/77 (84%)	3,5	0,2	CDKN1A,CDKN1B,KRAS,MAP2K1,MAPK3,NPR3,NRAS,PIK3R1,PIK3R2,PIK3R3,PTPN11,SRC
220	Pyridoxal 5'-phosphate Salvage Pathway	11/66 (17%)	55/66 (83%)	3,5	0,2	ACVR2A,CDK4,CDK6,CSNK1D,IRAK1,MAP2K1,MAP2K4,MAPK3,MAPK7,PAK5,PLK1
221	FXR/RXR Activation	16/126 (13%)	110/126 (87%)	3,5	0,1	AKT3,FOXO1,G6PC3,IL1F10,IL1RN,IL36A,IL36B,IL36G,IL36RN,IL37,LPL,MAP2K4,NR1I2,PPARG,RXRA,TNF
222	RhoGDI Signaling	20/180 (11%)	160/180 (89%)	3,5	0,1	ACTA2,ARHGDIA,ARHGEF3,ARPC3,CD44,CDC42,CDH5,CFL2,DIRAS3,ESR1,GNA13,ITGA2,ITGA5,PAK5,PRKCA,RHOA,RHOB,RHOG,RHOT1,SRC
223	EIF2 Signaling	23/224 (10%)	201/224 (90%)	3,4	0,1	ACTA2,AGO4,AKT3,ATF4,BCL2,CCND1,EIF3J,EIF4E,EIF4G2,GRB2,GSK3B,IGF1R,KRAS,MAP2K1,MAPK3,MYC,NRAS,PDPK1,PIK3R1,PIK3R2,PIK3R3,RAF1,VEGFA
224	PKCθ Signaling in T Lymphocytes	18/155 (12%)	137/155 (88%)	3,4	0,1	CACNA2D1,CHUK,FOS,GRB2,IKBKE,JUN,KRAS,MAP2K4,MAP3K12,MAPK3,NFATC1,NRAS,PIK3R1,PIK3R2,PIK3R3,PPP3CA,RELA,TRA
225	fMLP Signaling in Neutrophils	15/116 (13%)	101/116 (87%)	3,4	0,1	ARPC3,CDC42,KRAS,MAP2K1,MAPK3,NFATC1,NRAS,PIK3R1,PIK3R2,PIK3R3,PPP3CA,PRKCA,PRKCI,RAF1,RELA
226	MSP-RON Signaling Pathway	10/58 (17%)	48/58 (83%)	3,4	0,2	ACTA2,CSF1,IFNG,KLK10,NOS2,PIK3R1,PIK3R2,PIK3R3,TLR4,TNF
227	Superoxide Radicals Degradation	4/8 (50%)	4/8 (50%)	3,4	0,5	GPX7,SOD2,SOD3,TYRP1
228	Superpathway of Inositol Phosphate Compounds	21/199 (11%)	178/199 (89%)	3,3	0,1	ACP1,ATP1A2,CDC25A,CDIPT,DUSP1,DUSP12,DUSP2,DUSP23,INPP5D,PIK3R1,PIK3R2,PIK3R3,PPP1R7,PPP3CA,PPP5C,PTEN,PTPN11,PTPRJ,PTPRM,RASA1,SOCS3
229	Regulation of Actin-based Motility by Rho	13/94 (14%)	81/94 (86%)	3,3	0,1	ACTA2,ARHGDIA,ARPC3,CDC42,DIRAS3,ITGA2,ITGA5,PAK5,RHOA,RHOB,RHOG,RHOT1,WIPF1
230	Semaphorin Signaling in Neurons	10/60 (17%)	50/60 (83%)	3,2	0,2	CDC42,CFL2,DIRAS3,MAPK3,MET,PAK5,RHOA,RHOB,RHOG,RHOT1
231	Retinoic acid Mediated Apoptosis Signaling	10/60 (17%)	50/60 (83%)	3,2	0,2	APAF1,CASP3,CFLAR,FADD,IFNA1/IFNA13,IFNB1,IFNG,PARP8,RXRA,TNFRSF10A
232	Virus Entry via Endocytic Pathways	14/107 (13%)	93/107 (87%)	3,2	0,1	ABL1,ACTA2,CDC42,ITGA2,ITGA5,ITGB3,KRAS,NRAS,PIK3R1,PIK3R2,PIK3R3,PRKCA,PRKCI,SRC
233	Apelin Cardiac Fibroblast Signaling Pathway	6/23 (26%)	17/23 (74%)	3,1	0,3	AGTR1,AKT3,APLN,CCN2,IL6,TGFB3
234	CCR3 Signaling in Eosinophils	15/124 (12%)	109/124 (88%)	3,1	0,1	CCR3,CFL2,KRAS,MAP2K1,MAPK11,MAPK3,NRAS,PAK5,PIK3R1,PIK3R2,PIK3R3,PRKCA,PRKCI,RAF1,RHOA
235	D-myo-inositol (1,4,5,6)-Tetrakisphosphate Biosynthesis	16/142 (11%)	126/142 (89%)	2,9	0,1	ACP1,ATP1A2,CDC25A,DUSP1,DUSP12,DUSP2,DUSP23,PPP1R7,PPP3CA,PPP5C,PTEN,PTPN11,PTPRJ,PTPRM,RASA1,SOCS3
236	D-myo-inositol (3,4,5,6)-tetrakisphosphate Biosynthesis	16/142 (11%)	126/142 (89%)	2,9	0,1	ACP1,ATP1A2,CDC25A,DUSP1,DUSP12,DUSP2,DUSP23,PPP1R7,PPP3CA,PPP5C,PTEN,PTPN11,PTPRJ,PTPRM,RASA1,SOCS3
237	3-phosphoinositide Degradation	17/156 (11%)	139/156 (89%)	2,9	0,1	ACP1,ATP1A2,CDC25A,DUSP1,DUSP12,DUSP2,DUSP23,INPP5D,PPP1R7,PPP3CA,PPP5C,PTEN,PTPN11,PTPRJ,PTPRM,RASA1,SOCS3
238	DNA Methylation and Transcriptional Repression Signaling	7/35 (20%)	28/35 (80%)	2,9	0,2	ARID4B,DNMT1,DNMT3A,DNMT3B,H3-3A/H3-3B,H4C1,MECP2
239	Androgen Signaling	15/136 (11%)	121/136 (89%)	2,7	0,1	AR,CACNA2D1,CCND1,DNAJB1,GNA13,GTF2H1,JUN,MAPK3,POLR2C,PRKACB,PRKCA,PRKCI,RELA,SMAD3,SRC
240	DNA damage-induced 14-3-3σ Signaling	5/19 (26%)	14/19 (74%)	2,7	0,3	AKT3,BRCA1,CCNE1,CCNE2,TP53
241	Salvage Pathways of Pyrimidine Ribonucleotides	12/98 (12%)	86/98 (88%)	2,6	0,1	ACVR2A,AICDA,CDK4,CDK6,CSNK1D,IRAK1,MAP2K1,MAP2K4,MAPK3,MAPK7,PAK5,PLK1
242	cAMP-mediated signaling	21/228 (9%)	207/228 (91%)	2,6	0,1	ADORA2B,AGTR1,AKAP8,ATF4,CREB1,DRD3,DUSP1,ENPP6,LAMTOR3,MAP2K1,MAPK3,NPR3,PDE3A,PPP3CA,PRKACB,PTGIR,RAF1,S1PR1,SRC,STAT3,TBXA2R
243	FcγRIIB Signaling in B Lymphocytes	10/75 (13%)	65/75 (87%)	2,5	0,1	CACNA2D1,GRB2,INPP5D,KRAS,MAP2K4,NRAS,PDPK1,PIK3R1,PIK3R2,PIK3R3
244	L-dopachrome Biosynthesis	2/2 (100%)	0/2 (0%)	2,5	1,0	TYR,TYRP1
245	D-myo-inositol-5-phosphate Metabolism	16/157 (10%)	141/157 (90%)	2,5	0,1	ACP1,ATP1A2,CDC25A,DUSP1,DUSP12,DUSP2,DUSP23,PPP1R7,PPP3CA,PPP5C,PTEN,PTPN11,PTPRJ,PTPRM,RASA1,SOCS3
246	Endoplasmic Reticulum Stress Pathway	5/21 (24%)	16/21 (76%)	2,5	0,2	ATF4,ATF6,CASP3,CASP7,HSP90B1
247	White Adipose Tissue Browning Pathway	14/129 (11%)	115/129 (89%)	2,5	0,1	ATF4,BDNF,CACNA2D1,CAMP,CEBPB,CREB1,FGFR1,FGFR3,MAPK11,PPARG,PRKACB,RXRA,THRB,VEGFA
248	Mechanisms of Viral Exit from Host Cells	7/41 (17%)	34/41 (83%)	2,4	0,2	ACTA2,CHMP2A,LMNB1,NEDD4,PDCD6IP,PRKCA,PRKCI
249	CREB Signaling in Neurons	19/207 (9%)	188/207 (91%)	2,4	0,1	AKT3,ATF4,CACNA2D1,CREB1,GNA13,GRB2,GRIA2,KRAS,MAP2K1,MAPK3,NRAS,PIK3R1,PIK3R2,PIK3R3,POLR2C,PRKACB,PRKCA,PRKCI,RAF1

250	Apelin Pancreas Signaling Pathway	7/44 (16%)	37/44 (84%)	2,3	0,2	APLN,MAP2K4,PIK3R1,PIK3R2,PIK3R3,PRKACB,RELA
251	CCR5 Signaling in Macrophages	11/94 (12%)	83/94 (88%)	2,3	0,1	CACNA2D1,CCR5,FAS,FASLG,FOS,JUN,MAP2K4,MAPK11,PRKCA,PRKCI,TRA
252	Cdc42 Signaling	16/167 (10%)	151/167 (90%)	2,2	0,1	ARPC3,CDC42,CFL2,FOS,GSK3B,ITGA2,ITGA5,JUN,MAP2K4,MAPK11,PRKCI,RAF1,RASA1,SRC,TRA,WIPF1
253	MIF-mediated Glucocorticoid Regulation	6/34 (18%)	28/34 (82%)	2,2	0,2	CD14,MAPK3,MIF,PTGS2,RELA,TLR4
254	Gαi Signaling	13/125 (10%)	112/125 (90%)	2,2	0,1	AGTR1,DRD3,GRB2,KRAS,MAPK3,NPR3,NRAS,PRKACB,RAF1,S1PR1,SRC,STAT3,TBXA2R
255	Granzyme B Signaling	4/16 (25%)	12/16 (75%)	2,1	0,3	APAF1,CASP3,DFFA,LMNB1
256	Ephrin A Signaling	7/47 (15%)	40/47 (85%)	2,1	0,1	CDC42,CFL2,PIK3R1,PIK3R2,PIK3R3,PTPN11,RHOA
257	Xenobiotic Metabolism AHR Signaling Pathway	10/85 (12%)	75/85 (88%)	2,1	0,1	ABCG2,CYP1A1,CYP1B1,GSTM4,HDAC4,HSP90B1,IL6,NRIP1,RELA,TNF
258	Ephrin B Signaling	9/72 (13%)	63/72 (88%)	2,1	0,1	ACP1,CDC42,CFL2,CTNNB1,CXCR4,GNA13,MAPK3,PXN,RHOA
259	Apelin Cardiomyocyte Signaling Pathway	11/99 (11%)	88/99 (89%)	2,1	0,1	AKT3,APLN,HIF1A,MAPK11,MAPK3,MAPK7,PIK3R1,PIK3R2,PIK3R3,PRKCA,PRKCI
260	Caveolar-mediated Endocytosis Signaling	9/73 (12%)	64/73 (88%)	2,1	0,1	ABL1,ACTA2,EGFR,ITGA2,ITGA5,ITGB3,PRKCA,RAB5C,SRC
261	Synaptic Long Term Potentiation	13/129 (10%)	116/129 (90%)	2,1	0,1	ATF4,CREB1,GRIA2,KRAS,MAP2K1,MAPK3,NRAS,PPP1R7,PPP3CA,PRKACB,PRKCA,PRKCI,RAF1
262	Calcium Signaling	18/206 (9%)	188/206 (91%)	2,0	0,1	ACTA2,ATF4,CACNA2D1,CREB1,GRIA2,HDAC4,MAPK3,MEF2C,MEF2D,NFATC1,PPP3CA,PRKACB,TP63,Tpm1,TPM1,TPM2,TPM3,TRPV6
263	Hematopoiesis from Pluripotent Stem Cells	7/49 (14%)	42/49 (86%)	2,0	0,1	CSF1,CXCL8,IL10,IL6,KITLG,LIF,TRA
264	Xenobiotic Metabolism PXR Signaling Pathway	17/192 (9%)	175/192 (91%)	2,0	0,1	ABCB1,CES1,GSTM4,HSP90B1,NOS2,NR1I2,NRIP1,PPP1R7,PRKACB,PRKCA,PRKCI,RXRA,SMOX,UGT2B15,UGT2B17,UGT2B28,UGT8
265	CTLA4 Signaling in Cytotoxic T Lymphocytes	10/89 (11%)	79/89 (89%)	2,0	0,1	AKT3,CTLA4,GRB2,PIK3R1,PIK3R2,PIK3R3,PPP2R2A,PPP2R5C,PTPN11,TRA
266	NER Pathway	11/103 (11%)	92/103 (89%)	2,0	0,1	CHAF1A,GTF2H1,H3-3A/H3-3B,H4C1,POLD2,POLE2,POLE3,POLE4,POLR2C,PRIM1,UBE2I
267	RhoA Signaling	12/123 (10%)	111/123 (90%)	1,8	0,1	ACTA2,ANLN,ARPC3,CFL2,GNA13,IGF1,IGF1R,NEDD4,RHOA,RTKN,SEPTIN2,SEPTIN3
268	Transcriptional Regulatory Network in Embryonic Stem Cells	7/54 (13%)	47/54 (87%)	1,8	0,1	CDX2,EOMES,GATA6,H3-3A/H3-3B,H4C1,MEIS1,STAT3
269	Intrinsic Prothrombin Activation Pathway	6/42 (14%)	36/42 (86%)	1,8	0,1	COL1A1,COL1A2,COL3A1,COL5A3,F2,KLK10
270	Cardiomyocyte Differentiation via BMP Receptors	4/20 (20%)	16/20 (80%)	1,8	0,2	BMPR1B,BMPR2,MEF2C,SMAD4
271	α-Adrenergic Signaling	10/96 (10%)	86/96 (90%)	1,8	0,1	GYS1,KRAS,MAP2K1,MAPK3,NRAS,PHKB,PRKACB,PRKCA,PRKCI,RAF1
272	Synaptic Long Term Depression	16/189 (8%)	173/189 (92%)	1,7	0,1	CACNA2D1,GNA13,GRIA2,IGF1,IGF1R,KRAS,MAP2K1,MAPK3,NOS2,NPR3,NRAS,PPP2R2A,PPP2R5C,PRKCA,PRKCI,RAF1
273	Melatonin Signaling	8/72 (11%)	64/72 (89%)	1,6	0,1	MAP2K1,MAP2K4,MAP2K7,MAPK3,PRKACB,PRKCA,PRKCI,RAF1
274	Eumelanin Biosynthesis	2/5 (40%)	3/5 (60%)	1,6	0,4	MIF,TYR
275	dTMP De Novo Biosynthesis	2/5 (40%)	3/5 (60%)	1,6	0,4	DHFR,TYMS
276	Oleate Biosynthesis II (Animals)	3/13 (23%)	10/13 (77%)	1,6	0,2	FADS1,FADS2,SCD
277	Autophagy	7/61 (11%)	54/61 (89%)	1,5	0,1	ATG3,ATG9A,BCL2,LAMP2,MTOR,SQSTM1,VPS39
278	Protein Ubiquitination Pathway	20/273 (7%)	253/273 (93%)	1,5	0,1	ANAPC1,BRCA1,DNAJB1,DNAJB4,DNAJC19,FBXW7,HSP90B1,HSPA14,HSPA1A/HSPA1B,IFNG,MDM2,NEDD4,SMO,TRAF6,UBE2I,UBE2J1,UBE2S,UBE4A,USP12,USP46
279	Antioxidant Action of Vitamin C	10/109 (9%)	99/109 (91%)	1,4	0,1	CHUK,HMOX1,IKBKE,MAP2K4,MAPK11,MAPK3,NXN,RELA,TNF,TXNRD1
280	Superpathway of Melatonin Degradation	7/65 (11%)	58/65 (89%)	1,4	0,1	CYP1A1,CYP1B1,CYP51A1,SMOX,UGT2B15,UGT2B17,UGT2B28
281	Thioredoxin Pathway	2/7 (29%)	5/7 (71%)	1,3	0,3	NXN,TXNRD1
282	Glycogen Biosynthesis II (from UDP-D-Glucose)	2/7 (29%)	5/7 (71%)	1,3	0,3	GYS1,UGP2
283	Nicotine Degradation III	6/57 (11%)	51/57 (89%)	1,2	0,1	CYP1A1,CYP1B1,CYP51A1,UGT2B15,UGT2B17,UGT2B28
284	Differential Regulation of Cytokine Production in Macrophages and T Helper Cells by IL-17A and	3/18 (17%)	15/18 (83%)	1,2	0,2	IL10,IL6,TNF
285	Xanthine and Xanthosine Salvage	1/1 (100%)	0/1 (0%)	1,2	1,0	PNP
286	FAT10 Signaling Pathway	3/18 (17%)	15/18 (83%)	1,2	0,2	IFNG,SQSTM1,TNF
287	Melatonin Degradation I	6/60 (10%)	54/60 (90%)	1,1	0,1	CYP1A1,CYP1B1,CYP51A1,UGT2B15,UGT2B17,UGT2B28
288	Inflammasome pathway	3/20 (15%)	17/20 (85%)	1,1	0,2	MYD88,PANX1,TLR4
289	Sucrose Degradation V (Mammalian)	2/9 (22%)	7/9 (78%)	1,1	0,2	ALDOA,TPI1
290	Dopamine-DARPP32 Feedback in cAMP Signaling	12/163 (7%)	151/163 (93%)	1,0	0,1	ATF4,CREB1,CSNK1D,DRD3,KCNJ16,PPP1R7,PPP2R2A,PPP2R5C,PPP3CA,PRKACB,PRKCA,PRKCI
291	Prostanoid Biosynthesis	2/10 (20%)	8/10 (80%)	1,0	0,2	PTGES2,PTGS2
292	Nicotine Degradation II	6/65 (9%)	59/65 (91%)	1,0	0,1	CYP1A1,CYP1B1,CYP51A1,UGT2B15,UGT2B17,UGT2B28
293	Apelin Adipocyte Signaling Pathway	7/82 (9%)	75/82 (91%)	1,0	0,1	APLN,GPX7,HIF1A,MAPK11,MAPK3,MAPK7,PRKACB
294	Phagosome Maturation	11/151 (7%)	140/151 (93%)	1,0	0,1	ATP6AP1L,ATP6V0A1,ATP6V1C1,ATP6V1F,LAMP2,NAPG,RAB5C,TUBB2A,VAMP3,VPS39,VTI1B
295	Differential Regulation of Cytokine Production in Intestinal Epithelial Cells by IL-17A and IL-17F	3/23 (13%)	20/23 (87%)	0,9	0,1	IFNG,IL10,TNF
296	Ethanol Degradation IV	3/23 (13%)	20/23 (87%)	0,9	0,1	ACSS1,GPX7,TYRP1
297	UDP-D-xylose and UDP-D-glucuronate Biosynthesis	1/2 (50%)	1/2 (50%)	0,9	0,5	UGDH
298	Guanine and Guanosine Salvage I	1/2 (50%)	1/2 (50%)	0,9	0,5	PNP
299	S-methyl-5'-thioadenosine Degradation II	1/2 (50%)	1/2 (50%)	0,9	0,5	MTAP
300	Spermidine Biosynthesis I	1/2 (50%)	1/2 (50%)	0,9	0,5	SRM
301	Adenine and Adenosine Salvage I	1/2 (50%)	1/2 (50%)	0,9	0,5	PNP
302	Putrescine Biosynthesis III	1/2 (50%)	1/2 (50%)	0,9	0,5	ODC1
303	Thyroid Hormone Metabolism II (via Conjugation and/or Degradation)	4/38 (11%)	34/38 (89%)	0,9	0,1	DIO3,UGT2B15,UGT2B17,UGT2B28
304	tRNA Charging	4/39 (10%)	35/39 (90%)	0,9	0,1	HARS1,MARS2,NARS1,RARS1
305	Hematopoiesis from Multipotent Stem Cells	2/12 (17%)	10/12 (83%)	0,9	0,2	CSF1,KITLG
306	Guanosine Nucleotides Degradation III	2/12 (17%)	10/12 (83%)	0,9	0,2	NT5E,PNP
307	Bupropion Degradation	3/25 (12%)	22/25 (88%)	0,9	0,1	CYP1A1,CYP1B1,CYP51A1
308	Cell Cycle Control of Chromosomal Replication	5/56 (9%)	51/56 (91%)	0,8	0,1	CDK11B,CDK19,CDK4,CDK6,PRIM1
309	NAD Salvage Pathway II	3/26 (12%)	23/26 (88%)	0,8	0,1	ACP1,ACP2,NT5E
310	Gαs Signaling	8/107 (7%)	99/107 (93%)	0,8	0,1	ADORA2B,ATF4,CREB1,MAP2K1,MAPK3,PRKACB,PTGIR,SRC
311	Estrogen Biosynthesis	4/41 (10%)	37/41 (90%)	0,8	0,1	CYP1A1,CYP1B1,CYP51A1,HSD17B12
312	Urate Biosynthesis/Inosine 5'-phosphate Degradation	2/13 (15%)	11/13 (85%)	0,8	0,2	NT5E,PNP
313	NAD Phosphorylation and Dephosphorylation	2/13 (15%)	11/13 (85%)	0,8	0,2	ACP1,ACP2
314	DNA Double-Strand Break Repair by Homologous Recombination	2/14 (14%)	12/14 (86%)	0,8	0,1	ABL1,BRCA1
315	Leukotriene Biosynthesis	2/14 (14%)	12/14 (86%)	0,8	0,1	ALOX5,GSTM4
316	Thyronamine and Iodothyronamine Metabolism	1/3 (33%)	2/3 (67%)	0,8	0,3	DIO3
317	Glutathione Biosynthesis	1/3 (33%)	2/3 (67%)	0,8	0,3	GSS
318	Colanic Acid Building Blocks Biosynthesis	2/14 (14%)	12/14 (86%)	0,8	0,1	UGDH,UGP2
319	Glycerol-3-phosphate Shuttle	1/3 (33%)	2/3 (67%)	0,8	0,3	GPD1
320	Thyroid Hormone Metabolism I (via Deiodination)	1/3 (33%)	2/3 (67%)	0,8	0,3	DIO3
321	Dopamine Receptor Signaling	6/77 (8%)	71/77 (92%)	0,8	0,1	DRD3,PPP1R7,PPP2R2A,PPP2R5C,PRKACB,SMOX
322	Adenosine Nucleotides Degradation II	2/15 (13%)	13/15 (87%)	0,7	0,1	NT5E,PNP
323	nNOS Signaling in Neurons	4/47 (9%)	43/47 (91%)	0,7	0,1	CAPN8,PPP3CA,PRKCA,PRKCI
324	Chondroitin Sulfate Degradation (Metazoa)	2/16 (13%)	14/16 (88%)	0,7	0,1	CEMIP2,HYAL3
325	Triacylglycerol Degradation	4/47 (9%)	43/47 (91%)	0,7	0,1	AARSD1,ABHD16A,CES1,LPL
326	Acetone Degradation I (to Methylglyoxal)	3/31 (10%)	28/31 (90%)	0,7	0,1	CYP1A1,CYP1B1,CYP51A1
327	Arsenate Detoxification I (Glutaredoxin)	1/4 (25%)	3/4 (75%)	0,7	0,3	PNP
328	Heme Degradation	1/4 (25%)	3/4 (75%)	0,7	0,3	HMOX1
329	Spermine and Spermidine Degradation I	1/4 (25%)	3/4 (75%)	0,7	0,3	SMOX
330	Glutathione Redox Reactions II	1/4 (25%)	3/4 (75%)	0,7	0,3	TXNDC12
331	Melatonin Degradation II	1/4 (25%)	3/4 (75%)	0,7	0,3	SMOX
332	Acetate Conversion to Acetyl-CoA	1/4 (25%)	3/4 (75%)	0,7	0,3	ACSS1
333	Kinetochore Metaphase Signaling Pathway	7/101 (7%)	94/101 (93%)	0,7	0,1	ANAPC1,AURKB,MAD2L1,PLK1,PPP1R7,PPP2R5C,SMC1A
334	Calcium-induced T Lymphocyte Apoptosis	5/66 (8%)	61/66 (92%)	0,7	0,1	MEF2D,PPP3CA,PRKCA,PRKCI,TRA
335	RAN Signaling	2/17 (12%)	15/17 (88%)	0,7	0,1	KPNA2,KPNA3
336	γ-linolenate Biosynthesis II (Animals)	2/17 (12%)	15/17 (88%)	0,7	0,1	FADS1,FADS2
337	Dermatan Sulfate Degradation (Metazoa)	2/17 (12%)	15/17 (88%)	0,7	0,1	CEMIP2,HYAL3
338	Eicosanoid Signaling	5/66 (8%)	61/66 (92%)	0,7	0,1	ALOX5,PTGES2,PTGIR,PTGS2,TBXA2R
339	Circadian Rhythm Signaling	3/33 (9%)	30/33 (91%)	0,6	0,1	ATF4,CREB1,CSNK1D
340	Purine Nucleotides Degradation II (Aerobic)	2/18 (11%)	16/18 (89%)	0,6	0,1	NT5E,PNP
341	D-myo-inositol (1,3,4)-trisphosphate Biosynthesis	2/18 (11%)	16/18 (89%)	0,6	0,1	INPP5D,PTEN
342	Protein Citrullination	1/5 (20%)	4/5 (80%)	0,6	0,2	PADI1
343	Serine Biosynthesis	1/5 (20%)	4/5 (80%)	0,6	0,2	PSAT1
344	Citrulline-Nitric Oxide Cycle	1/5 (20%)	4/5 (80%)	0,6	0,2	NOS2
345	Apelin Muscle Signaling Pathway	2/19 (11%)	17/19 (89%)	0,6	0,1	AKT3,APLN
346	OX40 Signaling Pathway	6/90 (7%)	84/90 (93%)	0,6	0,1	BCL2,BCL2L1,JUN,MAP2K4,RELA,TRA
347	Endocannabinoid Neuronal Synapse Pathway	8/128 (6%)	120/128 (94%)	0,6	0,1	CACNA2D1,GRIA2,MAPK11,MAPK3,MAPK7,PPP3CA,PRKACB,PTGS2
348	CDP-diacylglycerol Biosynthesis I	2/20 (10%)	18/20 (90%)	0,6	0,1	GPAM,LCLAT1
349	Complement System	3/37 (8%)	34/37 (92%)	0,6	0,1	C8A,CD46,CFH
350	Ceramide Biosynthesis	1/6 (17%)	5/6 (83%)	0,5	0,2	SPTLC1
351	Pentose Phosphate Pathway (Non-oxidative Branch)	1/6 (17%)	5/6 (83%)	0,5	0,2	RPIA
352	Glycerol Degradation I	1/6 (17%)	5/6 (83%)	0,5	0,2	GPD1
353	UDP-N-acetyl-D-glucosamine Biosynthesis II	1/6 (17%)	5/6 (83%)	0,5	0,2	GFPT1
354	Adenine and Adenosine Salvage III	1/6 (17%)	5/6 (83%)	0,5	0,2	PNP
355	Zymosterol Biosynthesis	1/6 (17%)	5/6 (83%)	0,5	0,2	CYP51A1
356	Phosphatidylglycerol Biosynthesis II (Non-plastidic)	2/22 (9%)	20/22 (91%)	0,5	0,1	GPAM,LCLAT1
357	Trehalose Degradation II (Trehalase)	1/7 (14%)	6/7 (86%)	0,5	0,1	HK2
358	Superpathway of Serine and Glycine Biosynthesis I	1/7 (14%)	6/7 (86%)	0,5	0,1	PSAT1
359	Purine Ribonucleosides Degradation to Ribose-1-phosphate	1/7 (14%)	6/7 (86%)	0,5	0,1	PNP
360	Superpathway of D-myo-inositol (1,4,5)-trisphosphate Metabolism	2/23 (9%)	21/23 (91%)	0,5	0,1	INPP5D,PTEN
361	Retinol Biosynthesis	3/42 (7%)	39/42 (93%)	0,5	0,1	CES1,LPL,RDH10
362	Vitamin-C Transport	2/24 (8%)	22/24 (92%)	0,5	0,1	NXN,TXNRD1
363	Salvage Pathways of Pyrimidine Deoxyribonucleotides	1/8 (13%)	7/8 (88%)	0,4	0,1	AICDA
364	Lipid Antigen Presentation by CD1	2/26 (8%)	24/26 (92%)	0,4	0,1	CD1D,TRA
365	Glycolysis I	2/26 (8%)	24/26 (92%)	0,4	0,1	ALDOA,TPI1
366	Netrin Signaling	4/65 (6%)	61/65 (94%)	0,4	0,1	CACNA2D1,NFATC1,PPP3CA,PRKACB
367	Serotonin Degradation	4/67 (6%)	63/67 (94%)	0,4	0,1	SMOX,UGT2B15,UGT2B17,UGT2B28
368	Pentose Phosphate Pathway	1/10 (10%)	9/10 (90%)	0,4	0,1	RPIA
369	Assembly of RNA Polymerase II Complex	3/50 (6%)	47/50 (94%)	0,4	0,1	GTF2H1,POLR2C,TAF9B
370	γ-glutamyl Cycle	1/11 (9%)	10/11 (91%)	0,3	0,1	GSS
371	Purine Nucleotides De Novo Biosynthesis II	1/11 (9%)	10/11 (91%)	0,3	0,1	ADSS2
372	GDP-glucose Biosynthesis	1/11 (9%)	10/11 (91%)	0,3	0,1	HK2
373	Glucose and Glucose-1-phosphate Degradation	1/12 (8%)	11/12 (92%)	0,3	0,1	HK2
374	Glycogen Degradation II	1/12 (8%)	11/12 (92%)	0,3	0,1	MTAP
375	Cholesterol Biosynthesis I	1/13 (8%)	12/13 (92%)	0,3	0,1	CYP51A1
376	Cholesterol Biosynthesis II (via 24,25-dihydrolanosterol)	1/13 (8%)	12/13 (92%)	0,3	0,1	CYP51A1
377	Cholesterol Biosynthesis III (via Desmosterol)	1/13 (8%)	12/13 (92%)	0,3	0,1	CYP51A1
378	UDP-N-acetyl-D-galactosamine Biosynthesis II	1/13 (8%)	12/13 (92%)	0,3	0,1	HK2
379	Nucleotide Excision Repair Pathway	2/35 (6%)	33/35 (94%)	0,3	0,1	GTF2H1,POLR2C
380	Glycogen Degradation III	1/14 (7%)	13/14 (93%)	0,3	0,1	MTAP
381	Phenylalanine Degradation IV (Mammalian, via Side Chain)	1/14 (7%)	13/14 (93%)	0,3	0,1	SMOX
382	Glutamate Receptor Signaling	3/57 (5%)	54/57 (95%)	0,3	0,1	GRIA2,SLC1A4,SLC38A1
383	Superpathway of Citrulline Metabolism	1/15 (7%)	14/15 (93%)	0,3	0,1	NOS2
384	Choline Biosynthesis III	1/15 (7%)	14/15 (93%)	0,3	0,1	HMOX1
385	Extrinsic Prothrombin Activation Pathway	1/16 (6%)	15/16 (94%)	0,2	0,1	F2
386	Mismatch Repair in Eukaryotes	1/16 (6%)	15/16 (94%)	0,2	0,1	MSH2
387	Antigen Presentation Pathway	2/39 (5%)	37/39 (95%)	0,2	0,1	IFNG,MR1
388	Ubiquinol-10 Biosynthesis (Eukaryotic)	1/17 (6%)	16/17 (94%)	0,2	0,1	ECHDC1
389	D-myo-inositol (1,4,5)-trisphosphate Degradation	1/17 (6%)	16/17 (94%)	0,2	0,1	INPP5D
390	1D-myo-inositol Hexakisphosphate Biosynthesis II (Mammalian)	1/18 (6%)	17/18 (94%)	0,2	0,1	INPP5D
391	Triacylglycerol Biosynthesis	2/42 (5%)	40/42 (95%)	0,2	0,0	GPAM,LCLAT1
392	tRNA Splicing	2/43 (5%)	41/43 (95%)	0,2	0,0	ENPP6,PDE3A
393	Oxidative Ethanol Degradation III	1/19 (5%)	18/19 (95%)	0,2	0,1	ACSS1
394	The Visual Cycle	1/20 (5%)	19/20 (95%)	0,2	0,1	RDH10
395	Fatty Acid α-oxidation	1/20 (5%)	19/20 (95%)	0,2	0,1	PTGS2
396	Putrescine Degradation III	1/21 (5%)	20/21 (95%)	0,2	0,0	SMOX
397	Pyrimidine Deoxyribonucleotides De Novo Biosynthesis I	1/22 (5%)	21/22 (95%)	0,2	0,0	TYMS
398	Coagulation System	1/35 (3%)	34/35 (97%)	0,0	0,0	F2
399	G Protein Signaling Mediated by Tubby	1/31 (3%)	30/31 (97%)	0,0	0,0	ABL1
400	B Cell Development	1/36 (3%)	35/36 (97%)	0,0	0,0	CD40
401	Spliceosomal Cycle	1/48 (2%)	47/48 (98%)	0,0	0,0	RBM8A
402	Heparan Sulfate Biosynthesis	1/78 (1%)	77/78 (99%)	0,0	0,0	AARSD1
403	Retinoate Biosynthesis I	1/34 (3%)	33/34 (97%)	0,0	0,0	RDH10
404	Dopamine Degradation	1/30 (3%)	29/30 (97%)	0,0	0,0	SMOX
405	Heparan Sulfate Biosynthesis (Late Stages)	1/71 (1%)	70/71 (99%)	0,0	0,0	AARSD1
406	Pyrimidine Ribonucleotides De Novo Biosynthesis	1/44 (2%)	43/44 (98%)	0,0	0,0	RAD54L
407	Glutathione Redox Reactions I	1/24 (4%)	23/24 (96%)	0,0	0,0	GPX7
408	D-myo-inositol (1,4,5)-Trisphosphate Biosynthesis	1/25 (4%)	24/25 (96%)	0,0	0,0	CDIPT
409	Phospholipases	1/64 (2%)	63/64 (98%)	0,0	0,0	HMOX1
410	Tryptophan Degradation X (Mammalian, via Tryptamine)	1/25 (4%)	24/25 (96%)	0,0	0,0	SMOX
411	Glutathione-mediated Detoxification	1/32 (3%)	31/32 (97%)	0,0	0,0	GSTM4
412	TCA Cycle II (Eukaryotic)	1/24 (4%)	23/24 (96%)	0,0	0,0	CS
413	Pyrimidine Ribonucleotides Interconversion	1/42 (2%)	41/42 (98%)	0,0	0,0	RAD54L
414	Gluconeogenesis I	1/26 (4%)	25/26 (96%)	0,0	0,0	ALDOA
415	Noradrenaline and Adrenaline Degradation	1/35 (3%)	34/35 (97%)	0,0	0,0	SMOX
416	Ethanol Degradation II	1/32 (3%)	31/32 (97%)	0,0	0,0	ACSS1
417	Superpathway of Cholesterol Biosynthesis	1/29 (3%)	28/29 (97%)	0,0	0,0	CYP51A1
418	Oxidative Phosphorylation	1/109 (1%)	108/109 (99%)	0,0	0,0	NDUFA4
419	GABA Receptor Signaling	3/95 (3%)	92/95 (97%)	0,0	0,0	CACNA2D1,GPR37,KCNN4
420	Serotonin Receptor Signaling	1/43 (2%)	42/43 (98%)	0,0	0,0	SMOX
421	Phototransduction Pathway	1/53 (2%)	52/53 (98%)	0,0	0,0	PRKACB
422	GPCR-Mediated Integration of Enteroendocrine Signaling Exemplified by an L Cell	1/73 (1%)	72/73 (99%)	0,0	0,0	PRKACB
423	GPCR-Mediated Nutrient Sensing in Enteroendocrine Cells	4/112 (4%)	108/112 (96%)	0,0	0,0	CACNA2D1,PRKACB,PRKCA,PRKCI
424	Gustation Pathway	5/154 (3%)	149/154 (97%)	0,0	0,0	CACNA2D1,ENPP6,PANX1,PDE3A,PRKACB
